# Mechanism‐Based Fluorogenic *trans*‐Cyclooctene–Tetrazine Cycloaddition

**DOI:** 10.1002/anie.201610491

**Published:** 2016-12-27

**Authors:** Arcadio Vázquez, Rastislav Dzijak, Martin Dračínský, Robert Rampmaier, Sebastian J. Siegl, Milan Vrabel

**Affiliations:** ^1^ Institute of Organic Chemistry and Biochemistry of the Czech Academy of SciencesFlemingovo nám. 2166 10PragueCzech Republic

**Keywords:** bioorthogonal chemistry, cycloaddition, click reactions, heterocycles, imaging agents

## Abstract

The development of fluorogenic reactions which lead to the formation of fluorescent products from two nonfluorescent starting materials is highly desirable, but challenging. Reported herein is a new concept of fluorescent product formation upon the inverse electron‐demand Diels–Alder reaction of 1,2,4,5‐tetrazines with particular *trans*‐cyclooctene (TCO) isomers. In sharp contrast to known fluorogenic reagents the presented chemistry leads to the rapid formation of unprecedented fluorescent 1,4‐dihydropyridazines so that the fluorophore is built directly upon the chemical reaction. Attachment of an extra fluorophore moiety is therefore not needed. The photochemical properties of the resulting dyes can be easily tuned by changing the substitution pattern of the starting 1,2,4,5‐tetrazine. We support the claim with NMR measurements and rationalize the data by computational study. Cell‐labeling experiments were performed to demonstrate the potential of the fluorogenic reaction for bioimaging.

Fluorescent probes are an indispensable tool in biological research where they are used to investigate biological processes and to interrogate biomolecules involved in them.^1^ Of particular importance are probes that become fluorescent upon binding to or reacting with the biological target of interest. These so‐called fluorogenic probes enable rapid imaging of biomolecules with excellent signal‐to‐noise ratio.^2^ Recently, the combination of bioorthogonal reactions with fluorogenic reagents enabled application of a two‐step labeling protocol for this purpose. The first step involves the introduction of a chemical reporter group to the biomolecule of interest, which is subsequently used to attach a fluorophore tag by means of a selective bioorthogonal chemical reaction.^3^ Among other chemical ligations, the inverse electron‐demand Diels–Alder reaction (IEDDA) of 1,2,4,5‐tetrazines with strained dienophiles, owing in particular to its impressive kinetics, became extremely popular as a robust transformation for biomolecule labeling.^4^ The inherent photophysical properties of the tetrazine heterocyclic core recently led to the development of fluorescent turn‐on probes where the fluorescence of the attached fluorophore moiety is quenched through energy transfer and is restored upon reaction with the dienophile.^5^ Although the advantages of such probes are obvious, the attached fluorophore makes the synthesis unnecessarily complex and in addition, the intrinsic instability of some tetrazines under biological conditions^6^ may potentially impair their structure and lead to undesired background signals. In contrast, fluorogenic probes in which the fluorophore is built during the labeling reaction provide a system with enhanced performance. Such probes, which are activated in the course of the labeling reaction, are extremely scarce and, as such, difficult to design. One example represent fluorescent pyrazolines which are built upon the 1,3‐dipolar cycloaddition of nitrile imines with various alkenes.^7^ Recently, a conceptually similar fluorogenic styrene–tetrazine cycloaddition was reported.^8^ Herein, we disclose the unusual reactivity of particular *trans*‐cyclooctenes (TCOs) in combination with 1,2,4,5‐tetrazines which enables the rapid formation of tunable fluorescent products without the need for attachment of an extra fluorophore moiety. Although the IEDDA reaction was first applied to biomolecule labeling in 2008,4a,4b and has since been extensively used in many applications,4d to the best of our knowledge this phenomenon has remained unreported.

Our study began with a startling discovery that the two TCO isomers which are formed during their photochemical synthesis^9^ react with simple dipyridyltetrazine (diPyTet) differently. In particular, the axial TCO isomer **1**, assigned as the *rel*‐(1*R*‐4*E*‐p*S*), reacted to produce with diPyTet fluorescent product, while the equatorial isomer **2**, assigned as *rel*‐(1*R*‐4*E*‐p*R*), did not (Figure 1). Analysis of the reaction mixtures by HPLC‐MS showed that the axial TCO isomer leads to formation of the expected dihydropyridazine product while the equatorial isomer produced a more polar product having a mass greater than the expected product by 18. We assigned this signal to the product of an addition of a water molecule. In addition, when we performed the same reaction using a series of other *trans*‐cyclooctenes^10^ we got similar results.^11^ This data further underlined the unique performance of the axial TCO isomer **1**.

**Figure 1:**
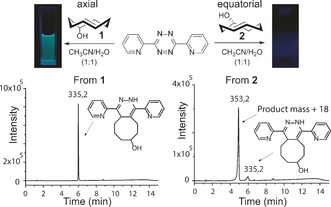
The reaction between two TCO isomers **1** and **2** and diPyTet leads to two different products. The observed product masses are shown over the HPLC signal.

To explain the differing behavior of axial and equatorial TCOs in forming fluorescent products, we monitored the reaction progress of **1** and **2** with diphenyltetrazine by NMR spectroscopy and performed a detailed analysis of the reaction mixtures (Scheme 1). The experimental data were complemented with in silico conformational analysis and density functional theory (DFT) modelling of the transition‐state structures.

**Scheme 1:**
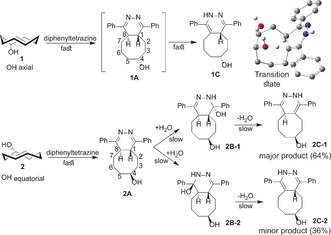
The product distribution arising from the reaction of diphenyltetrazine with the TCOs **1** and **2** as confirmed by NMR analysis. Upper right: the transition‐state connecting **1 A** and **1 C** (N‐protonated form).

The reaction of **1** with diphenyltetrazine leads to the very fast formation of a single final product (**1C**) and no intermediate could be detected (Scheme 1). We hypothesize that the initial Diels–Alder reaction leads to the 4,5‐dihydropyridazine intermediate **1A**, which isomerizes to **1C** by intramolecular hydrogen atom migration^12^ with participation of the TCO hydroxy group. The conformational analysis of **1A** shows that the hydroxy oxygen atom is very close (2.1 Å and 2.0 Å for neutral and N‐protonated molecule, respectively) to H8 in a low‐energy conformer of **1A** and a modest energy barrier of 15 kcal mol^−1^ for the hydrogen atom migration to the oxygen atom was found by DFT calculation (Scheme 1, upper right; see Figure S32 in the Supporting Information). In contrast, the equatorial TCO **2** leads to fast formation of the IEDDA product **2 A** followed by very slow hydration and dehydration reactions leading first to the intermediates **2 B** and then to final products **2 C** (Scheme 1). Two regioisomers of **2 B** and **2 C** were observed. The conformational analysis of **2 A** did not suggest any interaction between the bridgehead hydrogen atoms and the TCO hydroxy group, even in high‐energy conformers, and the reaction barrier of the intramolecular proton transfer in **2 A** is at least 10 kcal mol^−1^ higher than in **1 A**.^11^

To study the influence of various substituents on the photophysical properties of the click products we synthesized a series of tetrazines utilizing the Heck cross‐coupling methodology developed by Devaraj and co‐workers,5c and performed the click reaction with **1**.^11^ We found that the emission maxima strongly depended on the substitution pattern (Table 1; see Tables S1 and S2). In general, the presence of an electron‐withdrawing substituent in the olefinic region of the click products leads to a red shift in emission maxima. The same substituents were found to decrease the fluorescence quantum yield. All compounds have exceptionally large Stokes shifts (up to λ=240 nm) and show excellent fluorescence turn‐on properties (up to 90‐fold increase) except for the nitro substituent which abolished the fluorescence completely.^11^ We also found that the “nonconjugated” aryl substituent (phenyl, pyridyl, or thiophene) did not influence the photophysical properties significantly. In fact, this substituent is not needed for the fluorescence turn‐on and tetrazines substituted with only a methyl group in this position are fluorescent as well (data not shown). Our data demonstrate that the photophysical properties of the click products can be nicely tuned by simply changing the substituents of the starting tetrazine, thus giving instant access to a new type of fluorophores whose emissions range from *λ*=480 to 605 nm.

**Table 1:** Photophysical properties of selected click products. 
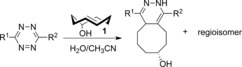

R^1^	R^2^	*λ*_Abs_/*λ*_Em_^[a]^	Stokes shift^[b]^	*Φ*_fl_/*ϵ*_max_^[c]^	Intensity increase
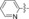	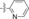	312/480	168	0.12/19	11‐fold
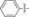	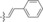	336/505	169	0.14/18	73‐fold
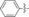	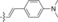	357/478	121	0.14/36	91‐fold
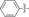	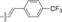	336/539	203	0.08/26	47‐fold
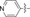	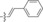	336/494	128	0.20/13	61‐fold
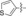	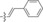	336/505	169	0.17/11	41‐fold
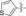	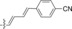	362/605	243	0.01/30	9‐fold
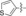	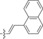	344/539	195	0.09/19	42‐fold

[a] Absorption and emission maxima were measured in CH_3_CN containing 5 % H_2_O and are given in nm. [b] Given in nm. [c] Quantum yields were determined by using quinine sulfate in 0.5 m H_2_SO_4_ as standard (*Φ*=0.55), extinction coefficients *ϵ*_max_ are in 10^3^ m ^−1^ cm^−1^.

The fluorescent products are sufficiently stable when incubated in phosphate buffered saline or fetal bovine serum, and time‐course HPLC‐MS analysis revealed the corresponding fully aromatic pyridazines as major side‐products formed under these reaction conditions over the course of time (see Figures S19–S22).

Kinetic measurements were performed to investigate the influence of the substituents on the reactivity of the tetrazines. The presence of electron‐withdrawing substituents leads to higher reaction rates in agreement with the inverse electron‐demand nature of the cycloaddition. All tetrazines reacted with **1** faster than with **2**, with second‐order rate constants ranging from 8 to 90 m ^−1^ s^−1^ (measured in CH_3_CN/H_2_O=4:1; see Table S3). As expected, further increase in reactivity was observed by increasing the water content (see Figure S18).^13^

We next studied the reaction on a model peptide (KYHWYGYTPQNVI). The ω‐amino group of the N‐terminal lysine was used to attach the tetrazine moiety by employing active ester chemistry.^11^ We next added the two TCO isomers separately to the resin and examined the beads under a fluorescent stereomicroscope. The experiment confirmed that addition of **1** leads to the rapid formation of the fluorescent product while resin beads treated with **2** only slowly start to fluoresce after a couple of hours (Figure 2).

**Figure 2:**
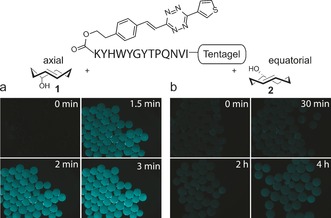
The fluorogenic click reaction was performed by using the tetrazine‐modified peptide shown above which was incubated with a) **1** or b) **2**. The pictures were captured at indicated time points and colors were adjusted using Las AF Lite program.

We also conducted the experiment in reverse order. This means that the peptide was first modified with various TCOs and then incubated with the respective tetrazine. Although this experiment validated the superior performance of the axial isomer, the difference compared to the equatorial isomer was not so significant.^11^ We also found that by attaching the TCO moiety through an electron‐withdrawing group (such as the carbamate) precluded the fluorogenic nature of the reaction. These findings fit to our theory about the involvement of the hydroxy substituent in the tautomerization of the dihydropyridazine product postulated from the computational study. However, these data also indicate that by attaching various substituents to the hydroxy group of the TCO the fluorogenic properties can be altered or may completely disappear. We believe that the mechanistic studies provided here could be helpful in the design and development of new TCO derivatives which can be further modified without perturbing their ability to form fluorescent products upon reaction with tetrazines.

To examine if the fluorogenic properties of the reaction will be preserved under biologically relevant conditions we synthesized the two probes depicted in Figure 3, which target the tetrazine moiety to different subcellular compartments. We chose Taxol because of its ability to bind and stabilize microtubules.^14^ Additionally, we synthesized a triphenylphosphonium‐derived tetrazine probe (TPP‐Tet) which is routed to mitochondria.^15^ We incubated live U2OS cancer cells with either the TPP‐Tet or Taxol‐Tet probe and subsequently added **1** to initiate the reaction. We found that **1** is cell permeable and in combination with the superb kinetics of the IEDDA reaction led to expeditious fluorescent cell labeling after only a few minutes (see Figures S26 and S27).^11^ This experiment demonstrates that the formation of the fluorescent 1,4‐dihydropyridazine product can be used for efficient and fast fluorogenic labeling of, for example, cellular compartments in live cells.

**Figure 3:**
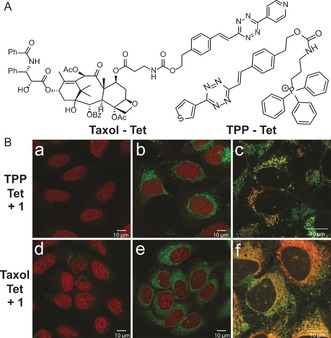
A) Structures of microtubule‐selective Taxol‐Tet probe and mitochondria‐selective TPP‐Tet probe. B) Confocal microscope images of U2OS cells treated with 5 μm TPP‐Tet or Taxol‐Tet probe. The nucleus was stained with DRAQ5 dye. a,d) negative controls (without **1**); b,e) merged channel images (after addition of TCO **1**); c,f) zoom of colocalization experiments using Mitotracker and Tubulin tracker, respectively. The images were acquired using *λ*=405 nm excitation for click products (emission window *λ*=450–550 nm). DRAQ5 and Mitotracker: ex. *λ*=633 nm, emission *λ*=667–748 nm. Tubulin tracker: ex. *λ*=561 nm, emission *λ*=600–650 nm.

In conclusion, we report on the discovery that the conformation of the TCO dienophile directly influences the formation of products in the IEDDA reaction of 1,2,4,5‐tetrazines. We found that among the dienophiles tested, only the axial TCO isomer **1** leads to the rapid formation of fluorescent 1,4‐dihydropyridazine products. The photochemical properties of the products can be easily modulated by the substitution pattern of the starting tetrazine, thus giving instant access to a new type of fluorophore with intriguing photophysical properties. The observed phenomenon was rationalized by detailed NMR analysis complemented by quantum chemical calculations. We demonstrate the practical utilization of the fluorogenic reaction for rapid labeling of intracellular compartments in live cells. We believe that the concept presented herein will lead to the development of an unrivaled class of fluorogenic tetrazine probes which will facilitate future application of the IEDDA reaction in chemical biology, materials science, diagnostics, and particularly, live‐cell imaging.

## Conflict of interest

The authors declare no conflict of interest.

## Supporting information

As a service to our authors and readers, this journal provides supporting information supplied by the authors. Such materials are peer reviewed and may be re‐organized for online delivery, but are not copy‐edited or typeset. Technical support issues arising from supporting information (other than missing files) should be addressed to the authors.

Supplementary
