# Supplementary material for: Mechanism‐Based Fluorogenic trans‐Cyclooctene–Tetrazine Cycloaddition
Source: Angew Chem Int Ed Engl. 2016 Dec 27;56(5):1334–7. doi: 10.1002/anie.201610491 (PMC5299526; doi:10.1002/anie.201610491)
Supplement: Supplementary file 1 — Supplementary [file ANIE-56-1334-s001.pdf]

Angewandte Chemie
Eine Zeitschrift der Gesellschaft Deutscher Chemiker

Supporting Information

Mechanism-Based Fluorogenic *trans*-Cyclooctene–Tetrazine Cycloaddition

*Arcadio Vázquez[+], Rastislav Dzijak[+], Martin Dračínský, Robert Rampmaier, Sebastian J. Siegl, and Milan Vrabel**

anie_201610491_sm_miscellaneous_information.pdf

Content

General information 2

HPLC analysis of the reaction of diPyTet with various TCOs 3

Synthetic procedures........ 4

Synthesis of TPP-Tet and Taxol-Tet probes 13

Synthesis of KYHWYGYTPQNVI model peptide 15

Fluorogenic click-labeling of Tetrazine-KYHWYGYTPQNVI peptide on Tentagel resin 15

Fluorogenic click-labeling of TCO-KYHWYGYTPQNVI peptides on Tentagel resin 18

Determination of fluorescence quantum yields 32

Table of photophysical properties of the click products using TCO 1 33

Copies of absorption and emission spectra of the click products using TCO 1 35

HPLC chromatograms and mass spectra of the click products using TCO 1 41

Kinetic measurements 57

Stability studies of the click products 59

Cell labeling experiments 62

NMR experiments and computational study 67

Conformational analysis 72

Cartesian coordinates of ground-state and transition-state structures 74

Copies of NMR spectra 77

References 97

General information

All reagents were purchased from Sigma-Aldrich, Alfa Aesar, ABCR, Acros or LC Laboratories (Taxol) and were used without further purification unless otherwise noted. All *trans*-cyclooctenes were synthesized using RPR-200 Rayonet photochemical reactor from *Southern New England Ultraviolet Company* equipped with 10 or 16 Hg-quartz iodine lamps 254 nm (2537 Å). To enable continuous flow set up for their synthesis a STEPDOS 03 RC membrane-metering pump from *KNF* was used. The TLC plates for analysis were from Merck silicagel 60 F_{254} and for preparative TLC were purchased from Macherey Nagel (SIL G-200, 2 mm silica layer, 200 × 200 mm, PH = 5, MF = 254, glass back). Other chromatographic purifications were conducted using 40-63 μm silicagel from Acros or on Teledyne Isco Combi*Flash* Rf200 system. The microwave reactions were performed using CEM microwave reactor. All mixtures of solvents are given in *v*/*v* ratio. ^{1}H and ^{13}C NMR spectroscopy was performed on Bruker Avance III™ HD 400 MHz and a Bruker Avance III™ HD 500 MHz. All ^{13}C NMR spectra were proton decoupled. Residual solvent peak was used as reference. Mass spectrometry was performed on AB SCIEX TripleTOF™ 5600, Thermo Fisher Scientific LTQ Orbitrap XL, Q-Tof micro (Waters) or SYNAPT G2 mass spectrometers. Fluorescence measurements were performed on Perkin Elmer LD-45 spectrophotometer equipped with a single cuvette reader. Ultraviolet absorption spectra were collected on Agilent Cary 60 spectrophotometer. HPLC experiments were performed on Shimadzu LCMS 2020 system equipped with single-quadrupole ESI-MS and PDA detector. Peptide click labeling experiments were analysed on Leica M205 fluorescent stereomicroscope equipped with pE-300^{white} LED light source and DFC3000 G grayscale camera. Cell images were acquired on Leica TCS SP5 confocal microscope equipped with HC PL APO CS2 63.0x1.40 OIL UV objective.

These compounds were prepared according to literature procedures and their analysis matched with reported data:

trans-cyclooct-4-enols **1** and **2**[1]

((2*s*,3a*R*,9a*S*,*E*)-3a,4,5,8,9,9a-hexahydrocycloocta[*d*][1,3]dioxol-2-yl)methanol[2]

(*rel*-1*R*,8*S*,9*R*,4*E*)-bicyclo[6.1.0]non-4-ene-9-ylmethanol[3]

(Z)-2-(cyclooct-4-en-1-yloxy)ethanol[4]

2-[*rel*-(1R-4E-pR)-cyclooct-4-en-1-yloxy]ethanol[5]

2-[*rel*-(1R-4E-pS)-cyclooct-4-en-1-yloxy]ethanol[5]

HPLC analysis of the reaction of diPyTet with various TCOs

Solution of dipyridyltetrazine (5 mM in CH_3CN/H_2O = 1/1) and equal amount of the corresponding TCO solution (12.5 mM, 2.5 equiv. in CH_3CN/H_2O = 1/1) were combined in HPLC vial and after 5 min measured on HPLC coupled to single quadrupole MS. We observed the formation of the fluorescent dihydropyridazine product (pictures acquired by using handheld 354 nm UV lamp irradiation) only with the axial TCO isomer while all other TCOs showed the major peak corresponding to product of addition of a molecule of water (Mol. peak + 18).

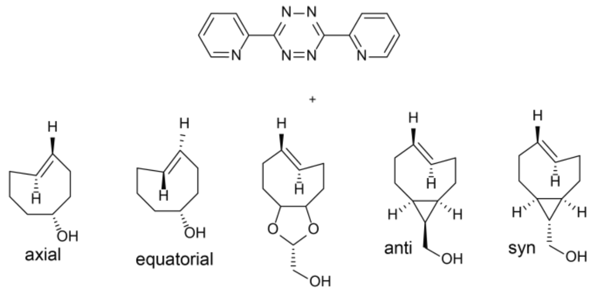

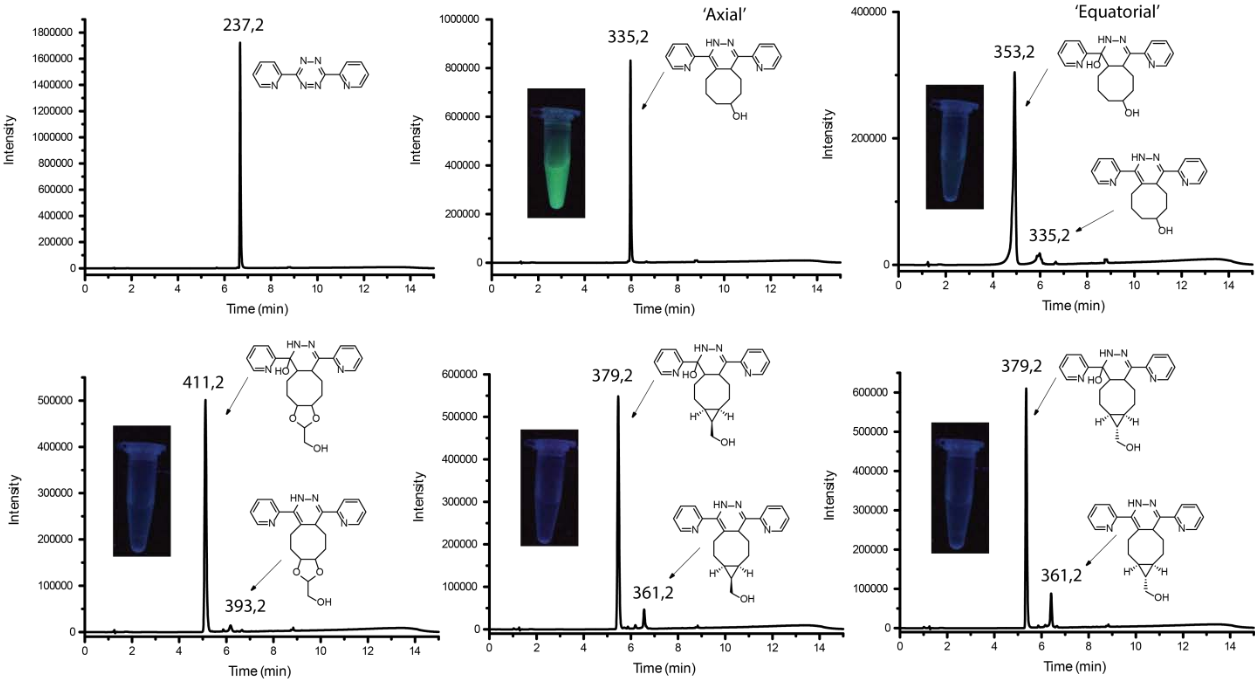

Figure S1. HPLC chromatograms of crude reaction mixtures of the reaction between dipyridyltetrazine and various TCOs. The observed molecular masses are indicated above the HPLC peaks.

Synthetic procedures

General procedure 1 (for 3-substituted-6-hydroxyethyl-1,2,4,5-tetrazines)

To a dried Schlenk tube were added in one portion under argon: aryl or heteroarylcarbonitrile, 3-hydroxypropionitrile (5 eq.), hydrazine monohydrate (10 eq.) and zinc triflate (10 mol%). The suspension was heated at 70 °C until starting material and all intermediates disappeared (24-48h, followed by HPLC-MS). The mixture was transferred to a two neck round bottom flask and a 2M solution of $NaNO_2$ in water (10 eq.) was added. Then a 2M HCl solution was added dropwise at 0 °C until no more gas evolved and the suspension became intensively red-pink in color (pH ~ 3). The red suspension was extracted with EtOAc and the organic phase was dried over Na_2SO_4, filtered and evaporated. Tetrazines were purified by silica gel column chromatography.

Synthesis of 3-(pyridin-4-yl)-6-hydroxyethyl-s-tetrazine 3A

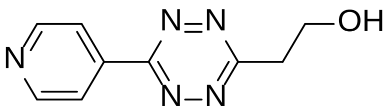

Following the general procedure 1 and using 19.2 mmol of 4-pyridinecarbonitrile, the final tetrazine was extracted with EtOAc (2x100 mL) and purified by silicagel column using CH_2Cl_2:MeOH (20:1) as eluent to afford, after recrystallization from DCM-MeOH-EtOH mixture, a pink solid (550 mg, 14%).
^{1}H NMR (400.0 MHz, $CDCl_3$): 8.94-8.92 (m, 2H), 8.46-8.44 (m, 2H), 4.34 (q, J = 5.8 Hz, 2H), 3.69 (dd, J = 6.1, 5.5 Hz, 2H), 2.31 (t, J = 6 Hz, 1H).
^{13}C NMR (100.0 MHz, $CDCl_3$): 169.6, 163.6, 151.3, 139.2, 121.4, 60.1, 37.9.
HRMS $[M]^+$ m/z calcd. for $[C_9H_9N_5O]^+$ 203.0807, found 203.0803.

The synthesis of the following compounds was performed according to general procedure 1 and their analytical data were published previously:
3-phenyl-6-hydroxyethyl-s-tetrazine[6]
3-(tiophen-3-yl)-6-hydroxyethyl-s-tetrazine[6]
(*E*)-3-phenyl-6-styryl-1,2,4,5-tetrazine[6]

General procedure 2 (for 3-substituted-6-(2-mesyl)ethyl-1,2,4,5-tetrazines)
To a 0 °C solution of 3-substituted-6-hydroxyethyl-1,2,4,5-tetrazines in anhydrous CH_2Cl_2 was added dropwise NEt_3 (1.1 eq.) and mesyl chloride (1.1 eq.) under argon. The solution was stirred at room temperature for 15 minutes. The red solution was diluted with CH_2Cl_2 and washed with H_2O. Organic phase was dried over Na_2SO_4 and solvents evaporated. Tetrazines were purified by chromatography (silica column).

Synthesis of 3-(pyridin-4-yl)- 6-(2-mesyl)ethyl-s-tetrazine 3B

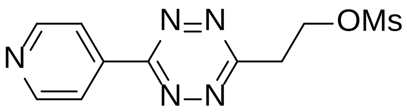

Following the general procedure 2 using 1.23 mmol of 3-(pyridin-4-yl)-6-hydroxyethyl-s-tetrazine, once reaction was finished, CH_2Cl_2 (50 mL) was added and organic phase was washed with H_2O (2x50 mL) and purified by silicagel column using Hexane:EtOAc (1:1) as eluent to afford a pink solid (270 mg, 78%).
^{1}H NMR (400.0 MHz, $CDCl_3$): 8.94-8.92 (m, 2H), 8.46-8.44 (m, 2H), 4.94 (t, *J* = 6.1 Hz, 2H), 3.89 (t, *J* = 6.1 Hz, 2H), 3.69 (q, *J* = 5.8 Hz, 2H), 3.04 (s, 3H).
^{13}C NMR (100.0 MHz, $CDCl_3$): 167.5, 163.8, 151.4, 139.0, 121.5, 65.8, 37.8, 35.1.
HRMS $[M+Na]^+$ m/z calcd. for $[C_{10}H_{11}N_5O_3SNa]^+$ 304.04748, found 304.04761.

The synthesis of the following compounds was performed according to general procedure 2 and their analytical data were published previously:
Synthesis of 3-phenyl-6-(2-mesyl)ethyl-s-tetrazine[6]
Synthesis of 3-(tiophen-3-yl)-6-(2-mesyl)ethyl-s-tetrazine[6]

General procedure 3 (for 3-substituted-6-alkenyl-1,2,4,5-tetrazines)
To a 10 mL microwave reaction tube were added all solid reagents: mesylated tetrazine derivatives (50 mg), $Pd_2(dba)_3$ (10 mol%), P(*o*-Tol)$_3$ (40 mol%) and aryl or heteroarylhalide (1.5 eq.). The flask was purged with argon (vacuum-argon cycles) and *N*,*N*-dicyclohexylmethylamine (3 eq.) and anhydrous DMF (to get final 0.1 M solution) were added. The mixture was heated in microwave reactor for the indicated time. The reaction mixture was cooled to room temperature and water (50 mL) was added and the mixture was extracted with ethyl acetate (2x50 mL). Organic phase was dried over Na_2SO_4 and solvents were evaporated. Products were purified by chromatography (silica column, flash or preparative TLC).

Synthesis of 3C

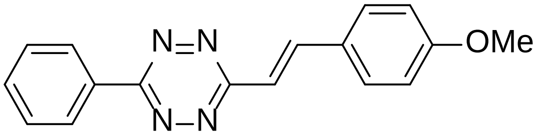

Following the general procedure 3, 3-phenyl-6-(2-mesyl)ethyl-s-tetrazine and 4-iodoanisole were used and the suspension was irradiated for 40 min at 50 °C. The product was isolated using Hexane:EtOAc (6:1) as eluent for chromatography. Red solid (22 mg, 43%).
^{1}H NMR (400.0 MHz, $CDCl_3$): 8.59-8.62 (m, 2H), 8.32 (d, *J* = 16 Hz, 1H) , 7.65-7.67 (m, 2H), 7.58-7.62 (m, 3H), 7.38 (d, *J* = 16 Hz, 1H), 6.97-6.99 (m, 2H), 3.87 (s, 3H).
^{13}C NMR (100.0 MHz, $CDCl_3$): 165.1, 163.0, 161.6, 141.0, 132.5, 132.2, 130.0, 129.4, 128.1, 127.9, 118.3, 114.6, 55.6.
HRMS $[M]^+$ m/z calcd. for $[C_{17}H_{14}N_4O]^+$ 290.1168, found 290.1169.

Synthesis of 3D

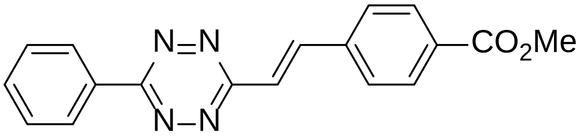

Following the general procedure 3, 3-phenyl-6-(2-mesyl)ethyl-s-tetrazine and methyl 4-iodobenzoate were used and the suspension was irradiated for 80 min at 50 °C. The product was isolated using CH_2Cl_2 as eluent for chromatography. Pink solid (20 mg, 35%).
^{1}H NMR (400.0 MHz, $CDCl_3$): 8.64-8.62 (m, 2H), 8.38 (d, J = 16 Hz, 1H), 8.14-8.11 (m, 2H), 7.79-7.76 (m, 2H,), 7.65-7.59 (m, 4H), 3.95 (s, 3H).
^{13}C NMR (100.0 MHz, $CDCl_3$): 166.6, 164.6, 163.3, 139.8, 139.5, 132.9, 131.9, 131.4, 130.4, 129.5, 128.2, 128.1, 123.2, 52.5.
HRMS $[M+Na]^+$ m/z calcd. for $[C_{18}H_{14}N_4O_2Na]^+$ 341.10090, found 341.10092.

Synthesis of 3E

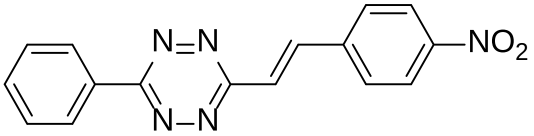

Following the general procedure 3, 3-phenyl-6-(2-mesyl)ethyl-s-tetrazine and 1-iodo-4-nitrobenzene were used and the suspension was irradiated for 80 min at 50 °C. The product was isolated using CH_2Cl_2 as eluent for chromatography. Red solid (15 mg, 28%).
^{1}H NMR (500.0 MHz, DMSO-d_6): 8.53 (m, 2H), 8.42 (d, J_{trans} = 16.3 Hz, 1H), 8.32 (m, 2H), 8.22 (m, 2H), 7.97 (d, J_{trans} = 16.3 Hz, 1H), 7.75-7.67 (m, 3H).
^{13}C NMR (125.7 MHz, DMSO-d_6): 164.2, 162.7, 148.0, 141.6, 137.8, 132.1, 132.9, 129.7, 129.6, 127.8, 125.7, 124.3.
HRMS $[M]^+$ m/z calcd. for $[C_{16}H_{11}N_5O_2]^+$ 305.0913, found 305.0911.

Synthesis of 3F

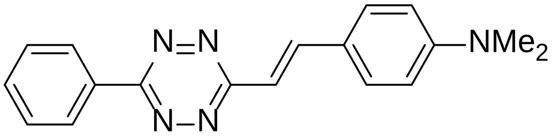

Following the general procedure 3, 3-phenyl-6-(2-mesyl)ethyl-s-tetrazine and 1-bromo-*N*,*N*-dimethyaniline were used and the suspension was irradiated for 45 min at 50 °C. The product was isolated using Hexane:EtOAc (10:1) as eluent for chromatography. Orange solid (45 mg, 83%).
^{1}H NMR (400.0 MHz, $CDCl_3$): 8.60-8.58 (m, 2H), 8.30 (d, J = 16 Hz, 1H), 7.63-7.58 (m, 5H), 7.29 (d, J = 16 Hz, 1H), 6.81-6.79 (m, 2H), 3.07 (s, 6H).
^{13}C NMR (100.0 MHz, $CDCl_3$): 165.4, 162.6, 141.7, 132.4, 132.2, 130.1, 129.3, 127.7, 40.7.
HRMS $[M]^+$ m/z calcd. for $[C_{18}H_{18}N_5]^+$ 304.15567, found 304.15580.

Synthesis of 3G

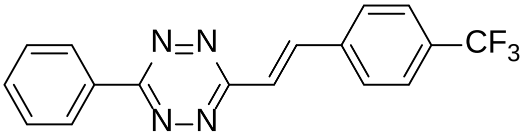

Following the general procedure 3, 3-phenyl-6-(2-mesyl)ethyl-s-tetrazine and 4-iodobenzotrifluoride were used and the suspension was irradiated for 3 hours at 50 °C. The product was isolated using Hexane:EtOAc (15:1) as eluent for chromatography. Pink solid (45 mg, 77%).

^{1}H NMR (400.0 MHz, $CDCl_3$): 8.65-8.62 (m, 2H), 8.38 (d, *J* = 16 Hz, 1H), 7.82 (d, *J* = 8 Hz, 2H), 7.72 (d, *J* = 8 Hz, 2H), 7.65-7.59 (m, 4H).
^{13}C NMR (100.0 MHz, $CDCl_3$): 164.5, 163.4, 139.3, 138.6, 133.0, 132.0, 131.9, 131.7, 129.5, 128.4, 128.2, 126.2 (q), 123.3.
HRMS $[M]^+$ m/z calcd. for $[C_{17}H_{11}N_4F_3]^+$ 328.0936, found 328.0936.

Synthesis of 3H

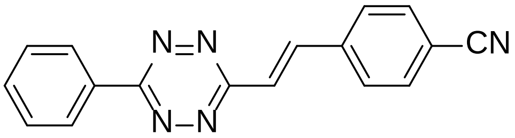

Following the general procedure 3, 3-phenyl-6-(2-mesyl)ethyl-s-tetrazine and 4-iodobenzonitrile were used and the suspension was irradiated for 90 min at 60 °C. The product was isolated using CH_2Cl_2 as eluent for chromatography. Pink solid (32 mg, 63%).
^{1}H NMR (400.0 MHz, $CDCl_3$): 8.65-8.62 (m, 2H), 8.34 (d, *J* = 16 Hz, 1H), 7.81-7.74 (m, 4H), 7.65-7.59 (m, 4H).
^{13}C NMR (100.0 MHz, $CDCl_3$): 164.3, 163.4, 139.5, 138.6, 133.0, 132.9, 131.8, 129.5, 128.6, 128.3, 124.4, 118.6, 113.4.
HRMS $[M+Na]^+$ m/z calcd. for $[C_{17}H_{11}N_5Na]^+$ 308.09067, found 308.09008.

Synthesis of 3I

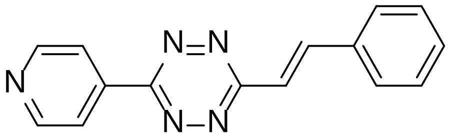

Following the general procedure 3, 3-(pyridin-4-yl)-6-(2-mesyl)ethyl-s-tetrazine and iodobenzene were used and the suspension was irradiated for 35 min at 50 °C. The product was isolated using Hexane:EtOAc (1:1) as eluent for chromatography. Red solid (41 mg, 88%).
^{1}H NMR (400.0 MHz, $CDCl_3$): 8.92-8.90 (m, 2H), 8.47-8.43 (m, 3H), 7.74-7.72 (m, 2H), 7.56 (d, *J* = 16 Hz, 1H), 7.50-7.45 (m, 3H).
^{13}C NMR (100.0 MHz, $CDCl_3$): 165.6, 162.0, 151.2, 143.0, 139.5, 135.0, 130.9, 129.3, 128.5, 121.2, 120.3.
HRMS $[M]^+$ m/z calcd. for $[C_{15}H_{11}N_5]^+$ 261.1014, found 261.1013.

Synthesis of 3J

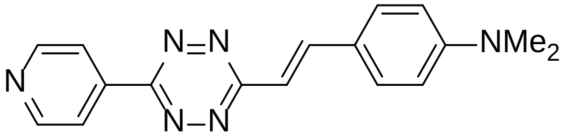

Following the general procedure 3, 3-(pyridin-4-yl)-6-(2-mesyl)ethyl-s-tetrazine and 1-bromo-*N*,*N*-dimethyaniline were used and the suspension was irradiated for 45 min at 50 °C. The product was isolated using CH_2Cl_2 as eluent for chromatography. Pink solid (33 mg, 61%).
^{1}H NMR (401 MHz, DMSO-d_6) δ 8.93-8.84 (m, 2H), 8.38 -8.31 (m, 2H), 8.27 (d, *J* = 16.0 Hz, 1H), 7.82-7.69 (m, 2H), 7.39 (d, *J* = 16.0 Hz, 1H), 6.86-6.72 (m, 2H), 3.03 (s, 6H).

^{13}C NMR (125.7 MHz, DMSO-d_6): 165.7, 160.9, 152.1, 151.1, 142.7, 139.8, 130.6, 122.6, 120.3, 114.5, 112.1, 39.9.
HRMS $[M+H]^+$ m/z calcd. for $[C_{17}H_{17}N_6]^+$ 305.15092, found 305.15093.

Synthesis of 3K

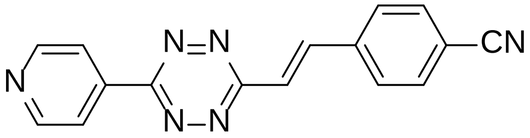

Following the general procedure 3, 3-(pyridin-4-yl)-6-(2-mesyl)ethyl-s-tetrazine and 4-iodobenzonitrile were used and the suspension was irradiated for 2 hours at 60 °C. The product was isolated using Hexane:EtOAc (1:1) as eluent for chromatography. Red solid (30 mg, 59%).
^{1}H NMR (400.0 MHz, $CDCl_3$): 8.95-8.91 (m, 2H) , 8.49-8.46 (m, 2H), 8.43 (d, J = 16 Hz, 1H) , 7.86-7.73 (m, 4H), 7.66 (d, J = 16 Hz, 1H).
^{13}C NMR (125.7 MHz, $CDCl_3$): 165.2, 162.3, 151.2, 140.3, 139.2, 139.1, 133.0, 128.8, 123.9, 121.4, 118.5, 113.8.
HRMS $[M]^+$ m/z calcd. for $[C_{16}H_{10}N_6]^+$ 286.0967, found 286.0968.

Synthesis of 3L

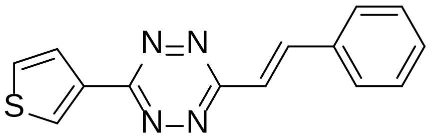

Following the general procedure 3, 3-(tiophen-3-yl)-6-(2-mesyl)ethyl-s-tetrazine and iodobenzene were used and the suspension was irradiated for 45 min at 60 °C. The product was isolated using Hexane:EtOAc (10:1) as eluent for chromatography. Red solid (33 mg, 71%).
^{1}H NMR (400.0 MHz, $CDCl_3$): 8.61 (dd, J = 3.2, 1.2 Hz, 1H), 8.33 (d, J = 16 Hz, 1H), 8.07 (dd, J = 5.2, 1.2 Hz, 1H), 7.72-7.69 (m, 2H), 7.54-7.52 (m, 2H), 7.48-7.42 (m, 3H).
^{13}C NMR (100.0 MHz, $CDCl_3$): 164.4, 160.9, 141.0, 135.3, 135.2, 130.4, 130.0, 129.2, 128.2, 127.6, 126.7, 120.9.
HRMS $[M+H]^+$ m/z calcd. for $[C_{14}H_{11}N_4S]^+$ 267.06989, found 267.06991.

Synthesis of 3M

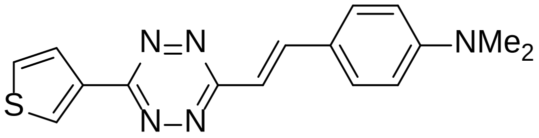

Following the general procedure 3, 3-(tiophen-3-yl)-6-(2-mesyl)ethyl-s-tetrazine and 1-bromo-*N*,*N*-dimethyaniline were used and the suspension was irradiated for 45 min at 60 °C. The product was isolated using Hexane:EtOAc (10:1) as eluent for chromatography. Brown-red solid (33 mg, 61%).
^{1}H NMR (400.0 MHz, $CDCl_3$): 8.55-8.54 (m, 1H), 8.26 (d, J = 16 Hz, 1H) , 8.05-8.04 (m, 1H) , 7.61-7.57 (m, 2H) , 7.51-7.49 (m, 1H), 7.24 (d, J = 16 Hz, 1H), 6.74-6.72 (m, 2H), 3.06 (s, 6H).
^{13}C NMR (100.0 MHz, $CDCl_3$): , 165.0, 160.3, 151.9, 141.5, 135.5, 130.0, 129.0, 127.4, 126.6, 123.4, 115.3, 112.1, 40.3.

HRMS $[M+H]^+$ m/z calcd. for $[C_{16}H_{16}N_5S]^+$ 310.11209, found 310.11213.

Synthesis of 3N

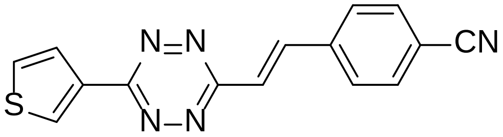

Following the general procedure 3, 3-(tiophen-3-yl)-6-(2-mesyl)ethyl-s-tetrazine and 4-iodobenzonitrile were used and the suspension was irradiated for 90 min at 70 °C. The product was isolated using Hexane:EtOAc (2:1) as eluent for chromatography. Red solid (15 mg, 30%).

^{1}H NMR (400.0 MHz, $CDCl_3$): 8.65 (dd, *J* = 3.0, 1.2 Hz, 1H), 8.31 (d, *J* = 16.3 Hz, 1H) , 8.08 (dd, *J* = 5.1, 1.2 Hz, 1H) , 7.80-7.73 (m, 4H), 7.59 (d, *J* = 16.3 Hz, 1H), 7.54 (dd, *J* = 5.1, 3.0 Hz, 1H).

^{13}C NMR (100.0 MHz, $CDCl_3$): 163.8, 161.0, 139.5, 138.3, 134.9, 132.9, 130.7, 128.5, 127.8, 126.7, 124.4, 118.6, 113.4.

HRMS $[M+H]^+$ m/z calcd. for $[C_{16}H_{10}N_5S]^+$ 292.06514, found 292.06518.

Synthesis of 3O

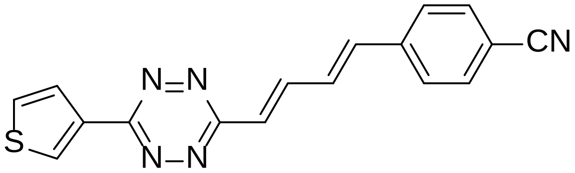

Following the general procedure 3, 3-(tiophen-3-yl)-6-(2-mesyl)ethyl-s-tetrazine and 4-[(1*E*)-2-iodoethenyl]benzonitrile[7] were used and the suspension was irradiated for 60 min at 70 °C. The product was isolated using CH_2Cl_2:Hexane (4:1) as eluent for chromatography. Pink solid (15 mg, 27%).

^{1}H NMR (500.0 MHz, DMSO-d_6, T = 100°C): 7.23 (d, 1H, ^3J = 15.7, C3-CH=CH=CH=C**H**-C*i*); 7.24 (dd, 1H, $^3J_{vic}$ = 15.5, ^4J = 0.9, C3-C**H**=CH=CH=CH-C*i*); 7.50 (ddd, 1H, $^3J_{vic}$ = 15.7, 10.9, ^4J = 0.9, C3-CH=CH=C**H**=CH-C*i*); 7.77 – 7.83 (m, 5H, H-5-thienyl, H-*o,m*-C_6H_4CN); 7.97 (dd, 1H, $J_{4,5}$ = 5.1, $J_{4,2}$ = 1.3, H-4-thienyl); 8.01 (ddd, 1H, $^3J_{vic}$ = 15.5, 10.9, ^4J = 0.9, C3-CH=C**H**=CH=CH-C*i*); 8.70 (dd, 1H, $J_{2,5}$ = 3.0, $J_{2,4}$ = 1.3, H-2-thienyl).

^{13}C NMR (125.7 MHz, DMSO-d_6, T = 100°C): 110.58 (C-*p*-C_6H_4CN); 118.21 (CN); 125.77 (CH-4-thienyl); 126.08 (C3-**C**H=CH=CH=CH-C*i*); 127.44 (CH-*o*-C_6H_4CN); 128.19 (CH-5-thienyl); 129.96 (CH-2-thienyl); 130.97 (C3-CH=CH=**C**H=CH-C*i*); 132.22 (CH-*m*-C_6H_4CN); 134.22 (C-3-thienyl); 136.94 (C3-CH=CH=CH=**C**H-C*i*); 139.38 (C3-CH=**C**H=CH=CH-C*i*); 140.58 (C-*i*-C_6H_4CN); 159.77 (C-6-tetrazin); 163.45 (C-3-tetrazin).

HRMS $[M+H]^+$ m/z calcd. for $[C_{17}H_{12}N_5S]^+$ 318.08079, found 318.08082.

Synthesis of 3P

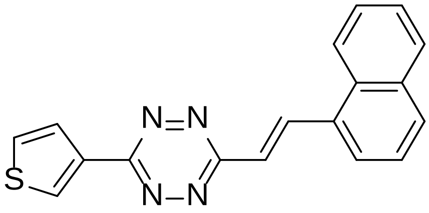

Following the general procedure 3, 3-(tiophen-3-yl)-6-(2-mesyl)ethyl-s-tetrazine and 1-iodonaphthalene were used and the suspension was irradiated for 60 min at 60 °C. The product was isolated using Hexane:Diethylether (4:1) as eluent for chromatography. Red solid (17 mg, 31%).

^{1}H NMR (400.0 MHz, $CDCl_3$): 9.20 (d, *J* = 16.3 Hz, 1H), 8.63 (dd, *J* = 3.0, 1.2 Hz, 1H), 8.35-8.33 (m, 1H), 8.10 (dd, *J* = 5.1, 1.2 Hz, 1H), 8.02-7.99 (m, 1H), 7.92 (m, 2H), 7.64-7.53 (m, 5H).

^{13}C NMR (100.0 MHz, $CDCl_3$): 164.4, 160.9, 137.7, 135.2, 133.9, 132.6, 131.7, 130.8, 130.1, 129.0, 127.6, 127.1, 126.7, 126.4, 125.7, 125.1, 123.5, 123.2.

HRMS $[M+H]^+$ m/z calcd. for $[C_{18}H_{13}N_4S]^+$ 317.08554, found 317.08566.

Synthesis of 3Q

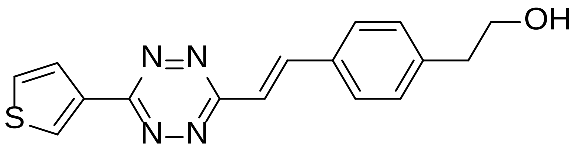

Following the general procedure 3, 3-(tiophen-3-yl)-6-(2-mesyl)ethyl-s-tetrazine and 4-bromophenethyl alcohol were used and the suspension was irradiated for 60 min at 80 °C. The product was isolated using CH_2Cl_2:MeOH (20:1) as eluent for chromatography. Red solid after preparative TLC (38 mg, 70%).

^{1}H NMR (401 MHz, DMSO-*d*$_6$) δ 8.74 (dd, *J* = 3.0, 1.3 Hz, 1H), 8.21 (d, *J* = 16.3 Hz, 1H), 7.96 (dd, *J* = 5.1, 1.3 Hz, 1H), 7.85 (dd, *J* = 5.1, 3.0 Hz, 1H), 7.81 – 7.75 (m, 2H), 7.58 (d, *J* = 16.3 Hz, 1H), 7.37 – 7.28 (m, 2H), 4.69 (t, *J* = 5.2 Hz, 1H), 3.65 (td, *J* = 6.9, 5.2 Hz, 2H), 2.78 (t, *J* = 6.9 Hz, 2H).

^{13}C NMR (101 MHz, DMSO) δ 163.9, 160.2, 142.3, 139.9, 134.8, 132.7, 130.3, 129.6, 128.8, 128.2, 126.0, 120.1, 61.9, 38.9.

HRMS $[M+H]^+$ m/z calcd. for $[C_{16}H_{15}ON_4S]^+$ 311.09611, found 311.09623.

Synthesis of 3R

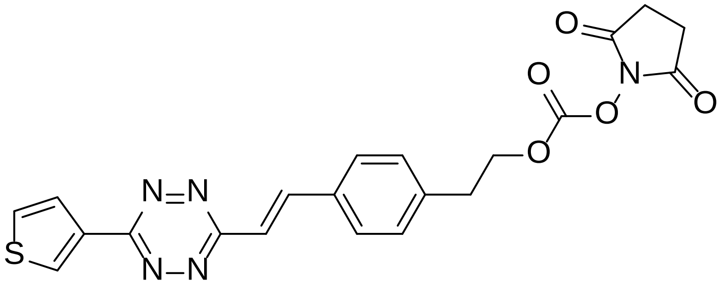

To a 0 °C suspension of **3Q** (22 mg, 0.07 mmol) in anhydrous acetonitrile (2 mL) triethylamine (0.04 mL, 0.284 mmol) and *N*,*N*'-disuccinimidyl carbonate (36 mg, 0.142 mmol) were added. The mixture was stirred at room temperature until the starting material was consumed (HPLC-MS). The precipitate was filtered and washed with CH_3CN to afford part of the product as a red solid. The acetonitrile solution was evaporated and the residue was purified by column chromatography CH_2Cl_2:MeOH (20:1) to get more of the final compound (in total 20mg 63 % yield).

^{1}H NMR (401 MHz, DMSO-*d*$_6$) δ 8.76 (d, *J* = 3.0 Hz, 1H), 8.24 (d, *J* = 16.3 Hz, 1H), 7.98 (d, *J* = 5.1 Hz, 1H), 7.86 (d, *J* = 7.2 Hz, 3H), 7.65 (d, *J* = 16.3 Hz, 1H), 7.41 (d, *J* = 7.8 Hz, 2H), 4.60 (t, *J* = 6.6 Hz, 2H), 3.09 (t, *J* = 6.6 Hz, 2H), 2.80 (s, 4H).

^{13}C NMR (101 MHz, DMSO) δ 169.9, 163.9, 160.2, 151.1, 139.6, 139.3, 134.8, 133.6, 130.4, 129.6, 128.9, 128.5, 126.1, 120.7, 71.2, 33.8, 25.3.
HRMS $[M+H]^+$ m/z calcd. for $[C_{21}H_{17}O_5N_5NaS]^+$ 474.08426, found 474.08413.

Synthesis of 3S

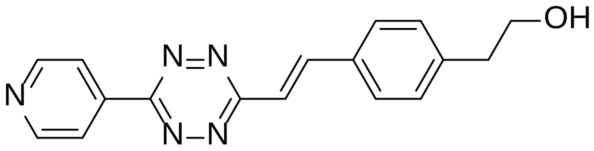

Following the general procedure 3, 3-(pyridin-4-yl)-6-(2-mesyl)ethyl-s-tetrazine and 4-bromophenethyl alcohol were used and the suspension was irradiated for 60 min at 80 °C. The product was isolated using CH_2Cl_2:MeOH (20:1) as eluent for chromatography. Red solid (30 mg, 19%).
^{1}H NMR (400 MHz, DMSO-d_6) δ 9.00-8.81 (m, 1H), 8.40-8.27 (m, 3H), 7.90-7.76 (m, 2H), 7.69 (d, *J* = 16.3 Hz, 1H), 7.35 (d, *J* = 8.1 Hz, 2H), 4.69 (t, *J* = 5.2 Hz, 1H), 3.65 (td, *J* = 6.9, 5.2 Hz, 2H), 2.79 (t, *J* = 6.9 Hz, 2H).
^{13}C NMR (101 MHz, DMSO) δ 165.0, 161.3, 151.0, 142.8, 141.6, 139.5, 132.6, 129.7, 128.4, 120.9, 119.9, 61.8, 38.9.
HRMS $[M+H]^+$ m/z calcd. for $[C_{17}H_{16}ON_5]^+$ 306.13494, found 306.13511.

Synthesis of 3T

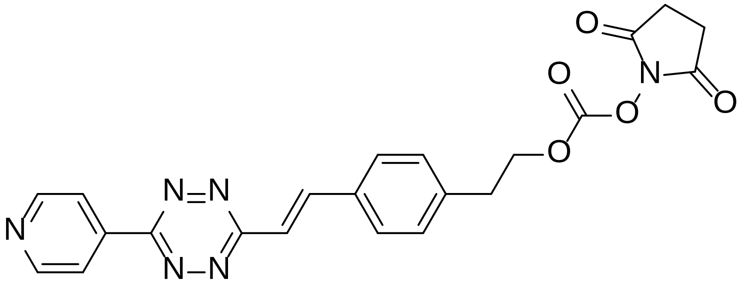

To a 0 °C suspension of **3S** (40 mg, 0.13 mmol) in anhydrous DMF (4 mL) triethylamine (0.07 mL, 0.524 mmol) and *N*,*N*'-disuccinimidyl carbonate (67 mg, 0.262 mmol) were added. The mixture was stirred at room temperature until the starting material was consumed. Solvent was evaporated and the residue was purified by chromatography column CH_2Cl_2:MeOH (20:1) to get a red solid (47 mg, 79%).
^{1}H NMR (400 MHz, $CDCl_3$-*d*) δ 8.91 (d, *J* = 5.2 Hz, 2H), 8.62-8.34 (m, 3H), 7.70 (d, *J* = 7.9 Hz, 2H), 7.54 (d, *J* = 16.2 Hz, 1H), 7.35 (d, *J* = 7.7 Hz, 2H), 4.55 (t, *J* = 6.8 Hz, 2H), 3.13 (t, *J* = 6.8 Hz, 2H), 2.84 (s, 4H).
^{13}C NMR (101 MHz, $CDCl_3$) δ 168.7, 165.6, 162.0, 151.6, 151.1, 142.6, 139.5, 139.4, 133.9, 129.9, 128.9, 121.3, 120.2, 71.2, 34.9, 25.6.
HRMS $[M+H]^+$ m/z calcd. for $[C_{22}H_{19}O_5N_6]^+$ 447.14114, found 447.14125.

Synthesis of 4A

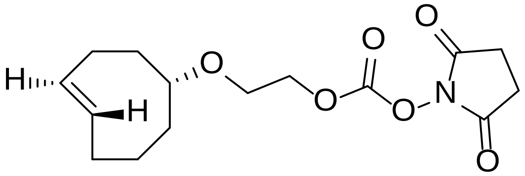

To a 0 °C solution of 2-[*rel*-(1R-4E-pR)-cyclooct-4-en-1-yloxy]ethanol (55 mg, 0.323 mmol) and triethylamine (0.18 mL, 1.29 mmol) in anhydrous CH_3CN (1 mL) was added in one portion *N*,*N*'-disuccinimidyl carbonate (165mg, 0.646mmol, 2 equiv.). After one hour at room temperature the solvent was evaporate and the residue was purified by silica column using Hexane:Diethylether as eluent (1:2) to afford a colorless liquid (70mg, 81%).

^{1}H NMR (401 MHz, $CDCl_3$-*d*) δ 5.56 (ddd, *J* = 15.9, 11.1, 3.6 Hz, 1H), 5.37 (ddd, *J* = 16.0, 11.0, 3.6 Hz, 1H), 4.38 (t, *J* = 4.8 Hz, 2H), 3.67-3.60 (m, 1H), 3.58-3.51 (m, 1H), 3.03 (ddt, *J* = 10.6, 5.1, 1.4 Hz, 1H), 2.81 (s, 4H), 2.41-2.31 (m, 2H), 2.30-2.16 (m, 1H), 2.11-2.03 (m, 1H), 2.01-1.75 (m, 4H), 1.58-1.44 (m, 2H).

^{13}C NMR (101 MHz, $CDCl_3$) δ 168.7, 151.8, 135.5, 132.4, 86.5, 70.6, 65.3, 40.7, 37.7, 34.6, 33.0, 31.8, 25.6.

HRMS $[M+H]^+$ m/z calcd. for $[C_{15}H_{22}O_6N]^+$ 312.14416, found 312.14411.

Synthesis of 4B

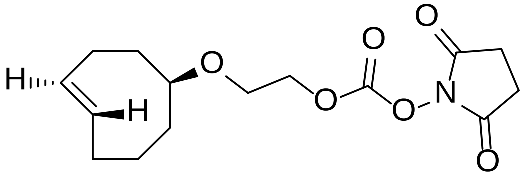

To a 0 °C solution of 2-[*rel*-(1R-4E-pS)-cyclooct-4-en-1-yloxy]ethanol (48 mg, 0.282 mmol) and triethylamine (0.16 mL, 1.13 mmol) in anhydrous CH_3CN (1 mL) was added in one portion *N*,*N*'-disuccinimidyl carbonate (145mg, 0.564mmol). After one hour at room temperature the solvent was evaporated and the residue was purified by silica column using Hexane:Diethylether as eluent (1:2) to afford a colorless liquid (71mg, 95%).

^{1}H NMR (400 MHz, $CDCl_3$-*d*) δ 5.69-5.56 (m, 1H), 5.46 (ddd, *J* = 16.0, 10.8, 3.4 Hz, 1H), 4.54-4.43 (m, 2H), 3.76-3.67 (m, 1H), 3.65-3.54 (m, 2H), 2.84 (s, 4H), 2.37-2.23 (m, 3H), 2.17 (dddd, *J* = 16.0, 10.1, 6.0, 1.2 Hz, 1H), 2.07-1.97 (m, 1H), 1.85-1.68 (m, 3H), 1.48 (dddd, *J* = 15.1, 13.6, 4.6, 1.2 Hz, 1H), 1.23-1.17 (m, 1H).

^{13}C NMR (101 MHz, $CDCl_3$): δ 168.7, 151.9, 136.2, 131.1, 75.5, 70.7, 66.0, 40.1, 34.6, 33.1, 29.8, 27.6, 25.6.

HRMS $[M+H]^+$ m/z calcd. for $[C_{15}H_{22}O_6N]^+$ 312.14416, found 312.14424.

Synthesis of TPP-Tet and Taxol-Tet probes

Synthesis of TPP-Tet probe

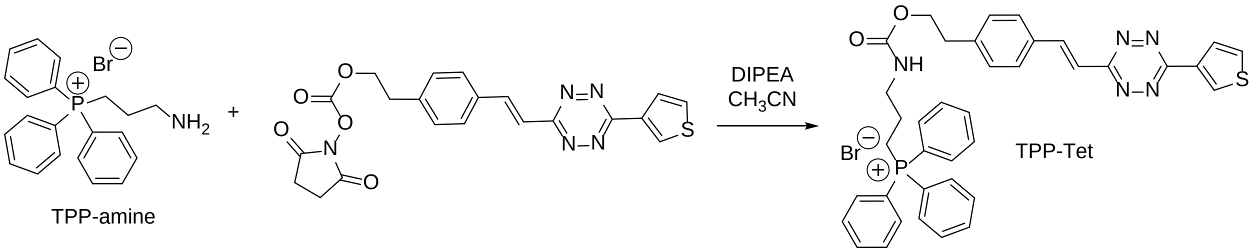

The NHS active ester **3R** (8 mg, 0.018 mmol) and TPP-amine[8] (9 mg, 0.022 mmol, 1.25 equiv) were dissolved in CH_3CN and cooled on ice-water bath. The flask was purged with argon and DIPEA (11 µL, 3,5 equiv) was added. The reaction mixture was stirred at room temperature until starting tetrazine disappeared as followed by HPLC-MS and/or TLC (DCM/MeOH = 9/1). The crude reaction mixture was concentrated on rotary evaporator, dissolved in minimum amount of DCM and the product was isolated by preparative TLC using DCM/MeOH = 9/1 as eluent. Isolated: 7 mg, yield 54%. The purity and identity of the product was verified by HPLC-MS (shown below) and HRMS.
HRMS $[M]^+$ m/z calcd. for $[C_{38}H_{35}O_2N_5PS]^+$ 656.2244, found 656.2245

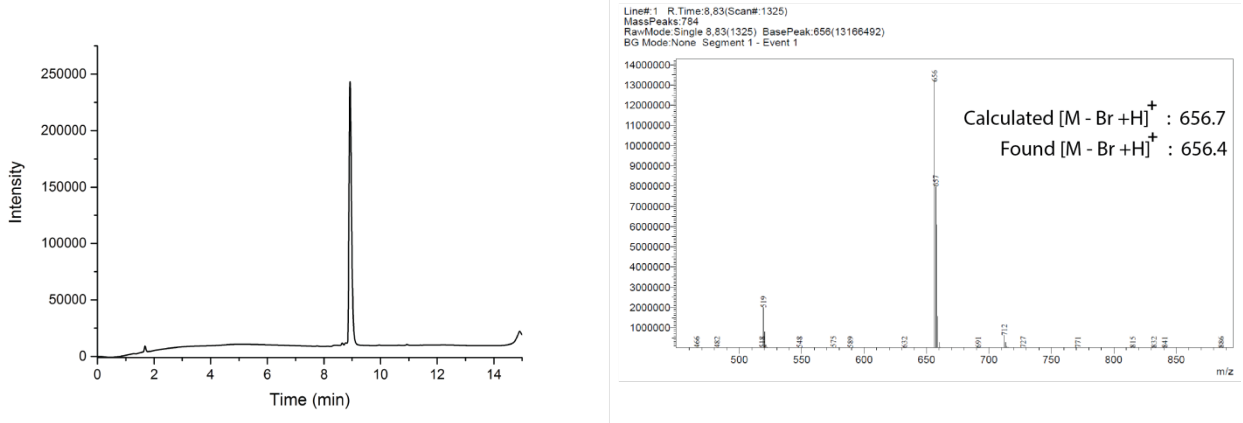

HPLC-MS analysis of TPP-Tet probe.
Conditions: solvent A: H_2O + 0.05% HCOOH; solvent B: CH_3CN + 0.05% HCOOH; gradient: 5% B → 95% B in 9 min, then 2 min 95% B and back to 5% B, Column: Luna® C18 column, 3u, 100A, 100 x 4.6 mm, 1 mL/min flow rate.

Synthesis of Taxol-Tet probe

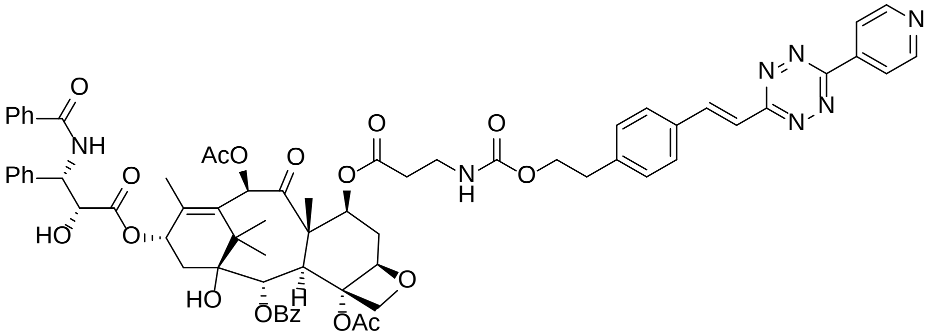

To a 0 °C solution of 7-β-alanyltaxol[9] (10 mg, 0.011 mmol) in anhydrous acetonitrile (1 mL) was added in one portion under flow of argon the active ester **3T** (5.4 mg, 0.012 mmol). The mixture was stirred at room temperature and the progress of the reaction was followed by HPLC-MS. After 18h the solvent was evaporated and the residue was purified by preparative TLC using CH_2Cl_2:MeOH (10:1) as eluent to get the product as red solid (6 mg, 43%). The purity and identity of the product was verified by HPLC-MS and HRMS.

HRMS $[M+H]^+$ m/z calcd. for $[C_{68}H_{69}O_{17}N_7Na]^+$ 1278.46421, found 1278.46488.

We performed also test click reaction with TCO **1** and verified the formation of the product by HPLC-MS. The products eluted in 9.1 and 9.9 min as mixture of two regioisomers with the same mass ($[M+H]^+$ m/z calcd. for $[C_{76}H_{84}N_5O_{18}]^+$ 1355.5, found 1355.6).

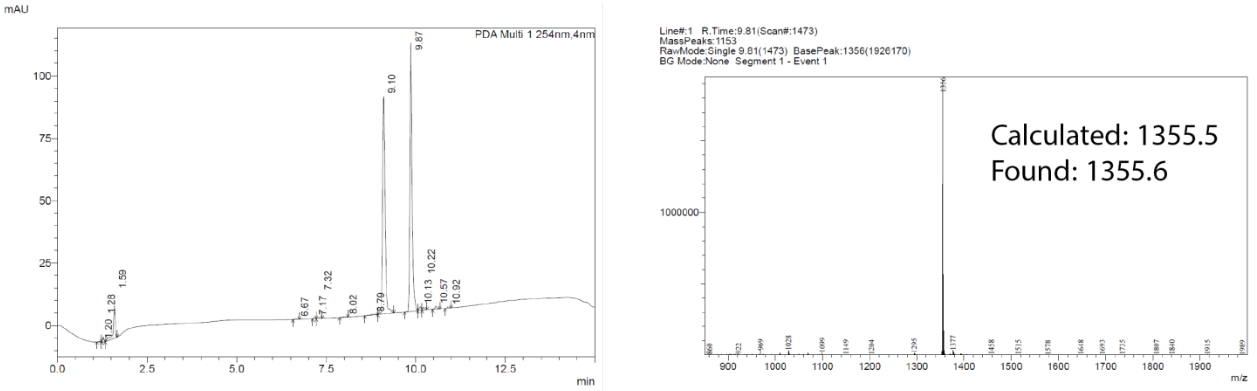

HPLC-MS analysis of Taxol-Tet probe after click reaction with **1**.

Conditions: solvent A: H_2O + 0.05% HCOOH; solvent B: CH_3CN + 0.05% HCOOH; gradient: 5% B → 95% B in 9 min, then 2 min 95% B and back to 5% B, Column: Luna® C18 column, 3u, 100A, 100 x 4.6 mm, 1 mL/min flow rate.

Synthesis of KYHWYGYTPQNVI model peptide

YHWYGYTPQNVI peptide was synthesized on TentaGel S OH resin (particle size 130 µm; Iris Biotech GmbH, Germany) using standard Fmoc chemistry on a PS3 peptide synthesizer (Protein Technologies, Inc., USA). The first amino acid was manually attached to the resin as a symmetrical anhydride (generated in situ using 10 eq amino acid and 5 eq DIC in DCM at 0 °C), further coupling steps used 3.3 eq amino acid and HBTU in DMF containing 0.2 M *N*-Methylmorpholine (NMM) for 20 min. After removal of the Fmoc protecting group (20 % piperidine (v/v) in DMF, 3x 5 min) the N-terminal lysine was added manually (6.6 eq amino acid and HBTU, 45 min) and the N-terminus was capped with acetic anhydride (7.5 % Ac_2O and 7.5 % NMM (v/v) in DMF, 1 h).

Fluorogenic click-labeling of Tetrazine-KYHWYGYTPQNVI peptide on Tentagel resin

100 mg of the resin containing the KYHWYGYTPQNVI peptide were incubated with 500 µL of TFA/TIPS/H_2O= 95/2.5/2.5 mixture for 1.5 h at room temperature to remove the side-chain protecting groups. After washing the resin with DMF (5x 5mL) and DCM (5x 5mL) part of the deprotected peptide (ca. 50mg) was reacted on the resin with Tetrazine NHS ester **3R** (5 mg) in DMF (200 µL) in the presence of DIPEA (5 µL) for 2 hours at room temperature. The progress of the reaction was evident since the beads became red as a result of the attached tetrazine moiety. A small portion of the peptide (ca. 5-10 mg) was cleaved from the resin by using 100 mM NaOH solution (50 µL, 1h at room temperature) to verify the success of the synthesis by ESI-MS (Figure S3). For the fluorogenic labeling experiment a small amount of the beads (in DMSO/ H_2O) was transferred on a glass slide and were placed under fluorescent stereomicroscope (equipped with grayscale camera: Leica DFC3000 G). A drop of the corresponding TCO isomer (50 mM solution in DMSO/H_2O = 1/1) was dropped onto the beads to initiate the reaction. The pictures were acquired at indicated time points (Figure S2). The peptides were then cleaved from the resin using 100 mM NaOH solution (1h at room temperature) and were analyzed by ESI-MS to confirm the formation of the click products. Please note that also in the case of the equatorial TCO isomer we found by HPLC-MS analysis the mass of the expected dihydropyridazine product rather than the water addition product. Presumably the additional treatment of the peptide (cleavage, neutralization etc.) promoted the final isomerization and led to the formation of the final dihydropyridazine product.

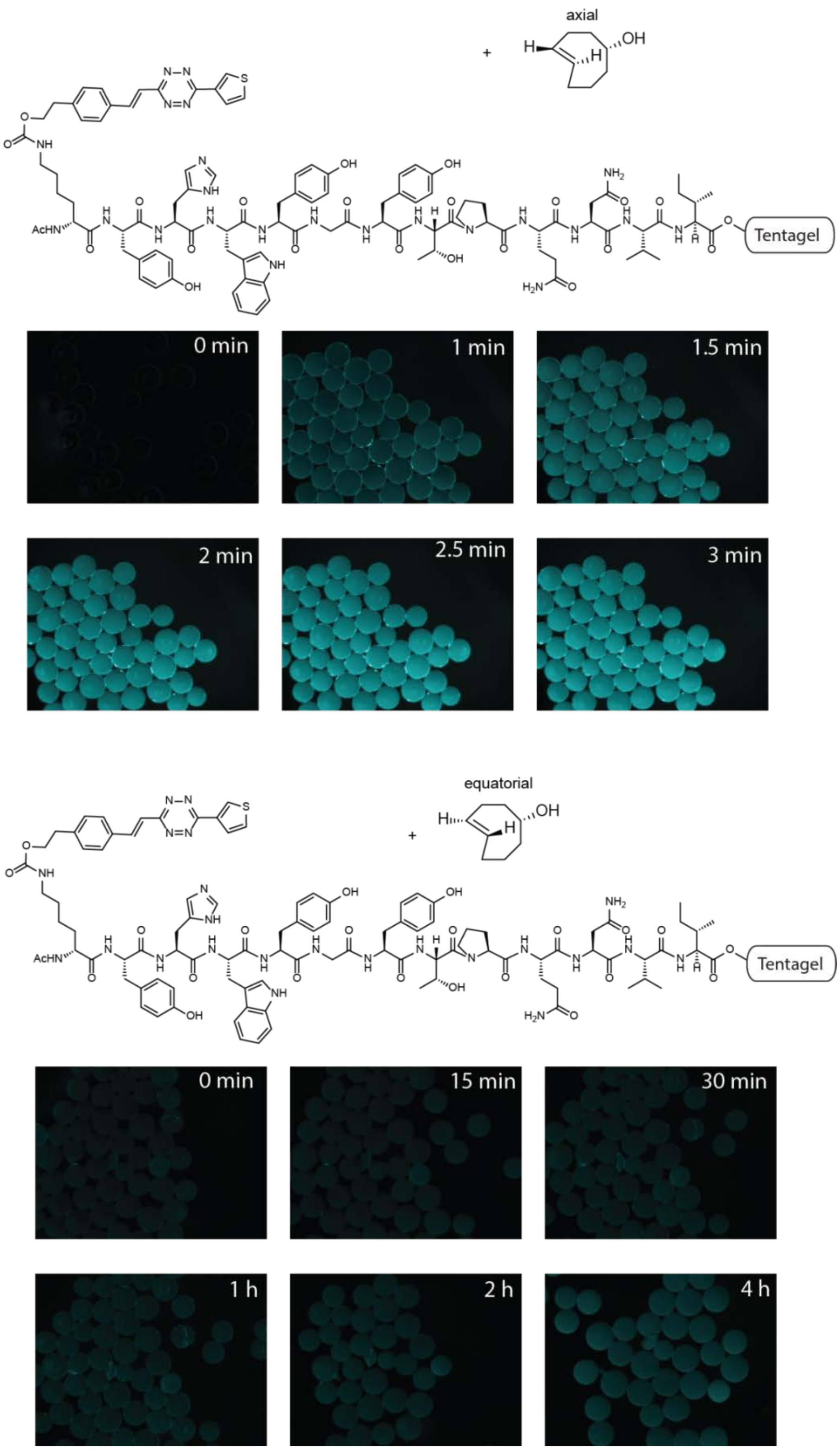

Figure S2. The resin beads containing the tetrazine modified peptide were incubated with the axial TCO **1** or equatorial TCO **2** and the pictures were captured with grayscale camera at indicated time points using fluorescent stereomicroscope and UV excitation (350 nm). The colour of the beads was adjusted using LAS AF Lite program.

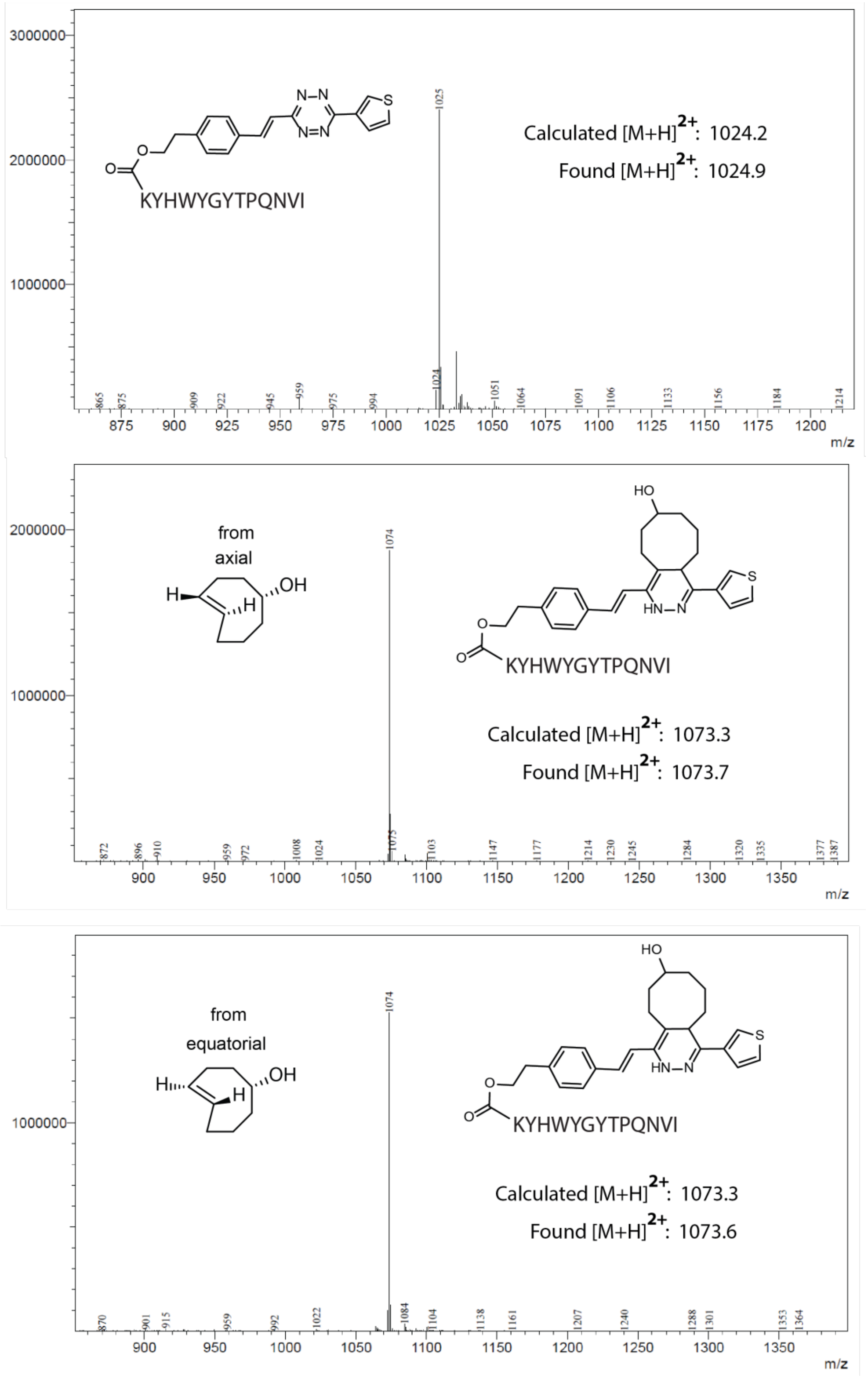

Figure S3. Mass spectra (ESI) of the peptides before and after labeling with TCOs **1** and **2**.

Fluorogenic click-labeling of TCO-KYHWYGYTPQNVI peptides on Tentagel resin

150 mg of the model peptide on Tentagel OH resin was washed with DMF (5x 5mL) and DCM (5x 5mL) and dried under vacuum. The side-chain protecting groups were removed by shaking the beads in 1 mL of TFA/TIPS/H_2O= 95/2.5/2.5 mixture for 1.5 h at room temperature. The beads containing the deprotected peptide were filtered using small SPA column (2 mL from GBiosciences), washed with DMF (5x 1mL), DCM (5x 1mL), EtOH (5x 1mL) and finally dried on SpeedVac. Small amount of the resin (ca. 5-10 mg) was weight into PP vial and the peptide was cleaved from the resin using 50 µL 100 mM NaOH (1h at room temperature). After neutralization with 1M HCl (5 µL, 1 equiv.) the solution was analyzed by HPLC-MS showing that the peptide synthesis was successful (Figure S8).

The rest of the deprotected peptide on the resin was divided into 4 PP vials and to each vial was added different TCO-*N*-hydroxysuccinimide active ester shown below (each 5 mg dissolved in 200 µL of DMF) followed by 5 µL of DIPEA. The reaction mixture was agitated at room temperature in the dark for 2 h to provide peptides modified with the corresponding TCO as verified by HPLC-MS after cleavage from the solid support by 100 mM NaOH and neutralization with 1M HCl (see below). Please note that the formed carbamate linker between TCO and the peptide is not completely stable toward the cleavage procedure (100 mM NaOH) and we often observed partial cleavage of the TCO moiety giving in HPLC measurements peak corresponding to starting unmodified peptide.

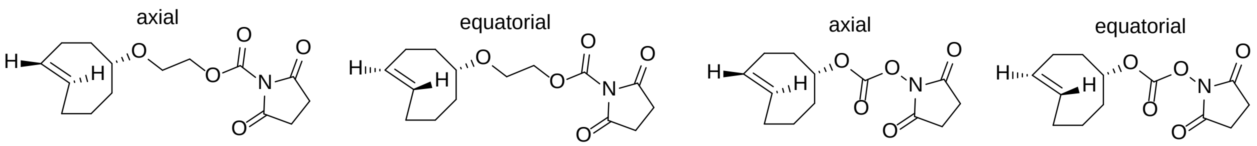

Structures of TCO-NHS esters used to modify the peptide

Next, the four peptides containing four different TCOs (two axial and two equatorial isomers) were washed with DMF (5x 1mL), DCM (5x 1mL) and finally suspended in DMSO/H_2O = 1/1. Tetrazine **3l** was added to each vial until the reddish color of the tetrazine persisted (excess of tetrazine). The beads were then washed, placed on microscope slide and inspected in time using Fluorescent stereomicroscope (Leica M205 FA). The pictures were captured by grayscale camera (Leica DFC3000 G) and later processed by LAS AF software.

Finally, small portion (ca. 5-10 mg) of the resin containing the modified peptide was treated with 100 mM NaOH (50 µL) for cleavage. After 1h the solution was neutralized with 1M HCl (5 µL, 1 equiv.). Since the peptides partially precipitated after neutralization, 10 µL acetonitrile was added to dissolve them and the peptides were analyzed by HPLC-MS (shown below) to verify the formation of the click products.

Conditions for HPLC: solvent A: H_2O + 0.05% HCOOH; solvent B: CH_3CN + 0.05% HCOOH; gradient: 5% B → 95% B in 9 min, then 2 min 95% B and back to 5% B, Column: Luna® C18 column, 3u, 100A, 100 x 4.6 mm, 1 mL/min flow rate.

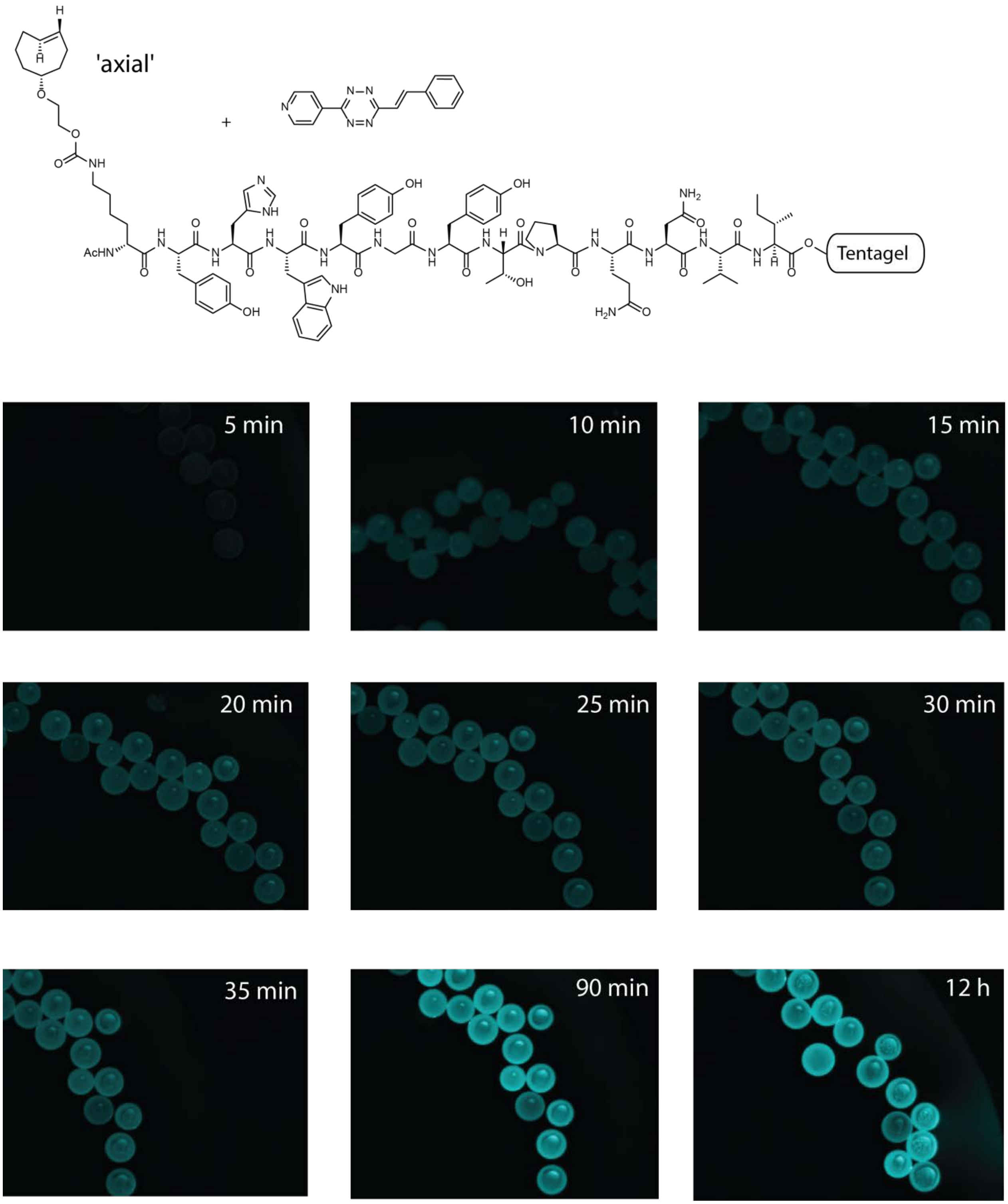

Figure S4. The resin beads containing the axial TCO modified peptide were incubated with **3l** and the pictures were captured with grayscale camera at indicated time points using fluorescent stereomicroscope and UV excitation (350 nm). The color of the beads was adjusted using LAS AF Lite program.

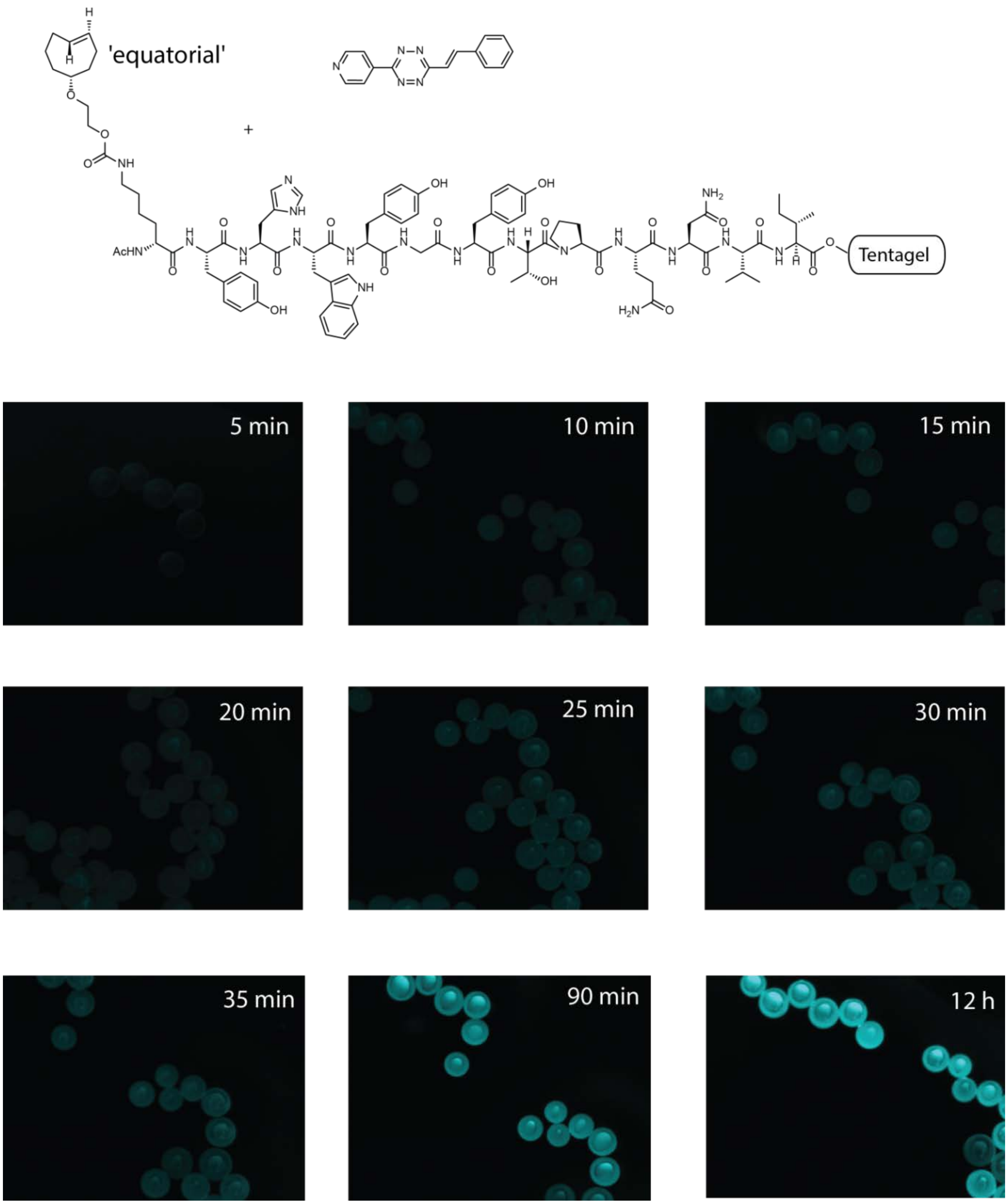

Figure S5. The resin beads containing the equatorial TCO modified peptide were incubated with **3l** and the pictures were captured with grayscale camera at indicated time points using fluorescent stereomicroscope and UV excitation (350 nm). The color of the beads was adjusted using LAS AF Lite program.

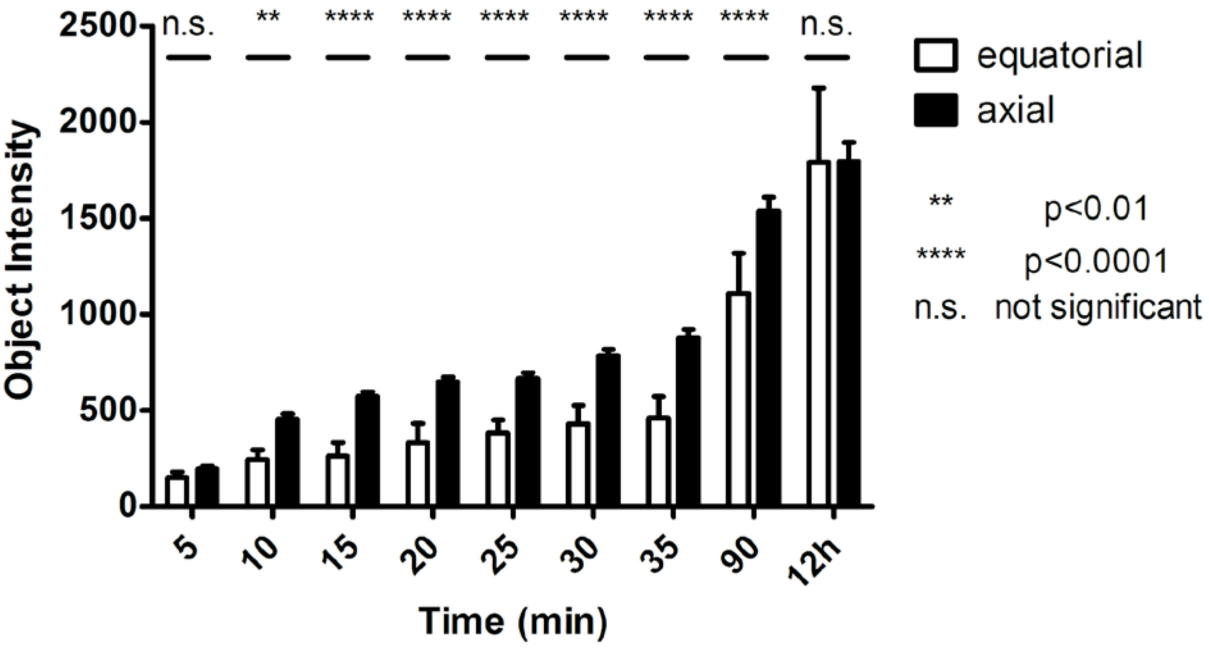

Figure S6. The fluorescence intensity (from experiments depicted in Figure S4 and S5) was quantified using the image analysis software CellProfiler.[10] Integrated intensities of single beads identified in each time point are plotted in the graph as a mean ± standard error of measurement.

The results show that the structure of the TCO influences the fluorogenic nature of the reaction. The experiment confirmed the superior properties of the axial isomer in this regard. However, the difference between the two axial and equatorial isomers was not so expressive and the fluorescence also developed much slower in this case (compare Figures S4, S5 with Figure S2).

Moreover, we found that by attaching the TCO moiety to the peptide via a carbamate linker almost completely abolishes the fluorogenic properties of the reaction (Figure S7). This supports conclusions from our computational studies where we found that the free OH group is involved in the tautomerization of the dihydropyridazine product proceeds via 1, 3-hydrogen shift rather than hydration-dehydration steps. It seems that further derivatization of the free OH group leads to alteration of the mechanism. This may also partially explain why this phenomenon remained undetected since the active esters of the *trans*-cycloocten-ols are the most commonly employed TCO derivatives for bioconjugations and in this form are also commercially available.

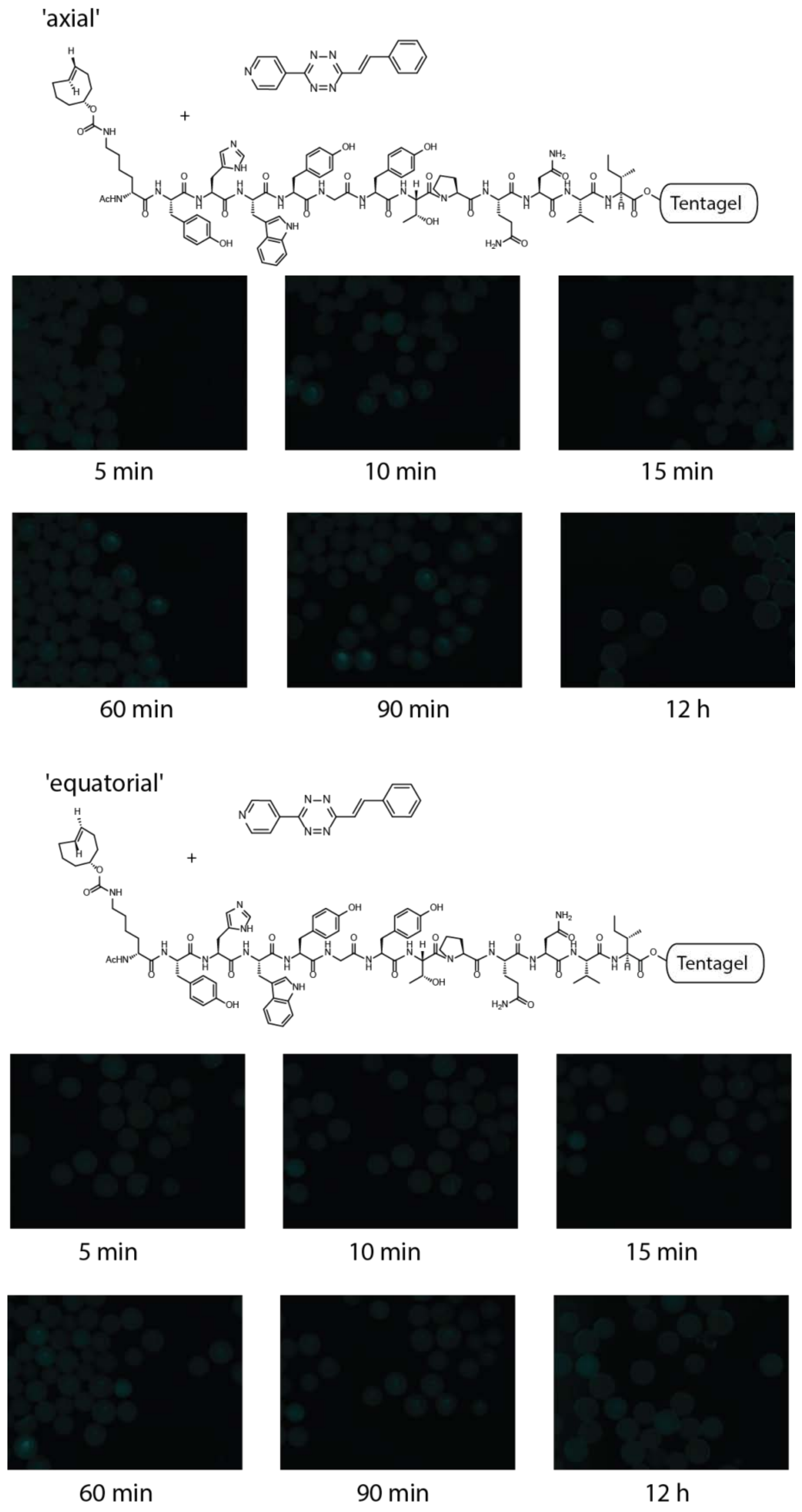

Figure S7. The resin beads containing the equatorial or axial TCO modified peptide were incubated with tetrazine **3l** and the pictures were captured with grayscale camera at indicated time points using fluorescent stereomicroscope with UV excitation (350 nm). The color of the beads was adjusted using LAS AF Lite program.

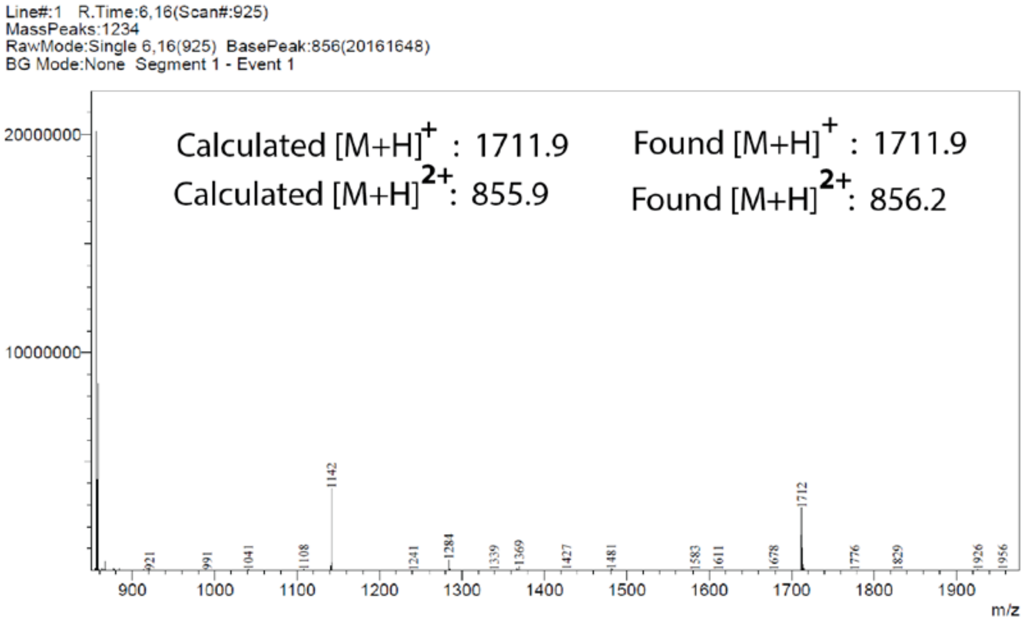

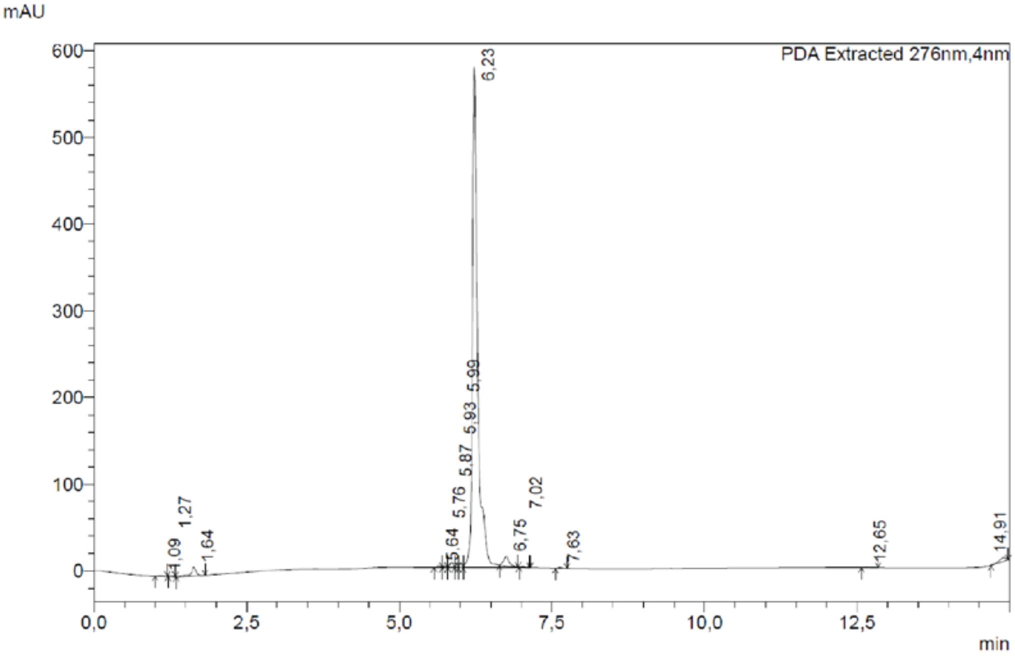

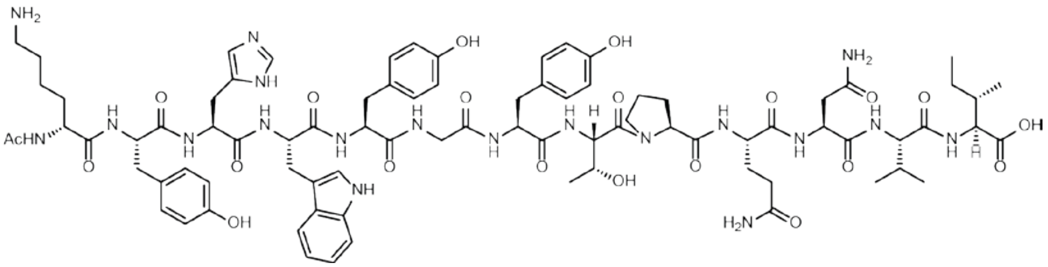

Figure S8. Mass spectra (ESI) and HPLC chromatogram of the starting peptide KYHWYGYTPQNVI

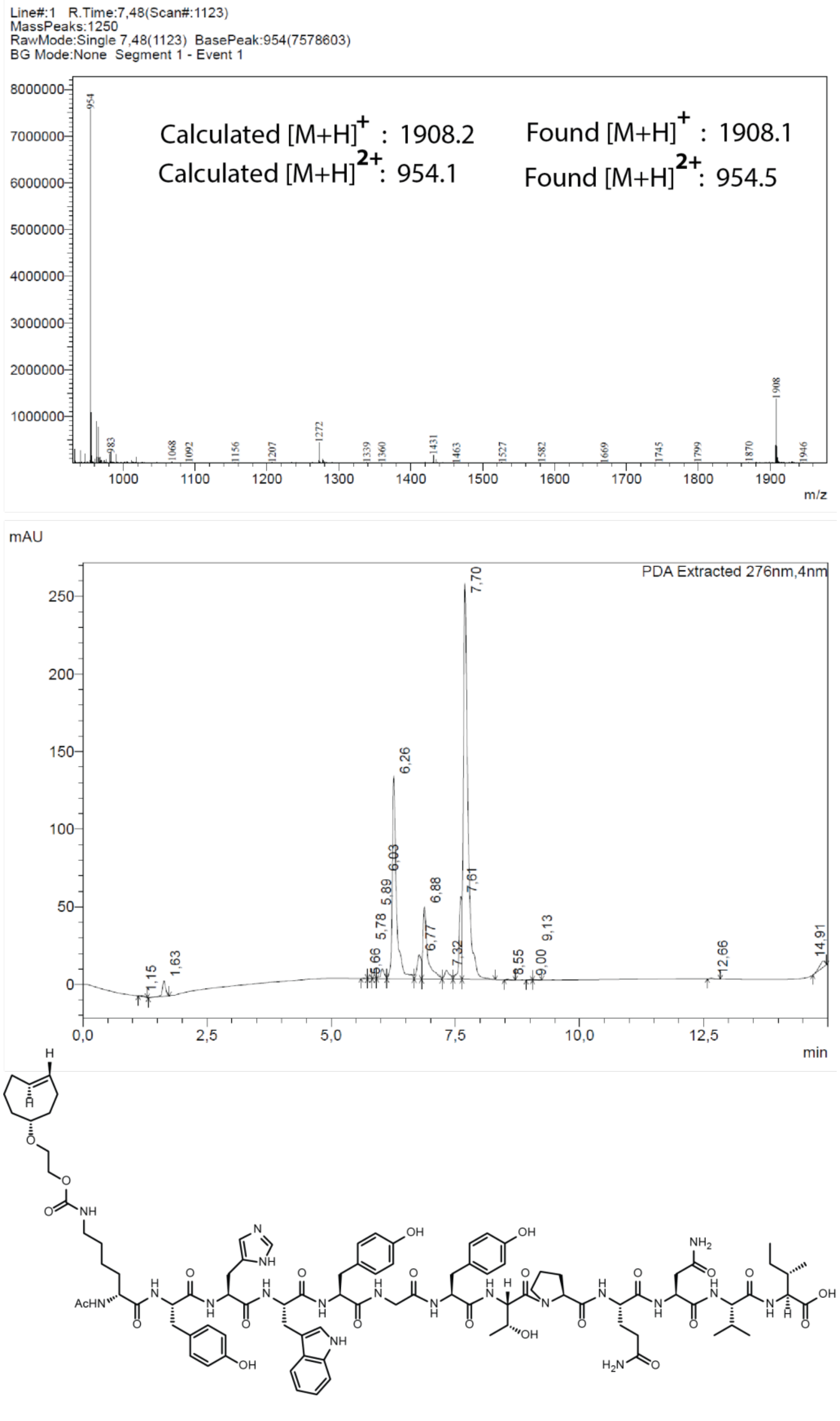

Figure S9. Mass spectra (ESI) and HPLC chromatogram of the axial TCO modified peptide. The smaller peak at 6.3 min corresponds to the starting peptide (result of the carbamate linker cleavage)

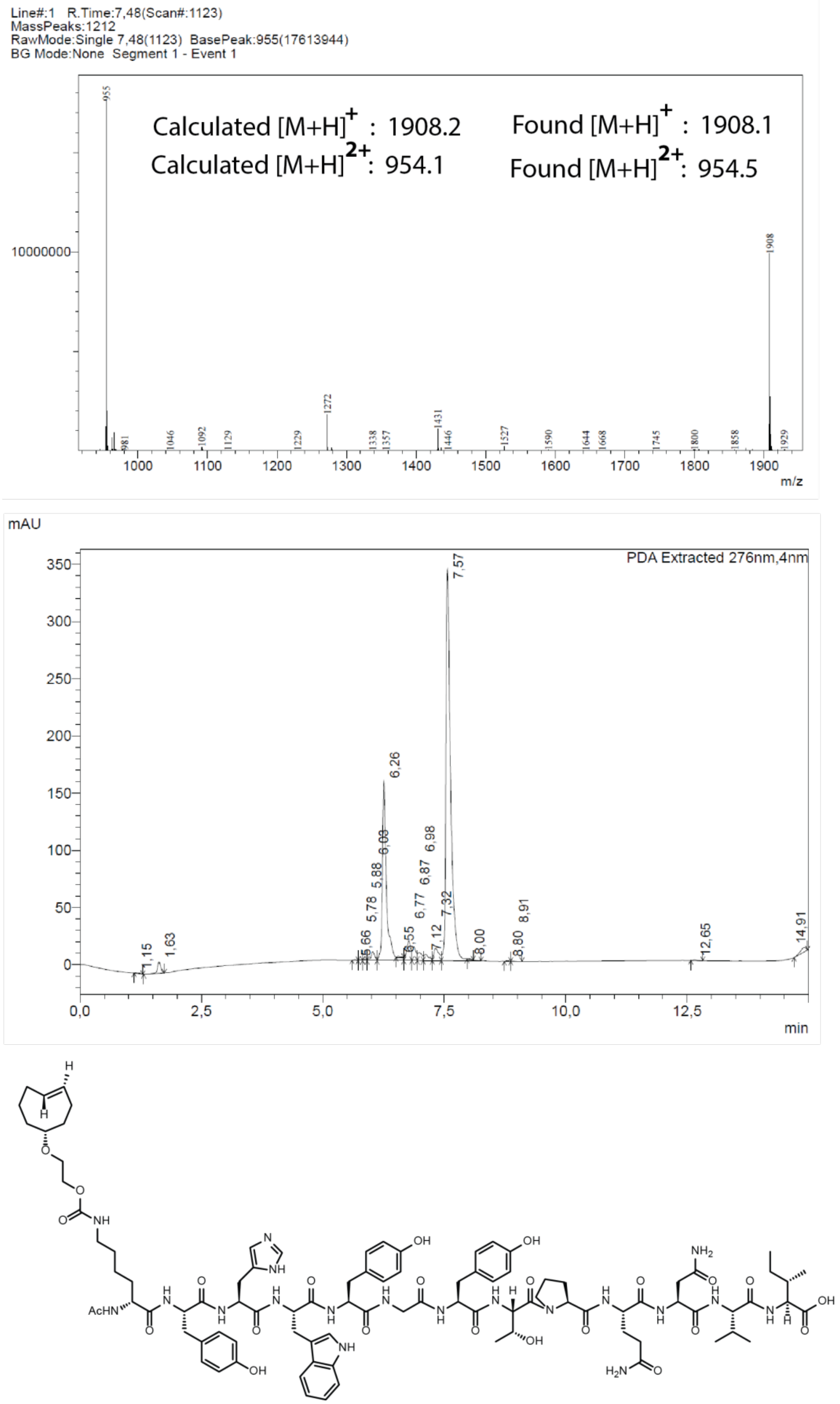

Figure S10. Mass spectra (ESI) and HPLC chromatogram of the equatorial TCO modified peptide. The smaller peak at 6.3 min corresponds to the starting peptide (result of the carbamate linker cleavage)

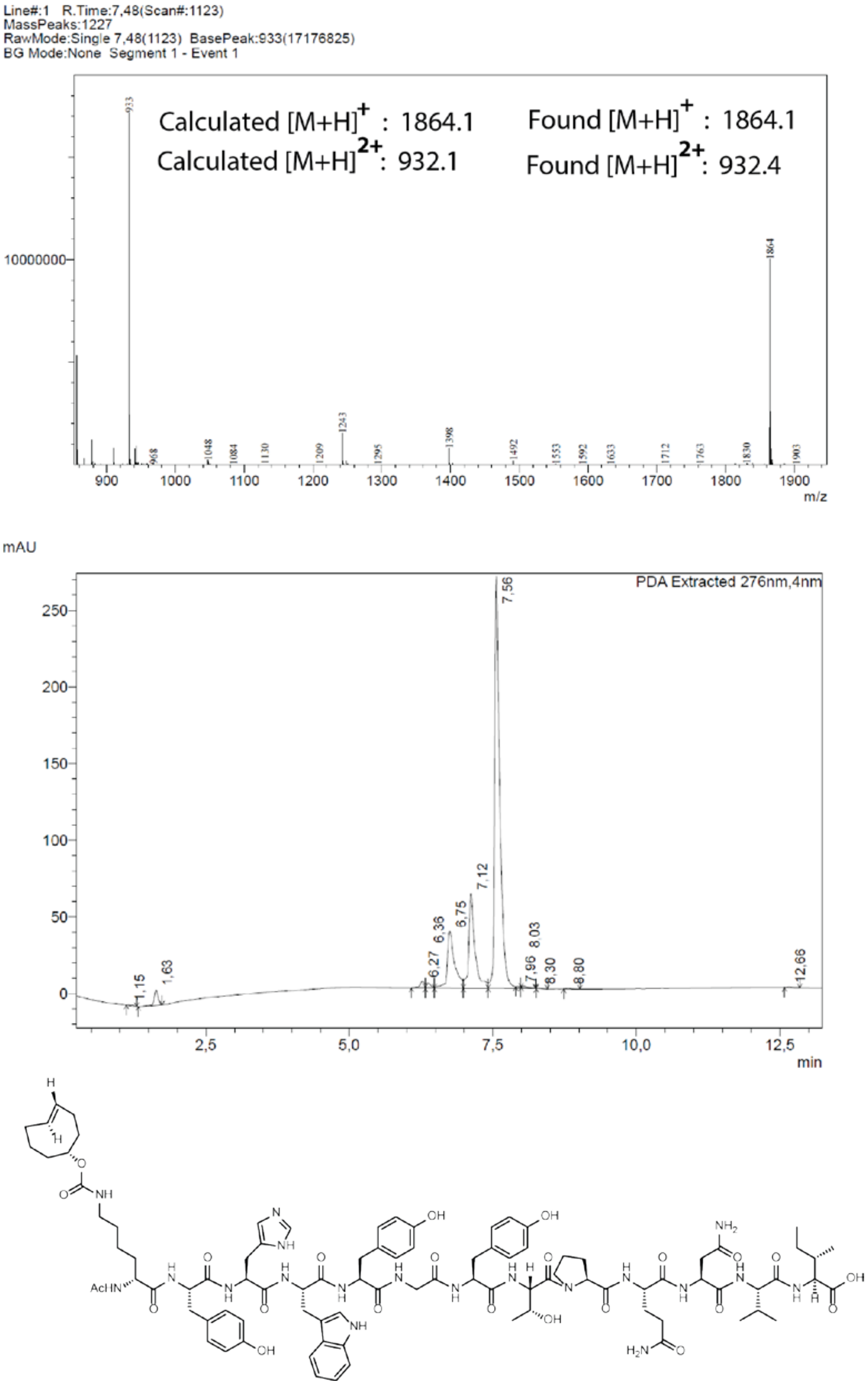

Figure S11. Mass spectra (ESI) and HPLC chromatogram of the axial TCO modified peptide

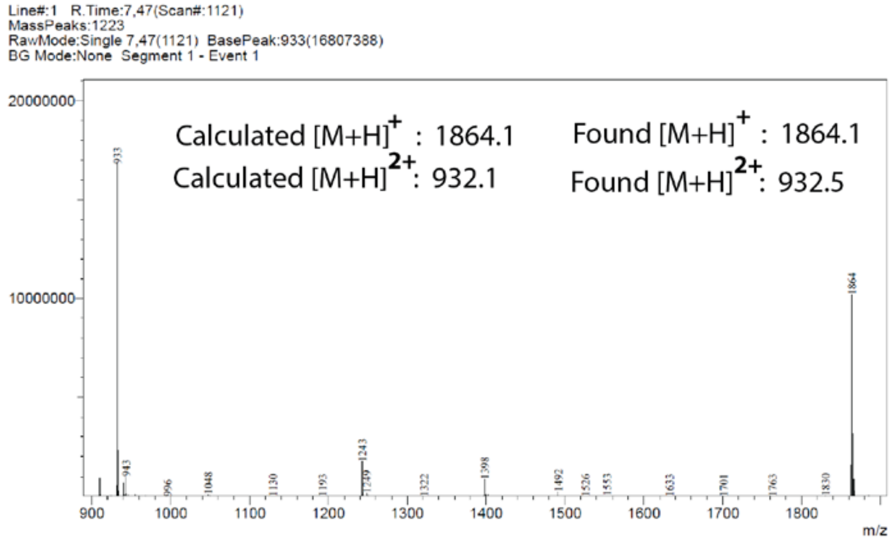

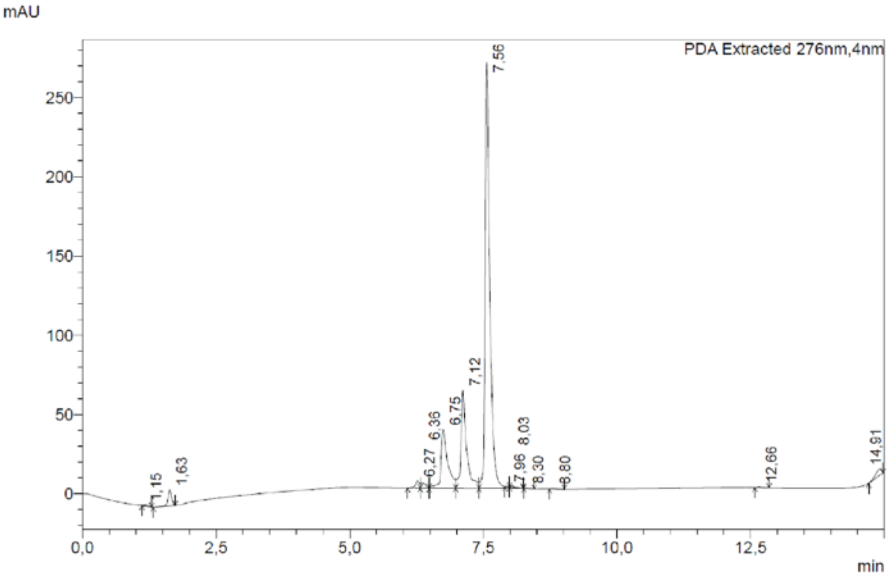

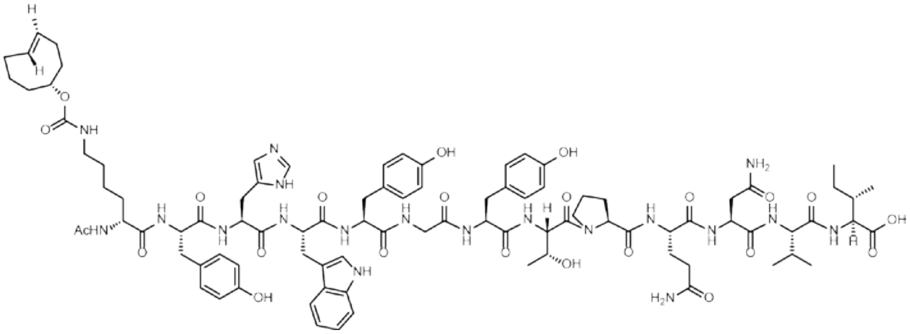

Figure S12. Mass spectra (ESI) and HPLC chromatogram of the equatorial TCO modified peptide

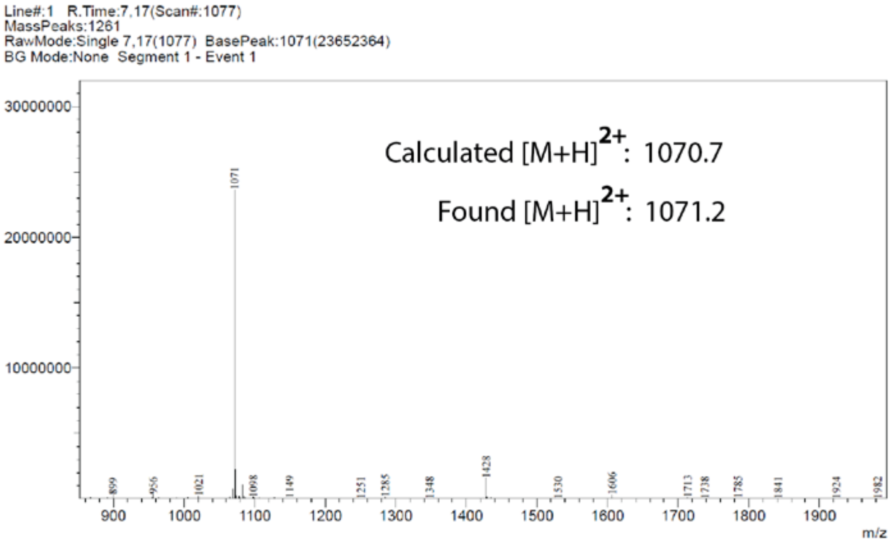

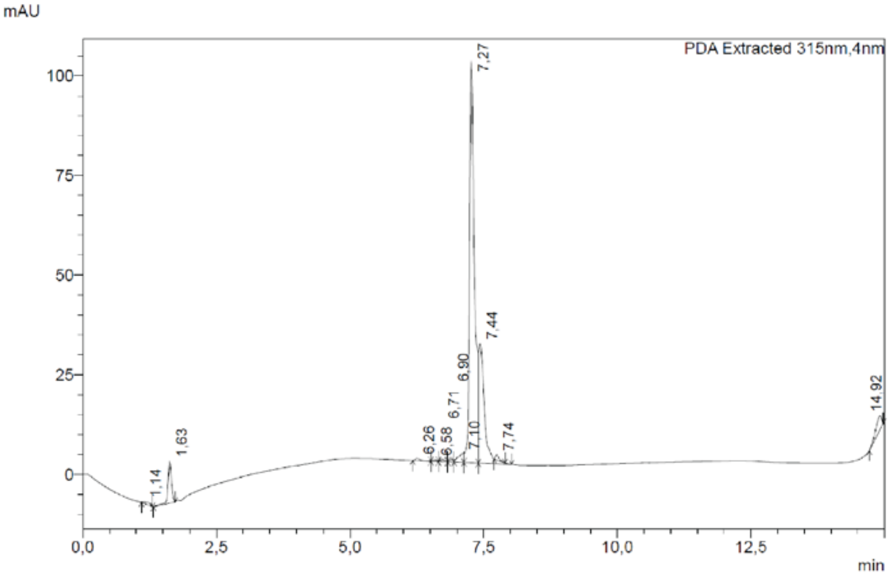

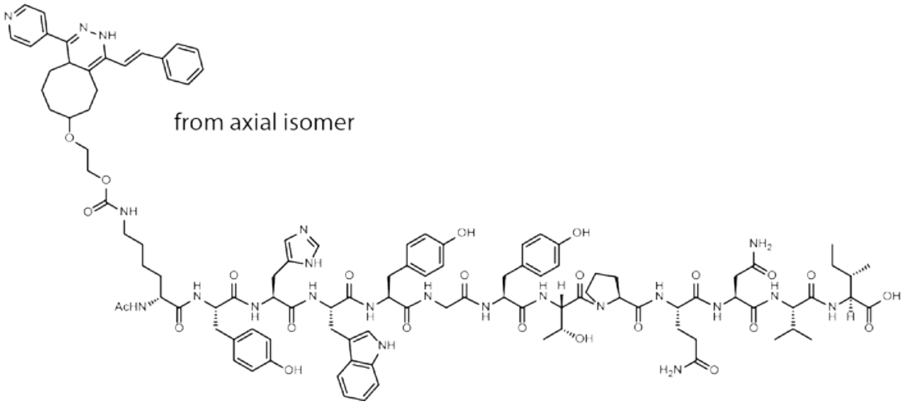

Figure S13. Mass spectra (ESI) and HPLC chromatogram of the axial TCO modified peptide after labeling with tetrazine **3l**

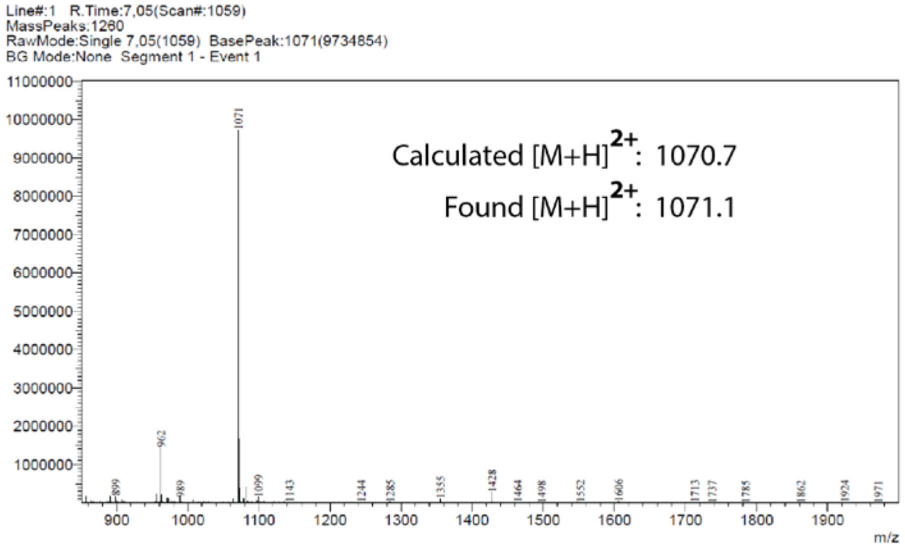

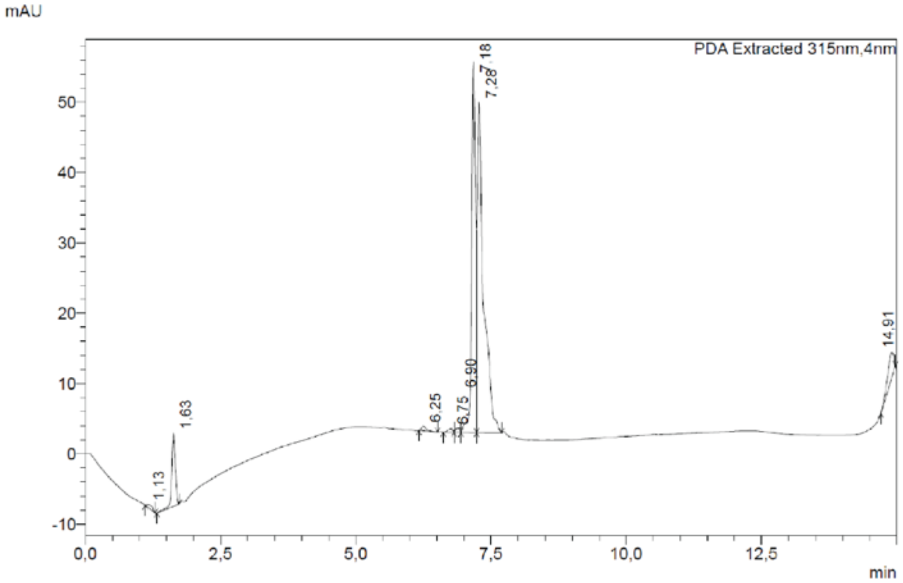

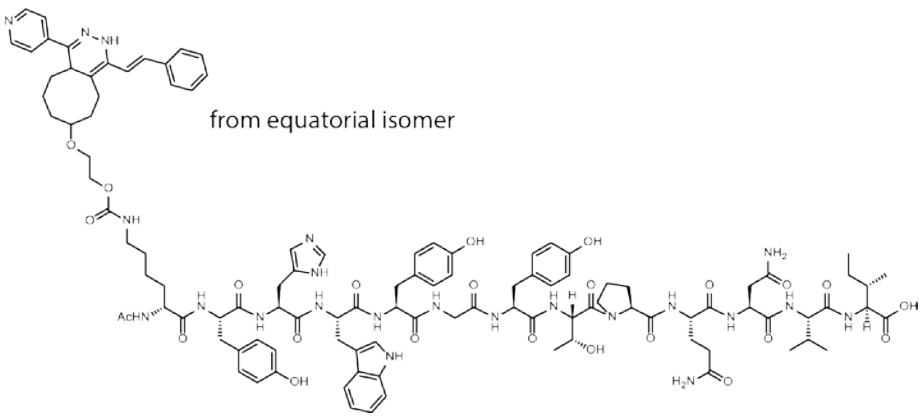

Figure S14. Mass spectra (ESI) and HPLC chromatogram of the equatorial TCO modified peptide after labeling with tetrazine **3l**

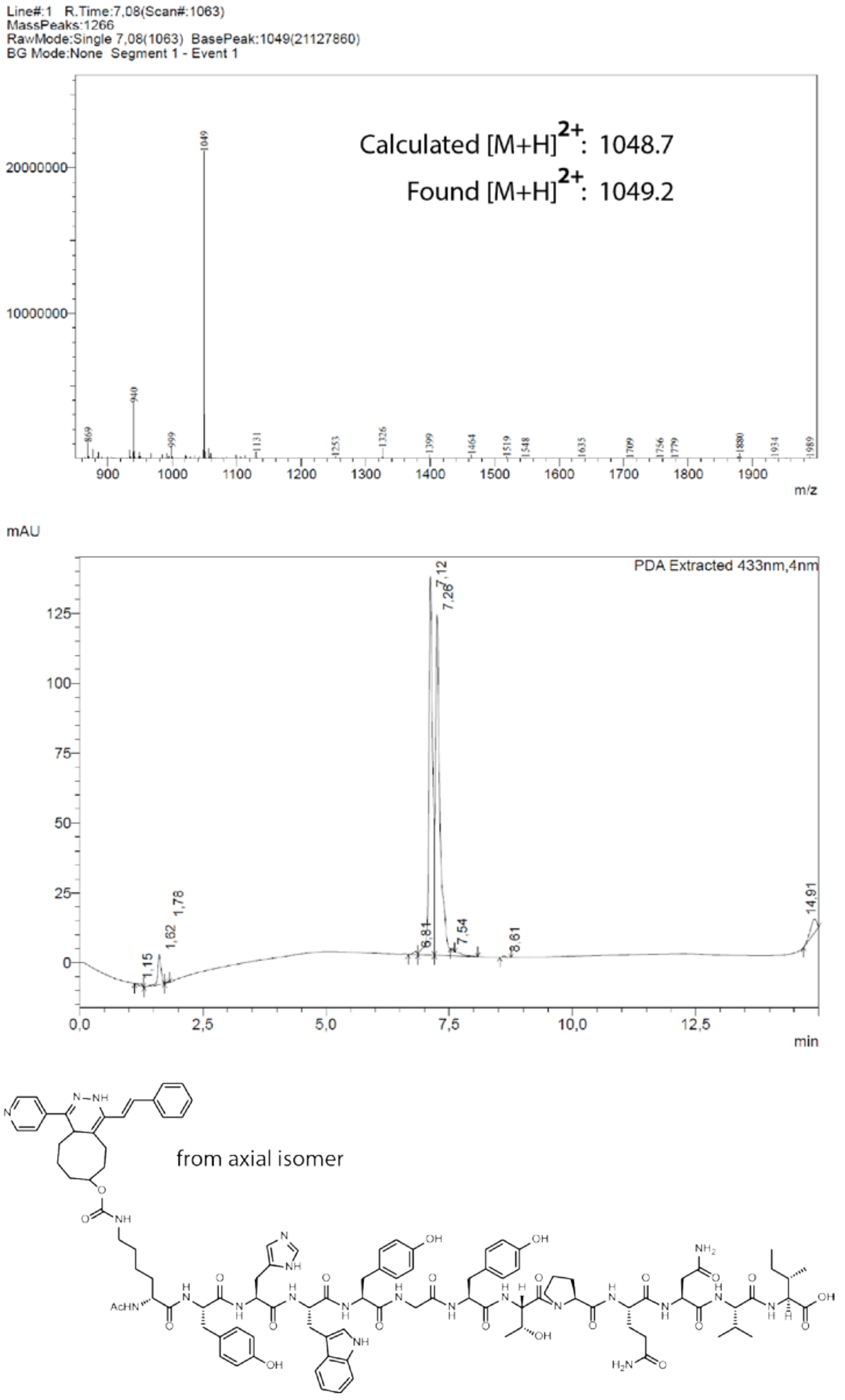

Figure S15. Mass spectra (ESI) and HPLC chromatogram of the axial TCO modified peptide after labeling with tetrazine **3l**

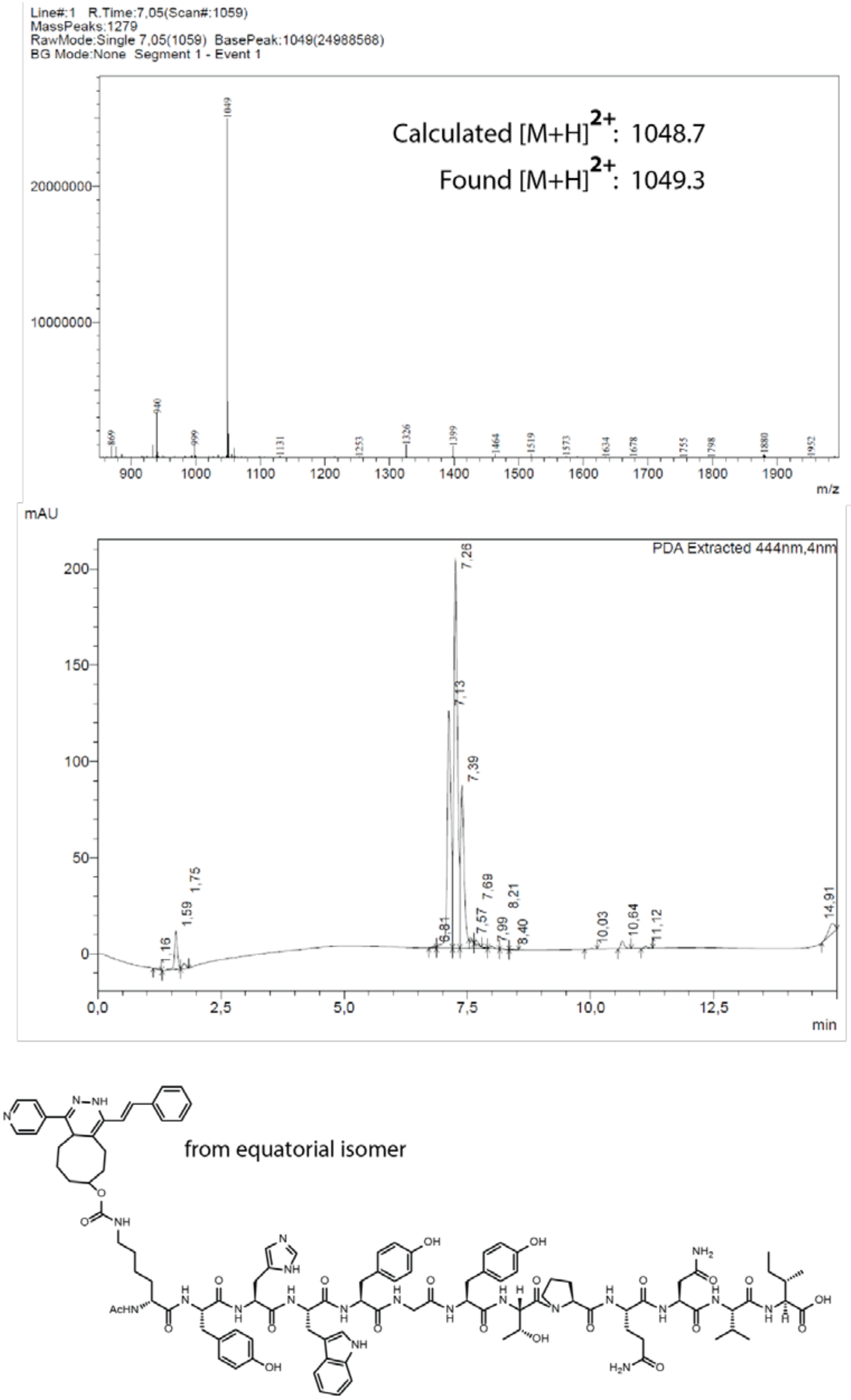

Figure S16. Mass spectra (ESI) and HPLC chromatogram of the equatorial TCO modified peptide after labeling with tetrazine **3l**

Click reactions of tetrazines 3C-3P with the axial TCO isomer

The tetrazines were dissolved in CH_3CN and H_2O was added to get 1.25 mM solution of tetrazines in CH_3CN containing 5% H_2O. These solutions were mixed in PP vials with 2 equivalents of the axial TCO isomer **1** (2.5 mM, in 5% H_2O in CH_3CN) giving 0,625 mM final tetrazine (or click product) concentrations. The reaction mixtures were incubated at room temperature for 1h. These stock solutions were then used directly for HPLC-MS measurements (HPLC and MS chromatograms are shown below), diluted to 25 µM for absorbance measurements and further to 1.25 µM for fluorescence measurements.

Conditions for HPLC: solvent A: H_2O + 0.05% HCOOH; solvent B: CH_3CN + 0.05% HCOOH; gradient: 5% B → 95% B in 9 min, then 2 min 95% B and back to 5% B, Column: Luna® C18 column, 3u, 100A, 100 x 4.6 mm, 1 mL/min flow rate.

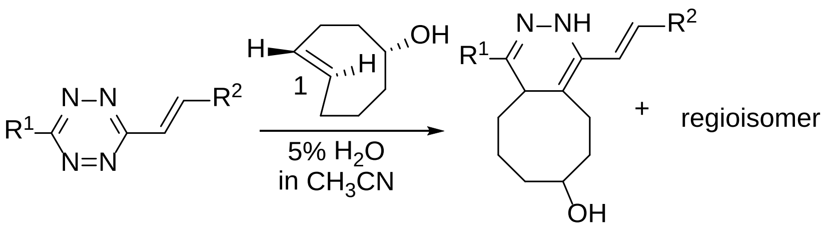

General scheme of the click reaction (for R^1 and R^2 see Table S1 and S2)

Determination of fluorescence quantum yields

Quantum yields of click products were measured at 25°C in CH_3CN containing 5% H_2O (1.25 µM final concentration) in 1 cm quartz cuvette. The experiment was performed on FluoroMax 4 spectrofluorometer (Jobin Yvon, Horiba) equipped with a 450 W xenon lamp using Quinine Sulfate (solution in 0.5M H_2SO_4) as reference (ϕ_{QS} = 0.55). The settings were as follows: Excitation wavelength 350 nm, slit 3.0 nm; Emission 400 – 680 nm, increment 1.0 nm, slit 3.0 nm and data algebra formula S1c/R1c. The fluorescence quantum yields were calculated using the following equation:

$$\phi_{sample} = \phi_{ref} \frac{F_{sample}}{F_{ref}} \times \frac{(1\text{-}10^{-abs})_{ref}}{(1\text{-}10^{-abs})_{sample}} \times \frac{n^2_{sample}}{n^2_{ref}}$$

Where:

$\boldsymbol{\phi_{ref}}$ is 0,55 (Quantum yield of Quinine sulfate in 0.5 M H_2SO_4)[11]

F are the integrated intensities (areas) of standard and the sample fluorescence spectra

abs is the absorbance of standard and sample at the excitation wavelength (350 nm)

n are the refractive indices for standard (1.333) and the sample solution (1.3404)

Table of photophysical properties of the click products using TCO 1

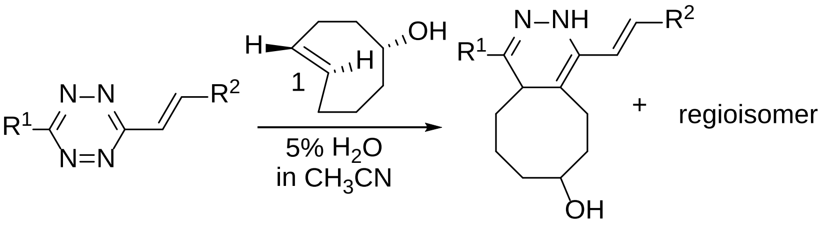

Table S1. Photophysical properties of the click products

Starting tetrazine		Click Product	λ_{Abs}/ λ_{Em} (nm)	Stokes Shift (nm)	ϕ_{fl}	Fl. intensity increase	ε_{max} x10^3 ($M^{-1}cm^{-1}$)
	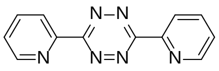	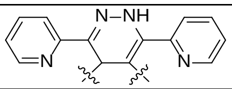	312/480	168	0,12	11-fold	18.9
**3U**	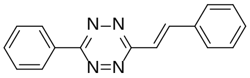	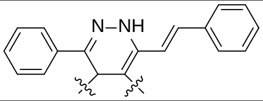	336/505	169	0,14	73-fold	17.7
**3C**	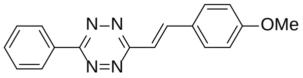	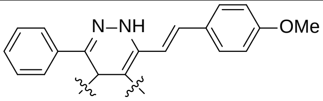	330/488	158	0,18	62-fold	13.5
**3F**	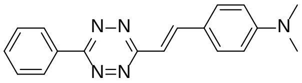	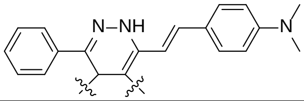	357/478	121	0,14	**91-fold**	**36.4**
**3D**	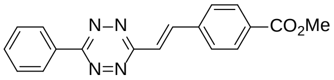	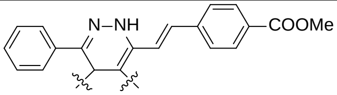	341/567	226	0,04	20-fold	25
**3E**	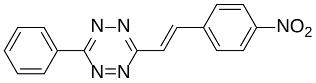	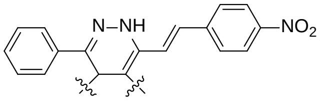	352/ -	-	-	-	24.9
**3G**	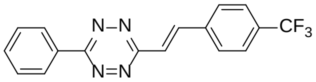	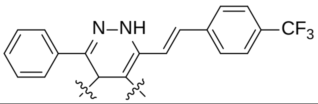	336/539	203	0,08	47-fold	26.4
**3H**	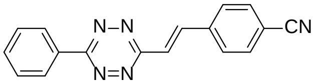	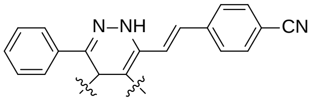	340/570	230	0,03	16-fold	23

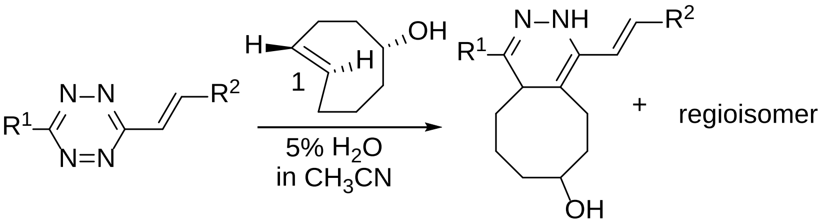

Table S2. Photophysical properties of the click products.

Starting tetrazine		Click Product	λ$_{Abs}$/ λ$_{Em}$ (nm)	Stokes Shift (nm)	ϕ_{fl}	Fl. intensity increase	ε_{max} x10^3 (M^{-1}cm^{-1})
**3I**	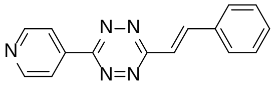	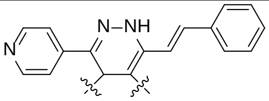	336/494	128	**0,20**	61-fold	12.9
**3J**	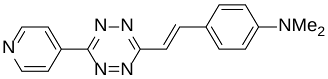	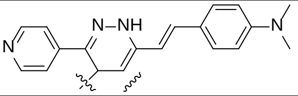	356/545	189	0,02	11-fold	20.8
**3K**	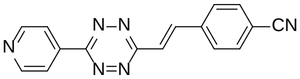	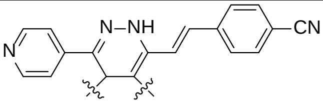	361/554	193	0,09	30-fold	12.8
**3L**	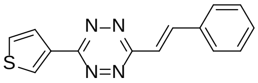	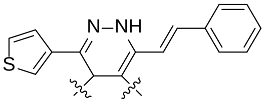	336/505	169	0,17	41-fold	11.1
**3M**	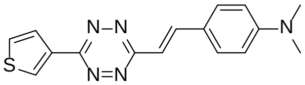	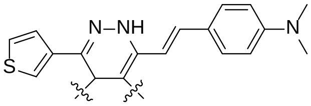	352/476	124	0,16	63-fold	20.3
**3N**	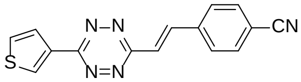	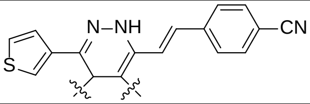	347/570	223	0,04	11-fold	22.6
**3O**	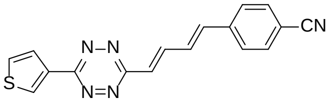	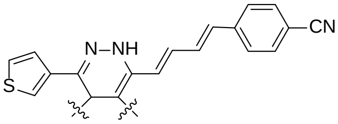	362/605	**243**	0,01	9-fold	29.6
**3P**	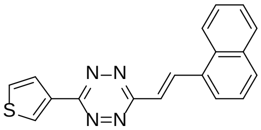	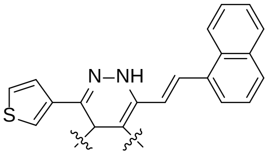	344/539	195	0,09	42-fold	18.5

Copies of absorption and emission spectra of the click products using TCO 1

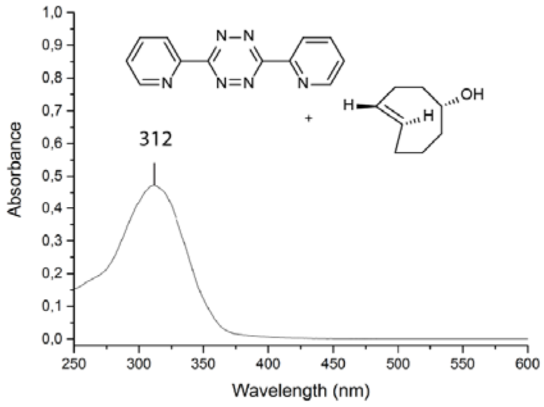

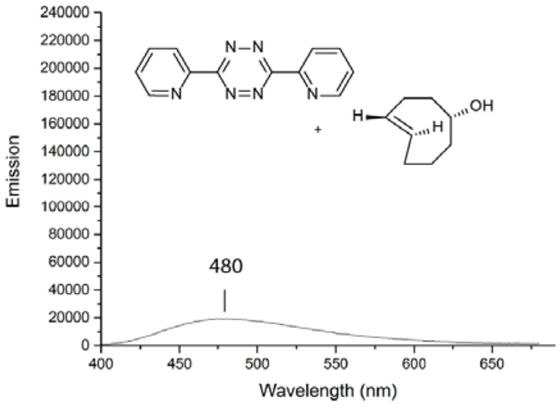

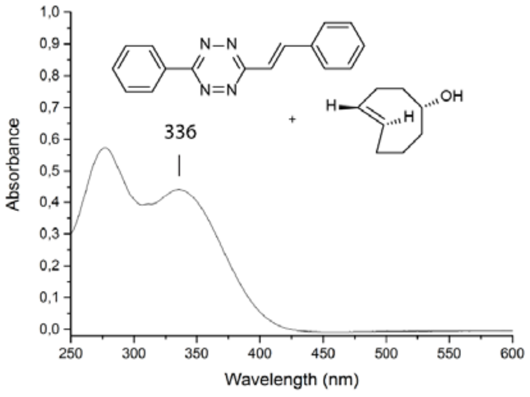

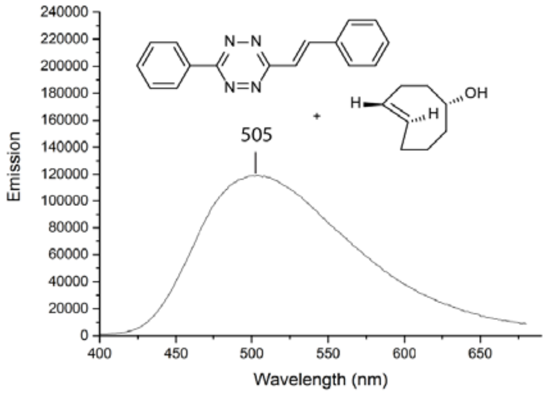

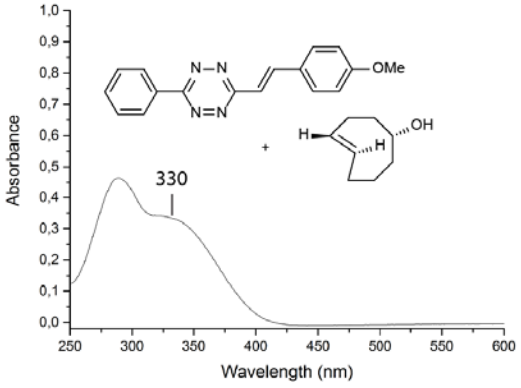

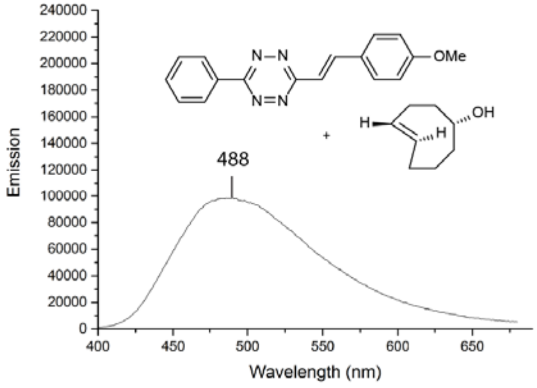

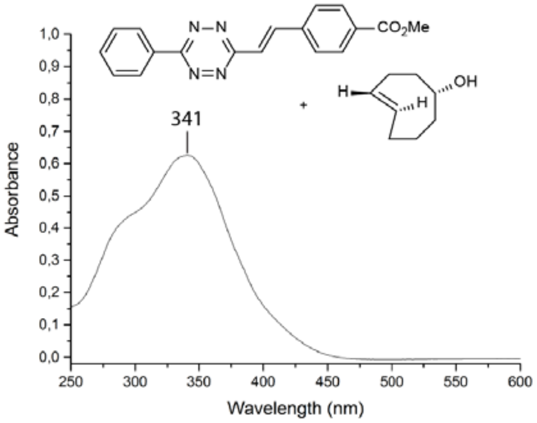

CO2Me
N–N
N=N
H
H
OH
+
341
Absorbance
Wavelength (nm)

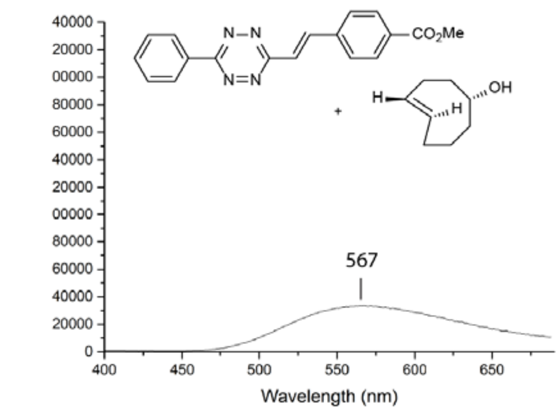

CO2Me
N–N
N=N
H
H
OH
+
567
Wavelength (nm)

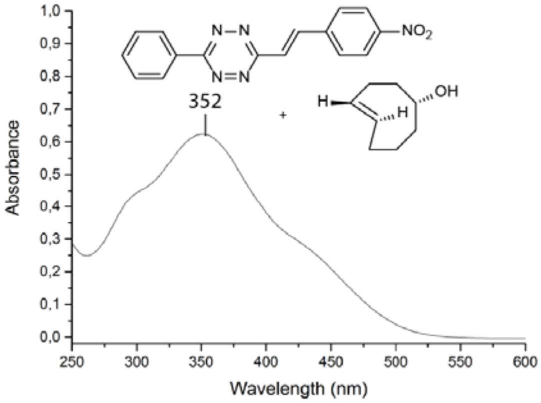

NO2
N–N
N=N
H
H
OH
+
352
Absorbance
Wavelength (nm)

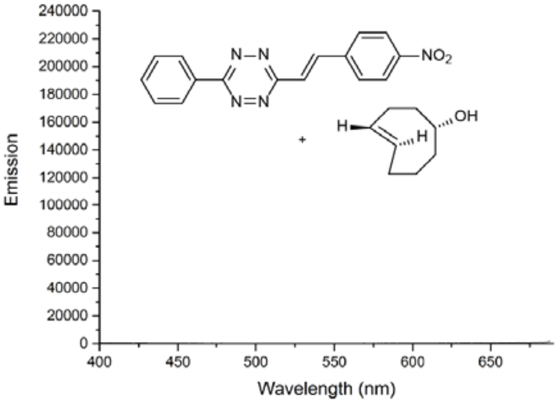

NO2
N–N
N=N
H
H
OH
+
Emission
Wavelength (nm)

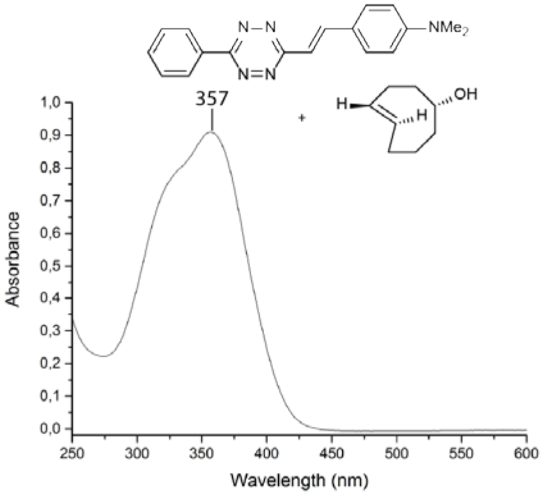

NMe2
N–N
N=N
H
H
OH
+
357
Absorbance
Wavelength (nm)

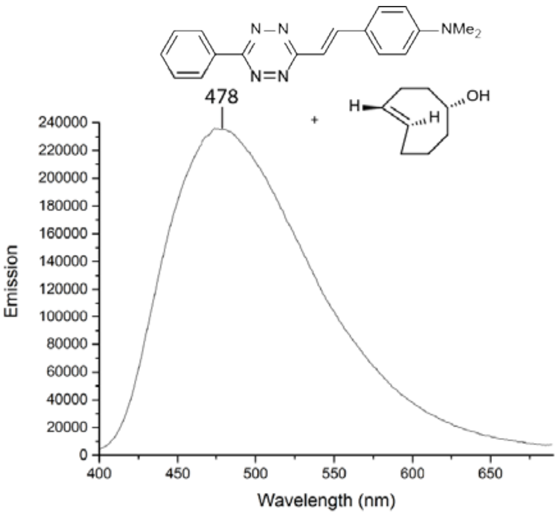

NMe2
N–N
N=N
H
H
OH
+
478
Emission
Wavelength (nm)

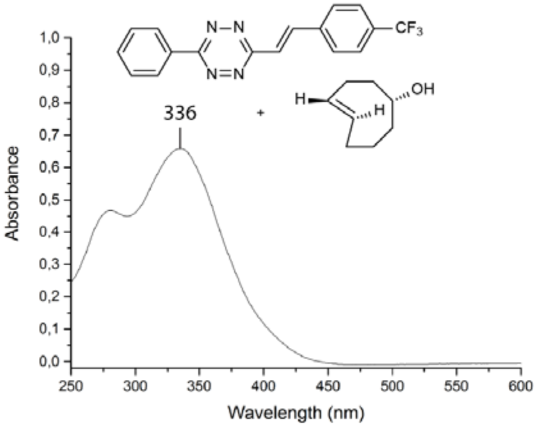
CF3
OH
H
336
Absorbance
Wavelength (nm)

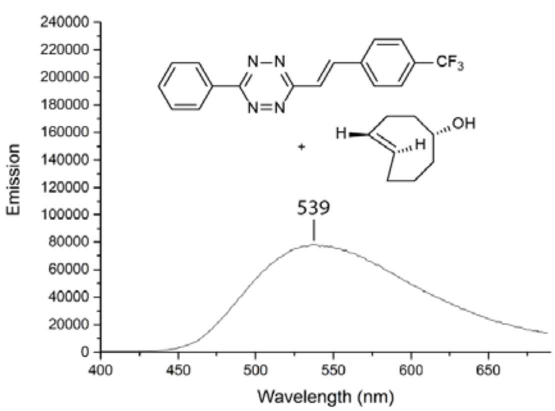
CF3
OH
H
539
Emission
Wavelength (nm)

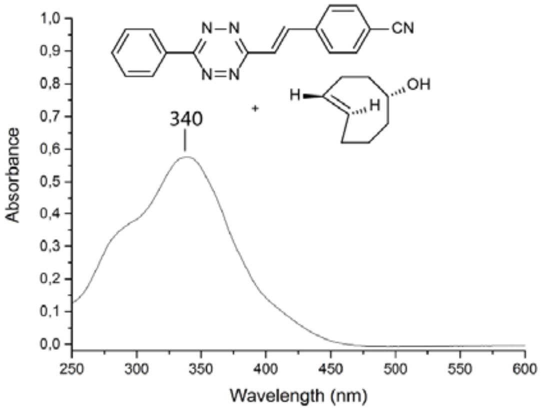
CN
OH
H
340
Absorbance
Wavelength (nm)

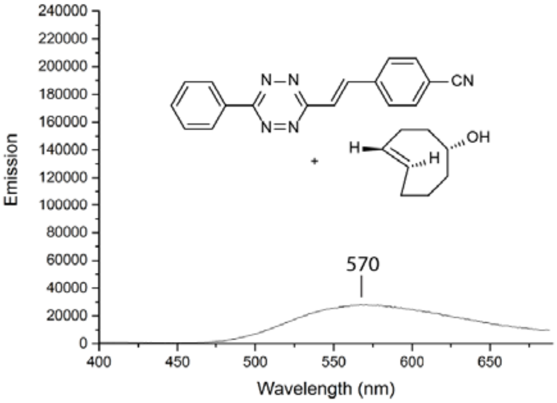
CN
OH
H
570
Emission
Wavelength (nm)

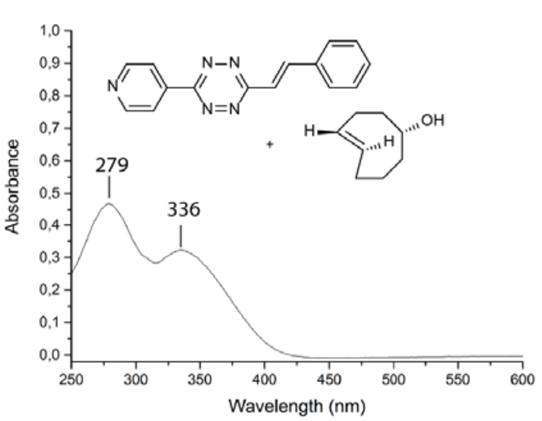
OH
H
279
336
Absorbance
Wavelength (nm)

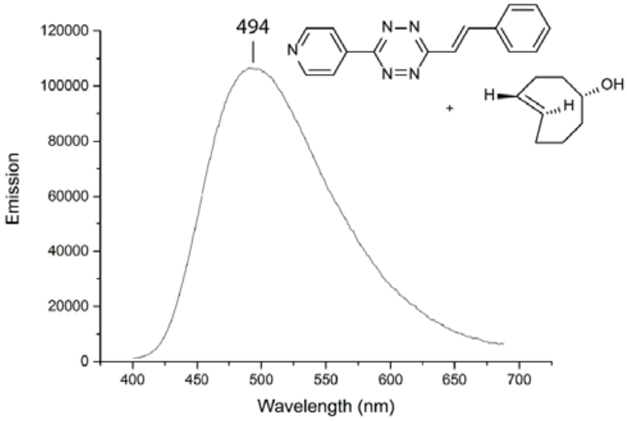
494
OH
H
Emission
Wavelength (nm)

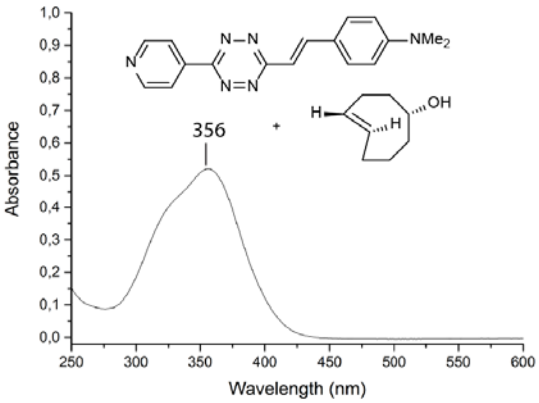
Absorbance
Wavelength (nm)
NMe2
N
OH
H
356

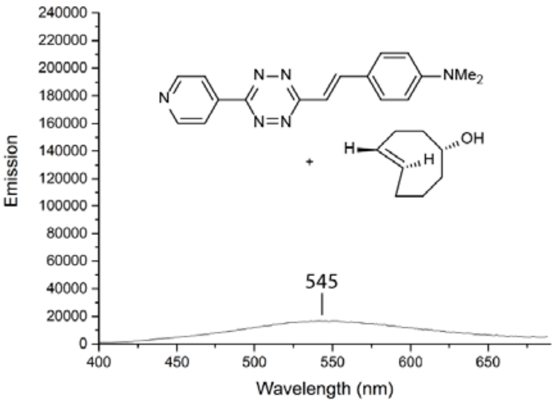
Emission
Wavelength (nm)
NMe2
N
OH
H
545

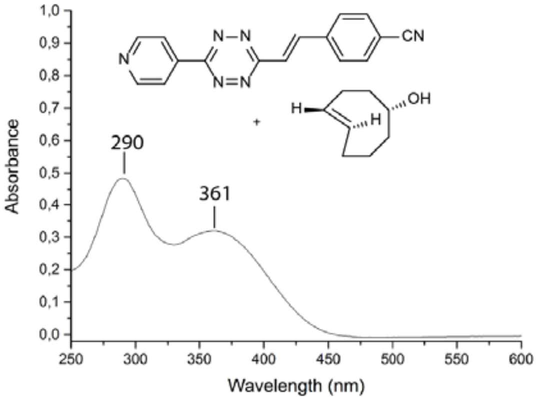
Absorbance
Wavelength (nm)
CN
N
OH
H
290
361

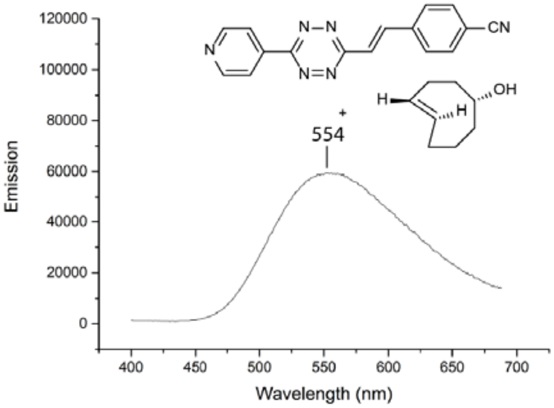
Emission
Wavelength (nm)
CN
N
OH
H
554

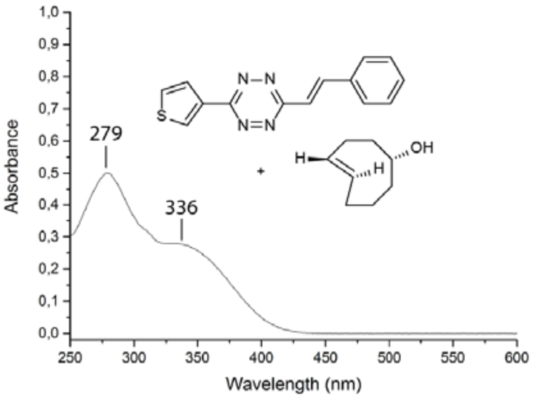
Absorbance
Wavelength (nm)
S
OH
H
279
336

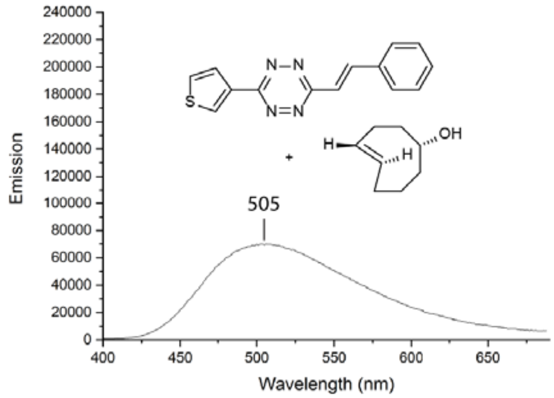
Emission
Wavelength (nm)
S
OH
H
505

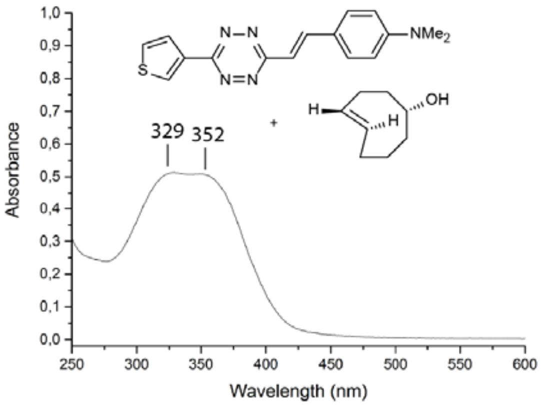

NMe2
OH
329
352
Absorbance
Wavelength (nm)

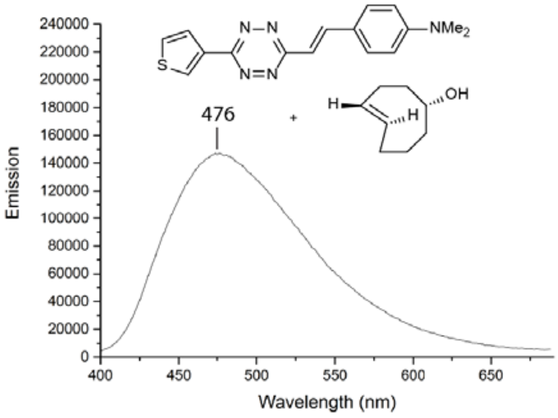

NMe2
OH
476
Emission
Wavelength (nm)

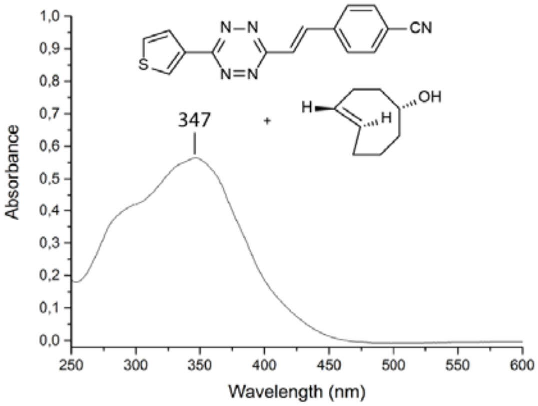

CN
OH
347
Absorbance
Wavelength (nm)

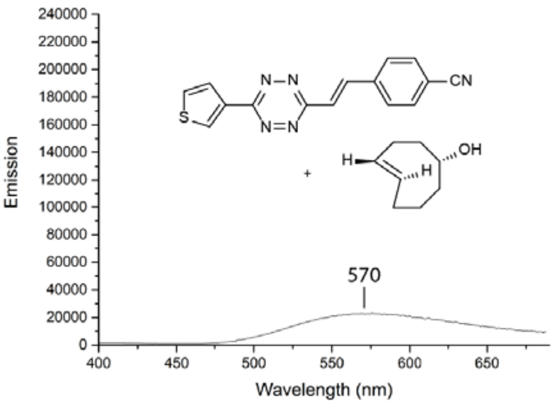

CN
OH
570
Emission
Wavelength (nm)

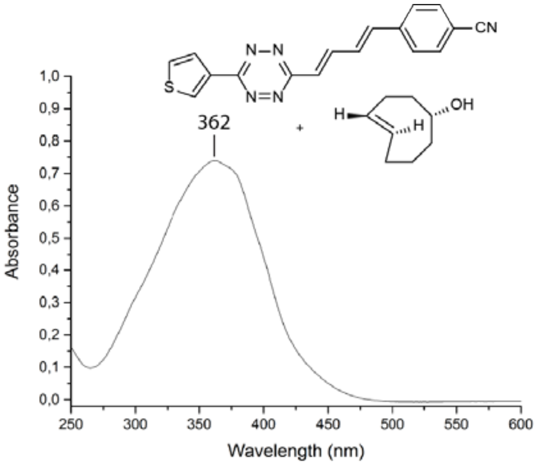

CN
OH
362
Absorbance
Wavelength (nm)

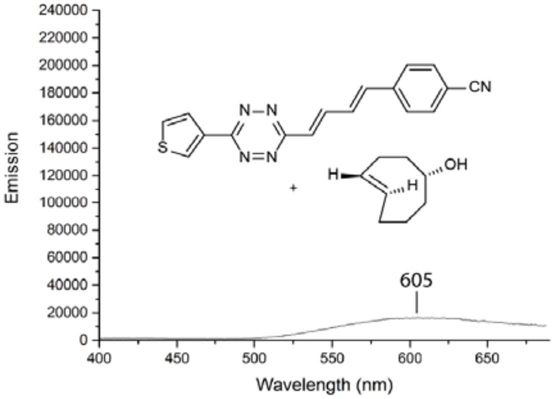

CN
OH
605
Emission
Wavelength (nm)

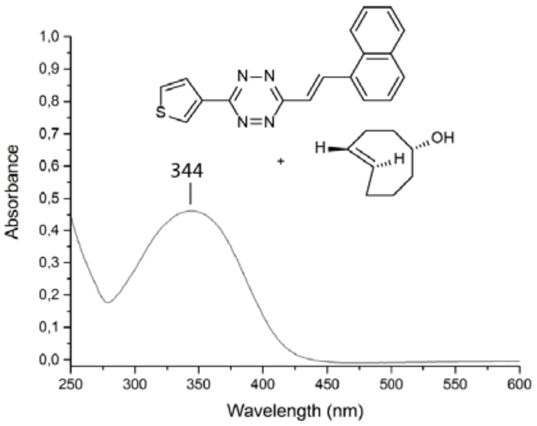
344
Absorbance
Wavelength (nm)

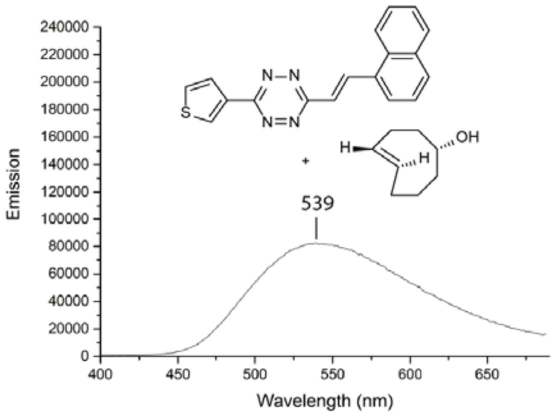
539
Emission
Wavelength (nm)

HPLC chromatograms and mass spectra of the click products using TCO 1

(please note that peaks in MS spectra with mass of the product + 41 is an artefact arising from the adduct of CH_3CN used as eluent in HPLC experiments). Conditions: solvent A: H_2O + 0.05% HCOOH; solvent B: CH_3CN + 0.05% HCOOH; gradient: 5% B → 95% B in 9 min, then 2 min 95% B and back to 5% B, Column: Luna® C18 column, 3u, 100A, 100 x 4.6 mm, 1 mL/min flow rate.

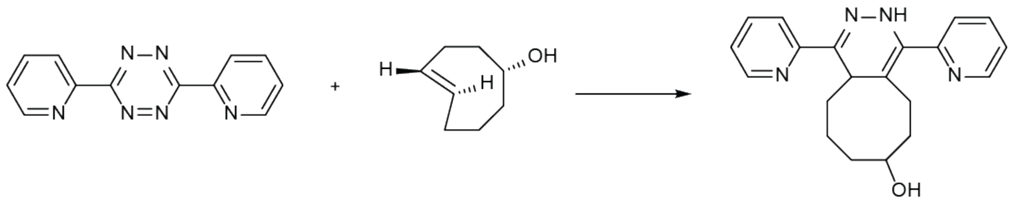

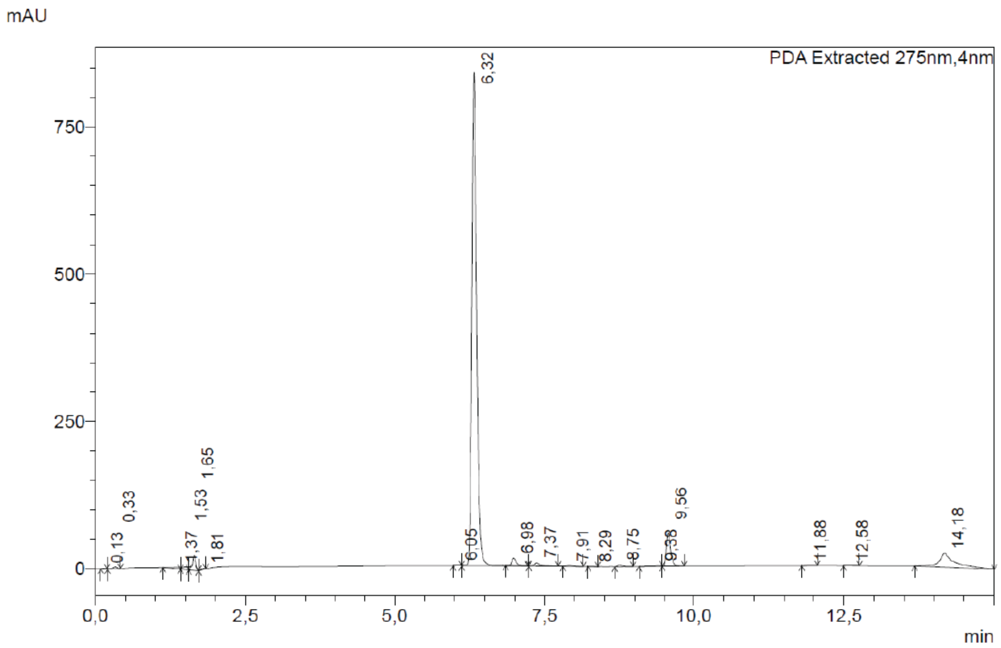

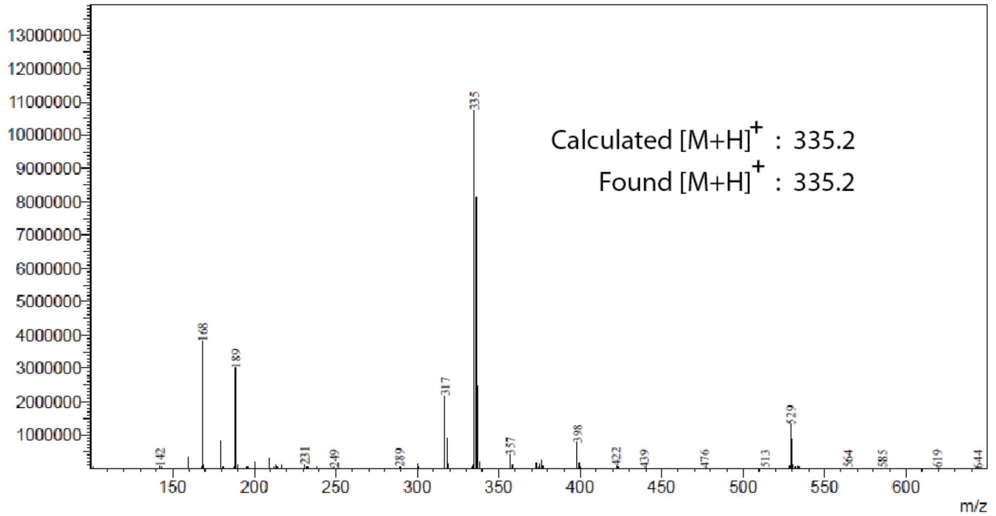

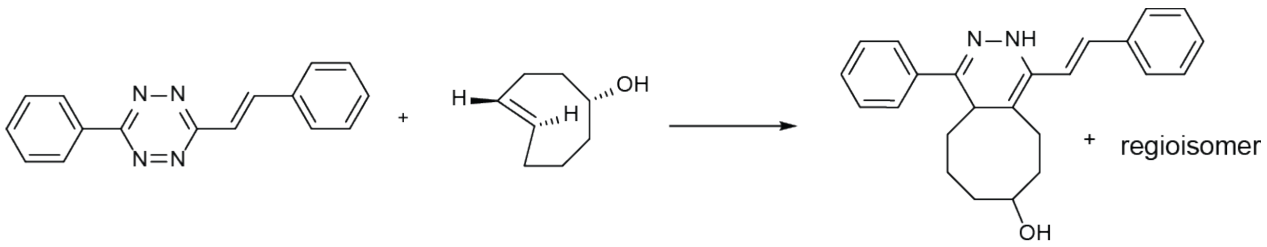
N–N
N=N
H
OH
H
N–NH
OH
+
regioisomer

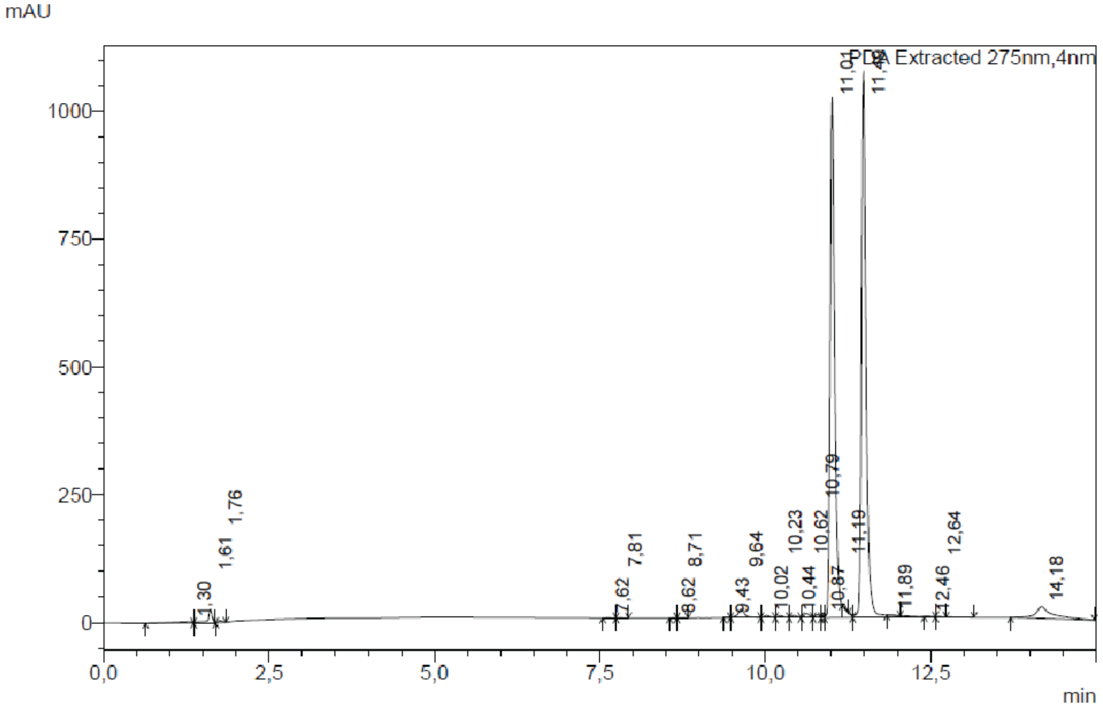
mAU
PDA Extracted 275nm,4nm
11,01
11,45
10,79
11,19
10,62
10,23
10,44
10,02
10,87
9,64
9,43
8,71
8,62
7,81
7,62
1,76
1,61
1,30
11,89
12,46
12,64
14,18
1000
750
500
250
0
0,0
2,5
5,0
7,5
10,0
12,5
min

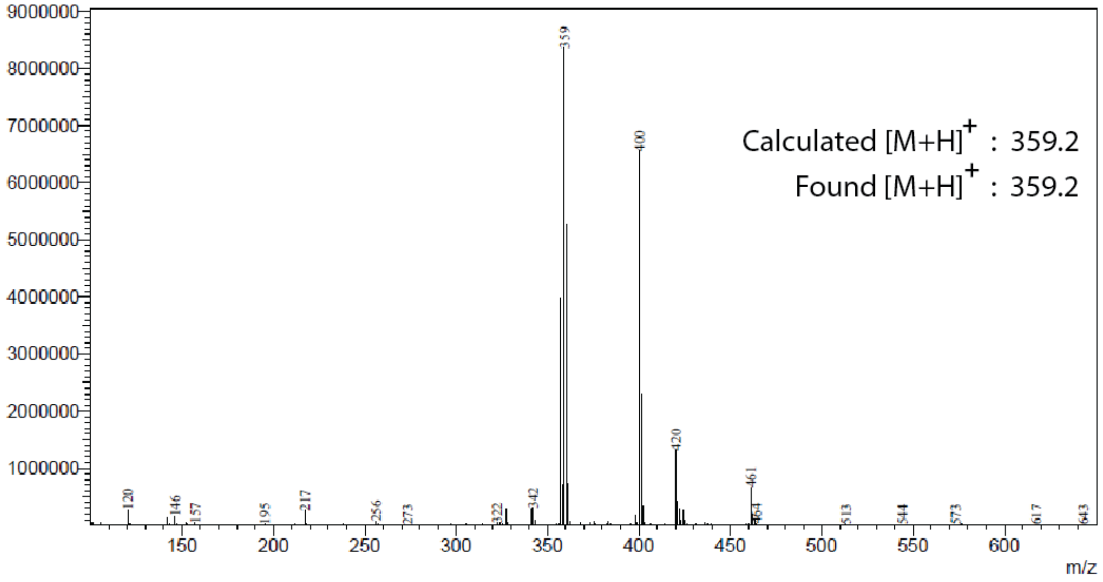
Calculated [M+H]+ : 359.2
Found [M+H]+ : 359.2
359
400
420
461
342
120
146
157
195
217
256
273
322
464
513
544
573
617
643
m/z

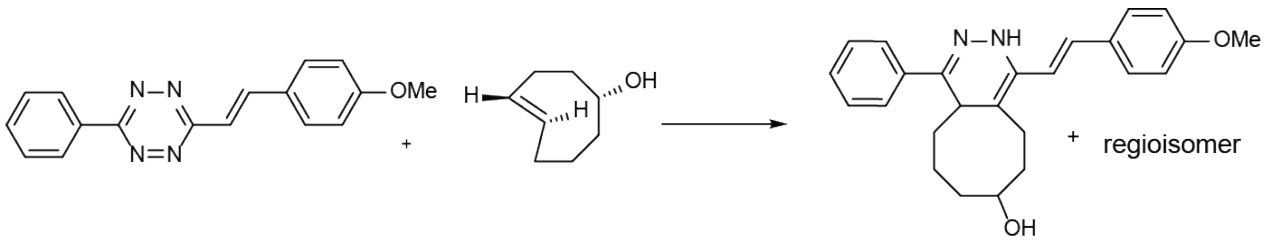
N–N
N=N
OMe
H
OH
N–NH
OMe
regioisomer
OH

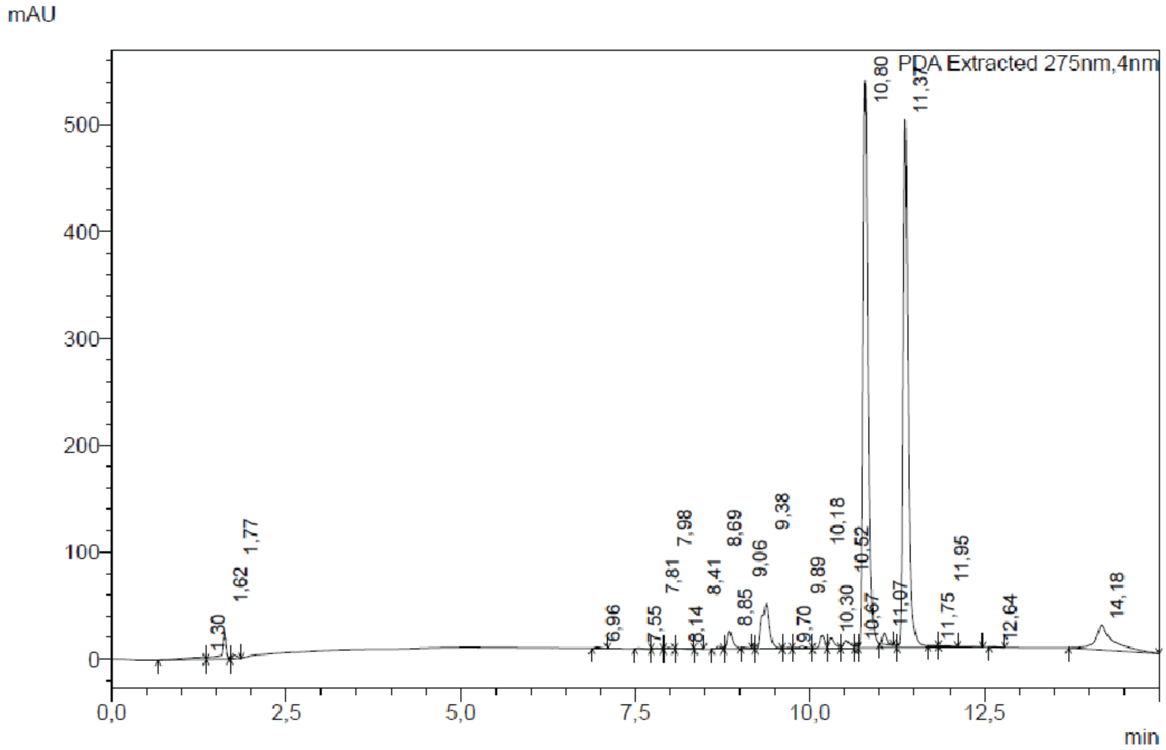
mAU
PDA Extracted 275nm,4nm
min

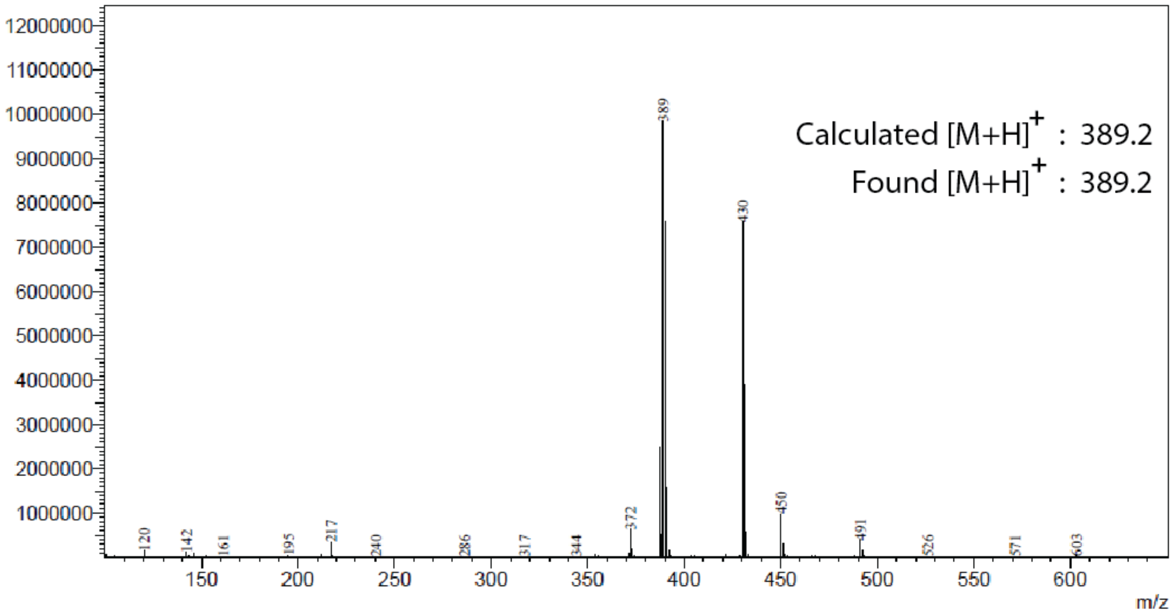
Calculated [M+H]+ : 389.2
Found [M+H]+ : 389.2
m/z

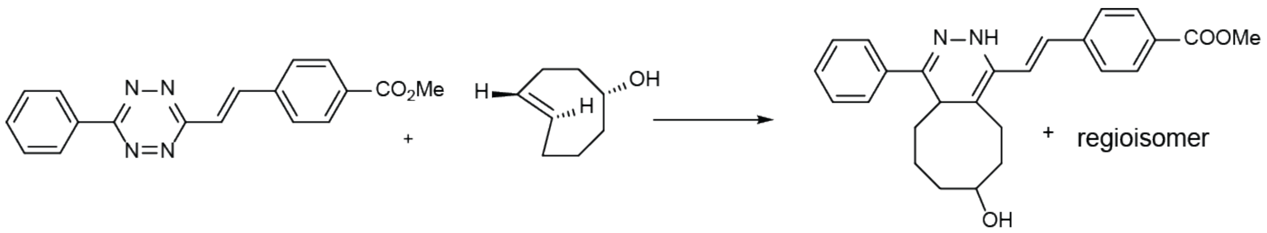
N–N
N=N
CO_2Me
+
H
OH
H
N–NH
COOMe
+ regioisomer
OH

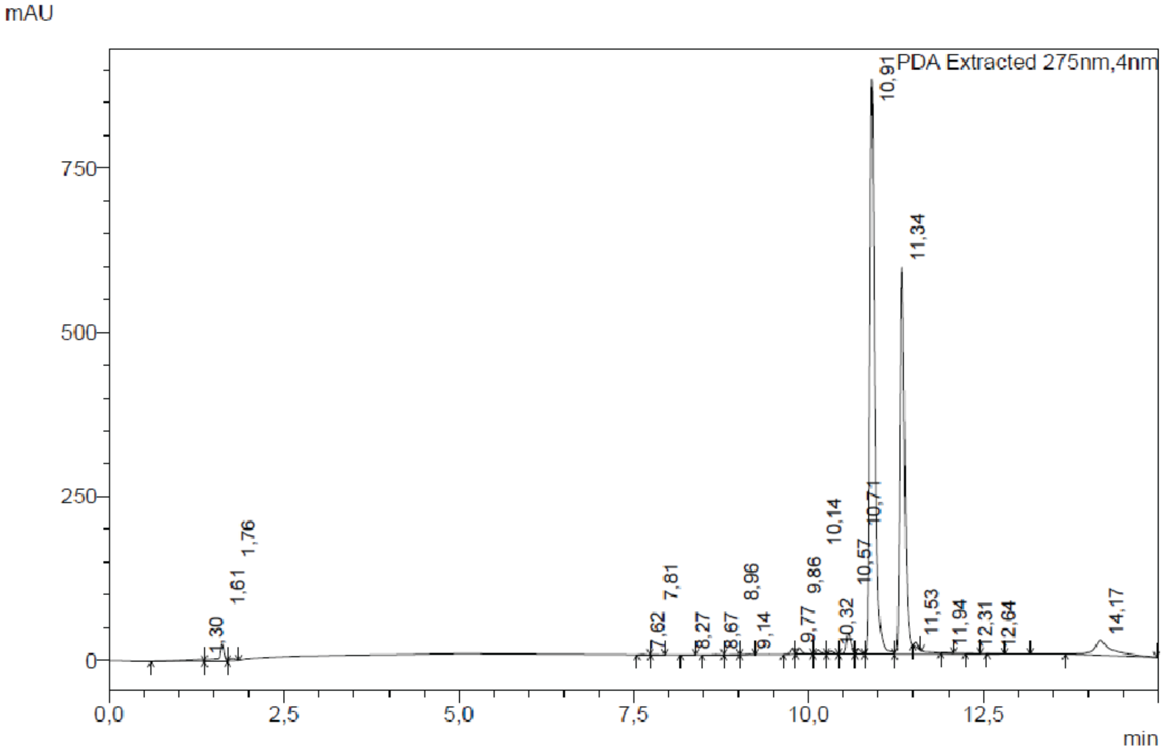
mAU
PDA Extracted 275nm,4nm
10,91
11,34
1,30
1,61
1,76
7,62
7,81
8,27
8,67
8,96
9,14
9,77
9,86
10,14
10,32
10,57
10,71
11,53
11,94
12,31
12,64
14,17
750
500
250
0
0,0
2,5
5,0
7,5
10,0
12,5
min

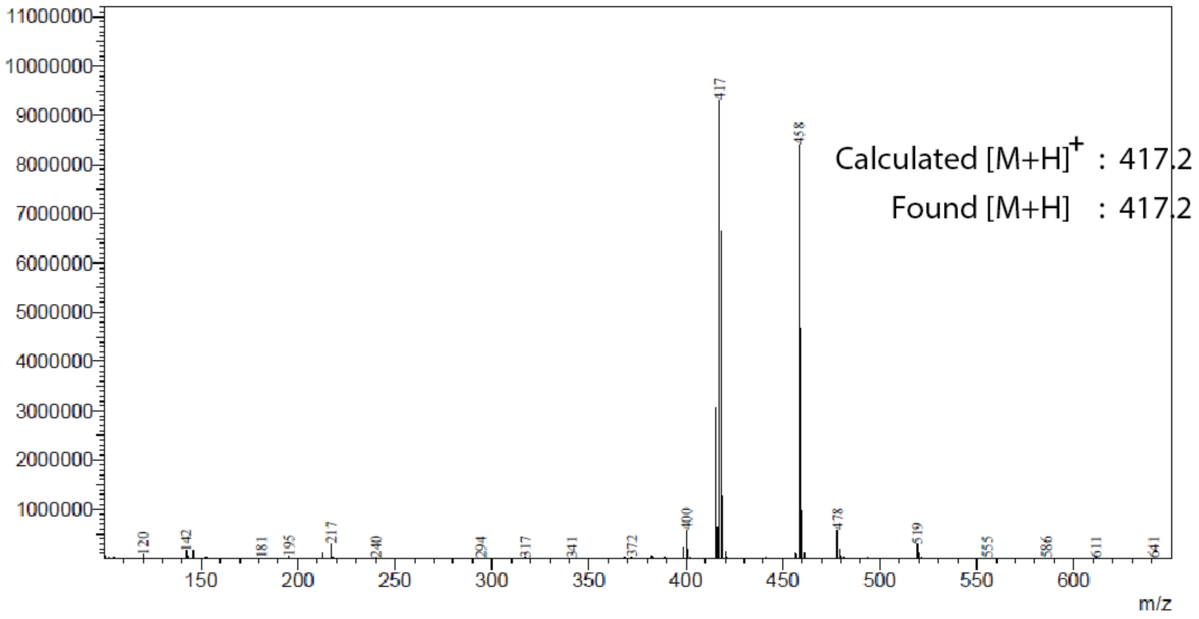
Calculated [M+H]+ : 417.2
Found [M+H] : 417.2
417
458
120
142
181
195
217
240
294
317
341
372
400
478
519
555
586
611
641
m/z

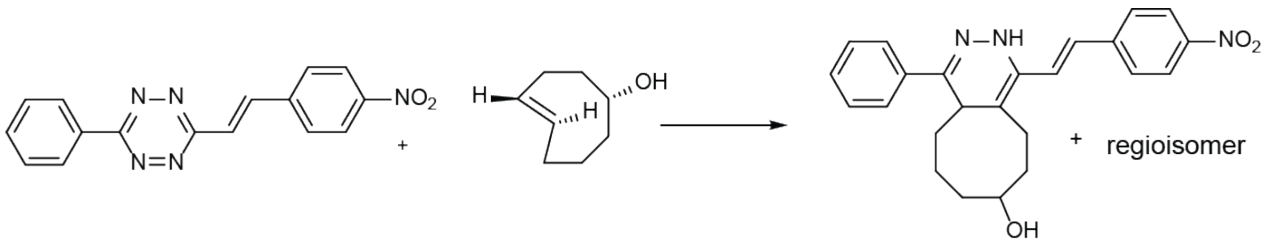
N—N
N=N
NO2
+
H
H
OH
N—NH
NO2
+ regioisomer
OH

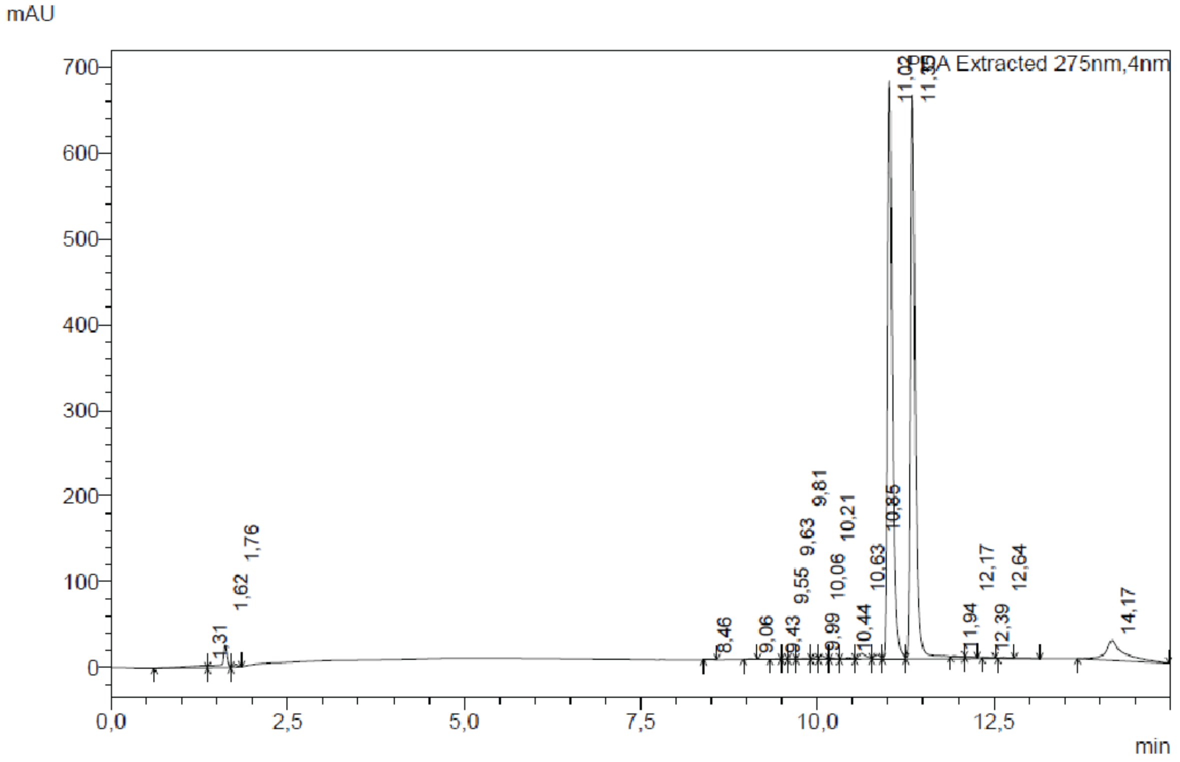
mAU
PDA Extracted 275nm,4nm
700
600
500
400
300
200
100
0
1,31
1,62
1,76
8,46
9,06
9,43
9,55
9,63
9,81
9,99
10,06
10,21
10,44
10,63
10,85
12,17
12,39
12,64
14,17
0,0
2,5
5,0
7,5
10,0
12,5
min

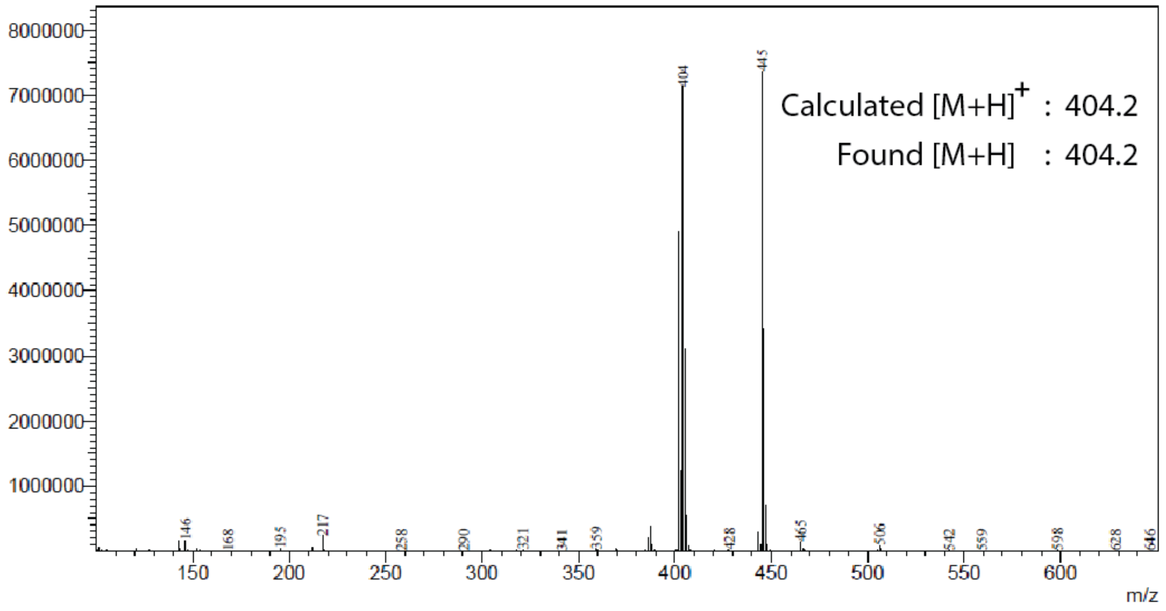
8000000
7000000
6000000
5000000
4000000
3000000
2000000
1000000
404
445
Calculated [M+H]+ : 404.2
Found [M+H] : 404.2
146
168
195
217
258
290
321
341
359
428
465
506
542
559
598
628
646
150
200
250
300
350
400
450
500
550
600
m/z

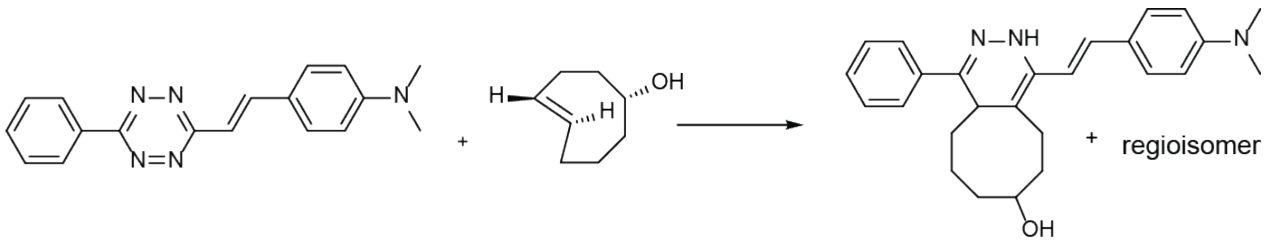
regioisomer

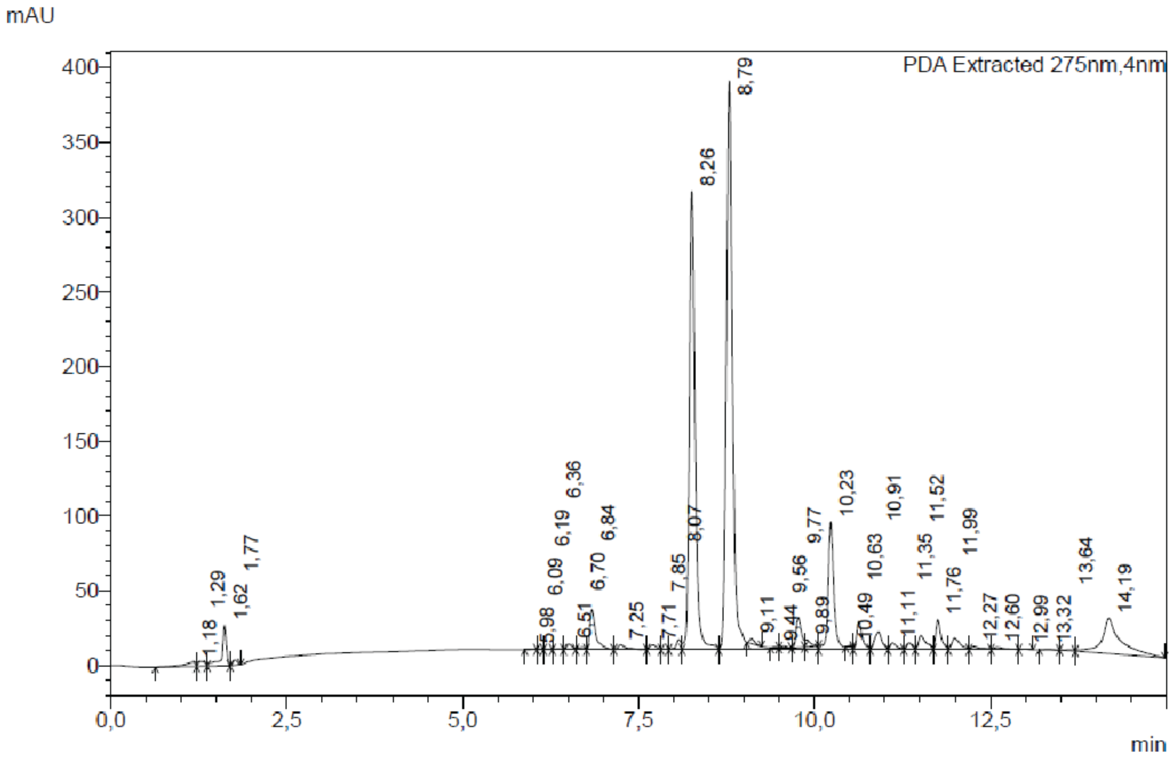
mAU
PDA Extracted 275nm,4nm
min

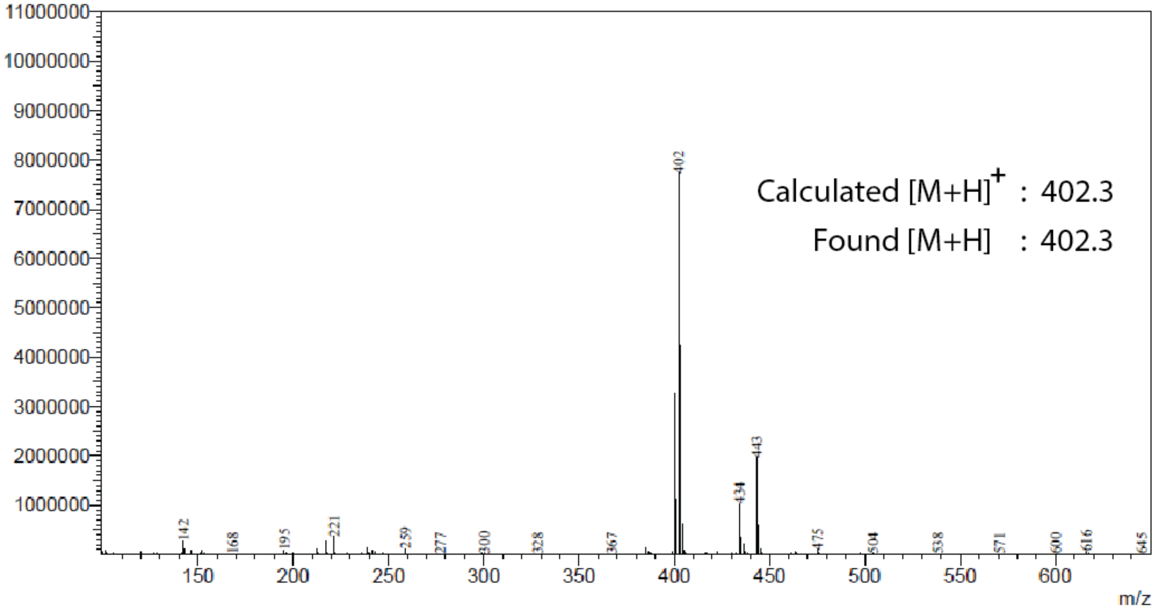
Calculated [M+H]+ : 402.3
Found [M+H] : 402.3
m/z

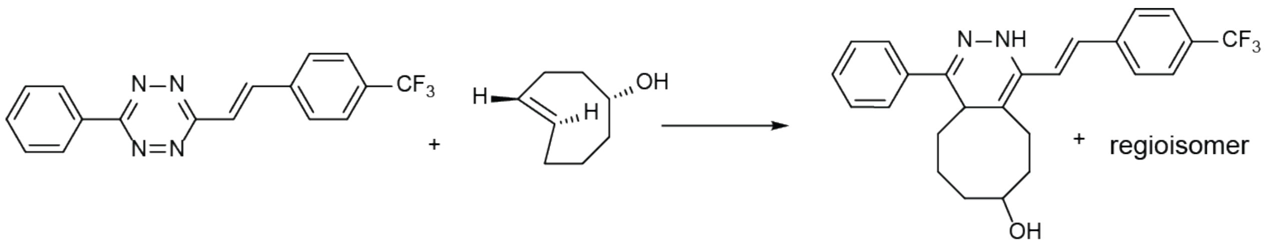

N–N
N=N
CF$_3$
H
OH
N–NH
OH
+
regioisomer

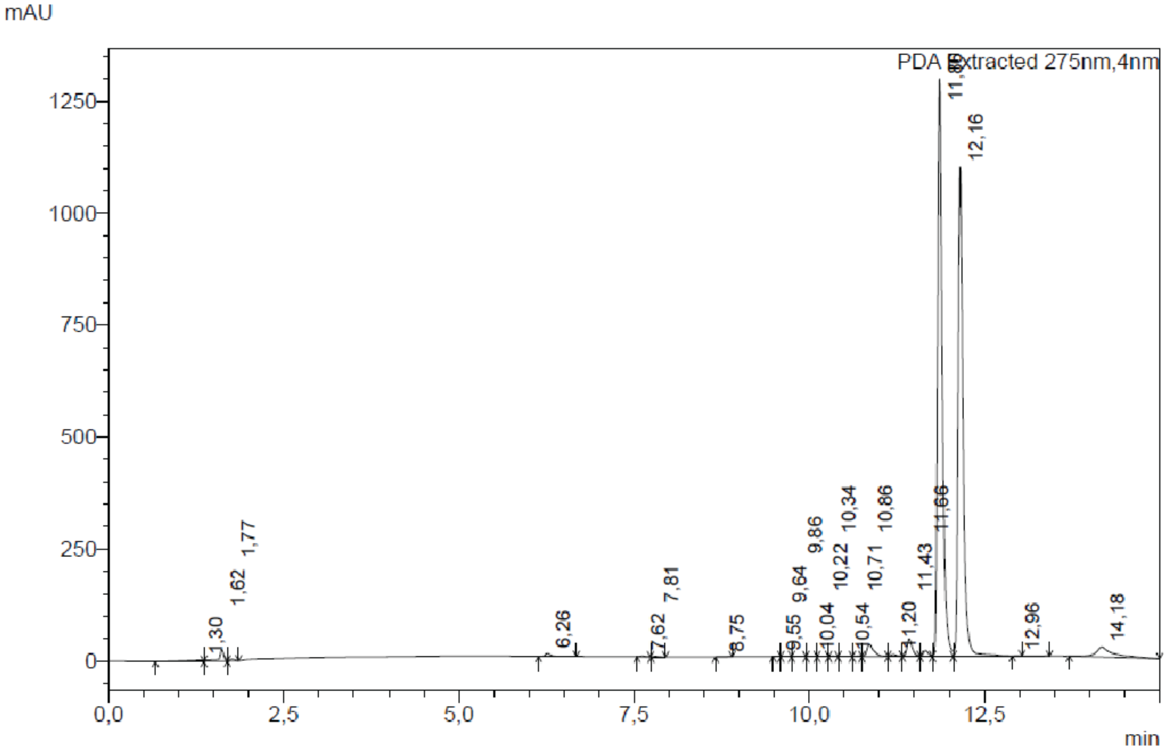

mAU
PDA Extracted 275nm,4nm
min

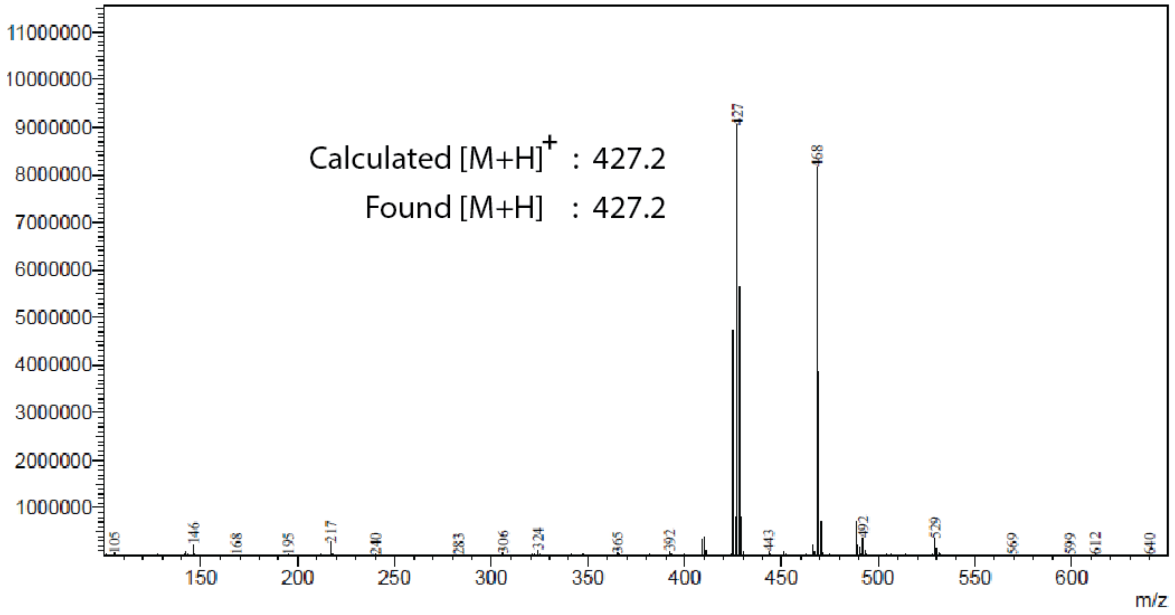

Calculated [M+H]$^+$: 427.2
Found [M+H] : 427.2
m/z

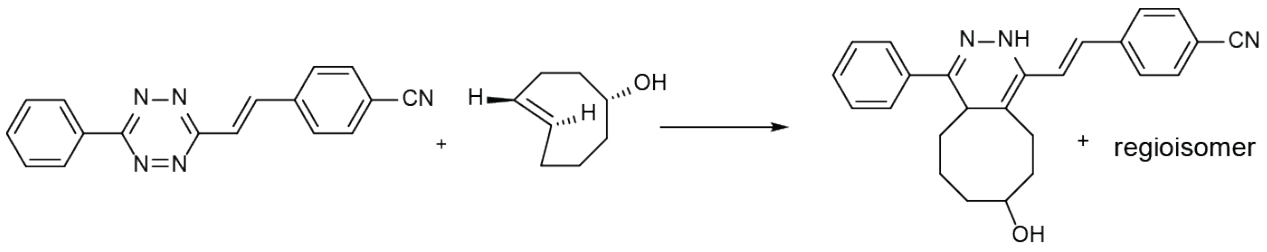
N–N
N=N
CN
H
H
OH
N–NH
CN
+ regioisomer
OH

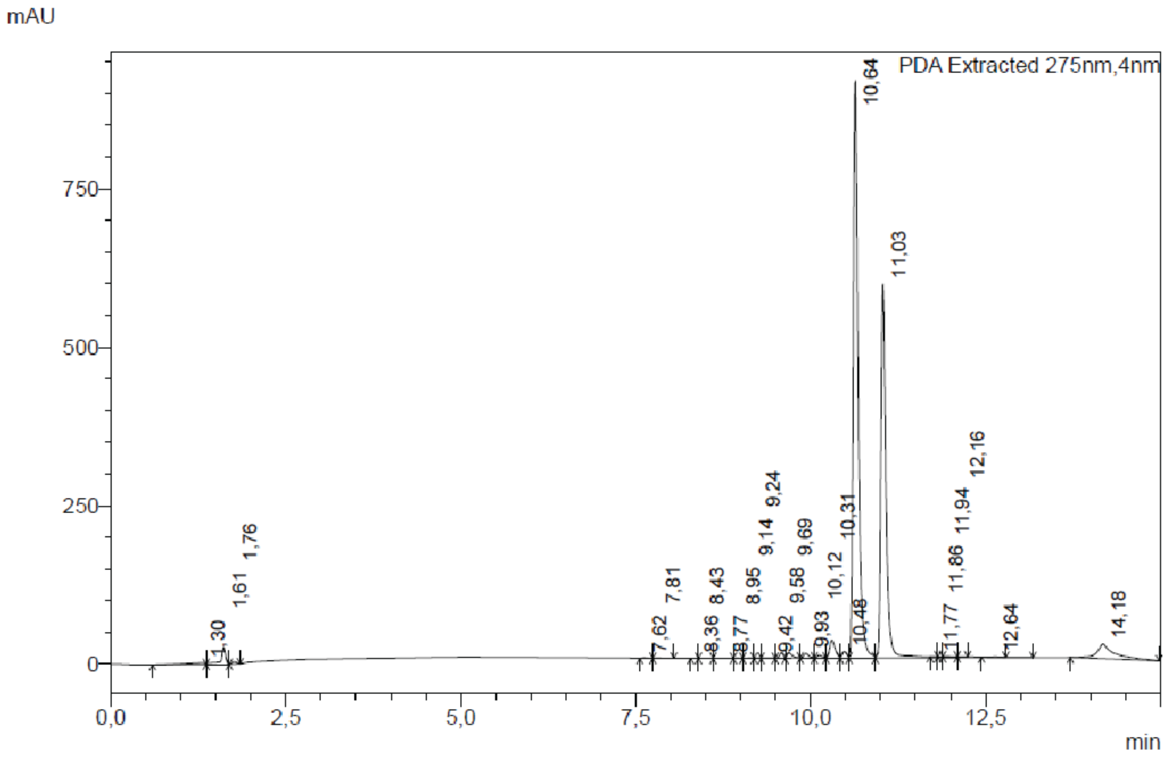
mAU
PDA Extracted 275nm,4nm
750
500
250
0
0,0
2,5
5,0
7,5
10,0
12,5
min
10,64
11,03
12,16
9,24
11,94
10,31
9,14
9,69
11,86
10,12
1,76
1,61
7,81
8,43
8,95
9,58
14,18
12,64
11,77
10,48
9,93
9,42
8,77
8,36
7,62
1,30

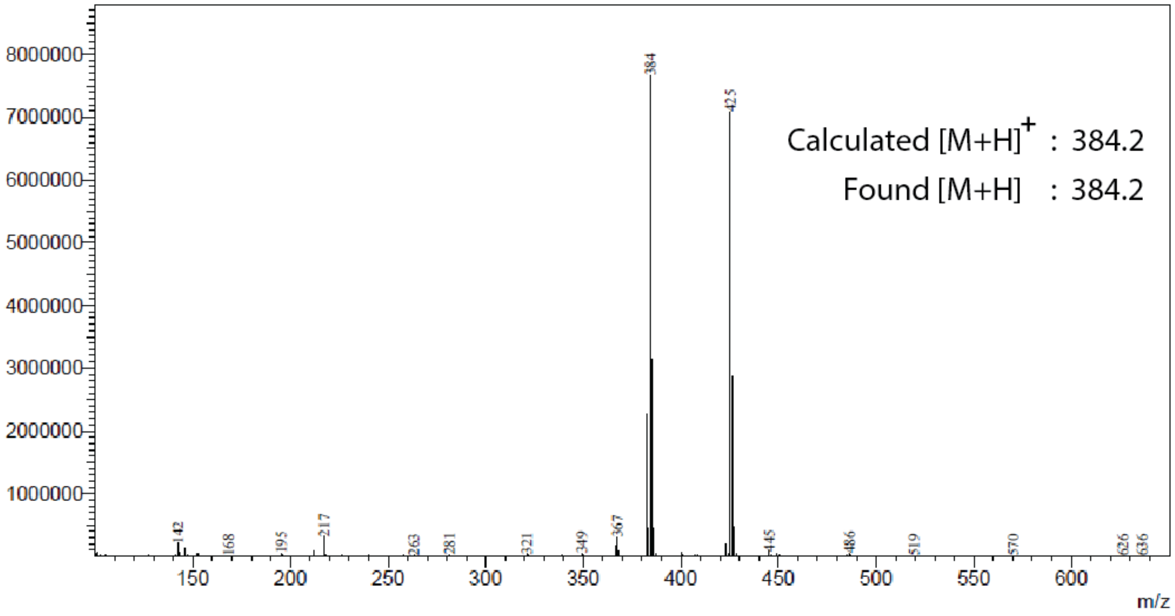
8000000
7000000
6000000
5000000
4000000
3000000
2000000
1000000
384
425
Calculated [M+H]+ : 384.2
Found [M+H] : 384.2
142
168
195
217
263
281
321
349
367
445
486
519
570
626
636
150
200
250
300
350
400
450
500
550
600
m/z

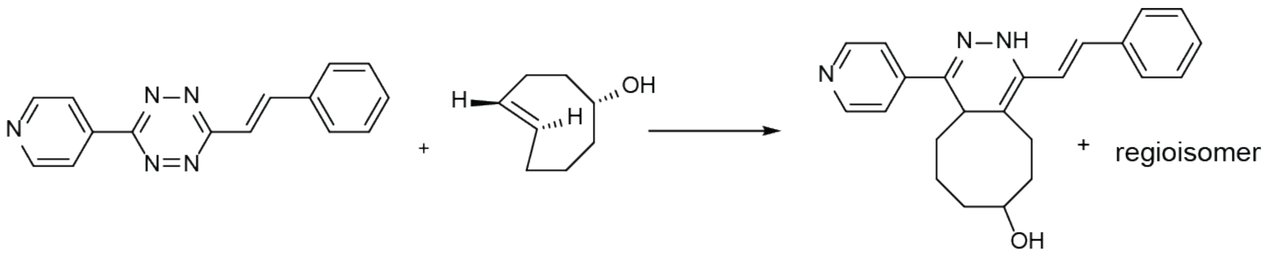
N
N–N
N=N
H
H
OH
+
N–NH
N
OH
+ regioisomer

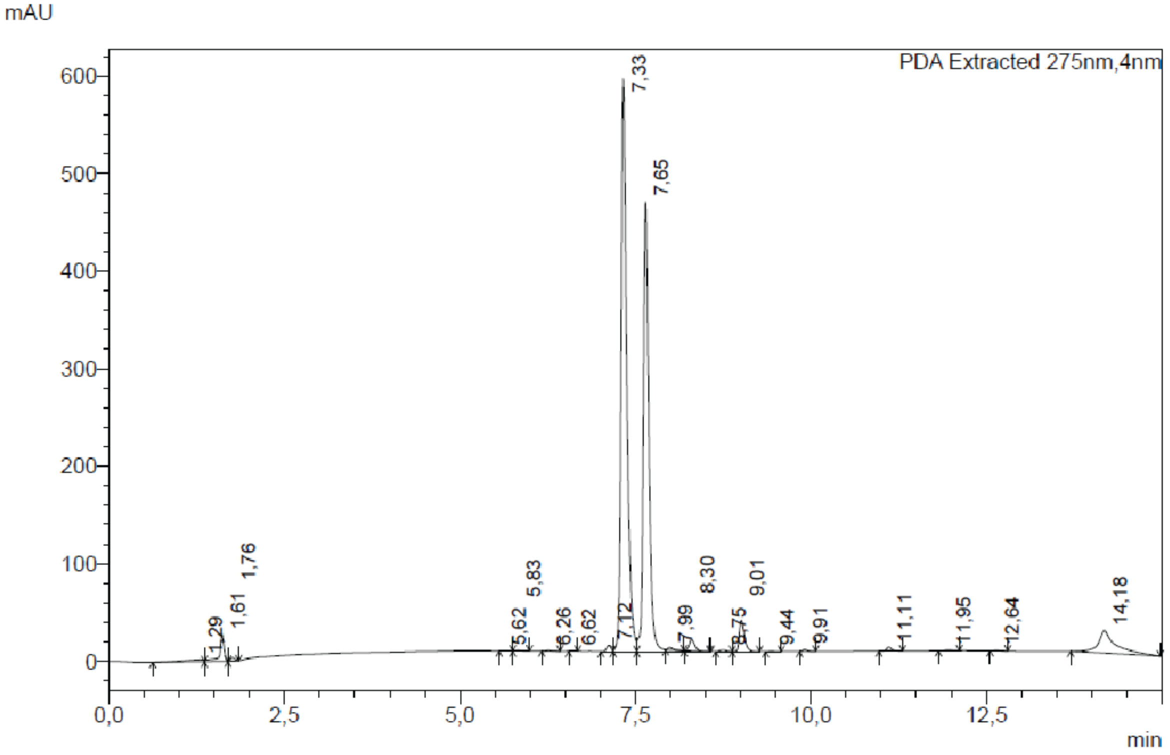
mAU
PDA Extracted 275nm,4nm
7,33
7,65
1,29
1,61
1,76
5,62
5,83
6,26
6,62
7,12
7,99
8,30
8,75
9,01
9,44
9,91
11,11
11,95
12,64
14,18
min

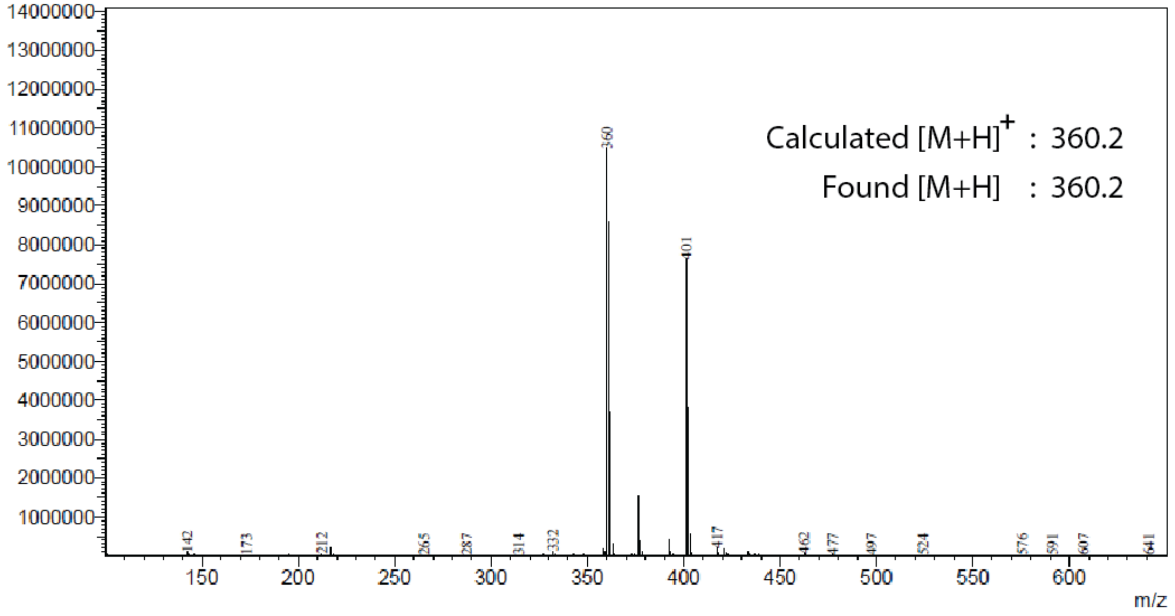
Calculated [M+H]+ : 360.2
Found [M+H] : 360.2
360
401
142
173
212
265
287
314
332
417
462
477
497
524
576
591
607
641
m/z

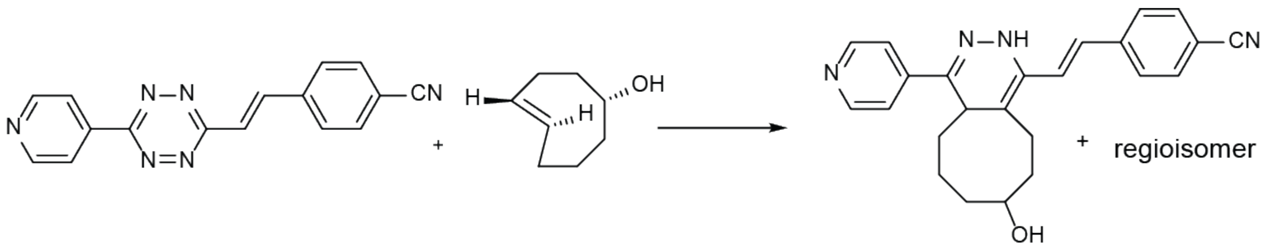
N
N–N
N=N
CN
H
H
OH
+
N–NH
N
CN
OH
+ regioisomer

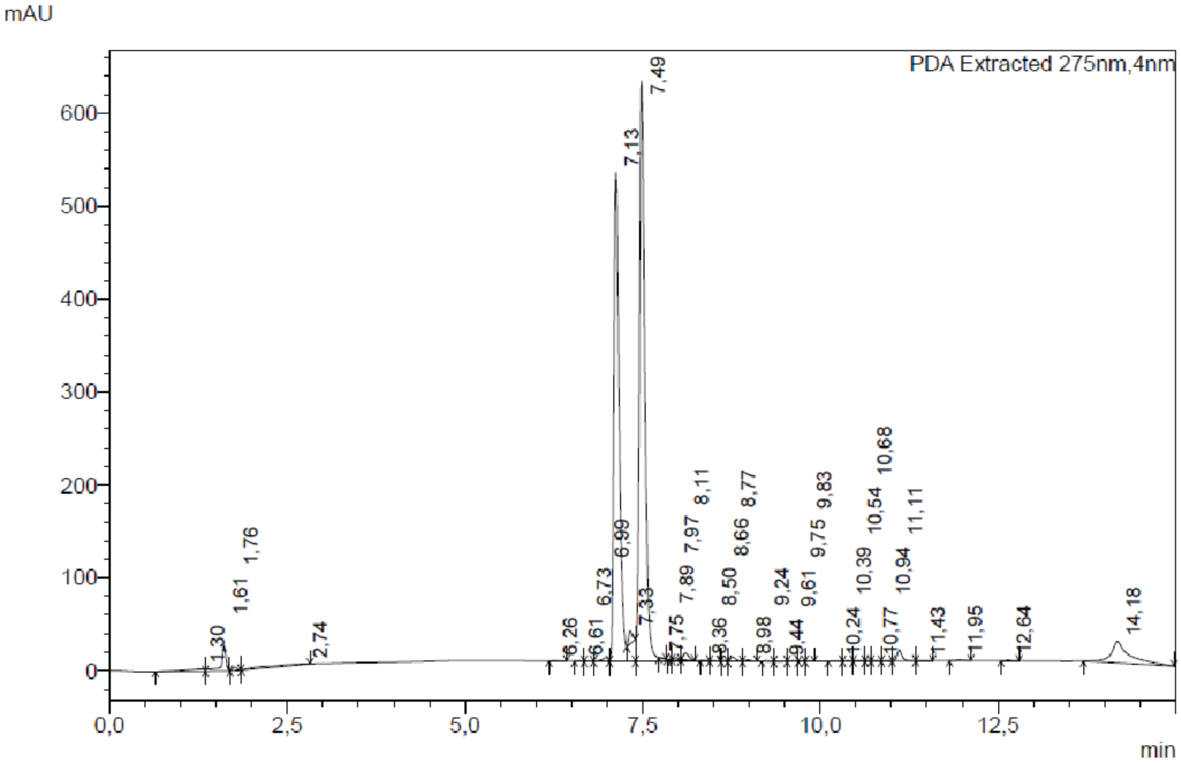
mAU
PDA Extracted 275nm,4nm
min

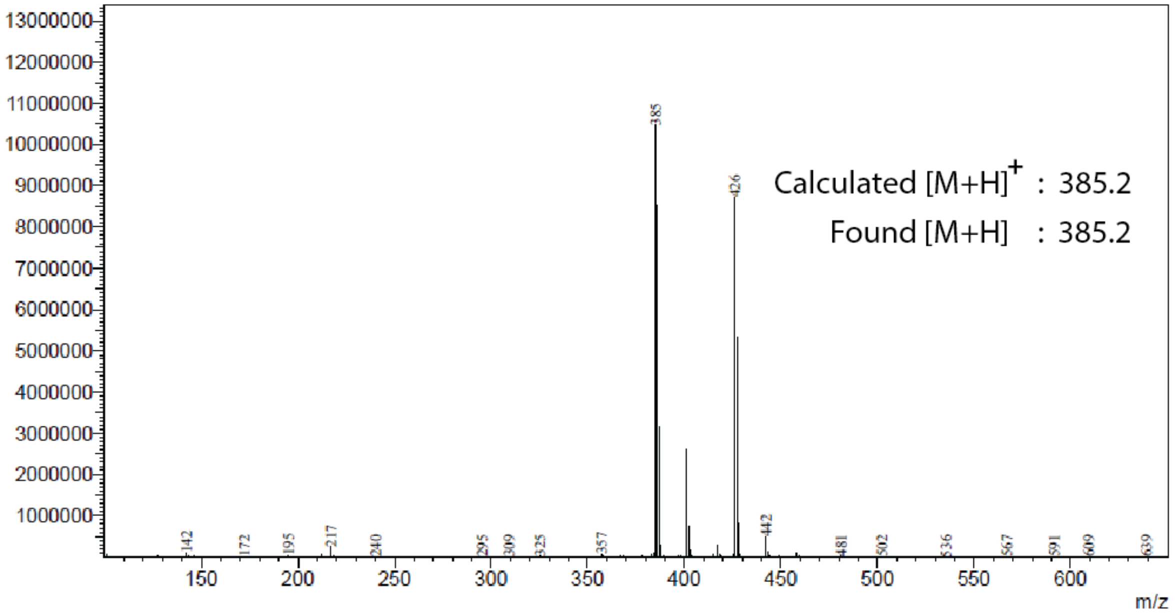
Calculated [M+H]+ : 385.2
Found [M+H] : 385.2
m/z

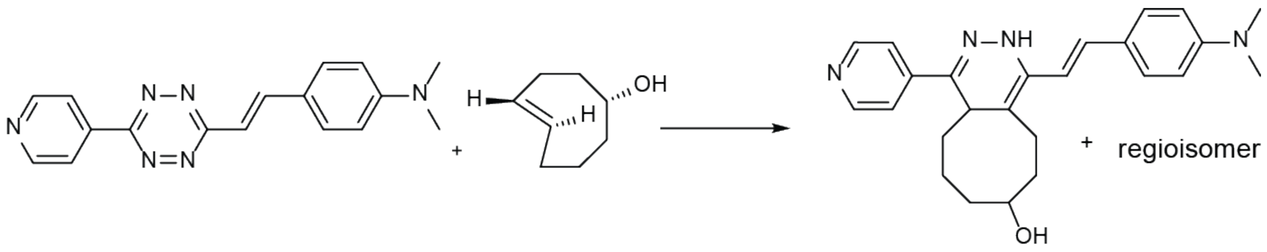
N—N
N=N
H
H
OH
N
N—NH
OH
+ regioisomer

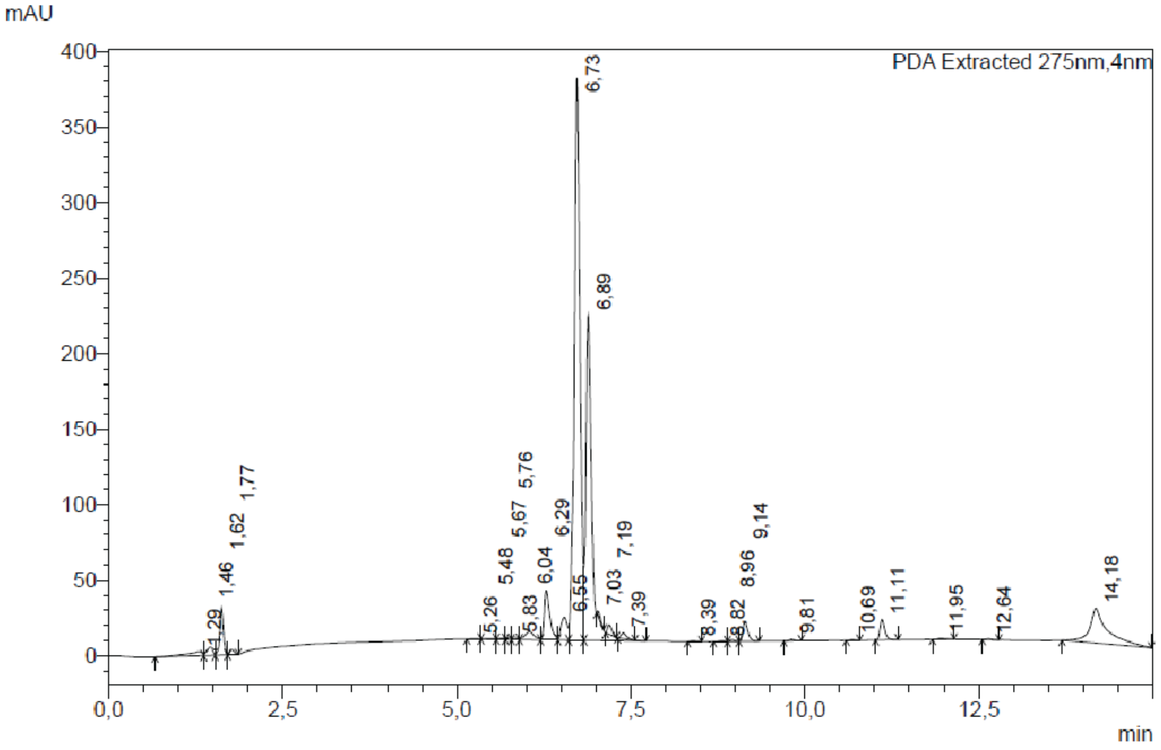
mAU
PDA Extracted 275nm,4nm
min

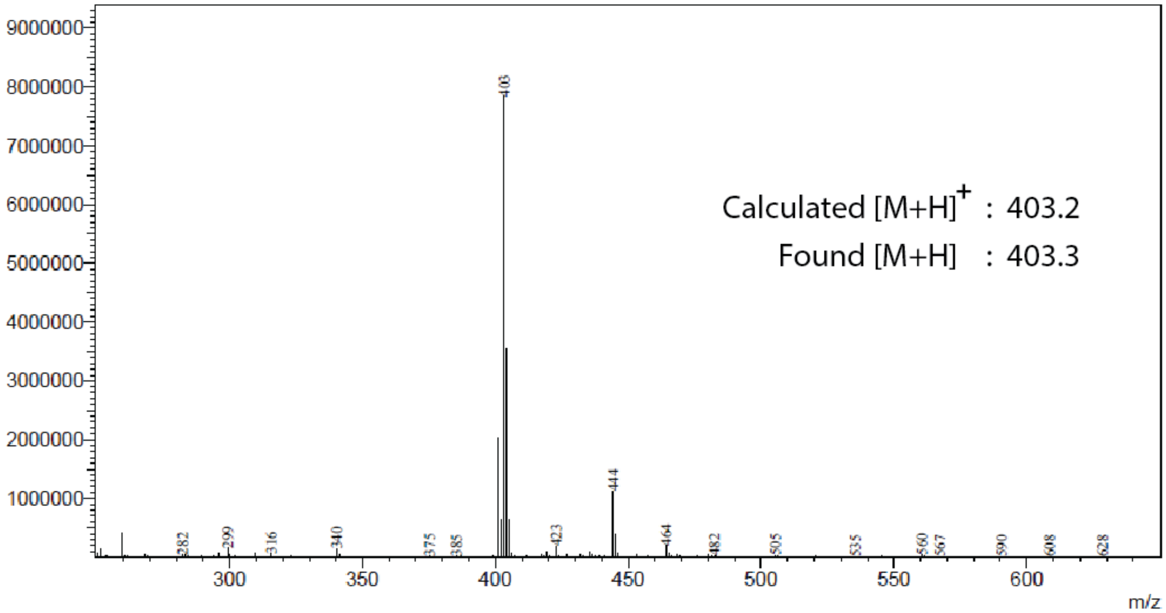
Calculated [M+H]$^+$: 403.2
Found [M+H] : 403.3
m/z

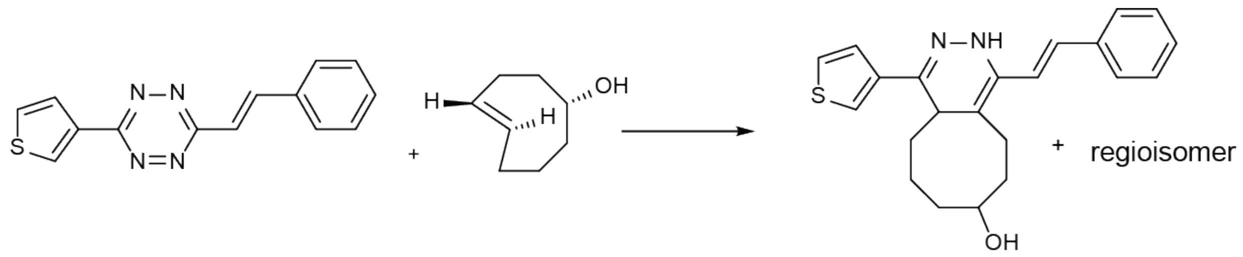

N–N
N=N
S
H
OH
H
N–NH
OH
+
regioisomer

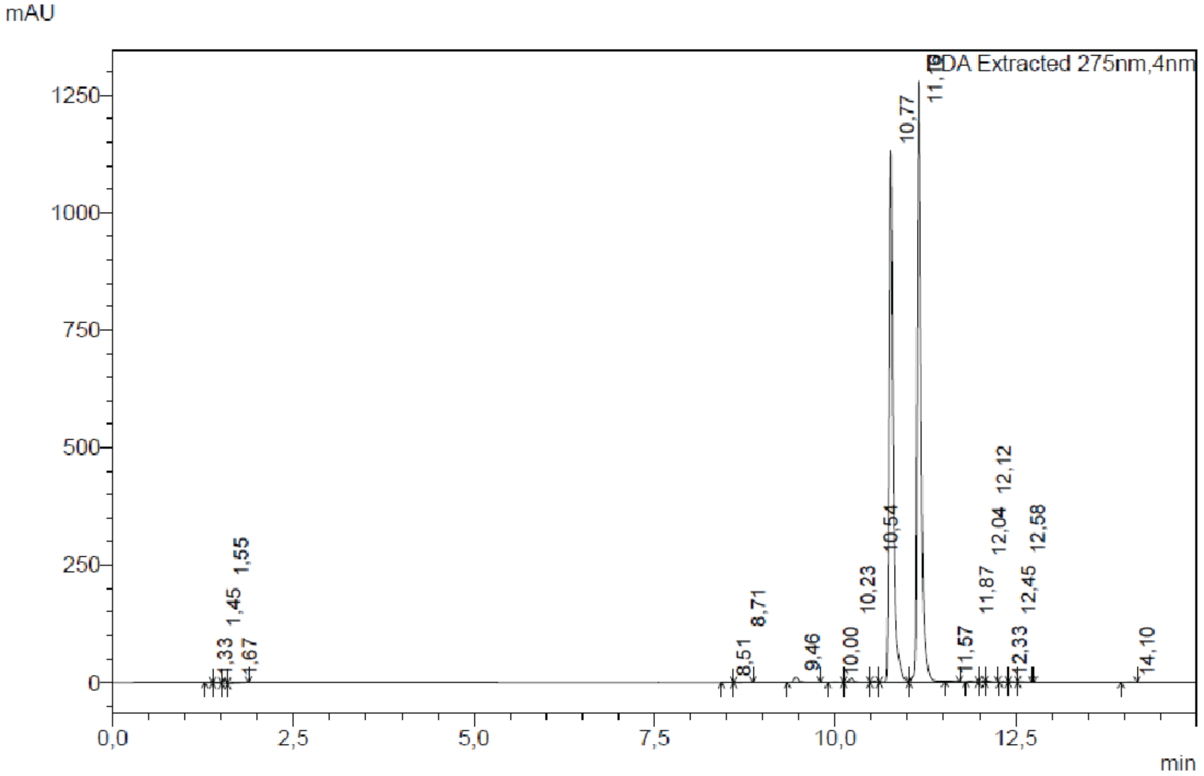

mAU
PDA Extracted 275nm,4nm
1250
1000
750
500
250
0
0,0
2,5
5,0
7,5
10,0
12,5
min
10,77
1,33
1,45
1,55
1,67
8,51
8,71
9,46
10,00
10,23
10,54
11,57
11,87
12,04
12,12
12,33
12,45
12,58
14,10

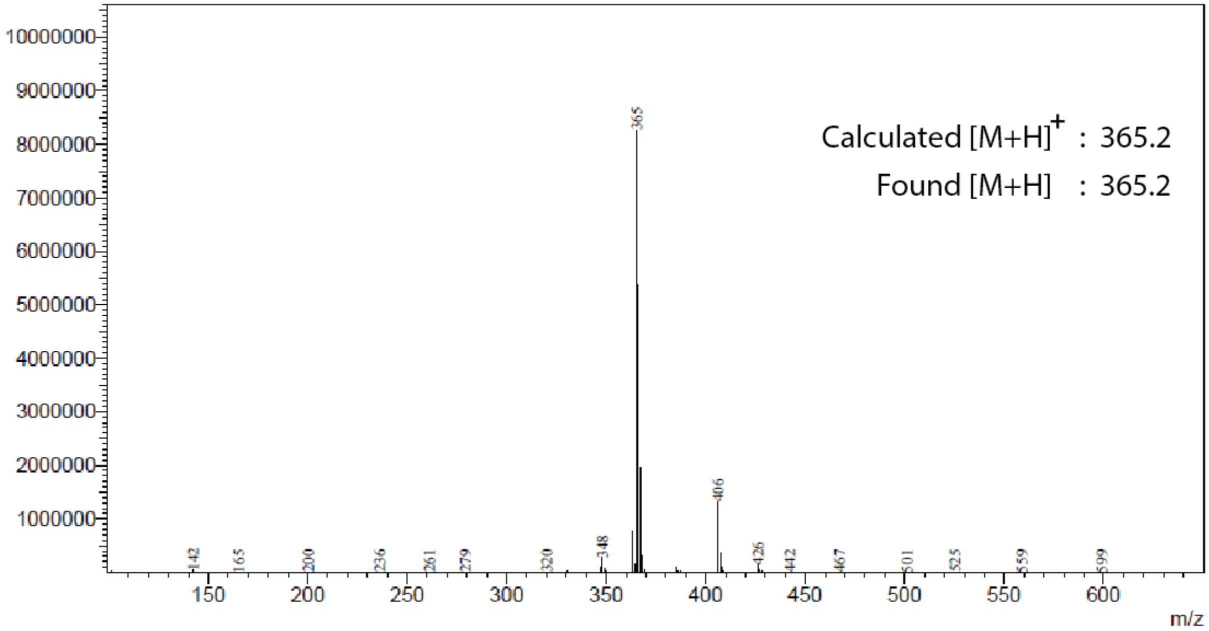

10000000
9000000
8000000
7000000
6000000
5000000
4000000
3000000
2000000
1000000
365
406
348
150
200
250
300
350
400
450
500
550
600
m/z
Calculated [M+H]+ : 365.2
Found [M+H] : 365.2

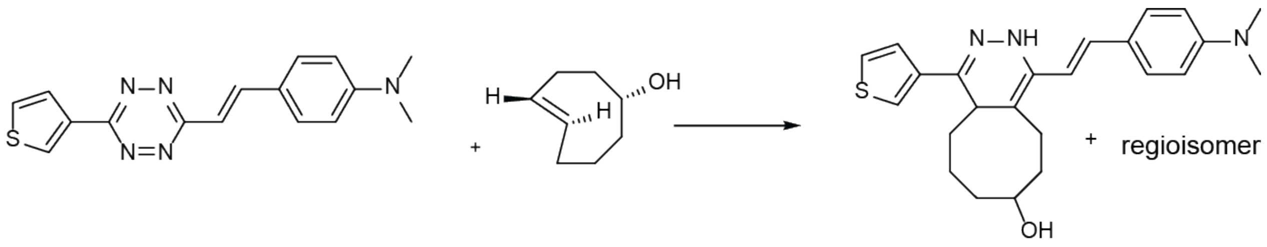
N–N
N=N
S
N
H
OH
H
N–NH
S
OH
+
+ regioisomer

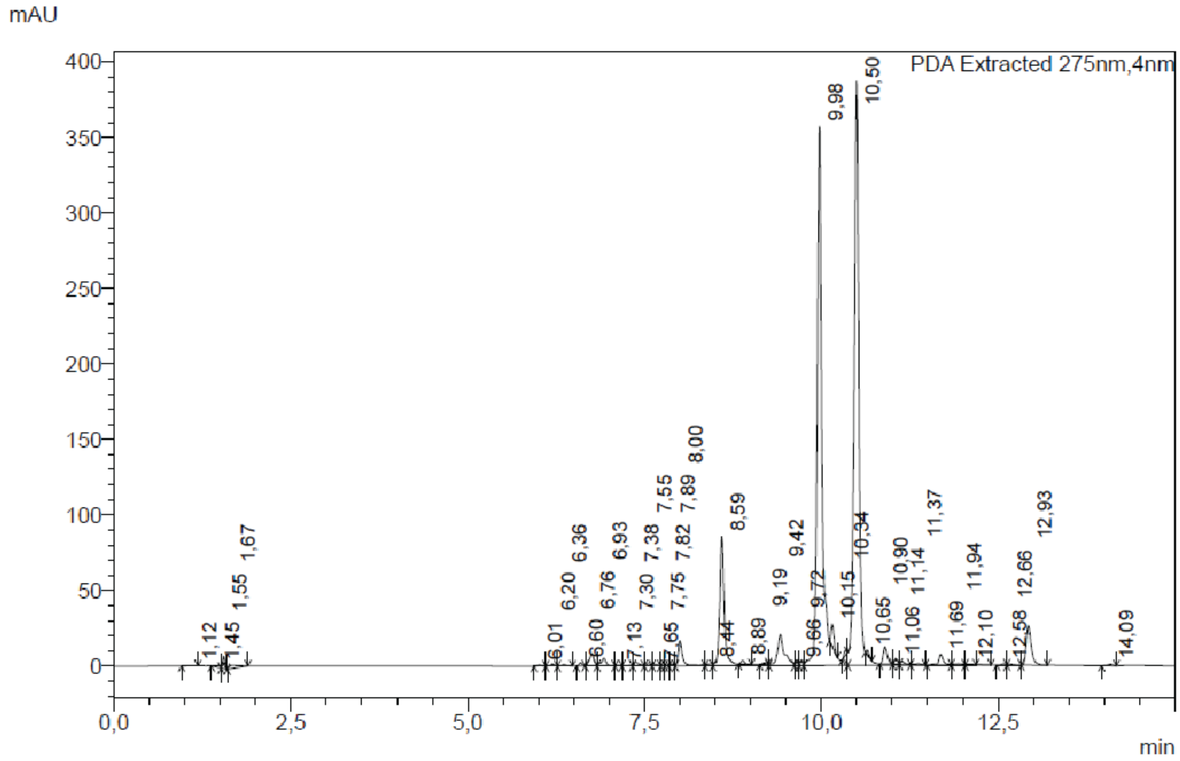
mAU
PDA Extracted 275nm,4nm
min
9,98
10,50
8,59
8,00

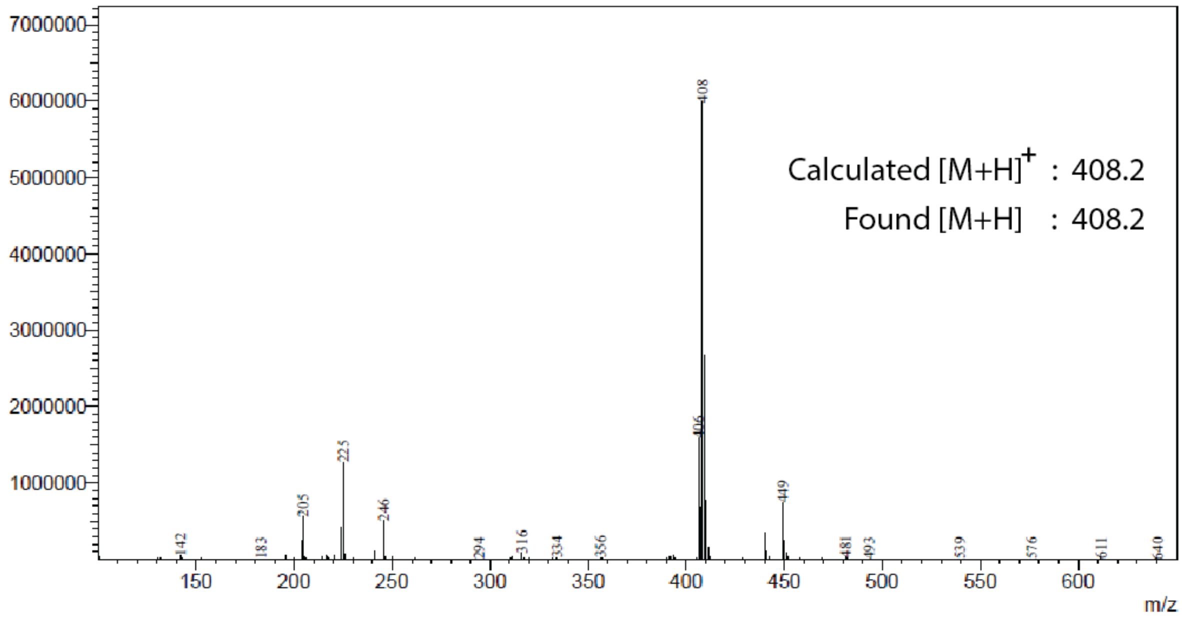
Calculated [M+H]+ : 408.2
Found [M+H] : 408.2
m/z
408
225
449

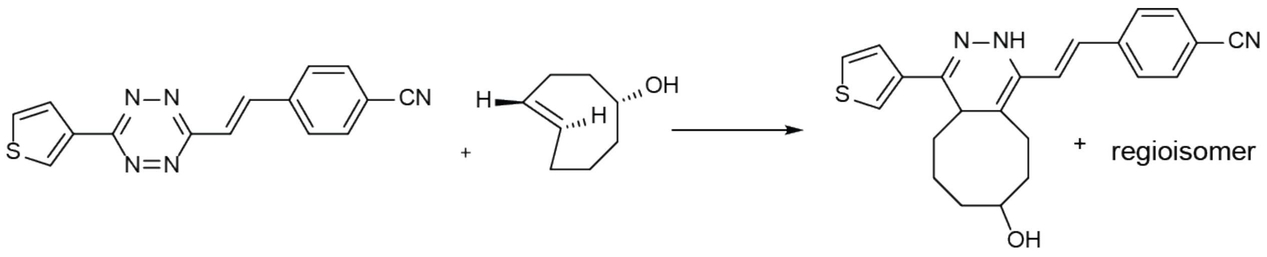
N–N
N=N
CN
S
H
OH
H
N–NH
CN
S
OH
+ regioisomer

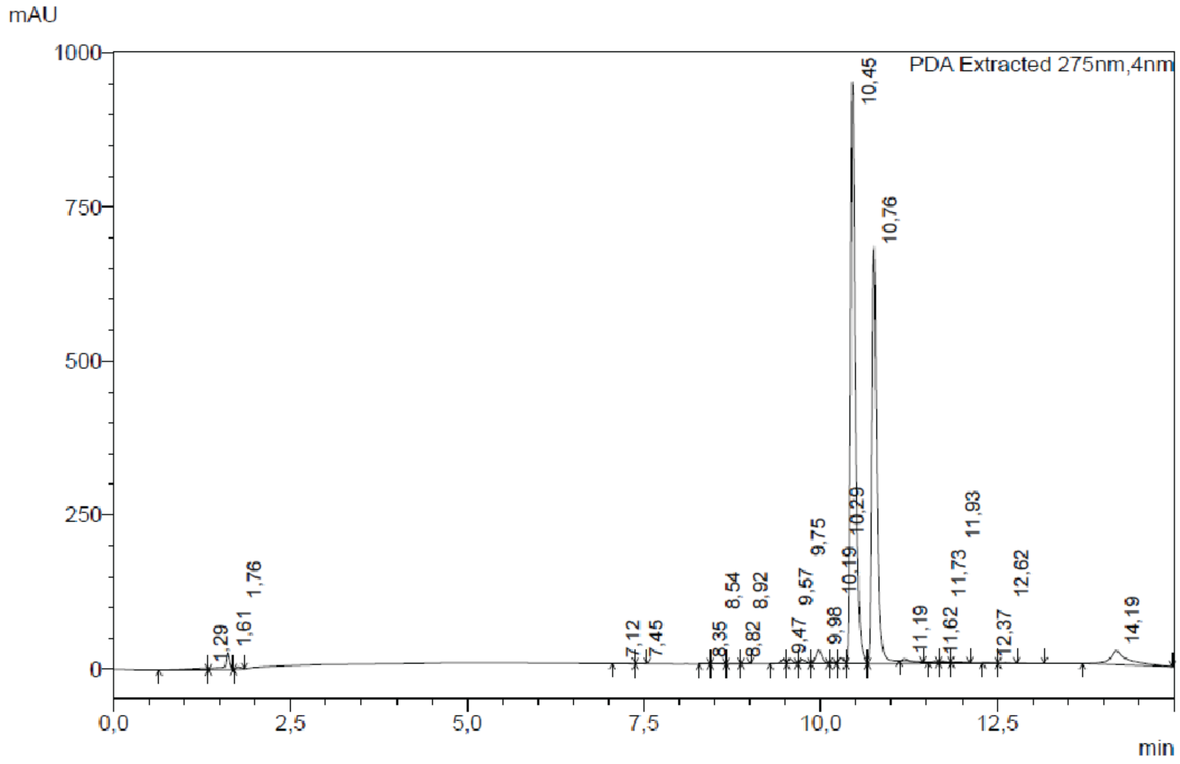
mAU
PDA Extracted 275nm,4nm
1000
750
500
250
0
0,0
2,5
5,0
7,5
10,0
12,5
min
10,45
10,76
1,29
1,61
1,76
7,12
7,45
8,35
8,54
8,82
8,92
9,47
9,57
9,75
9,98
10,19
10,29
11,19
11,62
11,73
11,93
12,37
12,62
14,19

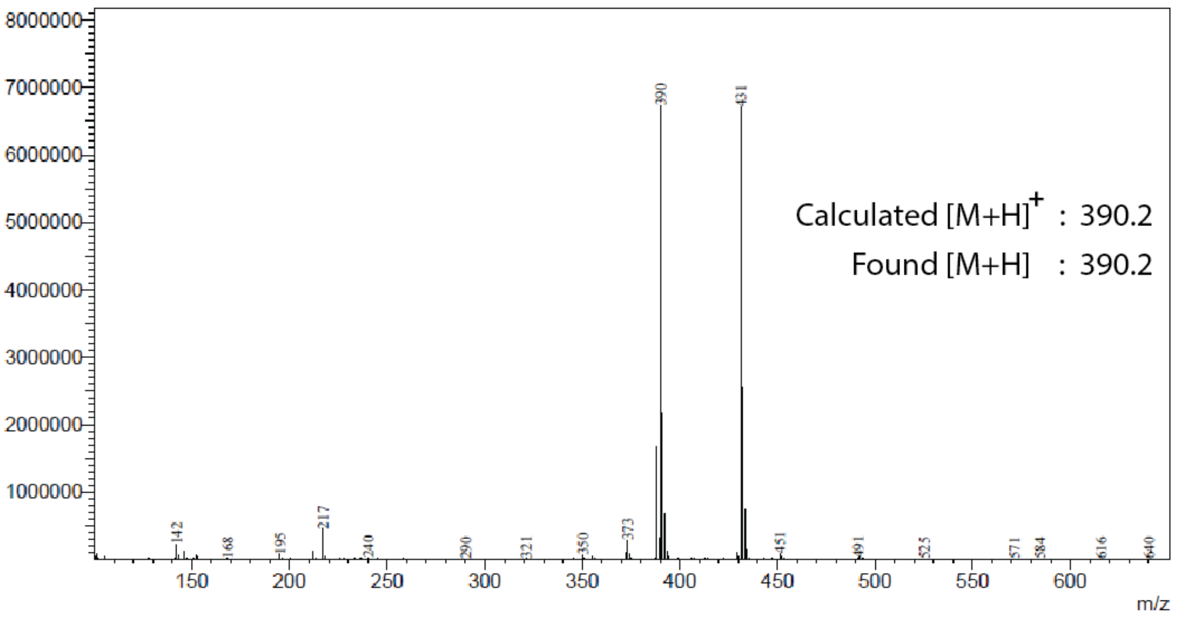
8000000
7000000
6000000
5000000
4000000
3000000
2000000
1000000
150
200
250
300
350
400
450
500
550
600
m/z
390
431
142
168
195
217
240
290
321
350
373
451
491
525
571
584
616
640
Calculated [M+H]+ : 390.2
Found [M+H] : 390.2

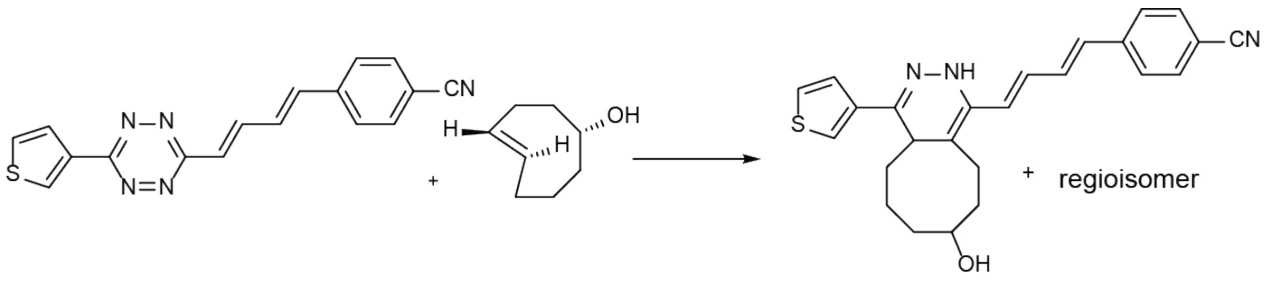
N–N
N=N
S
CN
H
H
OH
+
N–NH
OH
+ regioisomer

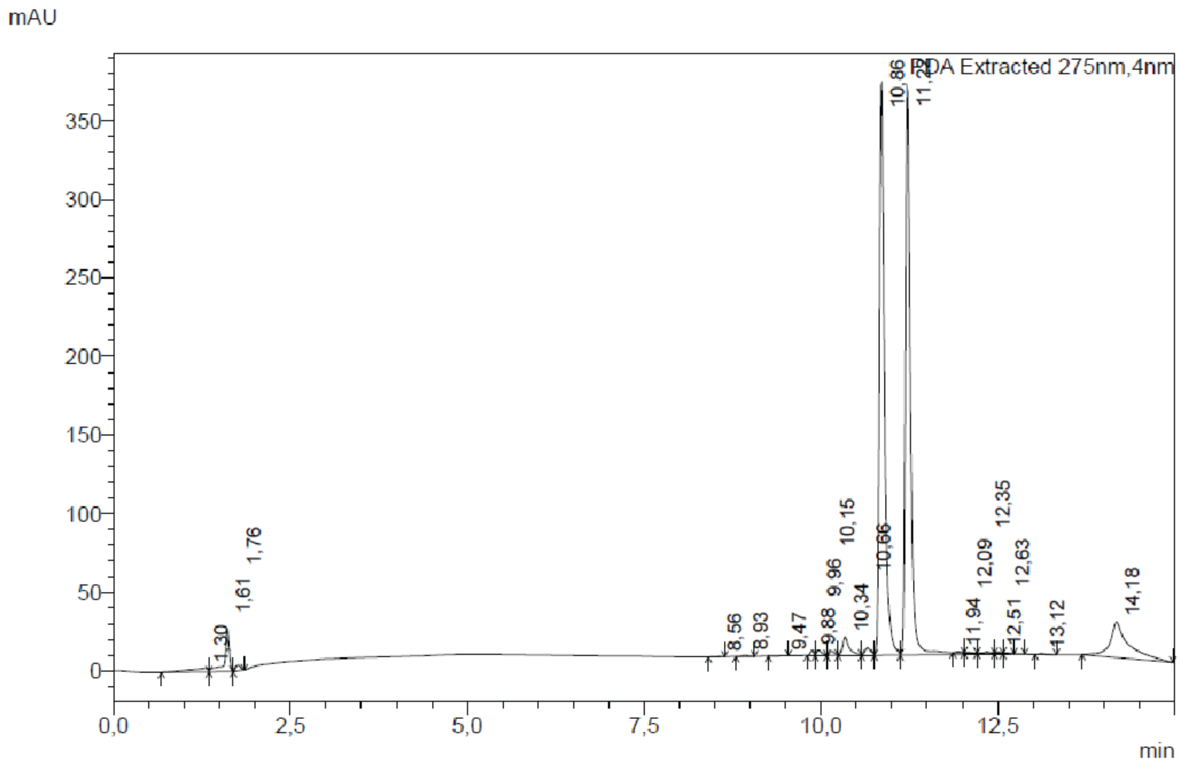
mAU
PDA Extracted 275nm,4nm
min

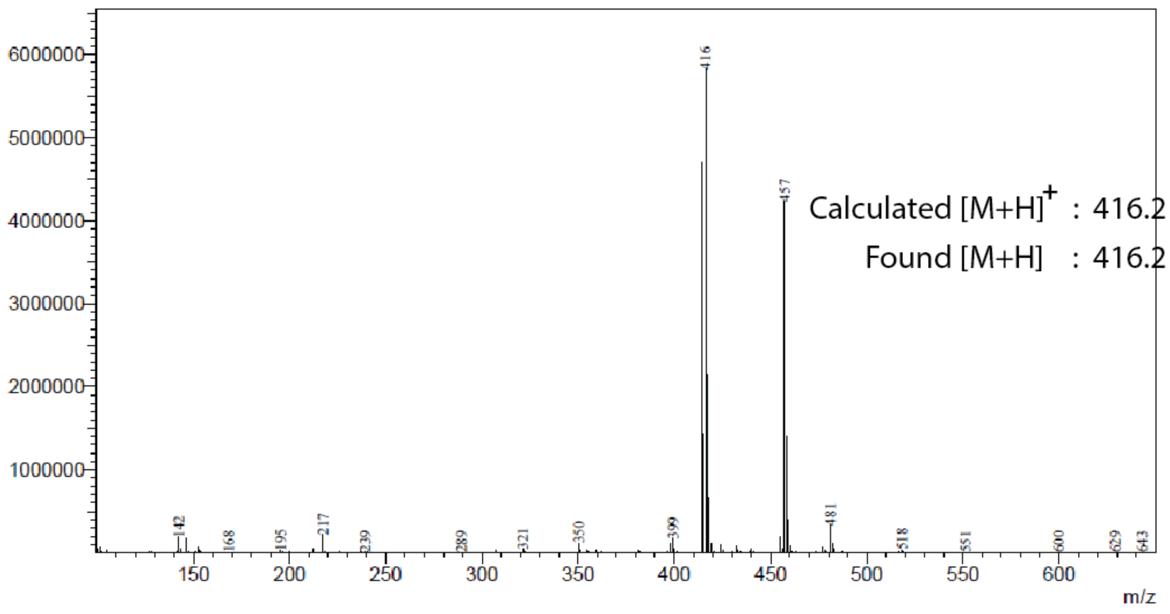
Calculated [M+H]+ : 416.2
Found [M+H] : 416.2
m/z

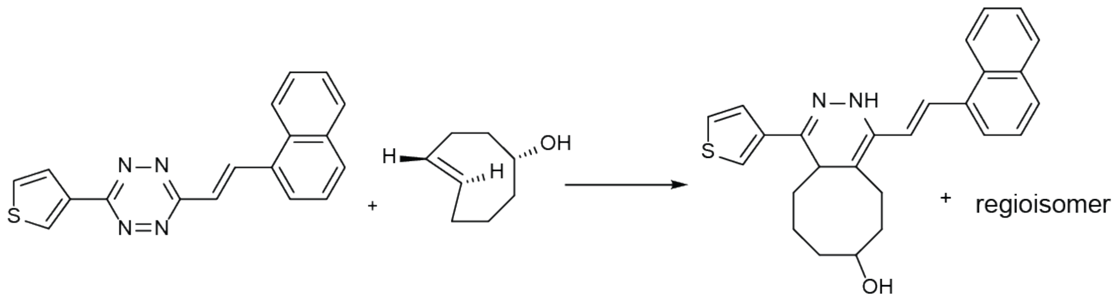

N–N
N=N
S
H
OH
N–NH
OH
+ regioisomer

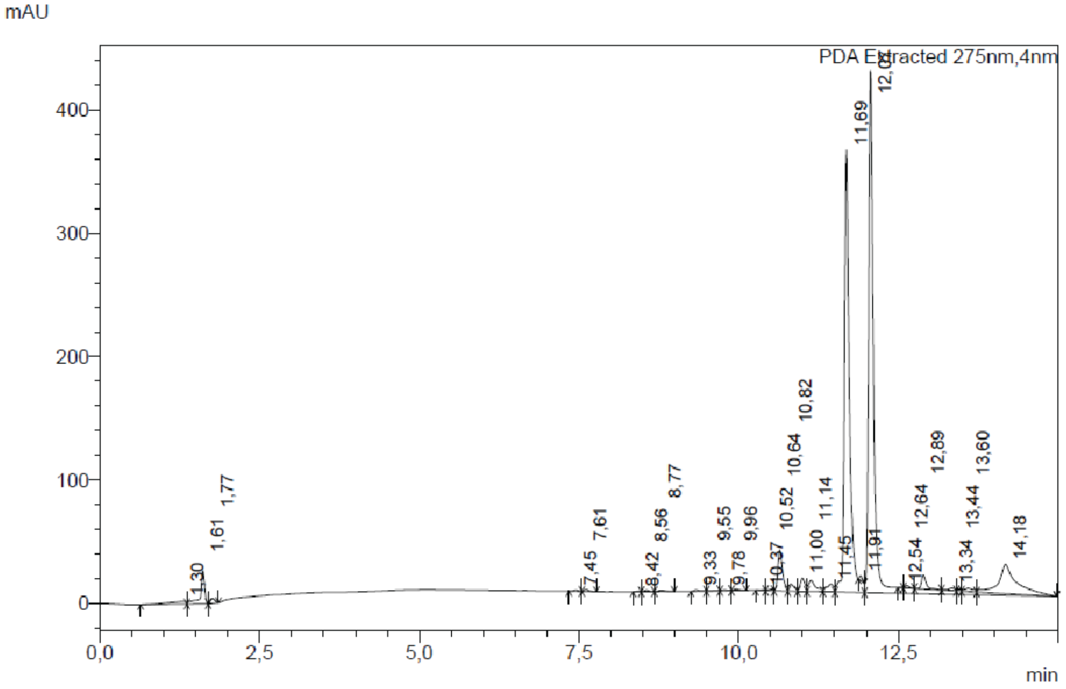

mAU
PDA Extracted 275nm,4nm
min

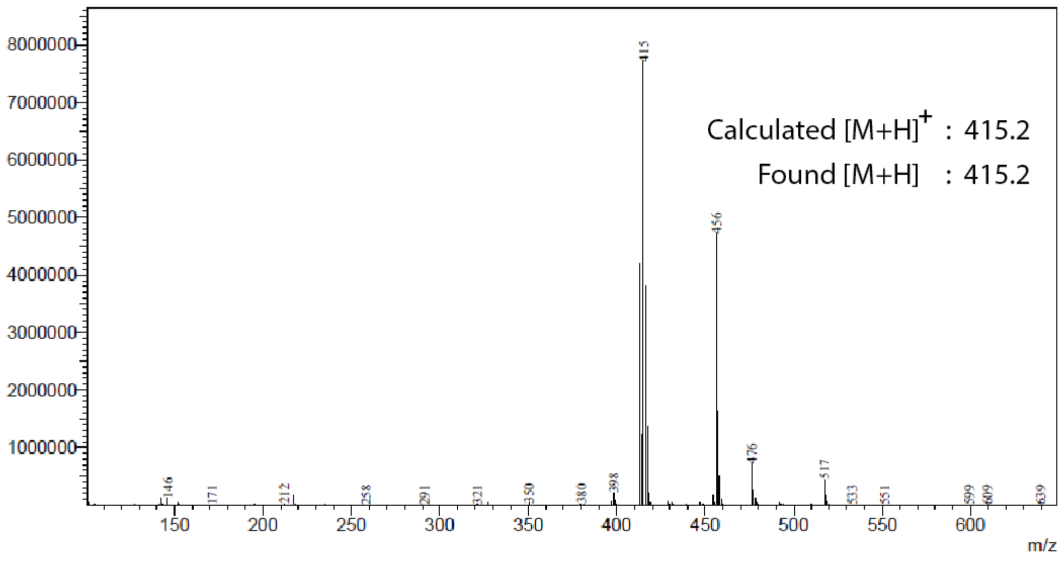

Calculated [M+H]+ : 415.2
Found [M+H] : 415.2
m/z

Kinetic measurements

Kinetic measurements were performed under pseudo first-order conditions using an excess of the corresponding axial TCO isomer **1** or the equatorial isomer **2** respectively. Second order rate constants were determined by following the decay in the concentration of the starting 1,2,4,5-tetrazine over time (see table S3 for the wavelength used for each tetrazine). The concentration decrease was monitored by UV/VIS spectrometry (performed on Agilent Cary 60). The measurements were performed in a mixture of CH_3CN/H_2O (4:1) at room temperature.

Conditions: 25 µM solution of the respective tetrazine in CH_3CN was added to a 250 µM solution of the TCO in CH_3CN/H_2O. The final tetrazine concentration was 12,5 µM and the final concentration of TCO 125 µM in CH_3CN/H_2O (4:1). The measurement was immediately started after addition of the tetrazine and brief mixing of the reaction mixture in the UV cuvette. The observed decrease of the tetrazine concentration was plotted against time and the data fitted with single exponential equation ($y = y_0 + Ae^{-k/t}$) using OriginPro software to provide the observed rate constants *k'*. The second order rate constants *k* were calculated by dividing the observed rate constants with the initial concentration of the TCO. All runs were conducted at least three times and the results are summarized in Table S3.

Table S3. The second-order rate constants *k* in M^{-1} s^{-1} of the reaction between 1,2,4,5-tetrazines and TCO isomers **1** and **2**.

Tetrazine	Abs. (nm)[a]	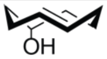	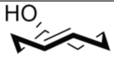
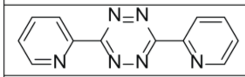	305	601 ± 57	235 ± 8
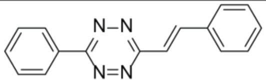	330	28 ± 7	11 ± 1
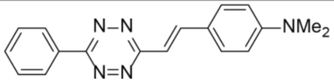	425	20 ± 3	14 ± 2
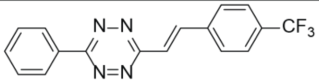	320	44 ± 5	19 ± 3
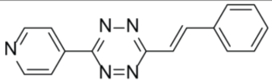	335	90 ± 14	43 ± 1
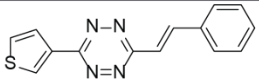	332	25 ± 1	8 ± 1
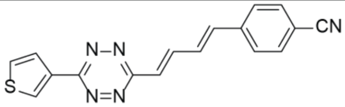	360	21 ± 5	12 ± 2
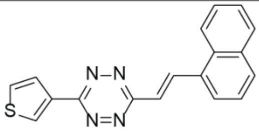	360	26 ± 1	12 ± 1

a) This wavelength was used to follow the decay in the concentration of starting tetrazine.

We next performed a kinetic scanning experiment to follow the progress of the reaction by changes in the absorption spectra over time using tetrazine **3l** as an example (Figure S17). This experiment was performed under conditions identical to the ones used for the above kinetic experiments. Note that the two TCO isomers lead under identical conditions to different spectra profiles indicating a different mechanism

of the reaction by using different TCO isomer. This is in agreement with the data observed during NMR studies of the reaction mechanism.

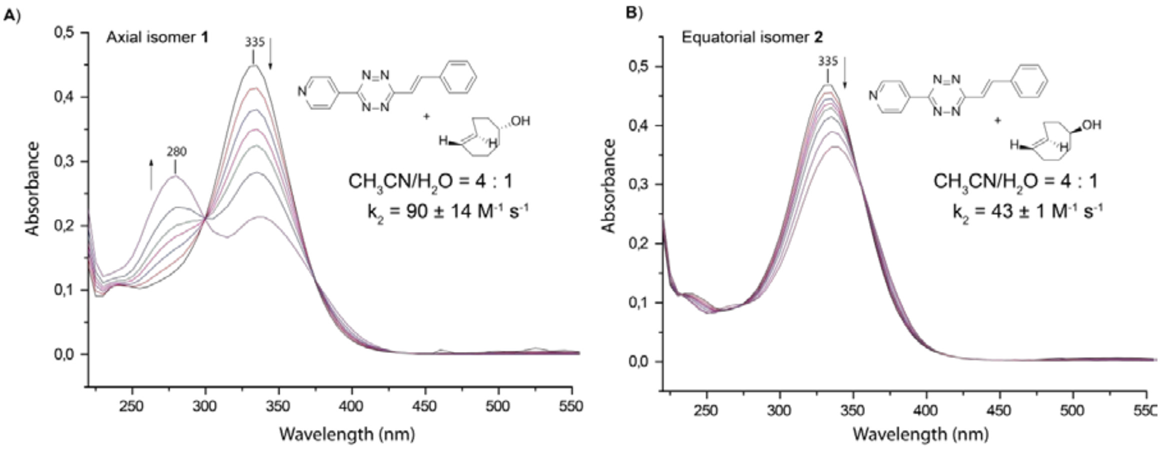

Figure S17. Scanning kinetic experiment showing the progress of the reaction of **3l** and TCO **1** and **2.**

Due to limited solubility of most of the tetrazines in water we were unable to perform the kinetic measurements in the presence of more water. Because it is known that the inverse electron-demand Diels-Alder reaction is accelerated in water we performed further experiments using tetrazine **3l** which is sufficiently water soluble (Figure S18). This experiments were performed using a manual stopped flow device connected to the UV spectrophotometer using 12,5 µM final tetrazine concentration and 125 µM final TCO concentration. As evident from this data the reaction rates increase substantially by increased water content what is in good agreement with the excellent reactivity observed during cell labeling experiments (Figure S26 and S27).

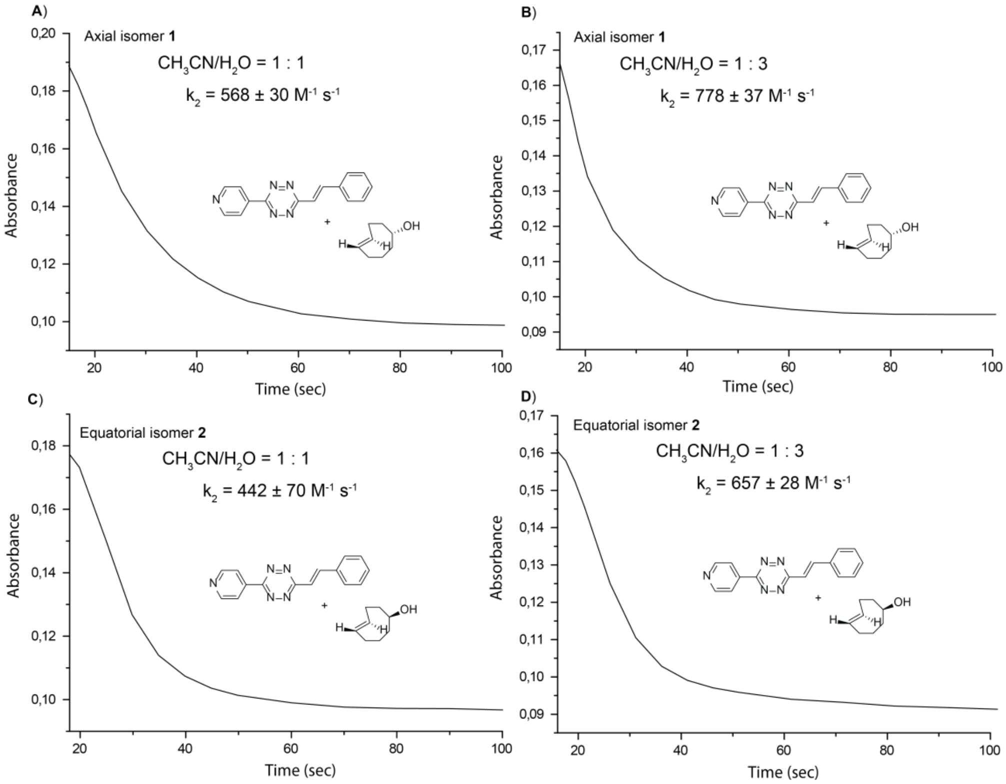

Figure S18. Kinetic data of the reaction of 3l with TCO **1** and **2** in aqueous solvents system.

Stability studies of the click products

Stability studies in CH_3CN/PBS buffer:

The stability of the click products with TCO **1** was studied for (*E*)-3-phenyl-6-styryl-1,2,4,5-tetrazine[6] as well as for the tetrazines **3C** and **3G**. The stability studies were performed in CH_3CN/PBS (1:1) at 37 °C and were monitored by HPLC-MS on a Luna® C18 column (3u, 100A, 100 x 4.6 mm) using a linear gradient of CH_3CN + 0.05% HCOOH (5→95% in 9 min) in H_2O + 0.05% HCOOH at a flow rate of 1.0 mL/min.

Conditions: 0.3 mL of a 1.25 mM solution of the tetrazine in CH_3CN were added to 75 µL of a 10 mM solution of TCO **1** in PBS. The mixture was diluted with PBS to a final volume of 0.6 mL in order to get 0.625 mM final concentration of the corresponding tetrazine using 2 eq of TCO **1**. The solutions were shaken at room temperature for 1 h and measured by HPLC-MS to verify the formation of the click products (indicated time 0 min in Figure S19-S21). For stability study, the click products were incubated at 37 °C for 168 h in total. During that time the reaction mixtures were measured several times by HPLC-MS (Figure S19-S21).

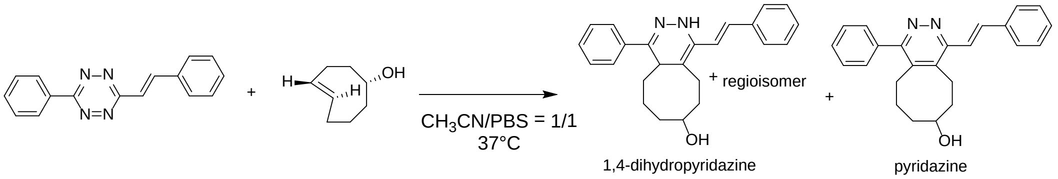

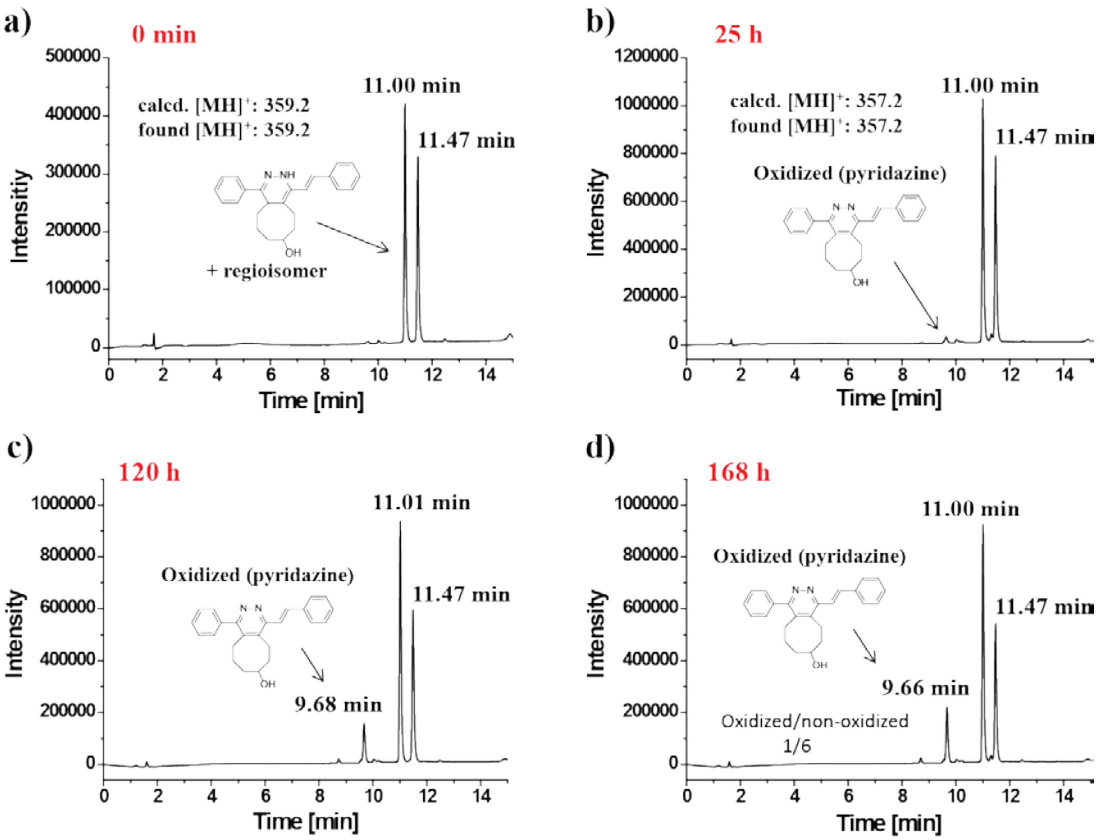

Figure S19. HPLC-MS analysis of the click product between (*E*)-3-phenyl-6-styryl-1,2,4,5-tetrazine and TCO **1** in CH_3CN/PBS (1:1) at 37 °C after a) 0 min, b) 25 h, c) 120 h and d) 168 h. Ratio of the dihydropyridazine/pyridazine products after 168h = 6/1.

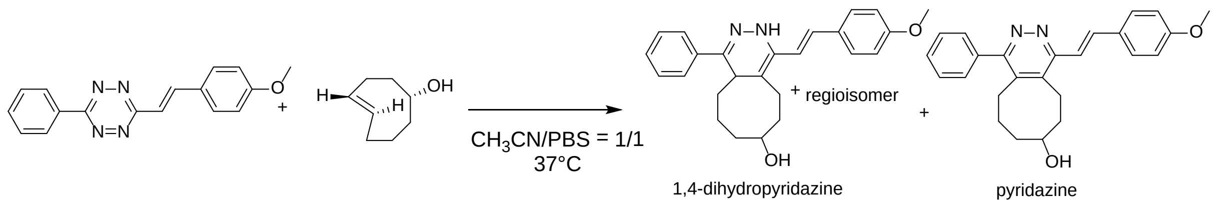

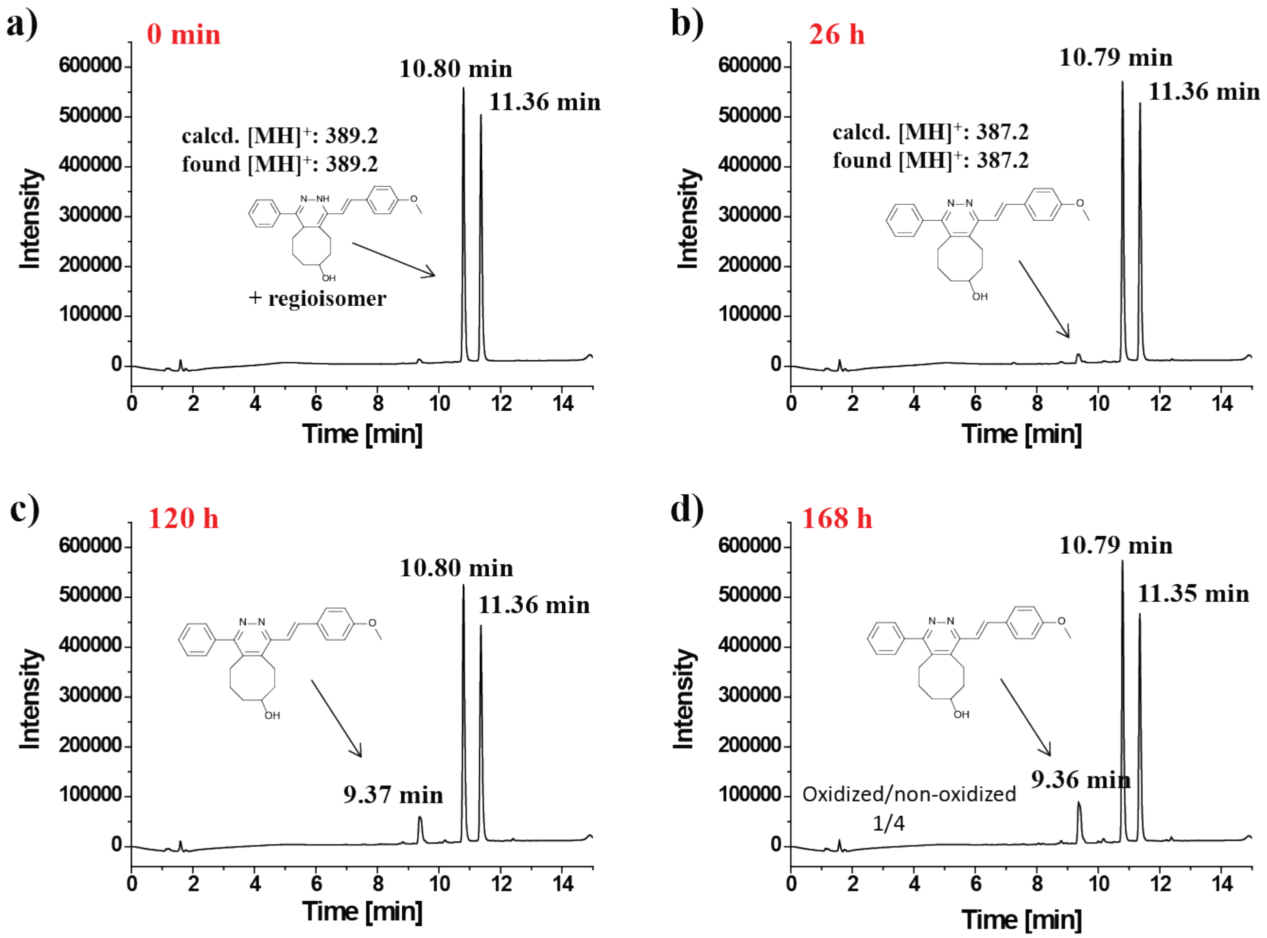

Figure S20. HPLC-MS analysis of the click product between tetrazine **3C** and TCO **1** in CH_3CN/PBS (1:1) at 37 °C after a) 0 min, b) 26 h, c) 120 h and d) 168 h. Ratio of the dihydropyridazine/pyridazine products after 168h = 4/1.

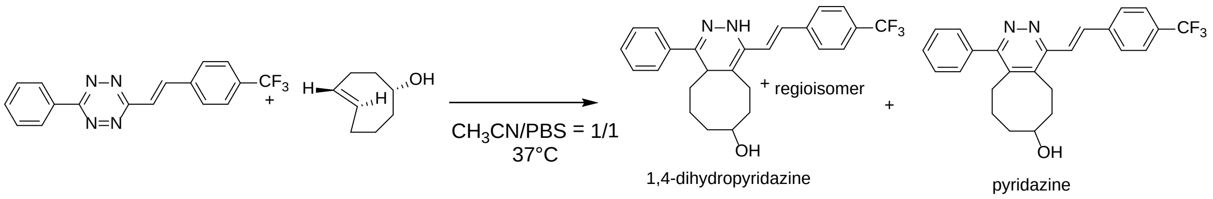

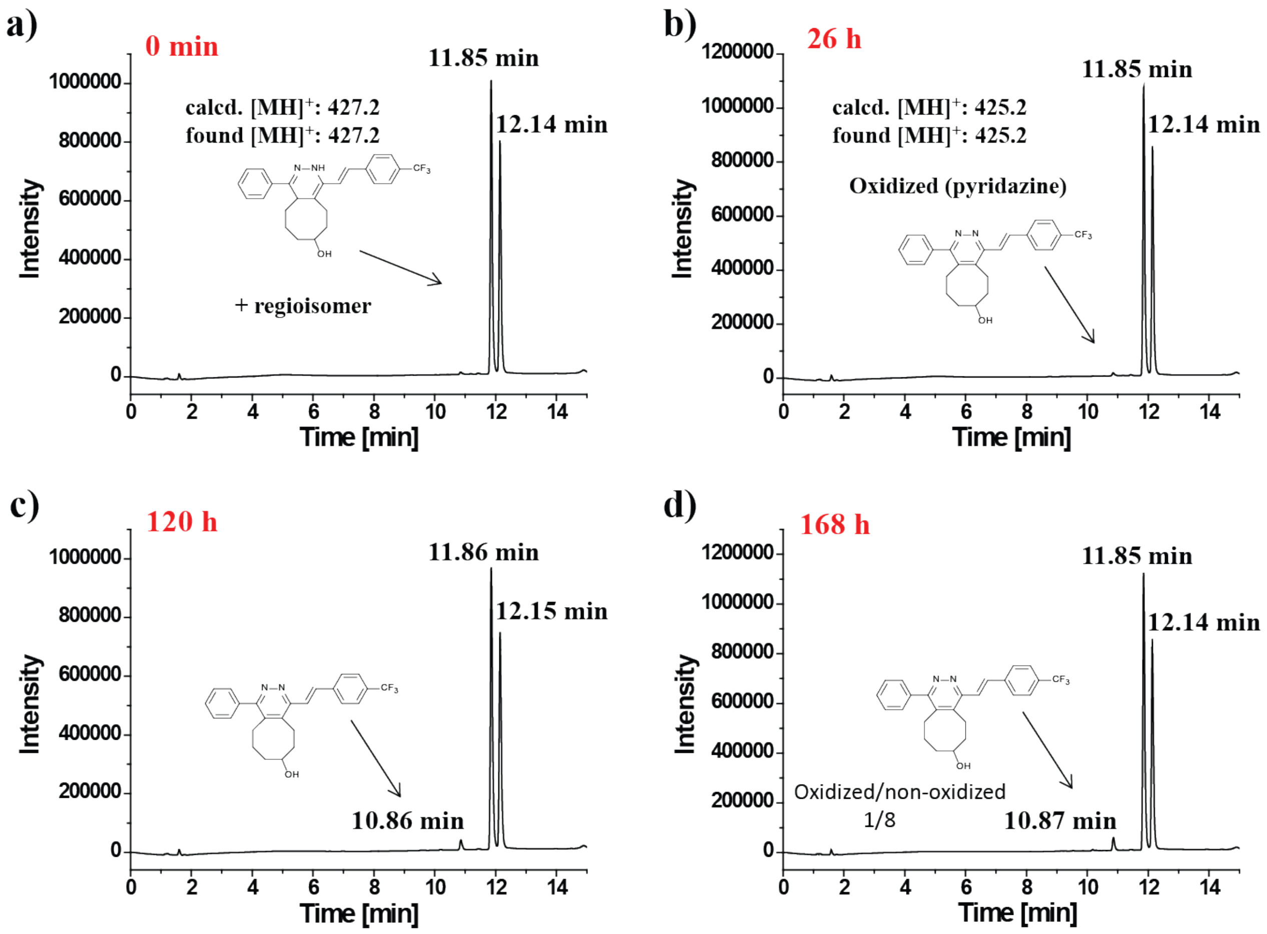

Figure S21. HPLC-MS analysis of the click product between tetrazine **3G** and TCO **1** in CH_3CN/PBS (1:1) at 37 °C after a) 0 min, b) 26 h, c) 120 h and d) 168 h. Ratio of the dihydropyridazine/pyridazine products after 168h = 8/1.

Stability studies in fetal bovine serum:

A 20 µL of (*E*)-3-phenyl-6-styryl-1,2,4,5-tetrazine solution in DMSO (50 mM) was added to 980 µL of serum (Fetal bovine serum from Biosera, 1x diluted with MiliQ water) containing the axial TCO isomer (20 µL of 100 mM in DMSO, corresponding to 2 equivalents). The reaction mixture in serum was incubated at 37 °C and was analyzed by HPLC-MS over time. A sample of the crude reaction mixture in serum was filtered through 0.2 µm syringe filter and was directly used for HPLC-MS. Another part of the sample was

mixed with 200 µL of DCM, rigorously vortexed and centrifuged. The upper (aqueous) part was removed with a pipette and the organic phase concentrated using Speedvac. After re-dilution in 100 µL of CH_3CN/H_2O (1/1) and centrifugation 50 µL of the sample were placed into HPLC vial and were analyzed by HPLC-MS (Figure S22).

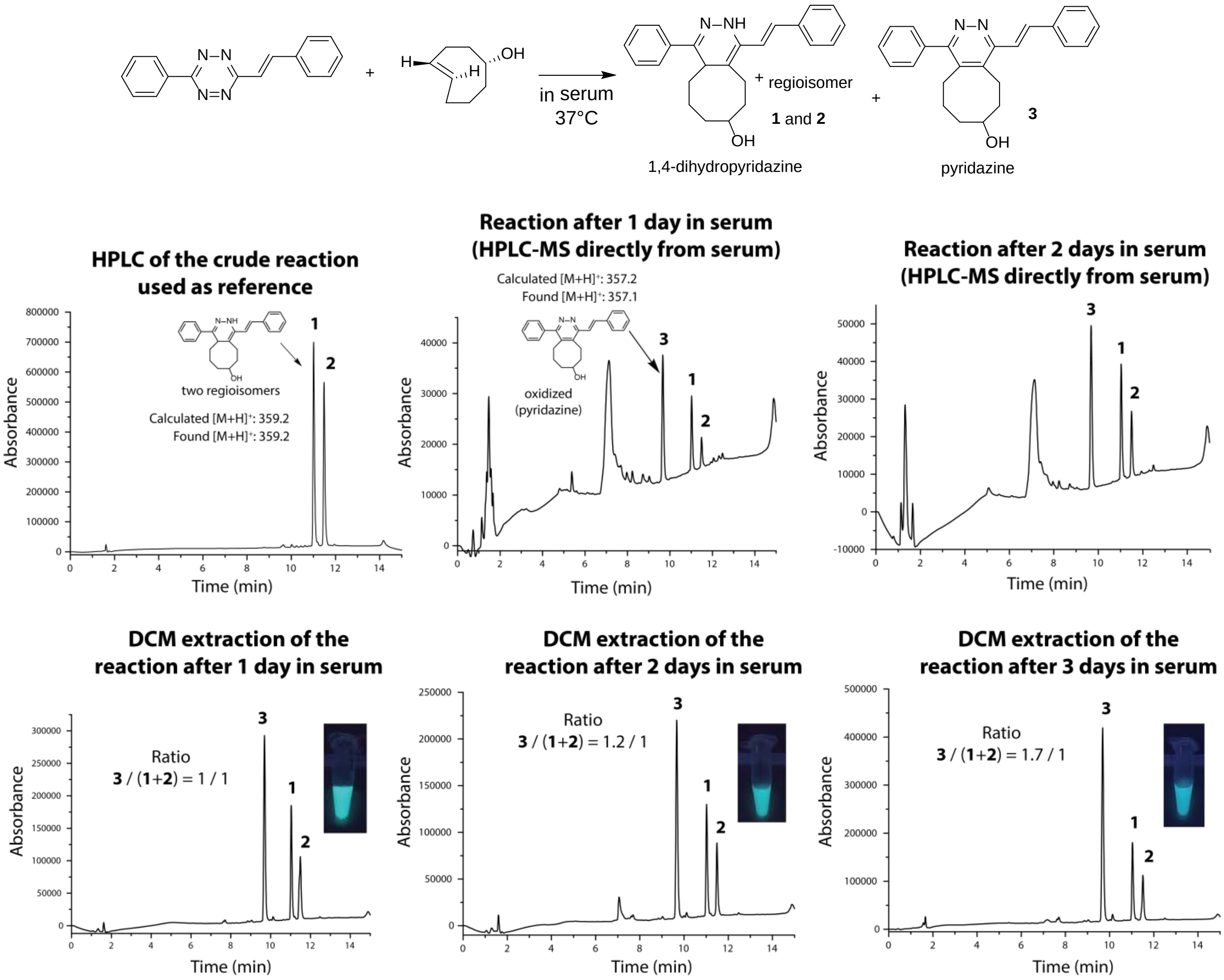

Figure S22. Stability study of the click product in FBS as analyzed by HPLC-MS over time.

Comment: after first day of incubation under the above conditions we observed a formation of a new product that was assigned (according to the observed mass) to the corresponding oxidation product (pyridazine labeled as **3** in Figure S22) giving a ratio between the pyridazine **3** and the 1,4-dihydropyridazines **1** and **2** = ca. 1 / 1 (according to integration of the peak areas). After the second day the ratio increased to: **3** / (**1** + **2**) = 1.2 / 1 and after third day further to: **3** / (**1** + **2**) = 1.7 / 1.

Cell labeling experiments

U2OS cells were maintained in high glucose DMEM (Sigma) supplemented with 10% FBS (Biosera) and 0.1mg/ml of penicillin-streptomycin (Sigma) at 37°C/5% CO_2. One day before the experiment $0.3x10^6$ cells were seeded at the 3.5 cm cultivation dishes with a coverglass in the bottom (SPL Life Sciences).

Tetrazine conjugated materials (TPP-Tet or Taxol-Tet) were dissolved in DMSO (5mM). Cells were incubated with the tetrazine conjugated compounds in complete media for indicated time points at 37°C. Cells were then washed once with media, and incubated for further 10-30 minutes in complete DMEM medium without phenol red containing DRAQ 5, Mitotracker deep red or Tubulin tracker (all from Thermo Scientific). In experiments with TPP-Tet and Taxol-Tet probes the cells were incubated prior to imaging for 10 minutes at 37°C with 25 or 50μM (5 or 10 equivalents, final concentration) of TCO **1** (stock 50 mM in DMSO). For real-time click experiment (Figure S26 and S27), TCO **1** was added to a dish mounted onto the microscope. Pictures of live cells were taken every minute in total time of 20 minutes. Images of live cells were taken using Leica TCS SP5 confocal microscope equipped with HC PL APO CS2 63.0x1.40 OIL UV objective. Excitation for click products was 405 nm. Emission was collected sequentially using Hyd detector in BrightR mode, with AOBS window set to 412-522 nm or 450-550 nm. DRAQ5 for nuclei staining was excited with 633 nm laser and collected in a window 667-748 nm. Mitotracker: excitation 633 nm, emission window 692-734 nm. Tubulin tracker: excitation 561 nm, emission window 600-650 nm. For details of each experiment see the figure captions. Brightness of the raw images was adjusted using FIJI software.[12]

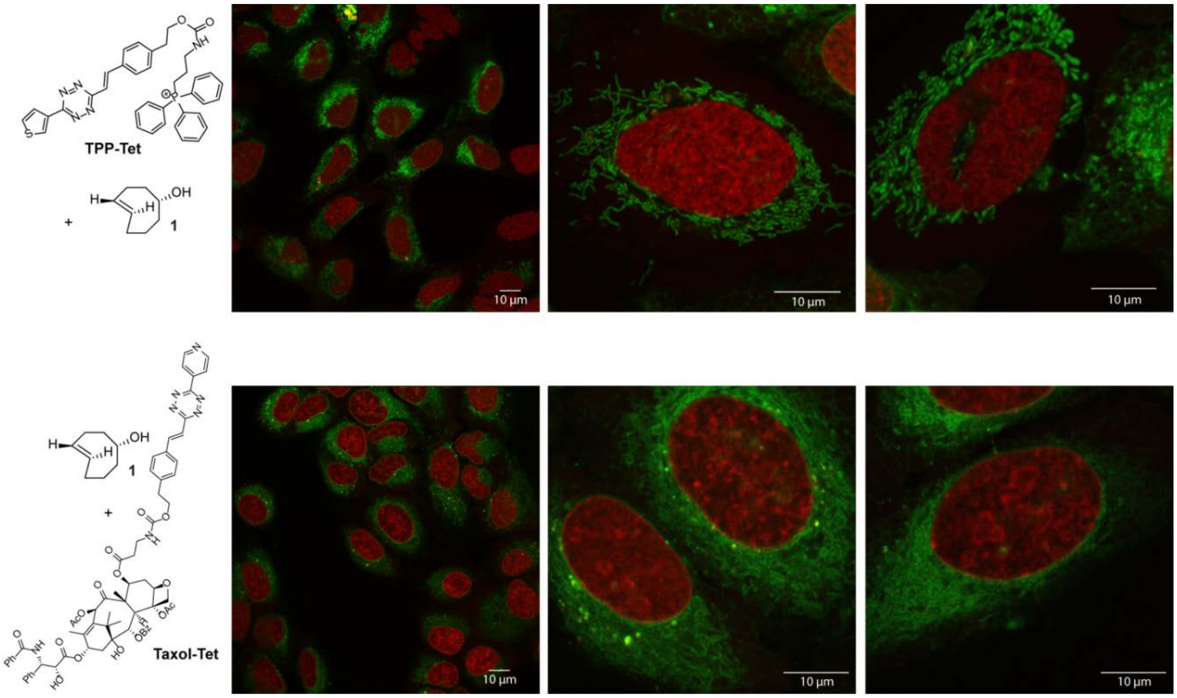

Figure S23. U2OS cell labelling experiments using mitochondria selective TPP-Tet probe and microtubule selective Taxol-Tet probe. Conditions: incubation of cells for 3 hours/37°C with the respective tetrazine probe (5 μM), washing and incubation with DMEM medium containing 500 nM DRAQ5 nuclear dye for 30 min and finally addition of TCO **1** (25 μM final). The images were acquired on confocal microscope using 405 nm excitation for click products (emission window 412-522 nm) and 633 nm laser excitation for DRAQ5 and a 667-748 nm emission window. Note: The signal intensity in case of the Taxol-Tet probe was lower than for the TPP-Tet probe and the laser intensity was therefore increased from 10 to 15%.

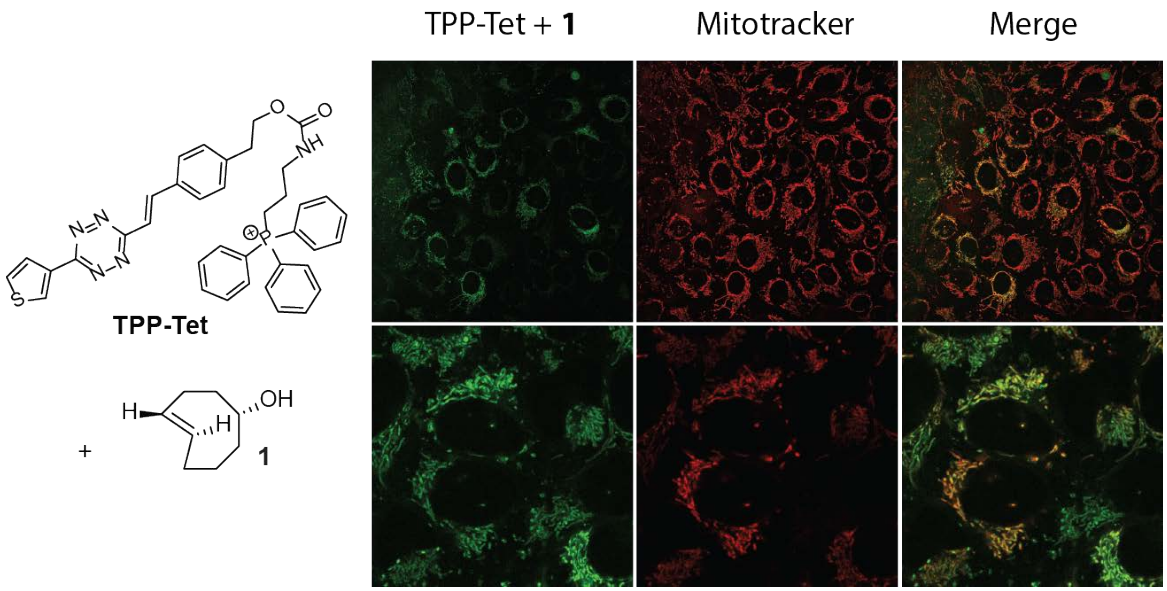

Figure S24. U2OS cell labeling experiments using mitochondria selective TPP-Tet probe. Conditions: incubation of cells for 0,5 hours/37°C with TPP-Tet probe (2 µM), washing and incubation with DMEM medium containing Mitotracker deep red (10 nM) for 10 min and finally addition of TCO **1** (50 µM final). The images were acquired on confocal microscope using 405 nm excitation for click products (laser intensity 10%, emission window 450-550 nm) and 633 nm laser excitation for Mitotracker deep red (laser intensity 19%, 692-734 nm emission window). Note: we observed heterogeneity in uptake of the TPP-Tet probe by U2OS cells. However, this has not been observed in HeLa cells (data not shown).

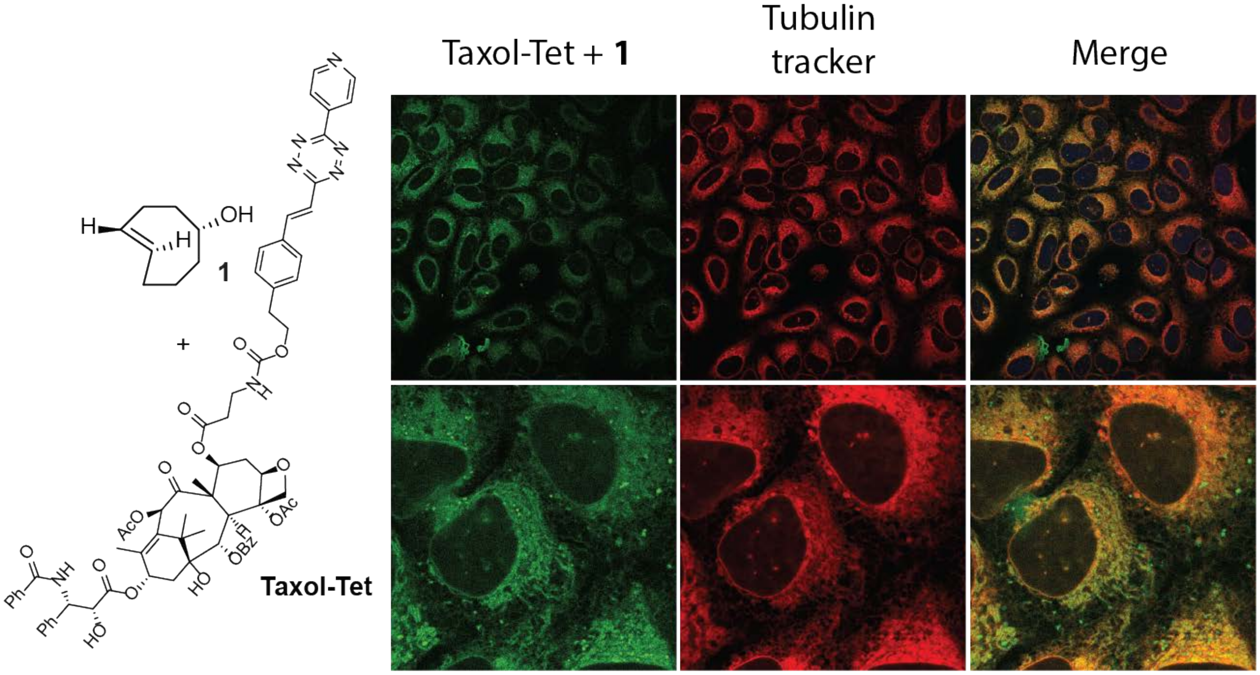

Figure S25. U2OS cell labelling experiments using microtubule selective Taxol-Tet probe. Conditions: incubation of cells for 0,5 hours/37°C with the respective Taxol-Tet probe (5 µM), washing and incubation with DMEM medium containing Tubulin tracker (BODIPY® 564/570 Taxol from Thermo Scientific) (0,1 µM final) for 10 min and finally addition of TCO **1** (50 µM final). The images were acquired on confocal microscope using 405 nm excitation for click products (laser intensity 10%, emission window 450-550 nm) and 561 nm laser excitation for Tubulin tracker (laser intensity 15%, 600-650 nm emission window).

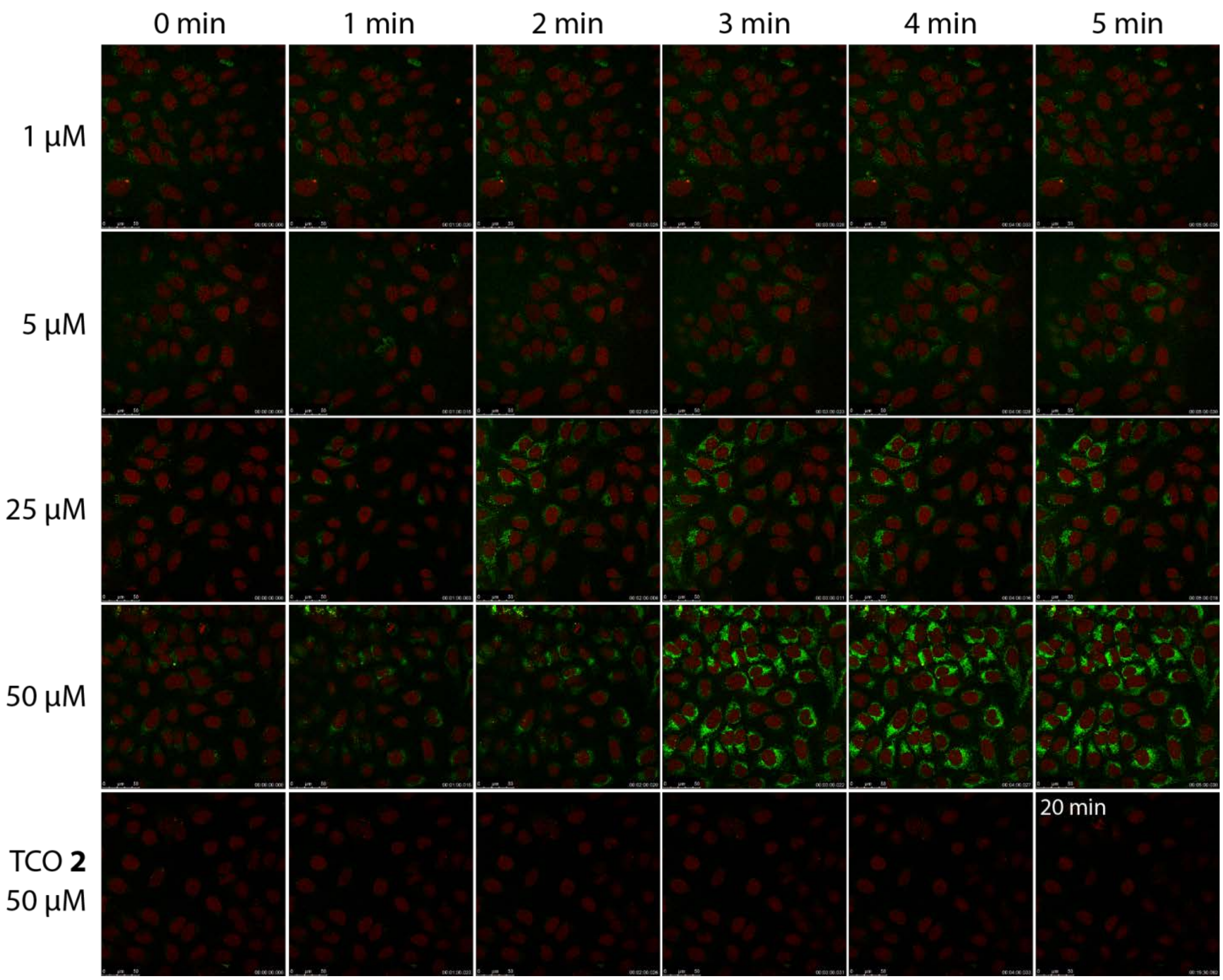

Figure S26. Time-lapse of the click reaction in live U2OS cells containing TPP-Tet probe (2 µM final, 0,5 h incubation) using different TCO **1** concentrations (1, 5, 25 or 50 µM) or TCO **2** (50 µM). The images were captured at indicated time points after 1h incubation with TPP-Tet probe. Note that in the case of TCO **2** no fluorescence formed in the cells even after 20 min.

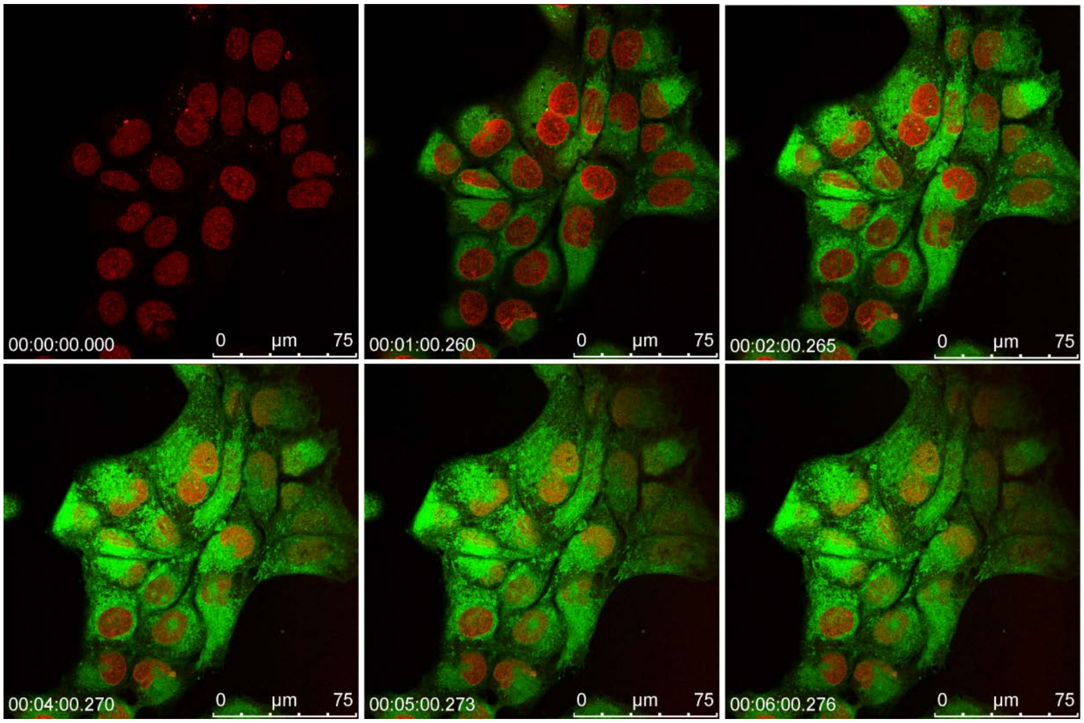

Figure S27. Time lapse of the click reaction in live U2OS cells containing TPP-Tet probe (10 µM). The images were captured at indicated time points after addition of 25 µM axial TCO **1**. Notice that conspicuous fluorescent signal appears already within 1 min after TCO addition. The fluorescence reaches its maximum intensity in only 2-3 min. Please note that microscope stage has moved slightly in Z-direction after 2-3rd minute.

NMR experiments and computational study

For the NMR monitoring of TCO reaction with diphenyltetrazine, the studied cyclooctenol (ca 5 mg) was dissolved in CD_3CN (1 mL) and D_2O (150 μL) solvent mixture. A suspension of diphenyltetrazine in CD_3CN was added to the solution under vigorous shaking until the violet color of diphenyltetrazine stopped disappearing. 500 μL of the reaction mixture was transferred to an NMR tube and NMR experiments were acquired periodically and the reaction progress was monitored. A combination of 1D (^1H and ^{13}C) experiments with 2D correlation experiments (H,H-COSY, H,C-HSQC, H,C-HMBC and ROESY) was used to determine the structure and conformation of the reactants, intermediates and final products.

The search for preferred conformations of the studied compounds was performed using one hundred simulated annealings to 1000 K followed by slow cooling to 200 K performed with every molecule. Molecular modeling program package Hyperchem 8 (Hypercube) was used. The conjugate gradient method for energy minimizations was used to convergence (less than 0.01 kJ mol^{-1} RMS force). Force field MM+ was used for all computations. General protocol for obtaining lowest-energy conformers by simulated annealing: optimized starting structure was subjected to dynamic run - 0.5 ps heating from 300 to 1000 °C, 0.7 ps equilibration and 1 ps cooling to 200 °C followed by energy minimization. Every next run started from the previously minimized structure. A set of 100 structures was so obtained for each compound and the calculated structures were then sorted according to the conformation to several structural types.

The lowest-energy conformations and other conformations important for the proposed reactions were subjected to geometry optimization at DFT level, using B3LYP functional,[13] standard 6-31+G(d,p) basis set and polarizable continuum model used for implicit acetonitrile solvation.[14] The Gaussian09 program package was used throughout this study.[15] The QST3 optimization method[16] was applied in the search for the transition state structures of the reaction, that is, the structures of the reactant, product, and estimated transition state were used as input for the TS search. The vibrational frequencies and free energies were calculated for all of the optimized structures, and the stationary-point character (a minimum or a first-order saddle point) was thus confirmed.

Comment

The reaction of TCOs 1 and 2 with diphenyltetrazine

The reaction of TCO **2** with diphenyltetrazine leads to a mixture of intermediates and products. The progress of the reaction was followed by NMR spectroscopy and all the components of the mixture were identified. An overview of the reaction is depicted in Scheme S1, illustrative examples of proton NMR spectra monitoring the reaction progress are shown in Figure S28 and the changes of relative concentrations of the mixture components during the hydration-dehydration reaction are shown in Figure S29. The configuration at the newly arisen asymmetric center (HN–C–OH) in the intermediates **2B** was not determined experimentally, but the conformational analysis predicts that the structures with the hydroxy group in pseudoaxial position (i.e. *trans* to the nearest bridgehead hydrogen atom) are by 1.5–2 kcal/mol more stable.

No signals of OH/NH hydrogens were observed during NMR measurements because the reaction was performed in the presence of D_2O. In agreement with the proposed mechanism, no attachment of deuterium to a carbon atom was observed.

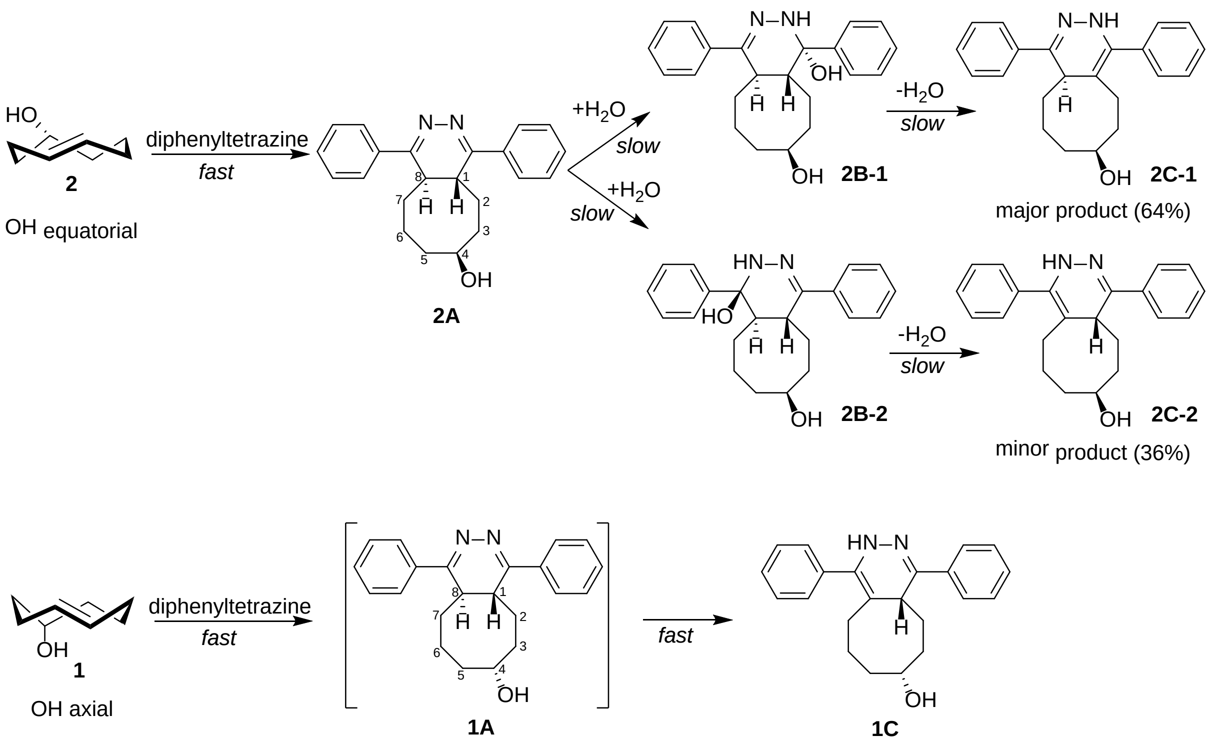

Scheme S1. The reaction of compounds **1** and **2** with diphenyltetrazine.

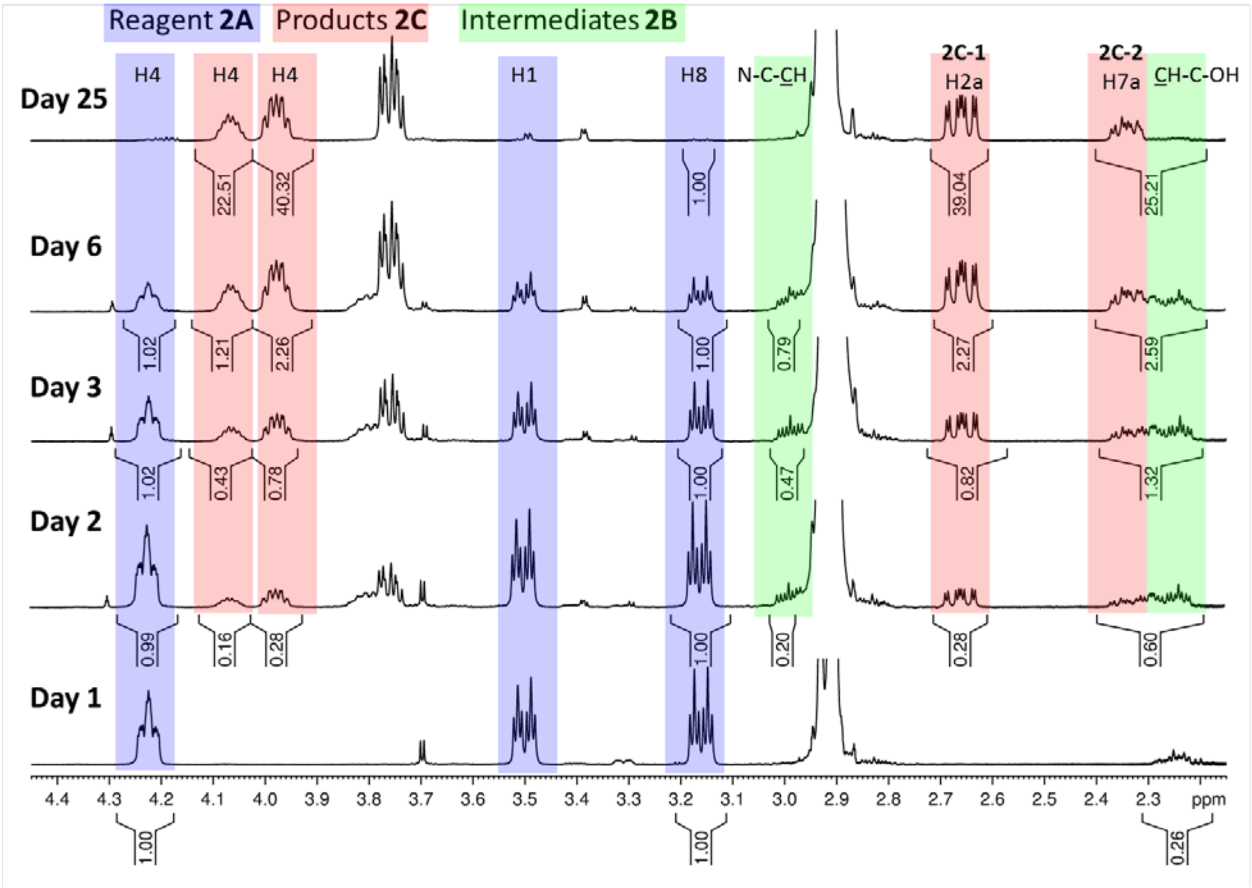

Figure S28. The ^{1}H NMR observation of the progress of the hydration/dehydration reaction of the diphenyltetrazine adduct to equatorial TCO **2**.

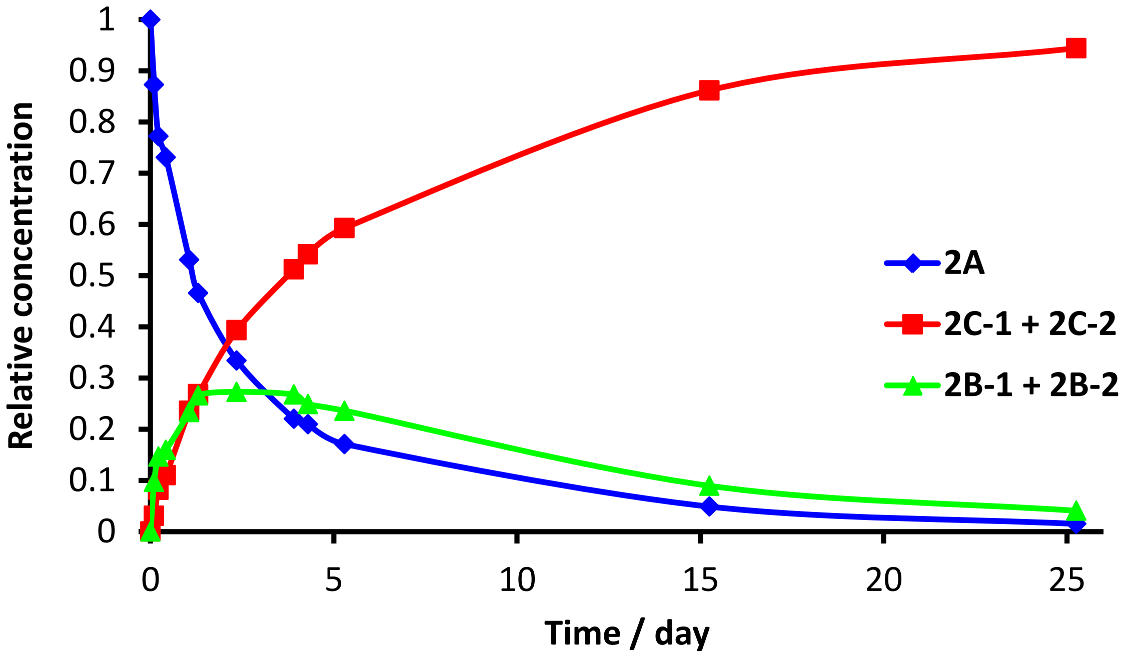

Figure S29. The progress of the hydration/dehydration reaction of the diphenyltetrazine adduct to equatorial TCO **2**.

The following compounds were identified in the reaction mixture during the hydration-dehydration reaction following the reaction of compound **2** with diphenyltetrazine:

2A

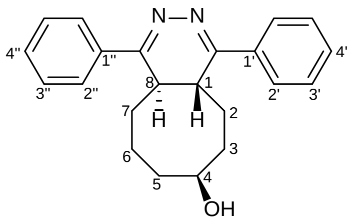

^{1}H NMR (CD_3CN + D_2O, 500.0 MHz): δ = 1.36–1.46 (m, 2H, H-2b and H-7b), 1.56 (m, 1H, H-6b), 1.70–1.78 (m, 2H, H-5b and H-7a), 1.87–2.02 (m, 4H, H-2a, H-5a and H-3), 2.06 (m, 1H, H-6a), 3.16 (dt, 1H, $J_{8\text{-}7b}$ = 12.9, $J_{8\text{-}7a}$ = $J_{8\text{-}1}$ = 4.2, H-8), 3.50 (dt, 1H, $J_{1\text{-}2b}$ = 12.6, $J_{1\text{-}2a}$ = $J_{1\text{-}8}$ = 3.8, H-1), 4.22 (dddd, 1H, *J* = 8.1, 7.4, 3.2 and 1.7, H-4), 7.45–7.51 (m, 6H, H-3', H-3'', H-4' and H-4''), 7.85–7.88 (m, 2H, H-2''), 7.95–7.98 (m, 2H, H-2').
^{13}C NMR (CD_3CN + D_2O, 125.7 MHz): δ = 20.28 (C-6), 29.04 (C-2), 31.49 (C-1), 32.15 (C-7), 32.42 (C-8), 32.70 (C-3), 35.80 (C-5), 69.52 (C-4), 128.45 and 128.50 (C-2' and C-2''), 129.60 and 129.65 (C-3' and C-3''), 131.43 and 131.50 (C-4' and C-4''), 137.23 and 137.35 (C-1' and C-1''), 162.63 and 162.74 (1-C-N and 8-C-N).

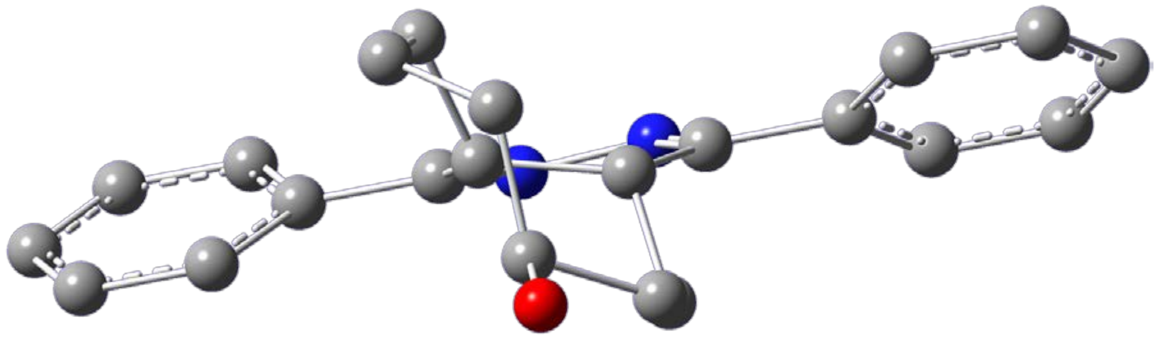

Figure S30. The lowest-energy conformation of compound **2A** found by molecular modeling (hydrogen atoms omitted for clarity). This conformation was confirmed experimentally by NMR spectroscopy. For example, ROESY experiment confirmed the spatial proximity of H-4 and H-8.

2B-1 and **2B-2**

These intermediates were present in the reaction mixture in ca 1:1 ratio, they were not isolated and ^{13}C NMR spectra were only partly assigned and only few characteristic ^{1}H NMR signals are presented here, other signals were overlapped by the signals of compounds **2A**, **2C-1** and **2C-2**.

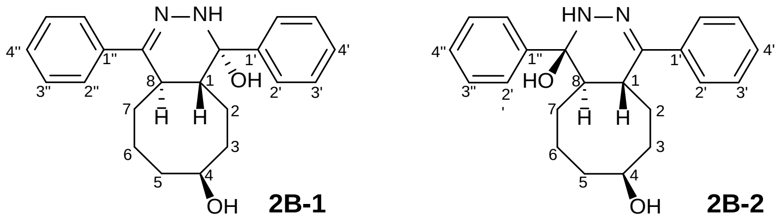

2B-1 **2B-2**

^{13}C NMR (CD_3CN + D_2O, 125.7 MHz): δ = 22.10, 22.57, 26.60, 30.75, 30.78, 33.93, 34.05, 35.25, 35.84, 36.23, 37.90 and 38.41 (N-C-<u>C</u>H), 49.00 and 49.89 (<u>C</u>H-C-OH), 71.60 and 72.14 (C-4), 84.81 and 84.94 (NH-<u>C</u>-OH), 126.88, 127.81, 127.86, 128.81, 128.84, 128.88, 128.89, 128.90, 129.30, 138.89 and 138.92 (N-C-<u>C</u>), 144.45 and 144.50 (NH-C(OH)-<u>C</u>), 155.18 and 155.21 (N-<u>C</u>).

^{1}H NMR (CD_3CN + D_2O, 500.0 MHz): δ = 2.24, 2.30, 2.92 and 2.99 (4 x m, 4H, H-1 and H-8), 3.80 (m, 2H, H-4).

2C-1

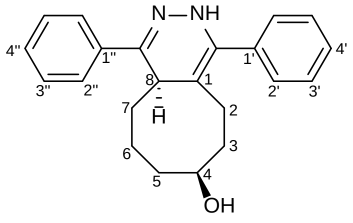

^{1}H NMR (CD_3CN + D_2O, 500.0 MHz): δ = 1.36–1.44 (m, 2H, H-6b and H-7b), 1.46–1.66 (m, 1H, H-7a), 1.70–1.80 (m, 3H, H-3b, H-5b and H-6a), 1.84–1.98 (m, 2H, H-2b and H-5a), 2.10 (m, 1H, H-3a), 2.66 (ddd, 1H, J_{gem} = 15.4, $J_{2a\text{-}3}$ = 10.4 and 2.9, H-2a), 3.76 (dd, 1H, $J_{8\text{-}7}$ = 11.7 and 4.1, H-8), 3.98 (m, 1H, H-4), 7.32–7.50 (m, 8H, H-2', H-3', H-4', H-3'' and H-4''), 7.79–7.83 (m, 2H, H-2'').

^{13}C NMR (CD_3CN + D_2O, 125.7 MHz): δ = 22.01 (C-6), 26.33 (C-2), 29.69 (C-7), 33.54 (C-3), 35.90 (C-5), 37.09 (C-8), 72.06 (C-4), 107.18 (C-1), 126.85 (C-2''), 129.15 and 129.17 (C-4' and C-4''), 129.49 (C-3''), 129.51 and 130.03 (C-2' and C-3'), 135.71 (1-<u>C</u>-NH), 136.34 and 137.36 (C-1' and C-1''), 141.99 (8-<u>C</u>-N).

2C-2

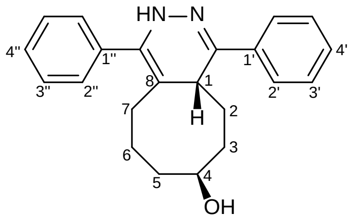

^{1}H NMR (CD_3CN + D_2O, 500.0 MHz): δ = 1.46–1.66 (m, 4H, H-2, H-5b and H-6b), 1.70–1.80 (m, 2H, H-3), 1.84–1.98 (m, 3H, H-5a, H-6a and H-7b), 2.34 (m, 1H, H-7a), 3.75 (dd, 1H, $J_{1\text{-}2}$ = 11.4 and 4.8, H-1), 4.07 (m, 1H, H-4), 7.32–7.50 (m, 8H, H-3', H-4', H-2'', H-3'' and H-4''), 7.79–7.83 (m, 2H, H-2').
^{13}C NMR (CD_3CN + D_2O, 125.7 MHz): δ = 24.33 (C-6), 25.94 (C-2), 31.30 (C-7), 33.44 (C-3), 37.11 (C-5), 37.15 (C-1), 71.50 (C-4), 106.80 (C-8), 126.87 (C-2'), 129.19 and 129.30 (C-4' and C-4''), 129.51 (C-2'' or C-3''), 129.60 (C-3'), 130.02 (C-2'' or C-3''), 136.22 (8-C-NH), 136.25 and 137.33 (C-1' and C-1''), 142.42 (1-C-N).

The reaction of TCO **1** with diphenyltetrazine leads to a single product **1C**:

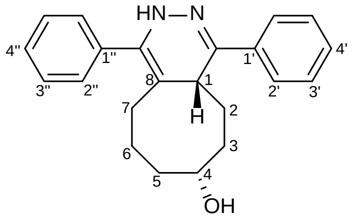

^{1}H NMR (CD_3CN + D_2O, 500.0 MHz): δ = 1.41 (dddd, 1H, J_{gem} = 13.7, $J_{2b\text{-}1}$ = 10.8, $J_{2b\text{-}3}$ = 6.0 and 2.7, H-2b), 1.52 (m, 1H, H-3b), 1.56–1.67 (m, 3H, H-2a, H-6b, H-7b), 1.75 (m, 1H, H-6a), 1.87 (m, 1H, H-3a), 1.90–1.94 (m, 2H, H-5), 2.42 (m, 1H, H-7a), 3.75 (dd, 1H, $J_{1\text{-}2}$ = 10.7 and 6.7, H-1), 3.82 (dq, 1H, $J_{4\text{-}3a}$ = 10.6, $J_{4\text{-}3b}$ = $J_{4\text{-}5}$ = 3.8), 7.33 (m, 1H, H-4'), 7.37–7.41 (m, 3H, H-3', H-4''), 7.42–7.47 (m, 4H, H-2'', H-3''), 7.80 (m, 2H, H-2').
^{13}C NMR (CD_3CN + D_2O, 125.7 MHz): δ = 22.67 (C-6), 25.87 (C-2), 30.14 (C-3), 33.07 (C-7), 34.95 (C-5), 36.86 (C-1), 71.53 (C-4), 105.89 (C-8), 126.85 (C-2'), 129.19 (C-4'), 129.47 (C-4''), 129.50 (C-3'), 129.57 and 130.09 (C-2'' and C-3''), 136.14 (C-1''), 136.98 (8-C-NH), 137.51 (C-1'), 142.91 (1-C-N).

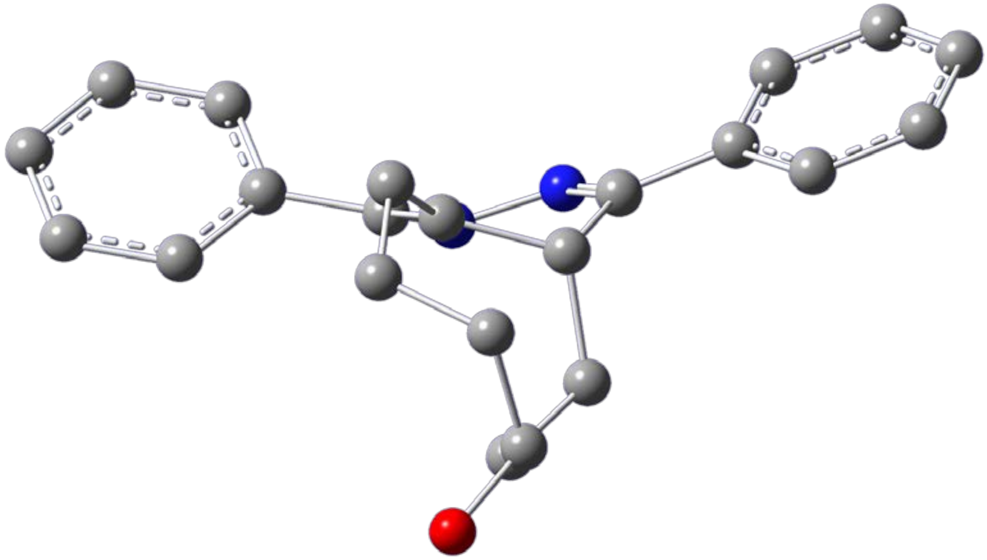

Figure S31. The lowest-energy conformation of compound **1C** found by molecular modeling (hydrogen atoms omitted for clarity). This conformation of the product was confirmed experimentally by NMR spectroscopy. For example, ROESY experiment confirmed the spatial proximity of H-1 and one of the hydrogen atoms at C-5, or large coupling constant between H-4 and one of H-3 confirmed that the torsion angle between these hydrogens is close to 180°.

Conformational analysis

Conformational analysis of compounds 1A, 1B, 2A and 2B

The conformational analysis of intermediate **1A** performed with molecular-mechanics simulated annealing shows that the hydroxyl oxygen is very close (2.1 Å) to hydrogen H-8 in a low-energy conformer and we hypothesize that a direct transfer of hydrogen H-8 to the hydroxyl oxygen may be the crucial low-barrier reaction leading to the formation of product **1C**. On the other hand, the conformational analysis of compound **2A** did not suggest any close contacts between the bridgehead hydrogens and the TCO hydroxy group. Relative energies of the lowest-energy conformers and conformers with the shortest bridgehead hydrogen – TCO hydroxyl oxygen together with the corresponding O···H distances are summarized in Table S4. Conformers 1–3 of compounds **1A** and **2A** were re-optimized by DFT method and the corresponding energies and distances are summarized in Table S5.

Table S4. Relative energies (kcal/mol), and O···H1 and O···H8 distances (Å) found for the lowest-energy conformation (Conformer 1), the conformation with the shortest O···H8 distance (Conformer 2) and O···H1 distance (Conformer 3) found by molecular mechanics in simulated annealings of compounds **1A**, **1B**, **2A** and **2B**.

	Conformer 1			Conformer 2			Conformer 3		
	E_{rel}	O···H1	O···H8	E_{rel}	O···H1	O···H8	E_{rel}	O···H1	O···H8
**1A**	0.00	4.06	4.15	3.48	4.19	2.26	2.09	3.81	2.34
**1B-1**	0.00	4.43	4.49	1.75	4.23	2.26	5.61	3.89	5.82
**1B-2**	0.00	4.44	4.51	1.77	4.20	2.27	2.88	3.86	5.60
**2A**	0.00	4.27	4.03	0.81	2.46	3.89	2.08	4.71	3.83
**2B-1**	0.00	3.21	4.52	1.79	2.25	4.54	7.32	3.50	4.38
**2B-2**	0.00	4.94	3.97	5.74	4.20	3.76	3.82	2.42	3.85

Table S5. Relative free energies (kcal/mol), and O···H1 and O···H8 distances (Å) found for Conformer 1–3 of compound **1A** and **2A** after geometry re-optimization with DFT method (B3LYP/6-31+G(d,p)).

	Conformer 1			Conformer 2			N-protonated Conformer 2		Conformer 3		
	G_{rel}	O···H1	O···H8	G_{rel}	O···H1	O···H8	O···H1	O···H8	G_{rel}	O···H1	O···H8
**1A**	0.00	4.22	4.19	2.14	4.12	2.11	4.09	2.01	2.28	3.88	2.32
**2A**	0.00	4.34	4.15	-0.15	2.54	3.98	2.49	3.99	-0.05	4.81	3.88

The calculated reaction mechanism

As discussed above, we expect that the first step in the reaction sequence leading to compounds **1C** and **2C** is the IEDDA followed by nitrogen elimination, which provides 4,5-dihydropyridazine intermediates **1A** and **2A**. The transition state structures were found for both these reactions starting from compound **1**; the reaction barriers were found to be 21.6 and 6.0 kcal/mol, respectively. Similar reaction barriers can also be expected for compound **2**.

Short distance was found between hydrogen H8 and TCO hydroxyl oxygen in a low-energy conformer of compound **1A**. We hypothesize that this conformer of **1A** can be protonated on a nitrogen atom (leading to even shorter O···H8 distance – Table S5) and hydrogen H8 can migrate to the TCO hydroxyl oxygen. This proton migration can be facilitated by hydration of the hydroxyl group enabling the transfer of the excess hydrogen from the hydroxyl to the solvent water molecules. The transition-state structure and a modest energy barrier (15 kcal/mol) for the hydrogen atom migration to the oxygen were found by DFT calculations.

No similarly short distance between the bridgehead hydrogen atoms and the TCO hydroxyl was found in any conformation of compound **2A** and the calculated reaction barrier of the intramolecular proton transfer in the **2A** conformer with the shortest O···H1 distance (2.49 Å – Table S5) is ca 10 kcal/mol higher than in **1A**.

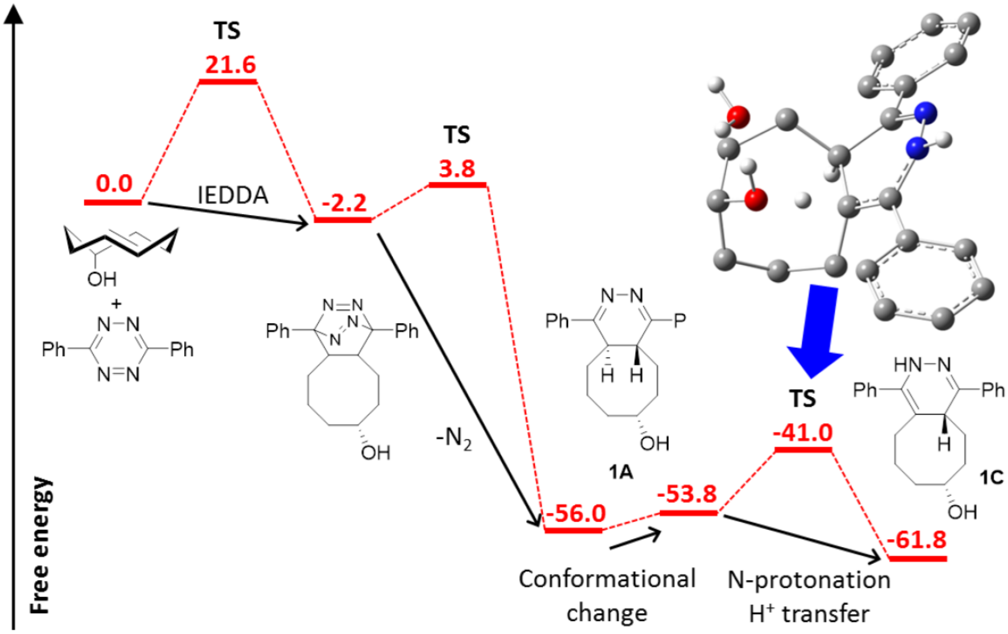

Figure S32. The calculated relative free energy profile of the reaction of axial TCO (1) with diphenyltetrazine and the subsequent prototropic rearrangement. Relative free energies are shown in kcal/mol; hydrogen atoms not important for the intramolecular proton transfer were omitted in the depicted transition-state structure.

Cartesian coordinates of ground-state and transition-state structures

Table S6. Atomic numbers, Cartesian coordinates (Å), electronic and free energies (a.u.) of the reactant, transition state and product of the IEDDA of compound **1** with dipheyltetrazine.

reactant				TS				product			
Z	*x*	*y*	*z*	*Z*	*x*	*y*	*z*	*Z*	*x*	*y*	*z*
7	0.7468	-1.9717	-1.2565	7	0.7087	-1.6787	-1.2731	7	0.6713	-1.5184	-1.2912
6	1.4440	-1.9633	-0.0996	6	1.3630	-1.3584	-0.0911	6	1.3046	-0.9892	-0.0509
6	0.4765	1.8122	0.1147	6	0.7011	0.7385	0.0854	6	0.7812	0.5018	0.0806
6	-0.8611	1.8568	0.1113	6	-0.6896	0.6794	0.1150	6	-0.7557	0.4511	0.1201
6	-1.1506	-2.1528	0.0111	6	-1.1713	-1.4761	-0.0092	6	-1.1863	-1.0695	-0.0150
7	-0.5600	-2.0686	-1.2008	7	-0.5728	-1.7436	-1.2299	7	-0.5705	-1.5620	-1.2683
6	1.2726	2.1884	1.3364	6	1.4377	1.2688	1.2968	6	1.4225	1.2153	1.2910
6	0.8831	3.6564	1.6982	6	0.8559	2.6439	1.7232	6	0.7621	2.5319	1.7449
6	0.6113	4.6232	0.5112	6	0.5872	3.6793	0.6045	6	0.6075	3.6585	0.7036
6	-0.8172	4.7019	-0.1094	6	-0.8022	3.6880	-0.0841	6	-0.7124	3.7110	-0.0847
6	-1.1390	3.8284	-1.3567	6	-1.0078	2.7545	-1.3014	6	-0.8578	2.7103	-1.2462
6	-1.6273	2.3729	-1.0796	6	-1.4803	1.3032	-1.0161	6	-1.4599	1.3234	-0.9458
6	2.9125	-1.8260	-0.1606	6	2.8431	-1.2443	-0.1516	6	2.8095	-1.1295	-0.1243
1	1.0000	1.8611	-0.8414	1	1.1530	0.9695	-0.8769	1	1.1108	0.9917	-0.8388
1	-1.3852	1.8632	1.0684	1	-1.1543	0.7330	1.0974	1	-1.1044	0.7500	1.1119
6	-2.6231	-2.2360	0.0748	6	-2.6551	-1.4974	0.0553	6	-2.6787	-1.3158	0.0258
1	2.3465	2.1193	1.1326	1	2.5013	1.3689	1.0636	1	2.4741	1.4091	1.0539
1	1.0659	1.5402	2.1968	1	1.3538	0.5817	2.1464	1	1.4195	0.5376	2.1524
1	1.6795	4.0909	2.3157	1	1.5637	3.0859	2.4342	1	1.3868	2.9117	2.5612
8	-0.9432	6.0968	-0.4919	8	-0.9572	5.0580	-0.5252	8	-0.7591	5.0581	-0.6096
1	-0.2426	3.7941	-1.9878	1	-0.0806	2.7347	-1.8865	1	0.1091	2.6155	-1.7550
1	-1.9107	4.3463	-1.9422	1	-1.7678	3.2095	-1.9494	1	-1.5324	3.1621	-1.9846
1	-1.4687	1.7704	-1.9823	1	-1.3688	0.7286	-1.9423	1	-1.4785	0.7809	-1.8972
1	-2.7031	2.3676	-0.8758	1	-2.5435	1.3014	-0.7611	1	-2.5051	1.4484	-0.6436
6	3.5648	-1.7075	-1.4015	6	3.4716	-0.8961	-1.3592	6	3.4750	-0.7747	-1.3076
6	4.9523	-1.5769	-1.4559	6	4.8609	-0.7829	-1.4252	6	4.8662	-0.8644	-1.3938
6	5.7057	-1.5632	-0.2770	6	5.6412	-1.0142	-0.2870	6	5.6110	-1.3102	-0.2970
6	5.0627	-1.6806	0.9601	6	5.0210	-1.3572	0.9186	6	4.9536	-1.6649	0.8839
6	3.6756	-1.8118	1.0214	6	3.6309	-1.4700	0.9892	6	3.5607	-1.5754	0.9718
6	-3.2766	-2.3395	1.3166	6	-3.3066	-1.3682	1.2936	6	-3.3585	-1.2019	1.2481
6	-4.6678	-2.4188	1.3737	6	-4.7003	-1.3813	1.3632	6	-4.7396	-1.3987	1.3131
6	-5.4240	-2.3966	0.1968	6	-5.4623	-1.5220	0.1980	6	-5.4601	-1.7116	0.1557
6	-4.7800	-2.2943	-1.0412	6	-4.8197	-1.6475	-1.0376	6	-4.7886	-1.8256	-1.0644
6	-3.3892	-2.2145	-1.1052	6	-3.4253	-1.6336	-1.1116	6	-3.4055	-1.6289	-1.1310
1	2.9820	-1.7177	-2.3151	1	2.8705	-0.7193	-2.2448	1	2.9055	-0.4345	-2.1669
1	5.4453	-1.4856	-2.4189	1	5.3338	-0.5142	-2.3652	1	5.3662	-0.5876	-2.3173
1	6.7859	-1.4617	-0.3220	1	6.7224	-0.9266	-0.3394	1	6.6927	-1.3804	-0.3634
1	5.6419	-1.6702	1.8784	1	5.6192	-1.5387	1.8066	1	5.5225	-2.0132	1.7411
1	3.1783	-1.9019	1.9800	1	3.1531	-1.7405	1.9239	1	3.0588	-1.8549	1.8909
1	-2.6916	-2.3569	2.2287	1	-2.7196	-1.2614	2.1997	1	-2.8068	-0.9678	2.1532
1	-5.1616	-2.4987	2.3373	1	-5.1908	-1.2810	2.3268	1	-5.2508	-1.3094	2.2672
1	-6.5071	-2.4582	0.2439	1	-6.5468	-1.5322	0.2532	1	-6.5340	-1.8657	0.2056
1	-5.3614	-2.2763	-1.9579	1	-5.4035	-1.7580	-1.9466	1	-5.3385	-2.0692	-1.9687
1	-2.8916	-2.1352	-2.0647	1	-2.9300	-1.7368	-2.0702	1	-2.8920	-1.7233	-2.0807
1	-0.0131	3.6313	2.3311	1	-0.0716	2.4812	2.2859	1	-0.2135	2.3276	2.2045
1	0.7897	5.6347	0.8928	1	0.6680	4.6703	1.0635	1	0.6646	4.6141	1.2358
1	1.3458	4.4684	-0.2896	1	1.3714	3.6344	-0.1629	1	1.4471	3.6499	-0.0049
1	-1.5562	4.4969	0.6800	1	-1.5739	3.4794	0.6734	1	-1.5510	3.5819	0.6179
1	-1.8003	6.2210	-0.9230	1	-1.8065	5.1416	-0.9810	1	-1.5781	5.1659	-1.1130
7	-0.4508	-2.1820	1.1661	7	-0.4844	-1.9023	1.1193	7	-0.5146	-1.7687	1.1165
7	0.8562	-2.0873	1.1103	7	0.7980	-1.8488	1.0753	7	0.7273	-1.7314	1.0927
E = -1146.994954				E = -1146.972939				E = -1147.015692			
G = -1146.637626				G = -1146.603235				G = -1146.641112			

Table S7. Atomic numbers, Cartesian coordinates (Å), electronic and free energies (a.u.) of the reactant, transition state and product of the nitrogen elimination reaction after IEDDA of compound **1** with dipheyltetrazine.

	reactant				TS				product		
Z	*x*	*y*	*z*	*Z*	*x*	*y*	*z*	*Z*	*x*	*y*	*z*
7	0.6713	-1.5184	-1.2912	7	0.6435	-1.3989	-1.5370	7	-2.4288	-1.6263	3.6265
6	1.3046	-0.9892	-0.0509	6	1.3631	-0.9297	0.0890	6	-1.2677	-0.7103	-0.6185
6	0.7812	0.5018	0.0806	6	0.7815	0.5019	0.1592	6	-0.5066	0.5947	-0.5416
6	-0.7557	0.4511	0.1201	6	-0.7636	0.4401	0.1534	6	0.9537	0.3982	-0.0766
6	-1.1863	-1.0695	-0.0150	6	-1.2142	-1.0464	0.0668	6	1.4563	-1.0056	-0.3360
7	-0.5705	-1.5620	-1.2683	7	-0.5257	-1.4475	-1.5108	7	-2.8957	-2.6277	3.6293
6	1.4225	1.2153	1.2910	6	1.3556	1.2042	1.4192	6	-0.6060	1.4391	-1.8340
6	0.7621	2.5319	1.7449	6	0.6738	2.5114	1.8651	6	0.1686	2.7740	-1.7625
6	0.6075	3.6585	0.7036	6	0.5715	3.6562	0.8384	6	0.0021	3.6270	-0.4830
6	-0.7124	3.7110	-0.0847	6	-0.7145	3.7259	-0.0013	6	0.9855	3.4057	0.6919
6	-0.8578	2.7103	-1.2462	6	-0.8250	2.7318	-1.1720	6	0.6575	2.2778	1.6981
6	-1.4599	1.3234	-0.9458	6	-1.4441	1.3459	-0.9010	6	1.1881	0.8611	1.3812
6	2.8095	-1.1295	-0.1243	6	2.8473	-1.0766	-0.0593	6	-2.7552	-0.6967	-0.6892
1	1.1108	0.9917	-0.8388	1	1.1268	1.0427	-0.7249	1	-1.0189	1.1747	0.2299
1	-1.1044	0.7500	1.1119	1	-1.1235	0.7419	1.1425	1	1.5826	1.0394	-0.6992
6	-2.6787	-1.3158	0.0258	6	-2.6965	-1.3108	0.0352	6	2.9181	-1.2860	-0.2975
1	2.4741	1.4091	1.0539	1	2.4182	1.4021	1.2408	1	-1.6634	1.6479	-2.0303
1	1.4195	0.5376	2.1524	1	1.3082	0.5051	2.2629	1	-0.2370	0.8594	-2.6897
1	1.3868	2.9117	2.5612	1	1.2588	2.8732	2.7183	1	-0.1693	3.3770	-2.6131
8	-0.7591	5.0581	-0.6096	8	-0.7283	5.0756	-0.5214	8	0.9723	4.6725	1.3928
1	0.1091	2.6155	-1.7550	1	0.1565	2.6384	-1.6524	1	-0.4260	2.2646	1.8670
1	-1.5324	3.1621	-1.9846	1	-1.4746	3.1911	-1.9279	1	1.0971	2.5573	2.6639
1	-1.4785	0.7809	-1.8972	1	-1.4637	0.8288	-1.8660	1	0.7180	0.1543	2.0761
1	-2.5051	1.4484	-0.6436	1	-2.4909	1.4797	-0.6078	1	2.2638	0.8282	1.5846
6	3.4750	-0.7747	-1.3076	6	3.5389	-0.3279	-1.0263	6	-3.5202	0.3458	-0.1338
6	4.8662	-0.8644	-1.3938	6	4.9201	-0.4641	-1.1800	6	-4.9156	0.3203	-0.1968
6	5.6110	-1.3102	-0.2970	6	5.6347	-1.3475	-0.3649	6	-5.5740	-0.7403	-0.8249
6	4.9536	-1.6649	0.8839	6	4.9548	-2.0970	0.5997	6	-4.8250	-1.7791	-1.3898
6	3.5607	-1.5754	0.9718	6	3.5717	-1.9675	0.7492	6	-3.4325	-1.7575	-1.3227
6	-3.3585	-1.2019	1.2481	6	-3.3682	-1.4888	1.2558	6	3.8752	-0.3041	-0.6143
6	-4.7396	-1.3987	1.3131	6	-4.7480	-1.7036	1.2842	6	5.2396	-0.6030	-0.5915
6	-5.4601	-1.7116	0.1557	6	-5.4765	-1.7489	0.0914	6	5.6756	-1.8832	-0.2392
6	-4.7886	-1.8256	-1.0644	6	-4.8143	-1.5759	-1.1269	6	4.7345	-2.8660	0.0892
6	-3.4055	-1.6289	-1.1310	6	-3.4340	-1.3553	-1.1571	6	3.3722	-2.5709	0.0601
1	2.9055	-0.4345	-2.1669	1	3.0018	0.3582	-1.6737	1	-3.0383	1.1765	0.3701
1	5.3662	-0.5876	-2.3173	1	5.4358	0.1203	-1.9361	1	-5.4861	1.1304	0.2477
1	6.6927	-1.3804	-0.3634	1	6.7095	-1.4507	-0.4813	1	-6.6586	-0.7557	-0.8780
1	5.5225	-2.0132	1.7411	1	5.5001	-2.7854	1.2389	1	-5.3264	-2.6031	-1.8891
1	3.0588	-1.8549	1.8909	1	3.0480	-2.5529	1.4957	1	-2.8508	-2.5569	-1.7677
1	-2.8068	-0.9678	2.1532	1	-2.8059	-1.4616	2.1838	1	3.5664	0.6953	-0.9014
1	-5.2508	-1.3094	2.2672	1	-5.2509	-1.8381	2.2374	1	5.9605	0.1667	-0.8509
1	-6.5340	-1.8657	0.2056	1	-6.5489	-1.9193	0.1117	1	6.7369	-2.1121	-0.2159
1	-5.3385	-2.0692	-1.9687	1	-5.3697	-1.6139	-2.0595	1	5.0631	-3.8610	0.3750
1	-2.8920	-1.7233	-2.0807	1	-2.9351	-1.2298	-2.1108	1	2.6423	-3.3284	0.3230
1	-0.2135	2.3276	2.2045	1	-0.3230	2.3019	2.2741	1	1.2379	2.6080	-1.9443
1	0.6646	4.6141	1.2358	1	0.6123	4.6035	1.3868	1	0.1548	4.6716	-0.7735
1	1.4471	3.6499	-0.0049	1	1.4392	3.6528	0.1648	1	-1.0279	3.5656	-0.1066
1	-1.5510	3.5819	0.6179	1	-1.5813	3.6010	0.6670	1	1.9962	3.2496	0.2828
1	-1.5781	5.1659	-1.1130	1	-1.5277	5.1941	-1.0532	1	1.5612	4.6087	2.1577
7	-0.5146	-1.7687	1.1165	7	-0.5344	-1.9140	0.9135	7	0.6908	-2.0087	-0.6219
7	0.7273	-1.7314	1.0927	7	0.7625	-1.8614	0.9106	7	-0.7095	-1.8777	-0.6013
	E = -1147.015692				E = -1147.002794				E = -1147.085500		
	G = -1146.641112				G = -1146.631626				G = -1146.726785		

Table S8. Atomic numbers, Cartesian coordinates (Å), electronic and free energies (a.u.) of the reactant, transition state and product of the H8 proton migration in compound **1A**.

	reactant				TS				product		
Z	*x*	*y*	*z*	*Z*	*x*	*y*	*z*	*Z*	*x*	*y*	*z*
7	-0.3080	-2.0911	0.5136	7	-0.2581	-1.5726	1.0129	7	-0.1860	-1.2874	1.2516
6	-1.1449	-1.2229	0.0078	6	-1.0335	-0.9266	0.1121	6	-0.9519	-0.8441	0.1689
6	-0.6233	0.0578	-0.5443	6	-0.4952	0.1773	-0.5646	6	-0.3865	0.0774	-0.6673
6	0.9065	0.2641	-0.3537	6	0.9701	0.5396	-0.2232	6	0.9903	0.6113	-0.2707
6	1.6496	-0.9770	0.1000	6	1.7169	-0.6773	0.3076	6	1.7707	-0.5300	0.3743
7	1.0803	-2.0704	0.4962	7	1.1188	-1.6161	0.9650	7	1.1827	-1.3708	1.1644
6	-1.0903	0.3185	-2.0031	6	-0.9813	0.5636	-1.9695	6	-0.9184	0.4211	-2.0441
6	-0.6309	1.6813	-2.5468	6	-0.6498	2.0091	-2.3911	6	-0.7648	1.8949	-2.4672
6	-1.0790	2.9505	-1.7957	6	-1.2557	3.1456	-1.5475	6	-1.5244	2.9483	-1.6451
6	-0.5877	3.2513	-0.3597	6	-0.8996	3.2120	-0.0587	6	-1.1542	3.1544	-0.1739
6	0.8816	2.8710	-0.0256	6	0.6011	3.0924	0.3031	6	0.3410	3.1448	0.2006
6	1.1897	1.4785	0.5720	6	1.1042	1.7146	0.7889	6	0.9644	1.8242	0.7059
6	-2.5779	-1.5352	0.0607	6	-2.4062	-1.4713	-0.0504	6	-2.2506	-1.5591	-0.0130
1	-1.1284	0.8185	0.0707	1	-1.2388	1.1237	0.2302	1	-1.5317	1.2267	0.4831
1	1.3364	0.4969	-1.3323	1	1.4584	0.8495	-1.1511	1	1.5063	0.9227	-1.1828
6	3.1330	-1.0023	0.0937	6	3.1821	-0.8257	0.1070	6	3.2301	-0.6933	0.1576
1	-2.1821	0.2613	-2.0443	1	-2.0591	0.3977	-2.0505	1	-1.9645	0.1151	-2.1330
1	-0.7031	-0.4732	-2.6562	1	-0.5094	-0.1113	-2.6973	1	-0.3619	-0.1829	-2.7765
1	-1.0202	1.7587	-3.5691	1	-1.0057	2.1401	-3.4191	1	-1.1193	1.9788	-3.5005
8	-1.5251	2.6870	0.5769	8	-1.6877	2.1590	0.6180	8	-1.9112	2.1405	0.6433
1	1.2439	3.5940	0.7150	1	0.8208	3.8077	1.1024	1	0.4949	3.8842	0.9931
1	1.4961	3.0333	-0.9208	1	1.1910	3.4143	-0.5627	1	0.8992	3.5108	-0.6683
1	0.6309	1.3432	1.5050	1	0.6020	1.4384	1.7233	1	0.4808	1.5147	1.6398
1	2.2489	1.4817	0.8451	1	2.1606	1.8356	1.0488	1	1.9982	2.0578	0.9816
6	-3.5164	-0.5081	0.2893	6	-3.5411	-0.6410	-0.0487	6	-3.4808	-0.8855	-0.0945
6	-4.8725	-0.8139	0.3794	6	-4.8149	-1.1912	-0.1923	6	-4.6708	-1.5973	-0.2641
6	-5.3105	-2.1334	0.2231	6	-4.9746	-2.5724	-0.3457	6	-4.6523	-2.9922	-0.3509
6	-4.3868	-3.1546	-0.0228	6	-3.8522	-3.4047	-0.3511	6	-3.4346	-3.6737	-0.2650
6	-3.0271	-2.8621	-0.1002	6	-2.5755	-2.8604	-0.1991	6	-2.2444	-2.9640	-0.0936
6	3.8795	-0.1976	-0.7872	6	3.9439	0.1756	-0.5228	6	3.9968	0.3068	-0.4687
6	5.2738	-0.2598	-0.7938	6	5.3202	0.0183	-0.7097	6	5.3721	0.1435	-0.6600
6	5.9453	-1.1130	0.0861	6	5.9631	-1.1395	-0.2685	6	6.0105	-1.0212	-0.2293
6	5.2132	-1.9105	0.9734	6	5.2172	-2.1442	0.3613	6	5.2593	-2.0262	0.3937
6	3.8211	-1.8591	0.9761	6	3.8453	-1.9920	0.5435	6	3.8881	-1.8672	0.5804
1	-3.1871	0.5166	0.4252	1	-3.4347	0.4298	0.0838	1	-3.5216	0.1957	-0.0161
1	-5.5880	-0.0212	0.5722	1	-5.6834	-0.5401	-0.1788	1	-5.6123	-1.0594	-0.3212
1	-6.3694	-2.3641	0.2855	1	-5.9677	-2.9962	-0.4593	1	-5.5787	-3.5433	-0.4817
1	-4.7249	-4.1758	-0.1643	1	-3.9665	-4.4769	-0.4761	1	-3.4083	-4.7569	-0.3342
1	-2.3256	-3.6573	-0.3331	1	-1.7082	-3.5132	-0.2249	1	-1.3024	-3.5025	-0.0414
1	3.3852	0.4670	-1.4867	1	3.4783	1.0898	-0.8717	1	3.5337	1.2274	-0.8056
1	5.8334	0.3594	-1.4879	1	5.8857	0.8053	-1.1993	1	5.9412	0.9315	-1.1441
1	7.0302	-1.1538	0.0839	1	7.0323	-1.2613	-0.4140	1	7.0784	-1.1486	-0.3796
1	5.7279	-2.5685	1.6668	1	5.7065	-3.0508	0.7051	1	5.7433	-2.9402	0.7259
1	3.2548	-2.4704	1.6695	1	3.2716	-2.7769	1.0224	1	3.3094	-2.6543	1.0504
1	0.4591	1.6952	-2.6625	1	0.4369	2.1384	-2.4460	1	0.2976	2.1602	-2.5047
1	-0.7558	3.7963	-2.4147	1	-0.9327	4.0983	-1.9836	1	-1.3572	3.9220	-2.1217
1	-2.1749	2.9974	-1.7688	1	-2.3498	3.1283	-1.6217	1	-2.6052	2.7702	-1.6951
1	-0.6487	4.3436	-0.2597	1	-1.3004	4.1458	0.3410	1	-1.6152	4.0846	0.1597
1	-1.3462	3.0427	1.4721	1	-1.6919	2.2530	1.6246	1	-2.0160	2.3671	1.6676
1	-0.6603	-2.9292	0.9664	1	-0.6565	-2.2982	1.5937	1	-0.5734	-2.0285	1.8191
1	-1.9264	3.8700	3.6635	1	-2.6882	2.5864	3.5499	1	-3.0848	3.1028	3.2512
8	-1.1067	3.6513	3.1985	8	-1.8127	2.3354	3.2210	8	-2.2620	2.6274	3.0593
1	-0.5326	4.4213	3.3154	1	-1.1793	2.8982	3.6899	1	-1.5585	3.0720	3.5564
	E = -1114.455009				E = -1114.432184				E = -1114.437856		
	G = -1114.056821				G = -1114.036376				G = -1114.038198		

Copies of NMR spectra

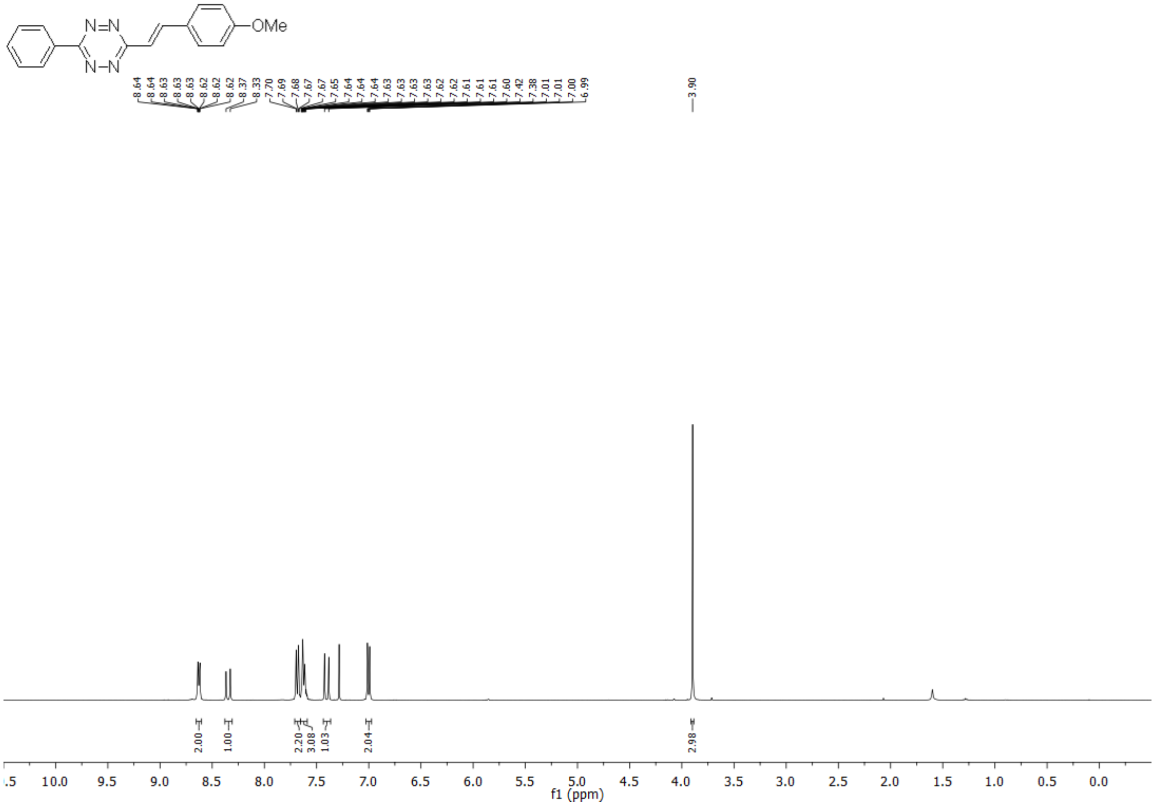

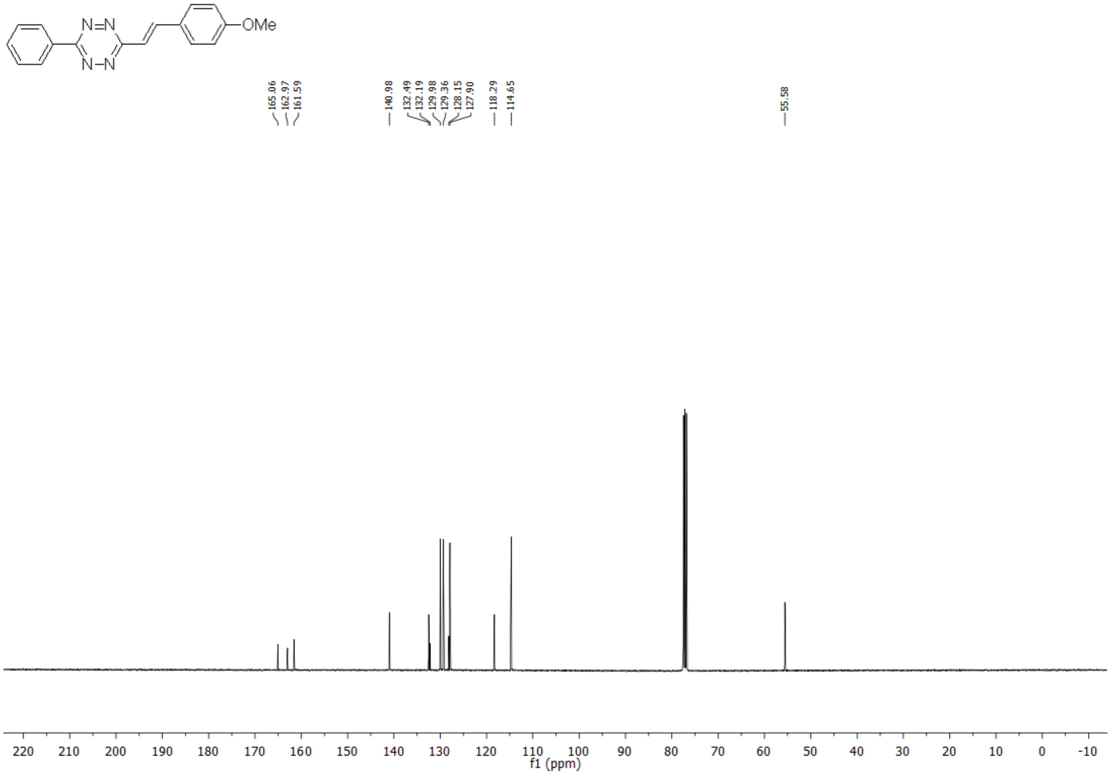

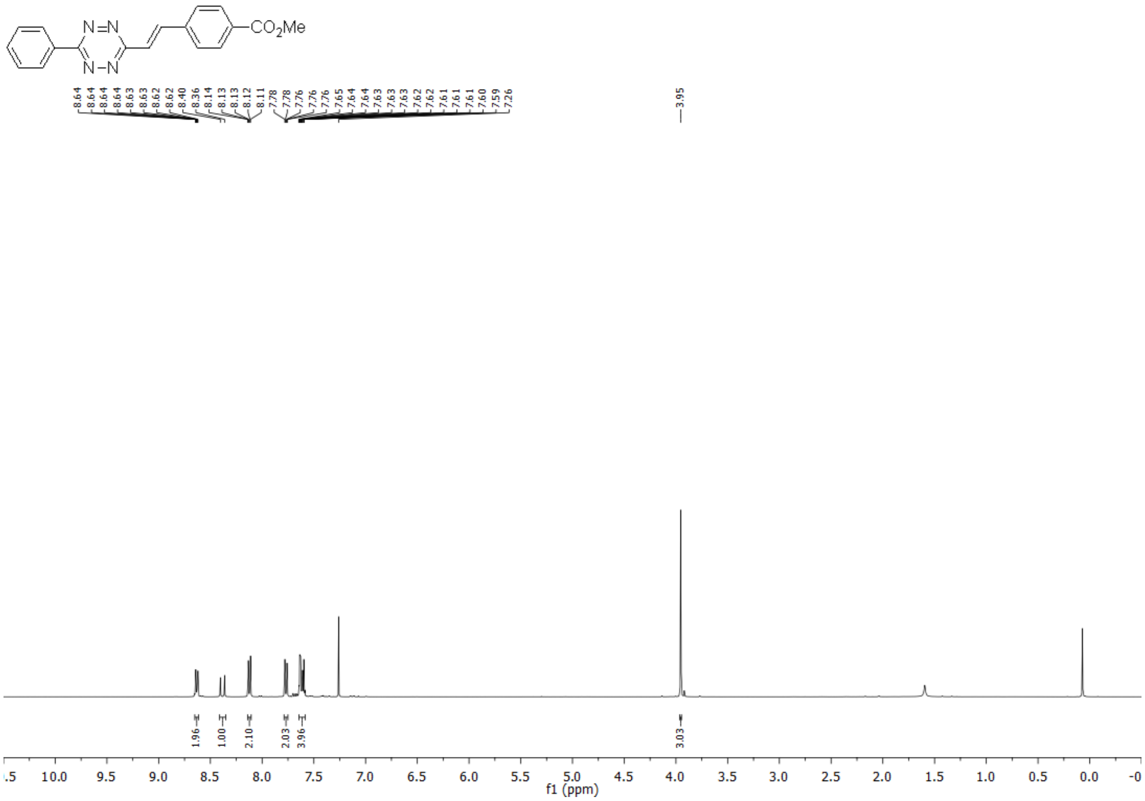
CO2Me
3.95
1.96
1.00
2.10
2.03
3.96
3.03
f1 (ppm)

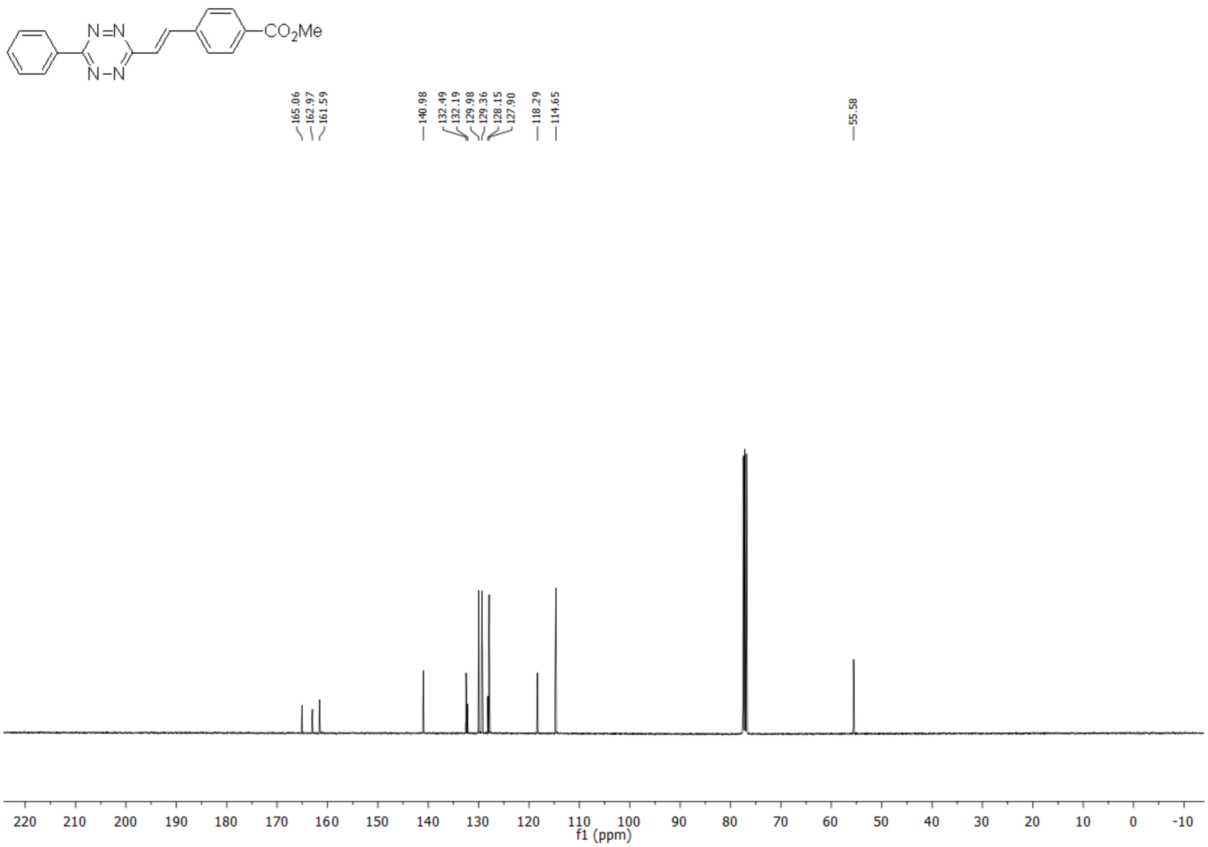
CO2Me
165.06
162.97
161.59
140.98
132.49
132.19
129.98
129.36
128.15
127.90
118.29
114.65
55.58
f1 (ppm)

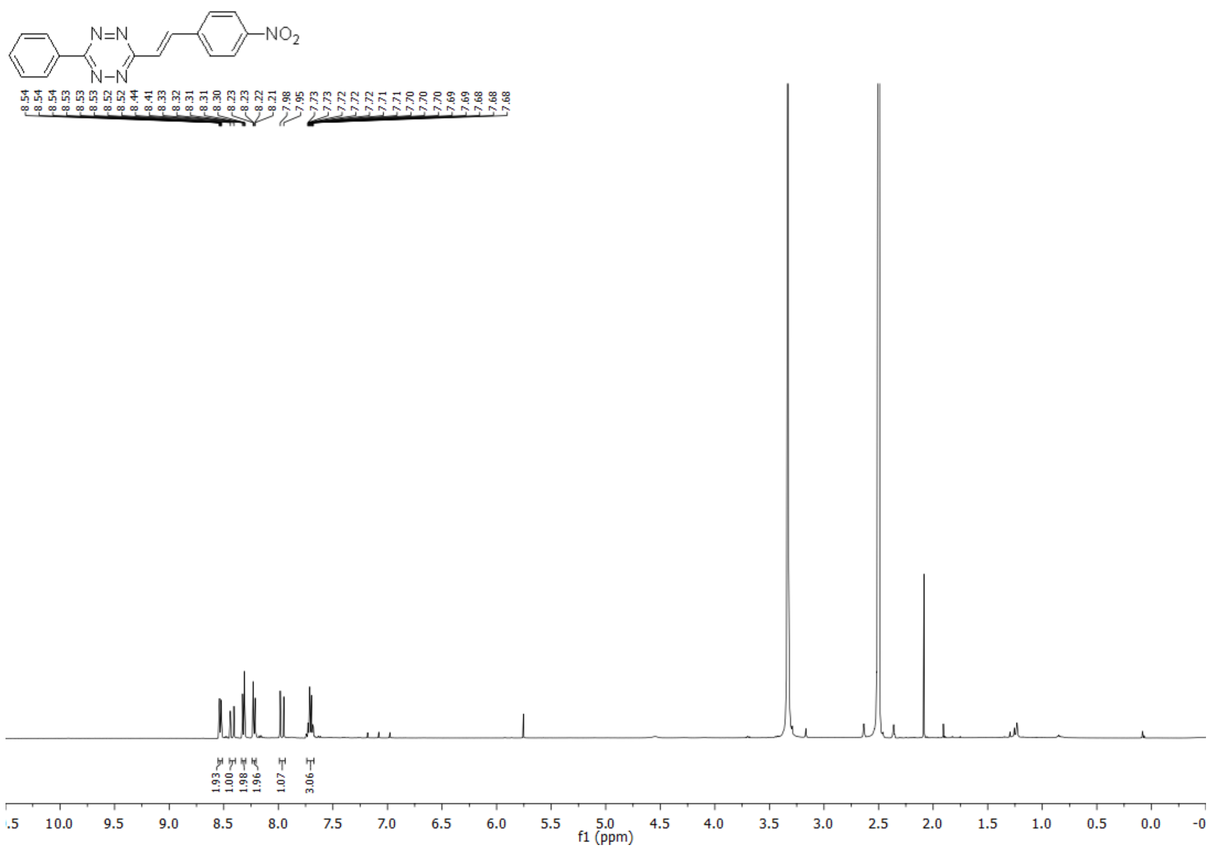
NO2
8.54
8.54
8.54
8.53
8.53
8.53
8.52
8.52
8.44
8.41
8.33
8.32
8.31
8.30
8.23
8.23
8.22
8.21
7.98
7.95
7.73
7.73
7.72
7.72
7.72
7.71
7.71
7.70
7.70
7.70
7.69
7.68
7.68
7.68
1.93
1.00
1.98
1.96
1.07
3.06
f1 (ppm)

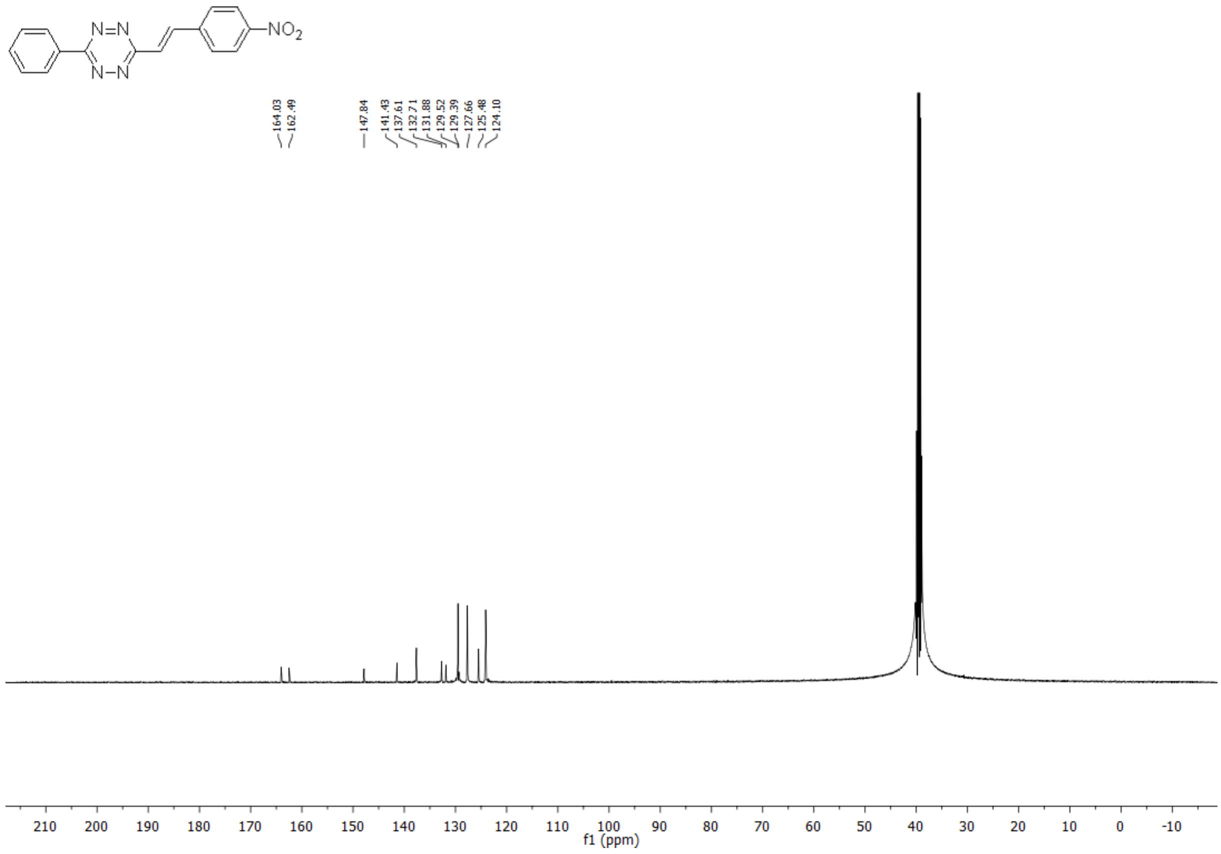
NO2
164.03
162.49
147.84
141.43
137.61
132.71
131.88
129.52
129.39
127.66
125.48
124.10
f1 (ppm)

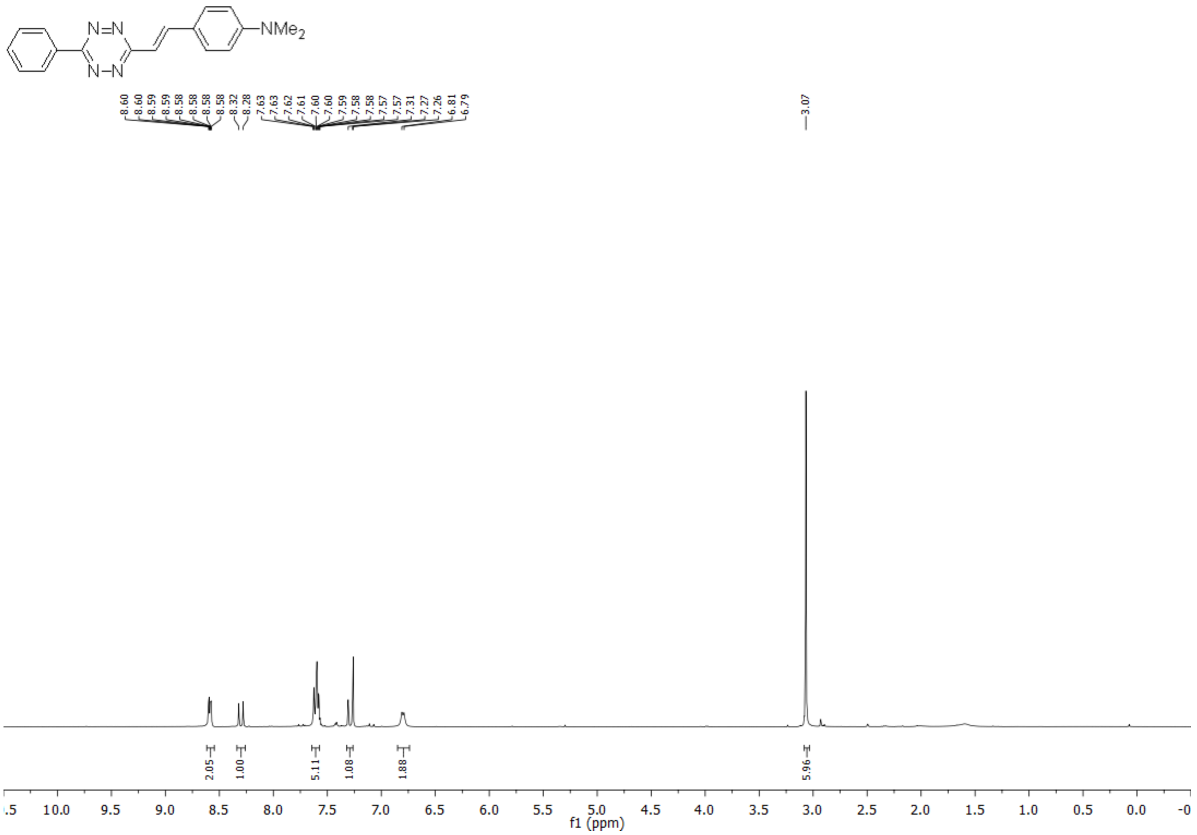
N=N
N–N
NMe2
8.60
8.60
8.59
8.59
8.58
8.58
8.58
8.58
8.32
8.28
7.63
7.63
7.62
7.61
7.60
7.60
7.59
7.58
7.58
7.57
7.57
7.31
7.27
7.26
6.81
6.79
3.07
2.05
1.00
5.11
1.08
1.88
5.96
f1 (ppm)

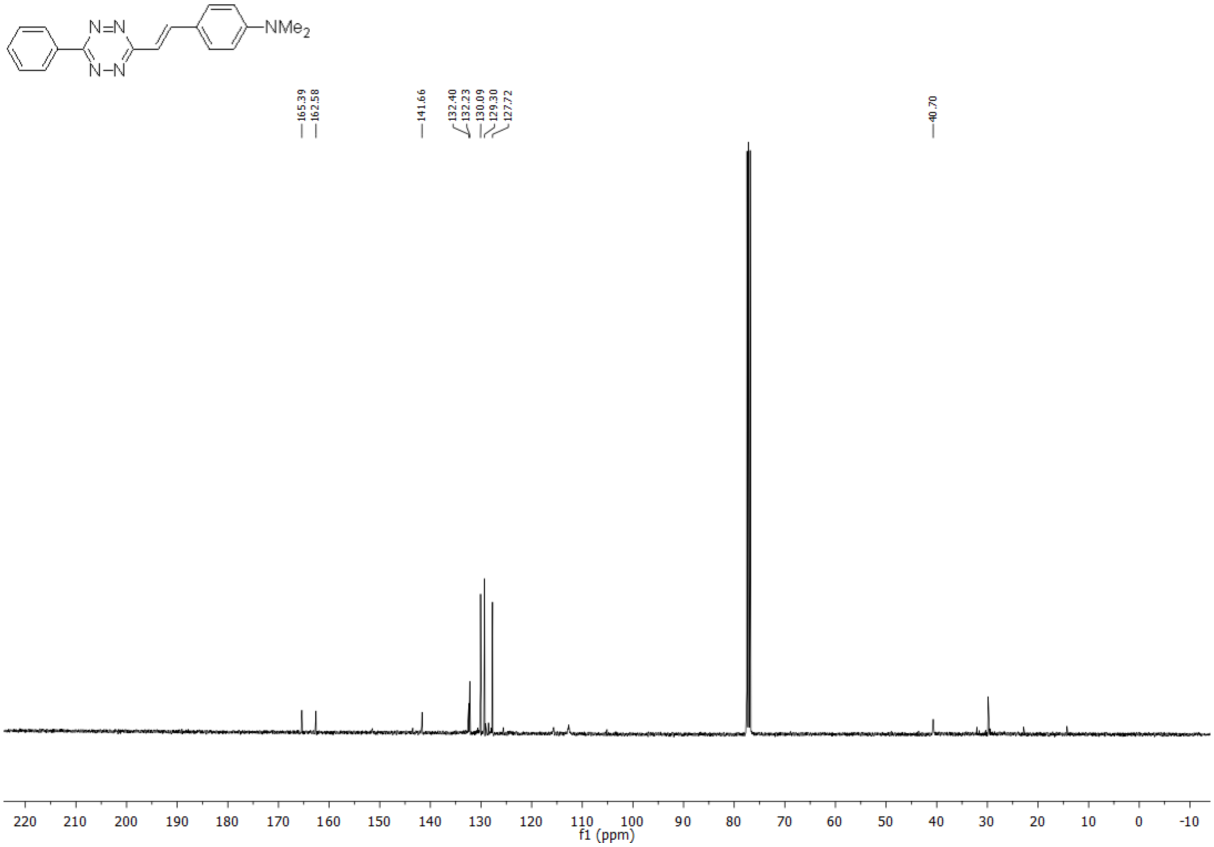
N=N
N–N
NMe2
165.39
162.58
141.66
132.40
132.23
130.09
129.30
127.72
40.70
f1 (ppm)

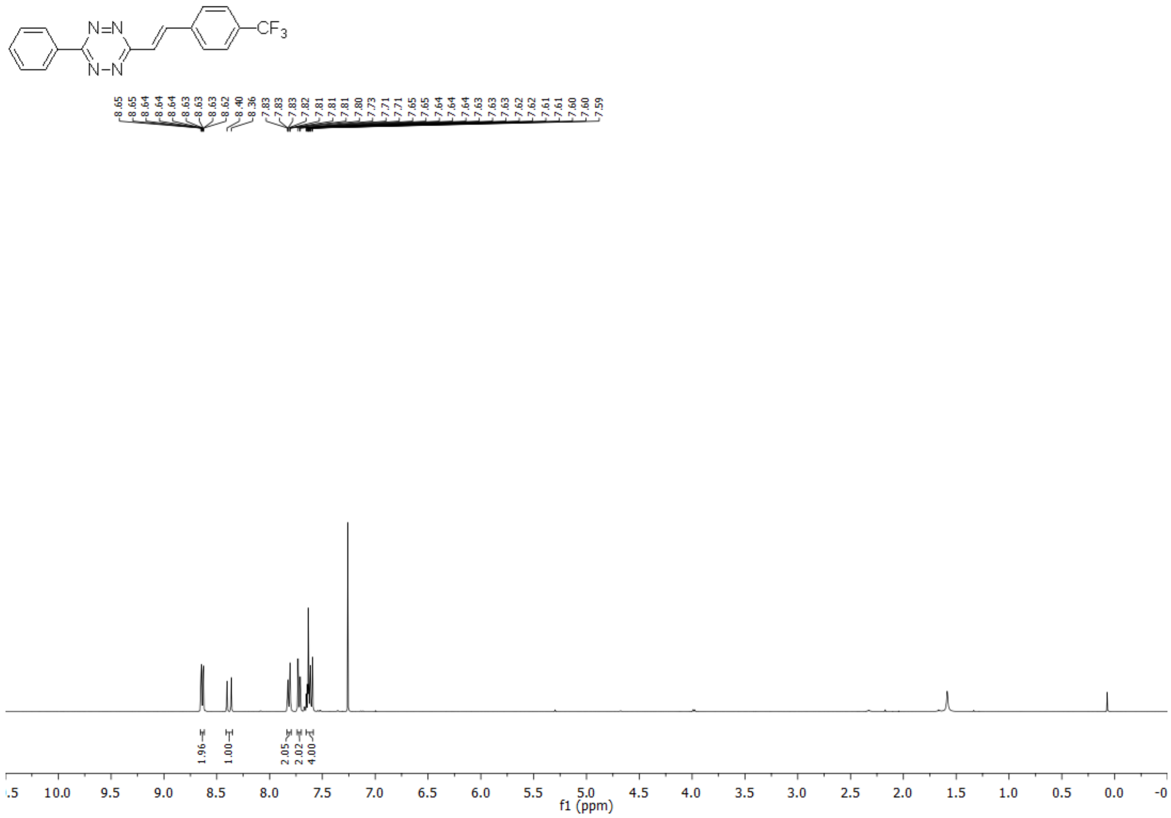
CF3
N=N
N–N
1.96
1.00
2.05
2.02
4.00
f1 (ppm)

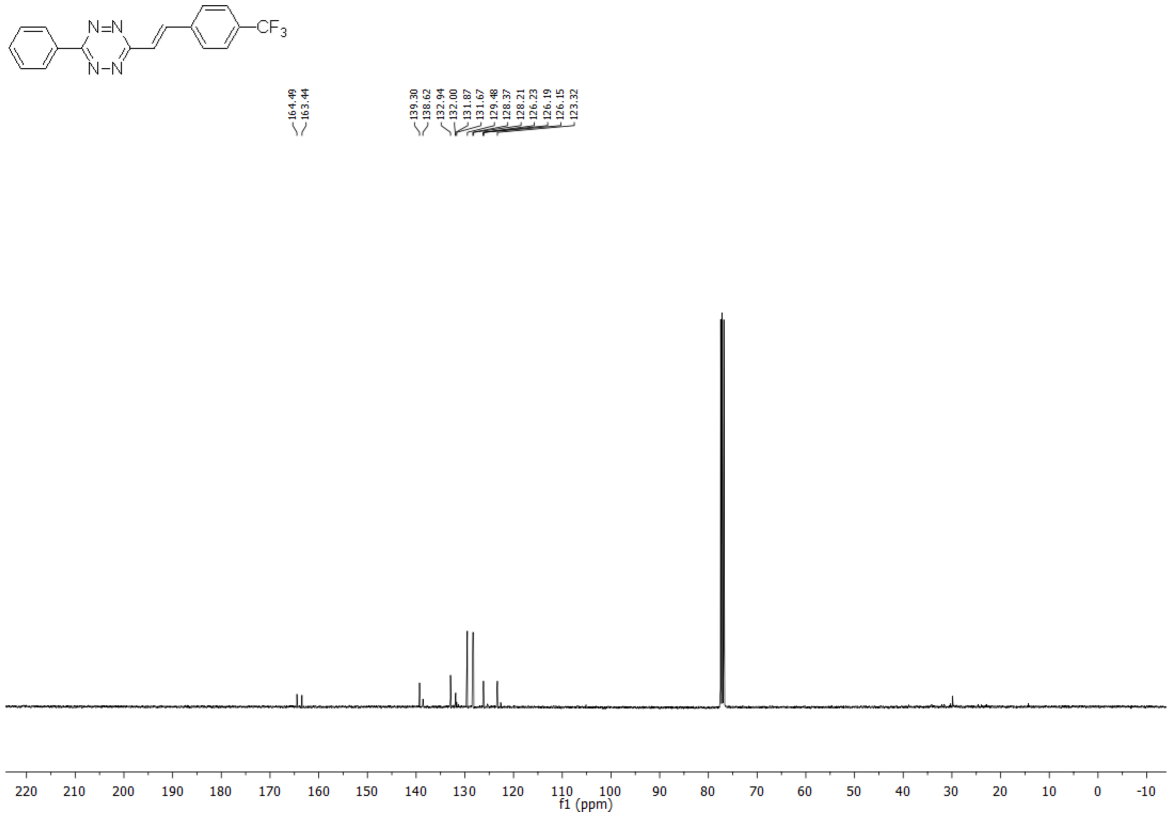
CF3
N=N
N–N
164.49
163.44
139.30
138.62
132.94
132.00
131.87
131.67
129.48
128.37
128.21
126.23
126.19
126.15
123.32
f1 (ppm)

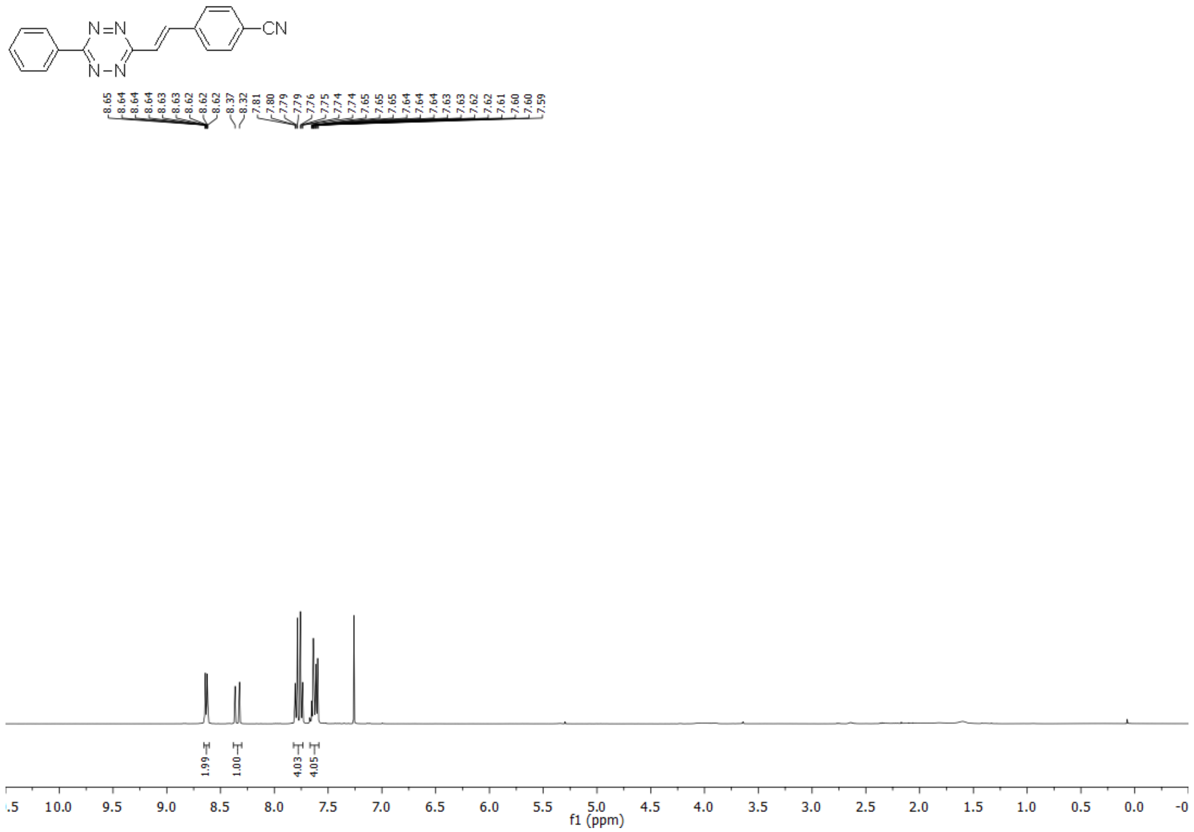
CN
N=N
N–N
8.65 8.64 8.64 8.63 8.63 8.62 8.62 8.62 8.37 8.32 7.81 7.80 7.79 7.79 7.76 7.75 7.74 7.74 7.65 7.65 7.65 7.64 7.64 7.63 7.63 7.62 7.62 7.61 7.60 7.59
1.99
1.00
4.03
4.05
f1 (ppm)

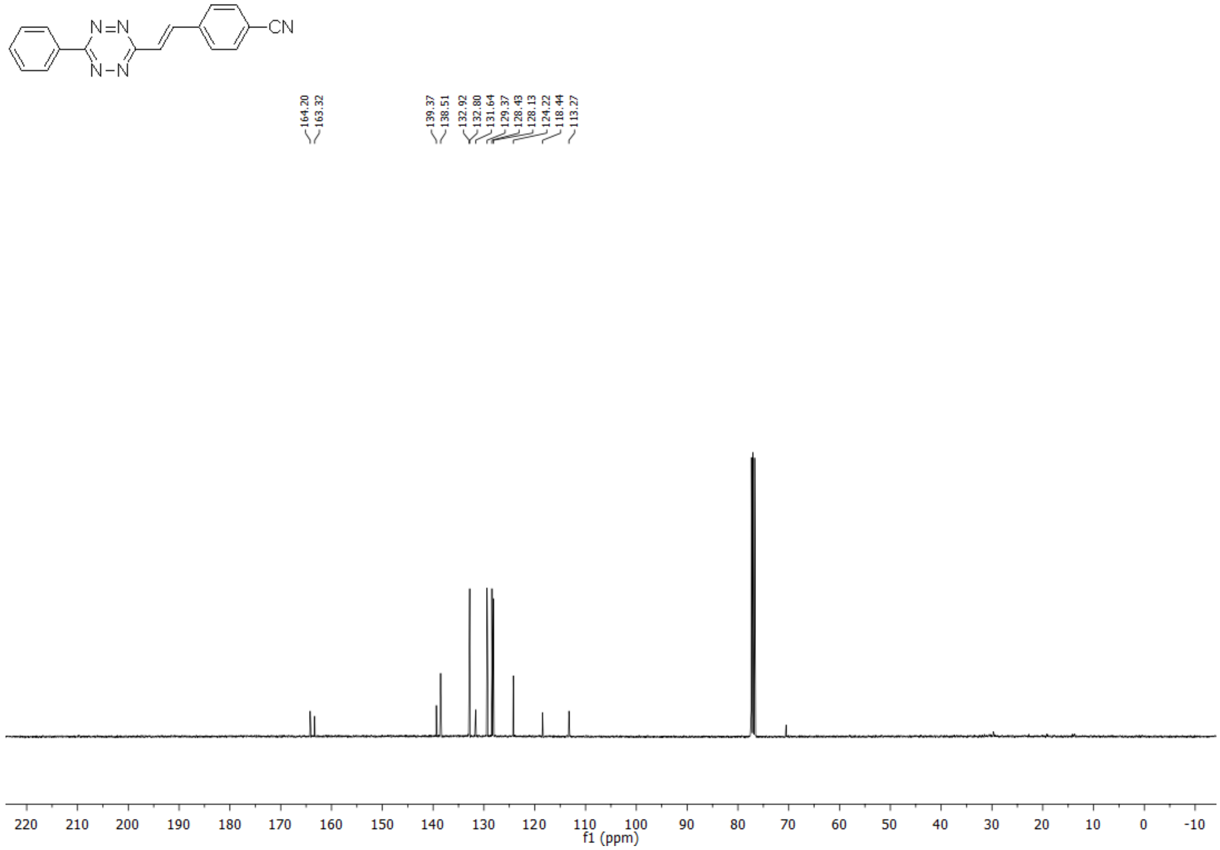
CN
N=N
N–N
164.20
163.32
139.37
138.51
132.92
132.80
131.64
129.37
128.43
128.13
124.22
118.44
113.27
f1 (ppm)

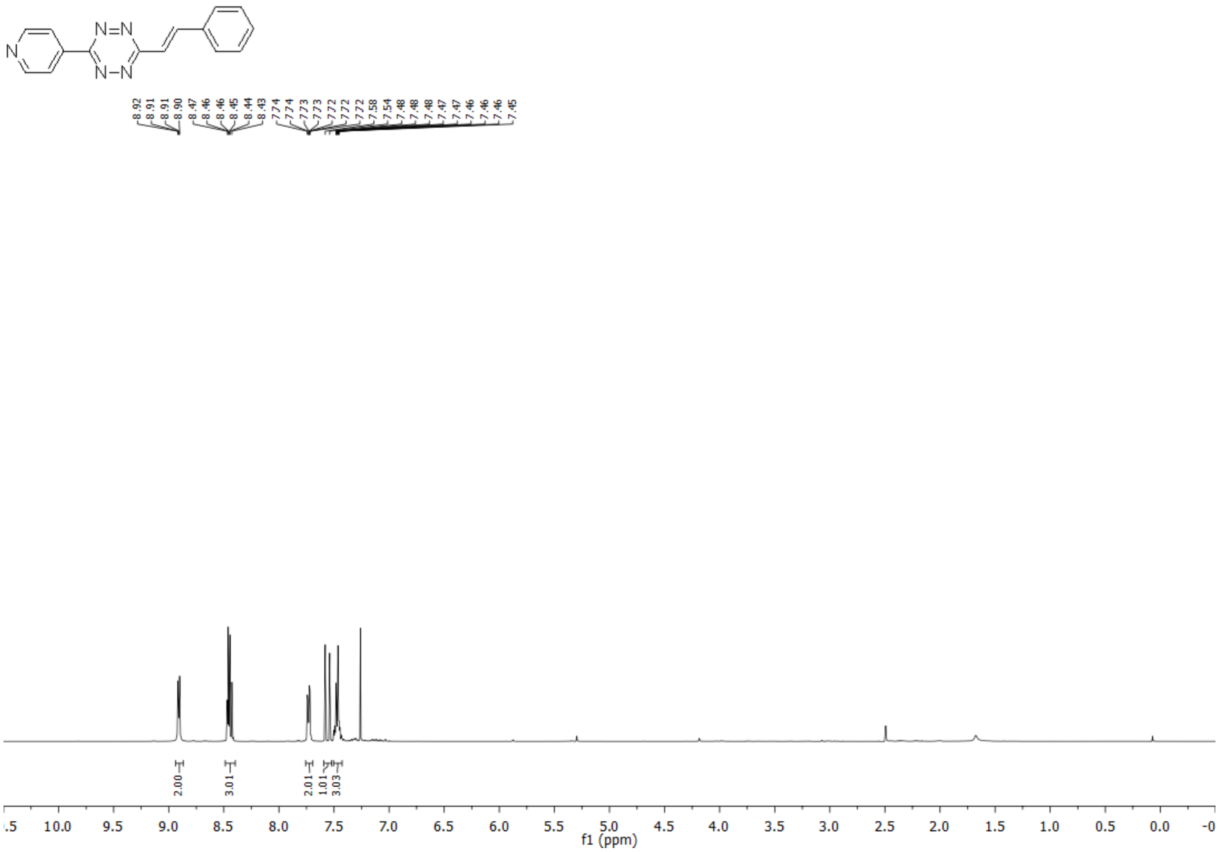
8.92
8.91
8.91
8.90
8.47
8.46
8.46
8.45
8.44
8.43
7.74
7.74
7.73
7.73
7.72
7.72
7.72
7.72
7.58
7.54
7.48
7.48
7.48
7.47
7.47
7.46
7.46
7.46
7.45
2.00
3.01
2.01
1.01
3.03
f1 (ppm)

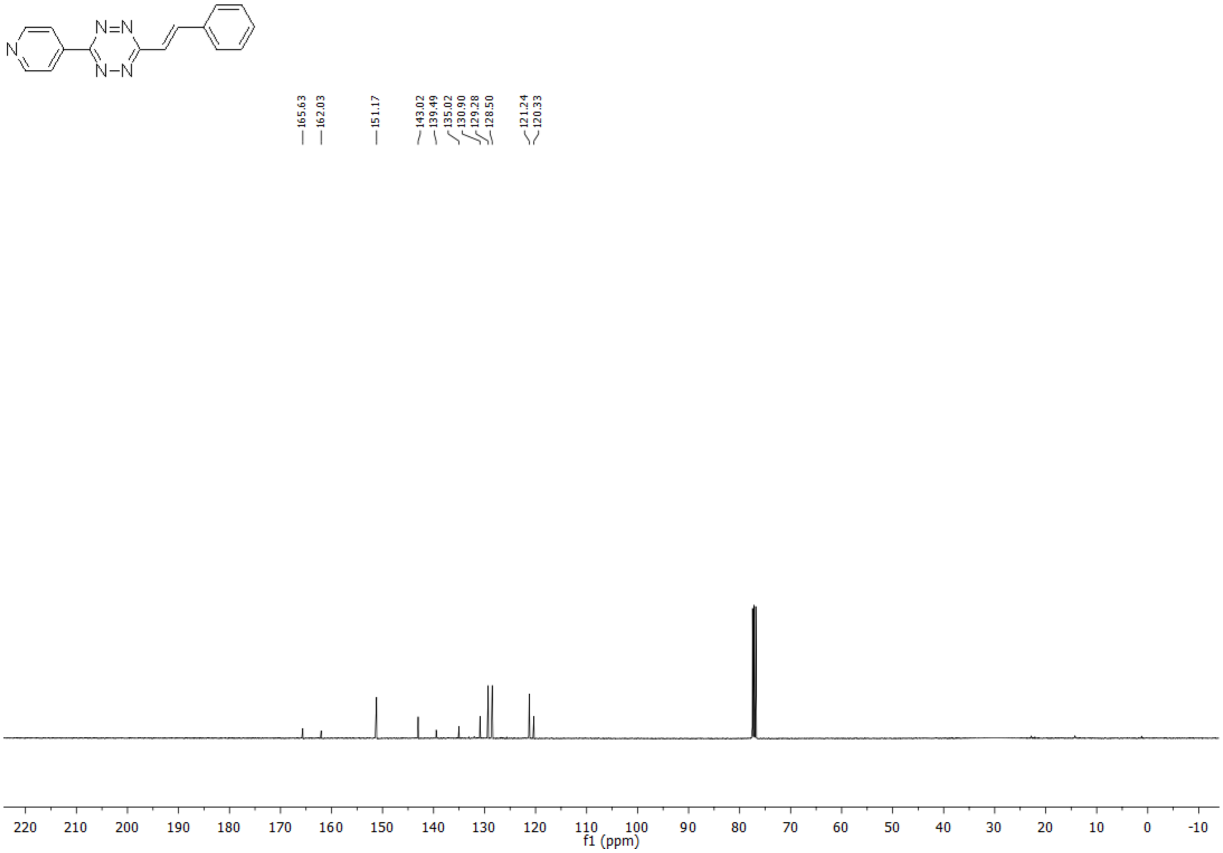
165.63
162.03
151.17
143.02
139.49
135.02
130.90
129.28
128.50
121.24
120.33
f1 (ppm)

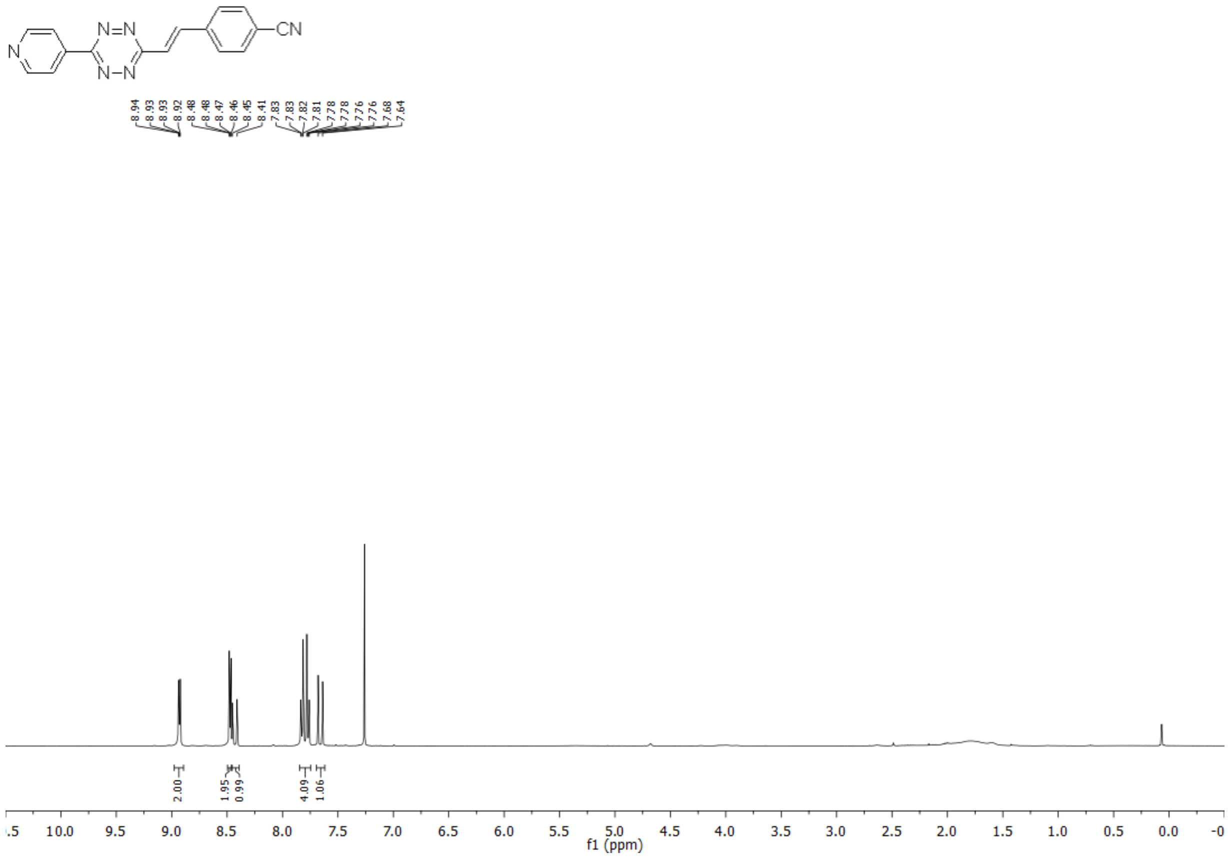
N=N
N–N
N
CN
8.94
8.93
8.93
8.92
8.48
8.48
8.47
8.46
8.45
8.41
7.83
7.83
7.82
7.81
7.78
7.78
7.76
7.76
7.68
7.64
2.00
1.95
0.99
4.09
1.06
f1 (ppm)

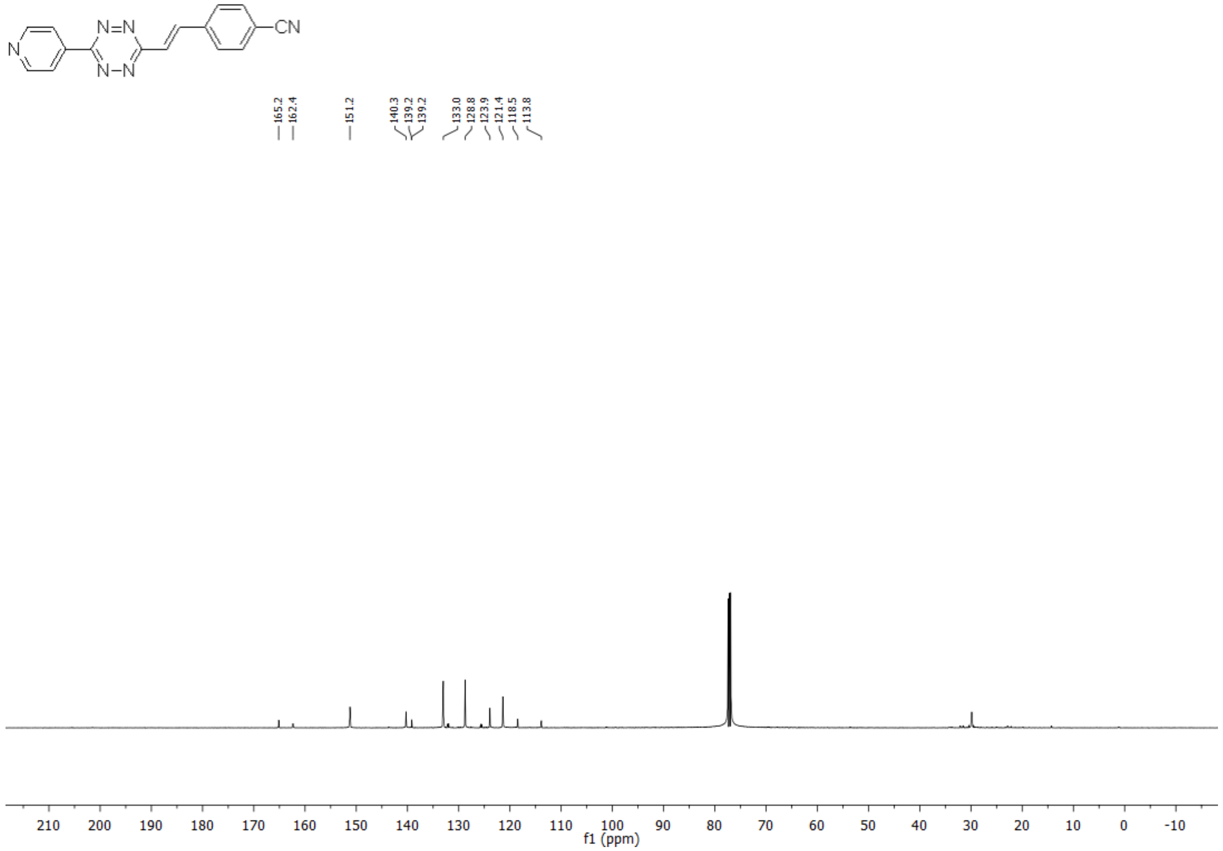
N=N
N–N
N
CN
165.2
162.4
151.2
140.3
139.2
139.2
133.0
128.8
123.9
121.4
118.5
113.8
f1 (ppm)

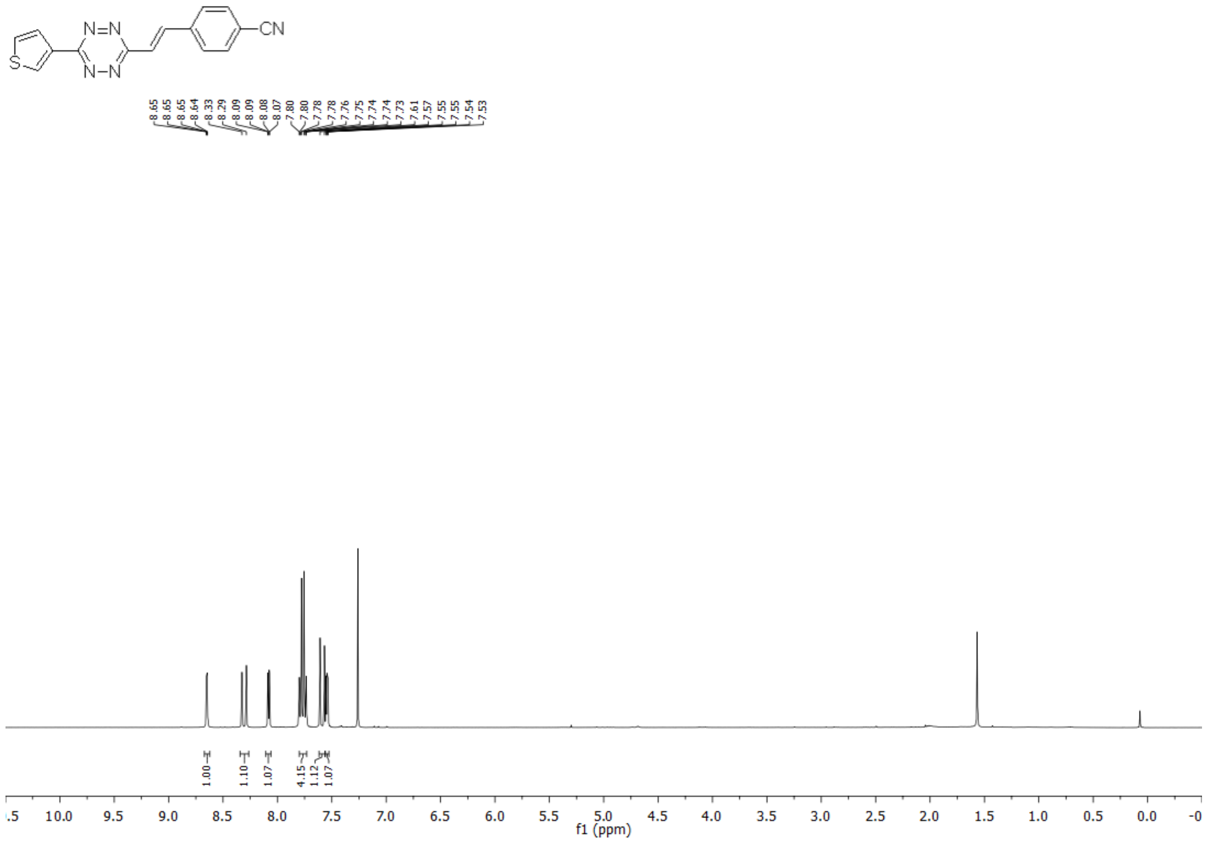
CN
8.65
8.65
8.65
8.64
8.33
8.29
8.09
8.09
8.08
8.07
7.80
7.80
7.78
7.78
7.76
7.75
7.74
7.74
7.73
7.61
7.57
7.55
7.55
7.54
7.53
1.00
1.10
1.07
4.15
1.12
1.07
10.0 9.5 9.0 8.5 8.0 7.5 7.0 6.5 6.0 5.5 5.0 4.5 4.0 3.5 3.0 2.5 2.0 1.5 1.0 0.5 0.0
f1 (ppm)

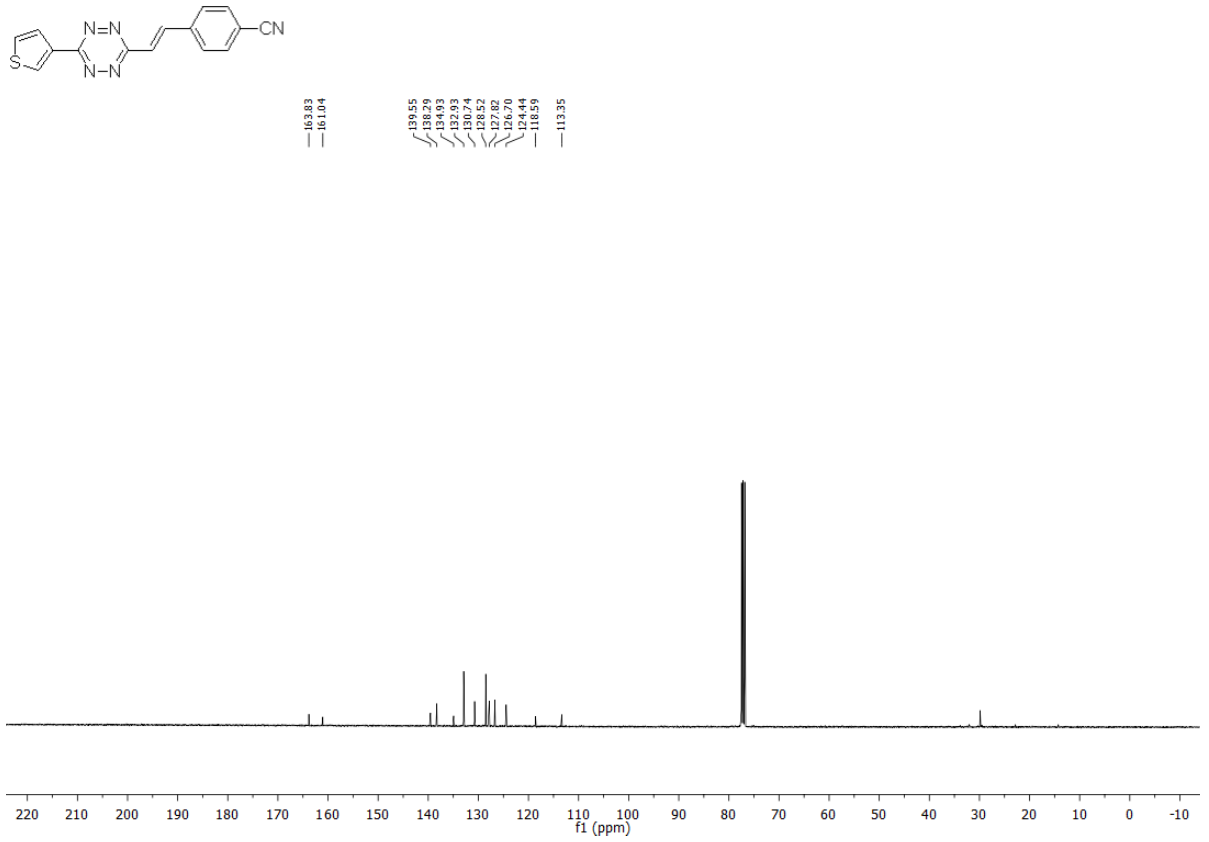
CN
163.83
161.04
139.55
138.29
134.93
132.93
130.74
128.52
127.82
126.70
124.44
118.59
113.35
220 210 200 190 180 170 160 150 140 130 120 110 100 90 80 70 60 50 40 30 20 10 0 -10
f1 (ppm)

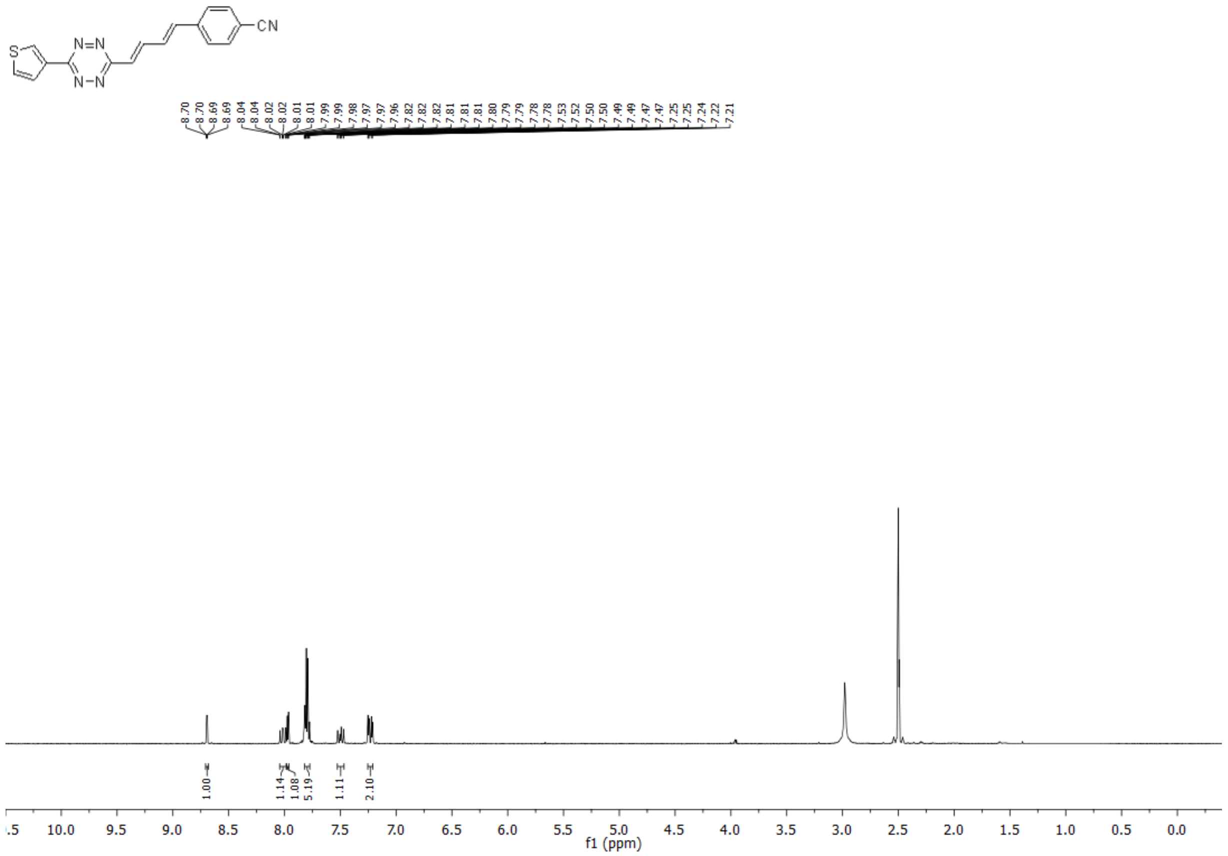
N=N
N-N
S
CN
8.70
8.70
8.69
8.69
8.04
8.04
8.02
8.02
8.01
8.01
7.99
7.99
7.98
7.97
7.97
7.96
7.82
7.82
7.82
7.81
7.81
7.81
7.80
7.79
7.79
7.78
7.78
7.53
7.52
7.50
7.50
7.49
7.49
7.47
7.25
7.25
7.24
7.22
7.21
1.00
1.14
1.08
5.19
1.11
2.10
f1 (ppm)

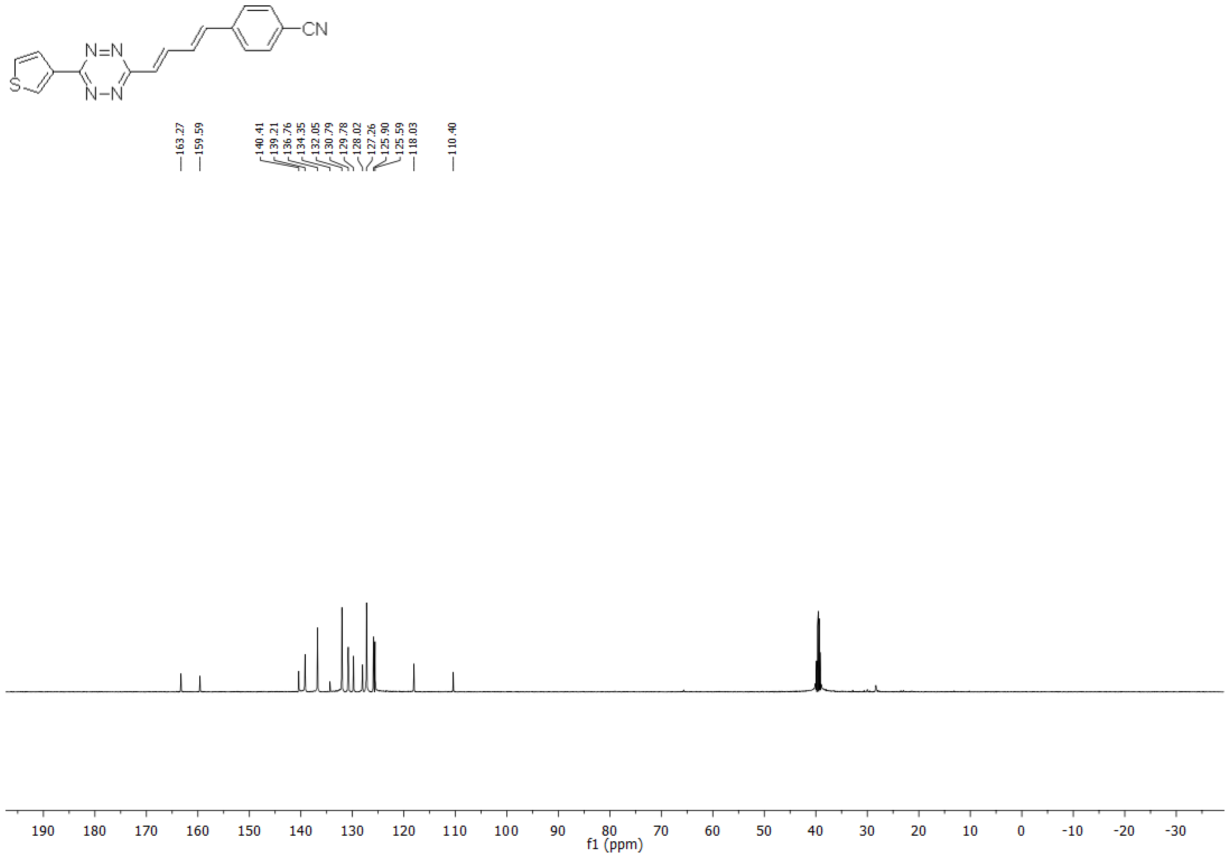
N=N
N-N
S
CN
163.27
159.59
140.41
139.21
136.76
134.35
132.05
130.79
129.78
128.02
127.26
125.90
125.59
118.03
110.40
f1 (ppm)

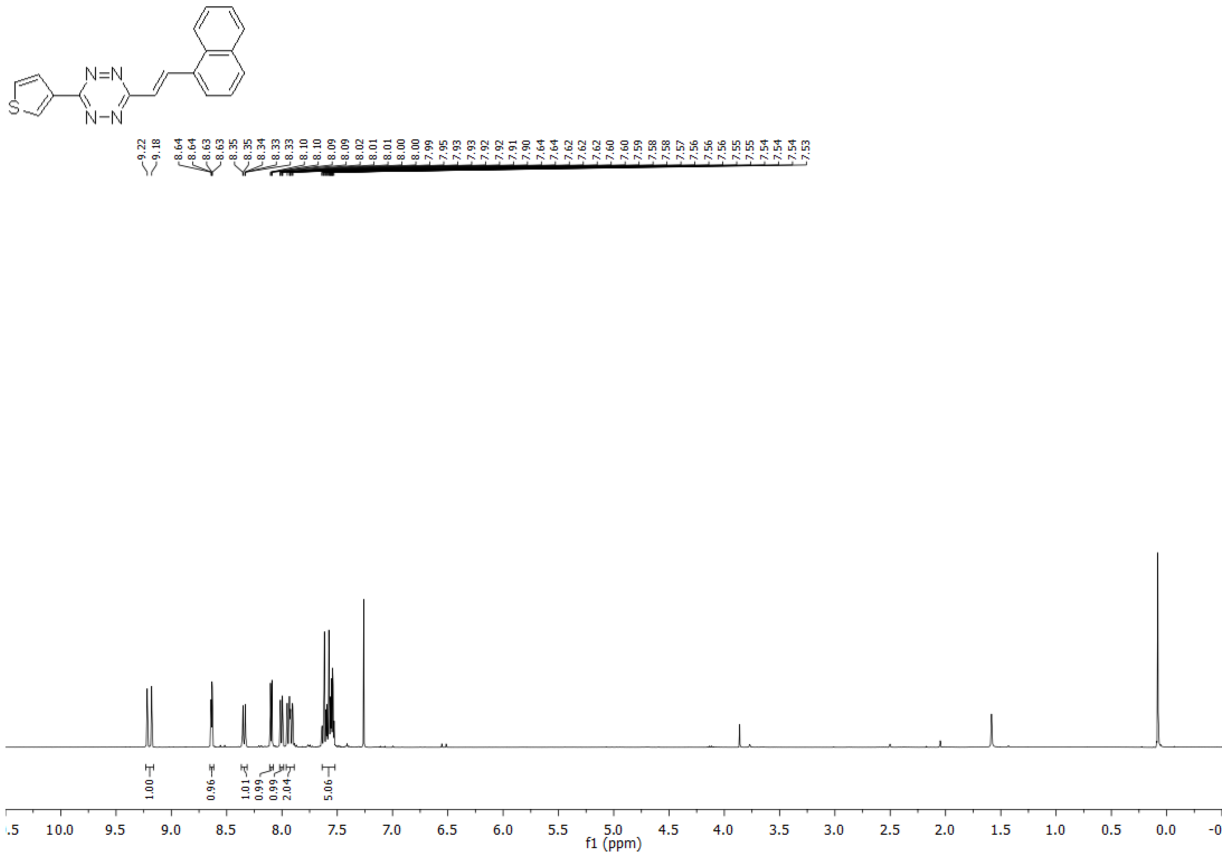

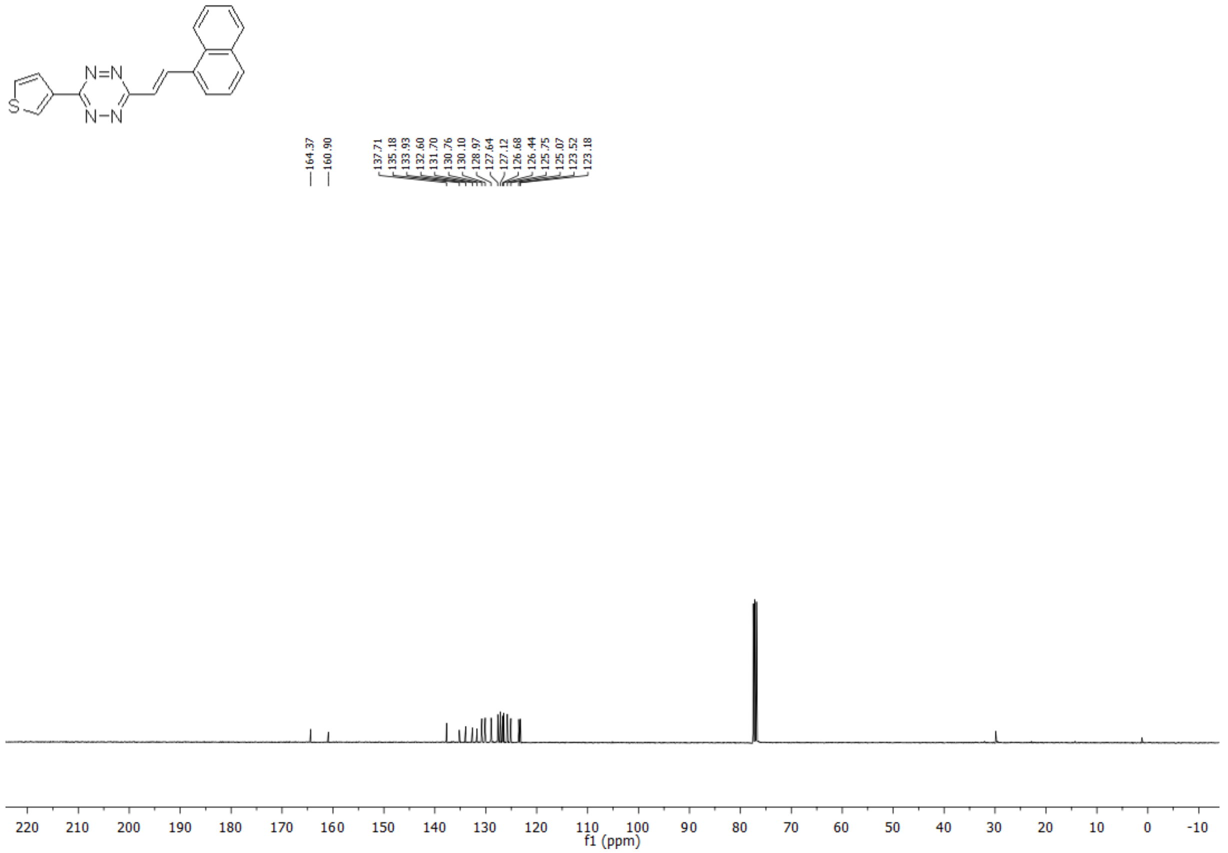

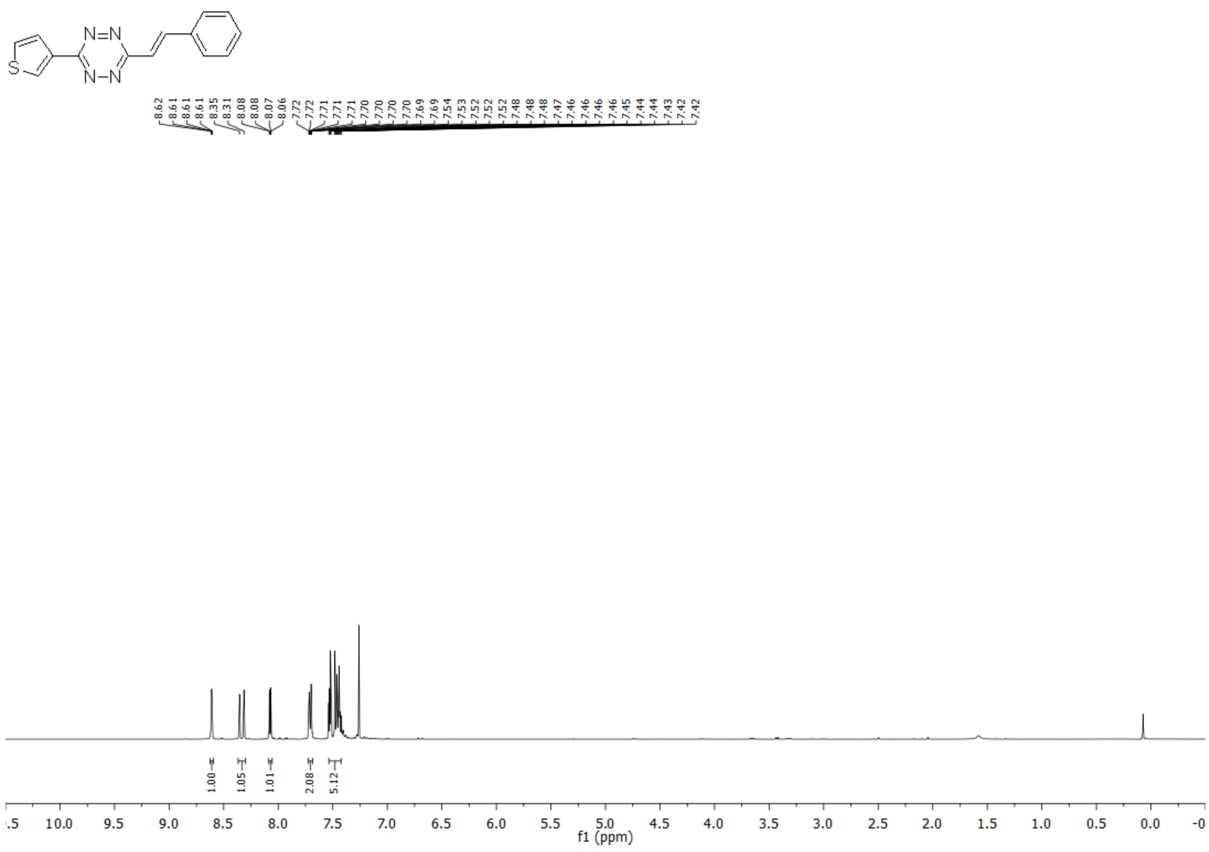
N=N
N–N
S
8.62 8.61 8.61 8.61 8.35 8.31 8.08 8.08 8.07 8.06 7.72 7.72 7.71 7.71 7.71 7.70 7.70 7.70 7.69 7.69 7.54 7.53 7.52 7.52 7.52 7.48 7.48 7.48 7.47 7.46 7.46 7.46 7.45 7.44 7.44 7.43 7.42 7.42
1.00 1.05 1.01 2.08 5.12
f1 (ppm)

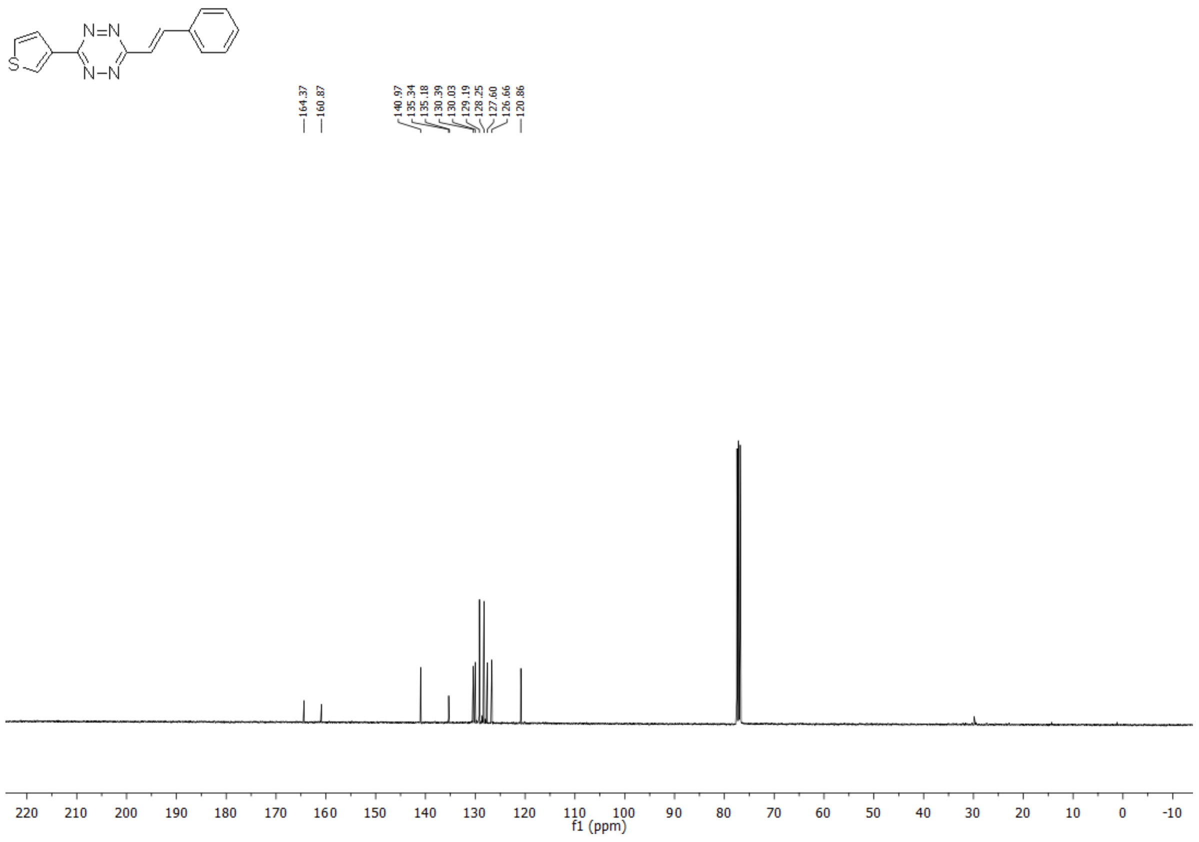
N=N
N–N
S
164.37 160.87
140.97 135.34 135.18 130.39 130.03 129.19 128.25 127.60 126.66 120.86
f1 (ppm)

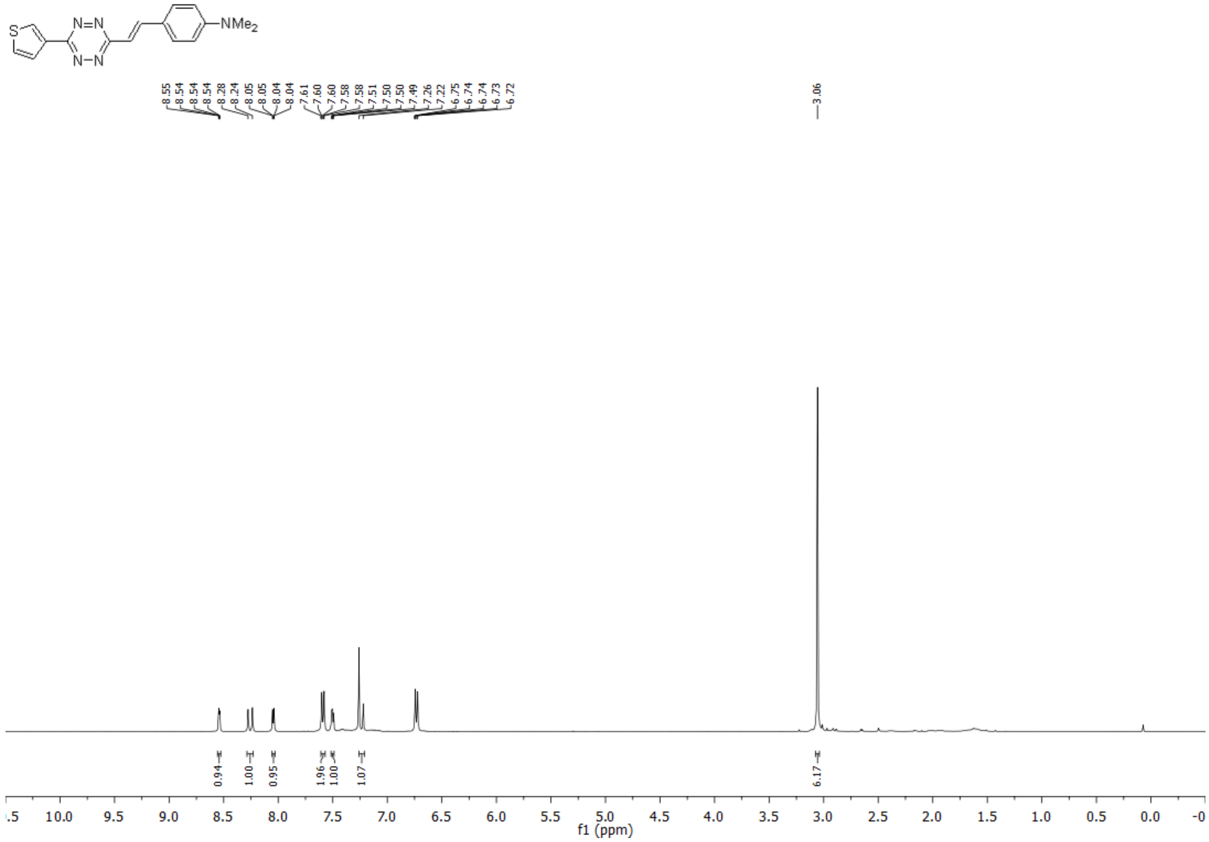
NMe2
8.55 8.54 8.54 8.54 8.28 8.24 8.05 8.05 8.04 8.04 7.61 7.60 7.60 7.58 7.58 7.51 7.50 7.50 7.49 7.26 7.22 6.75 6.74 6.74 6.73 6.72
3.06
0.94 1.00 0.95 1.96 1.00 1.07
6.17
f1 (ppm)

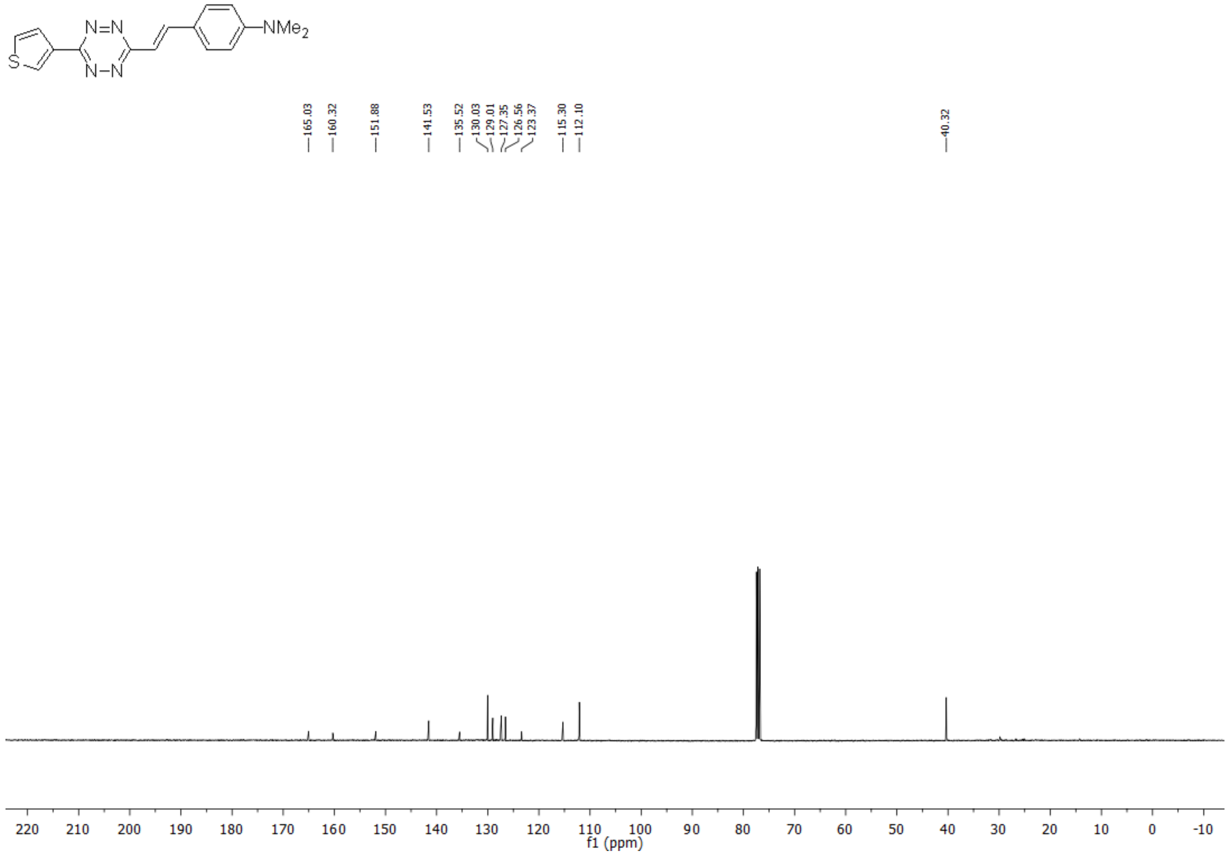
NMe2
165.03 160.32 151.88 141.53 135.52 130.03 129.01 127.35 126.56 123.37 115.30 112.10
40.32
f1 (ppm)

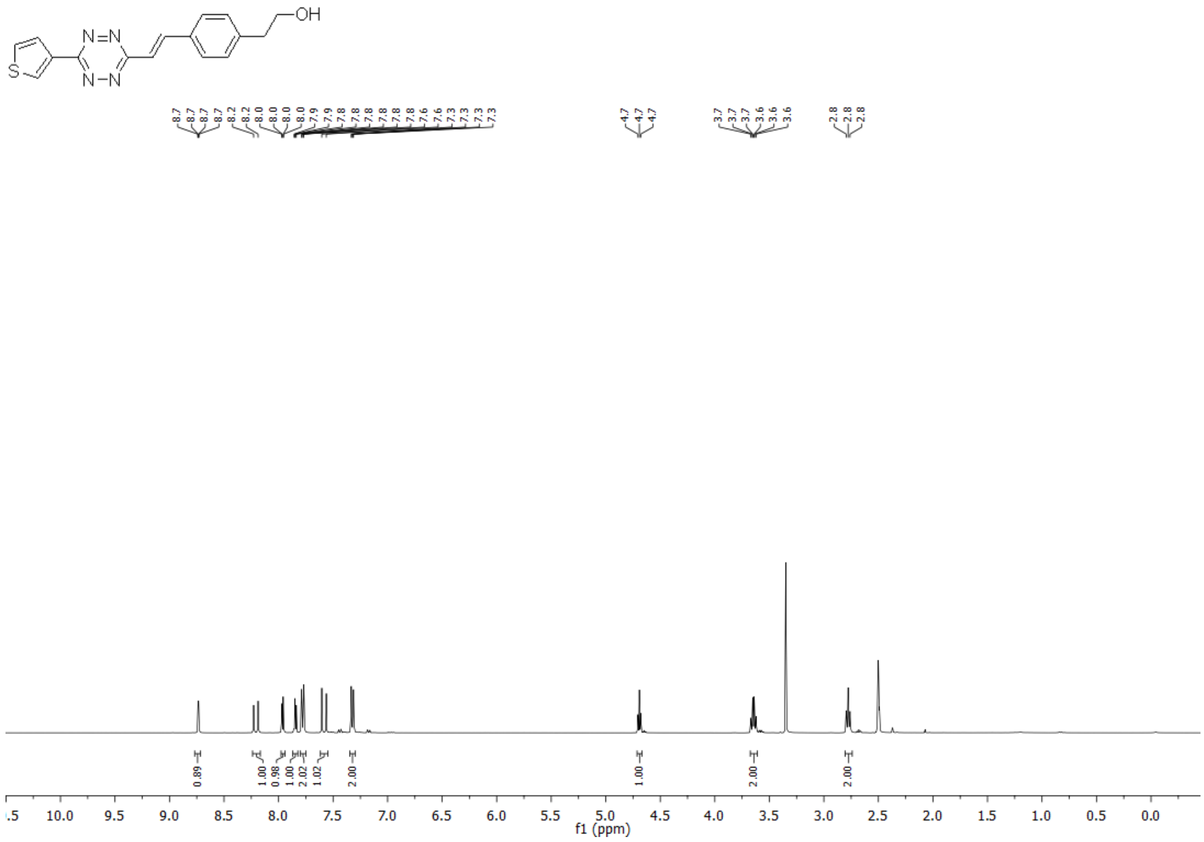
OH
f1 (ppm)

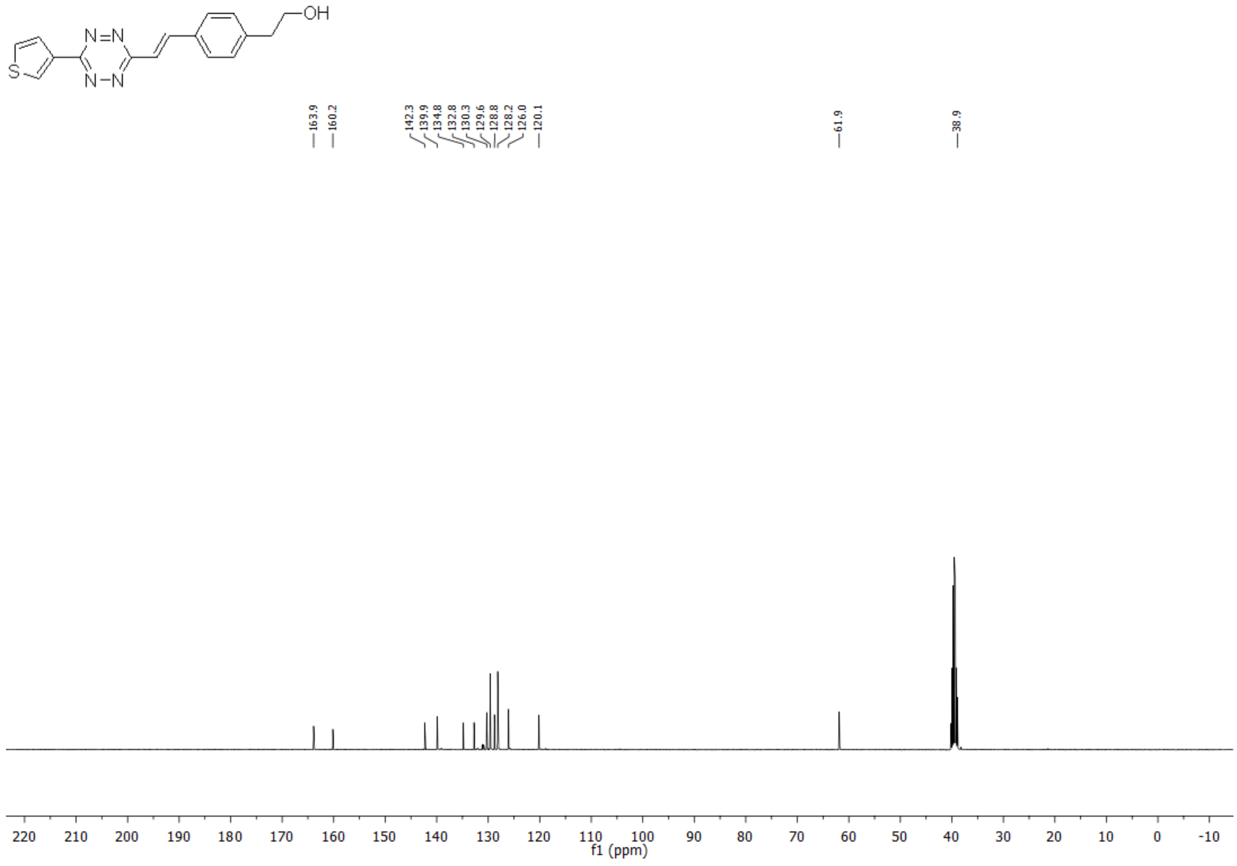
OH
163.9
160.2
142.3
139.9
134.8
132.8
130.3
129.6
128.8
128.2
126.0
120.1
61.9
38.9
f1 (ppm)

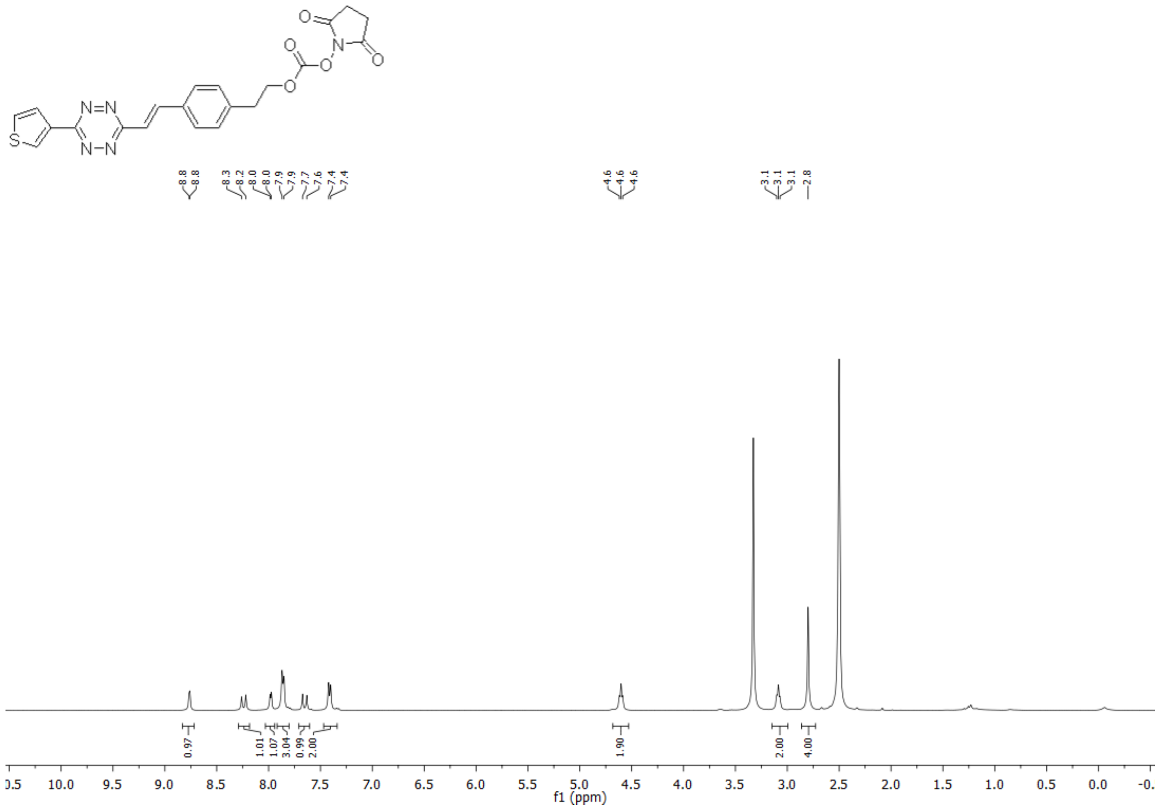
N=N
N–N
S
O
8.8 8.8 8.3 8.2 8.0 8.0 7.9 7.9 7.7 7.6 7.4 7.4
4.6 4.6 4.6
3.1 3.1 3.1 2.8
0.97 1.01 1.07 3.04 0.99 2.00
1.90
2.00 4.00
f1 (ppm)

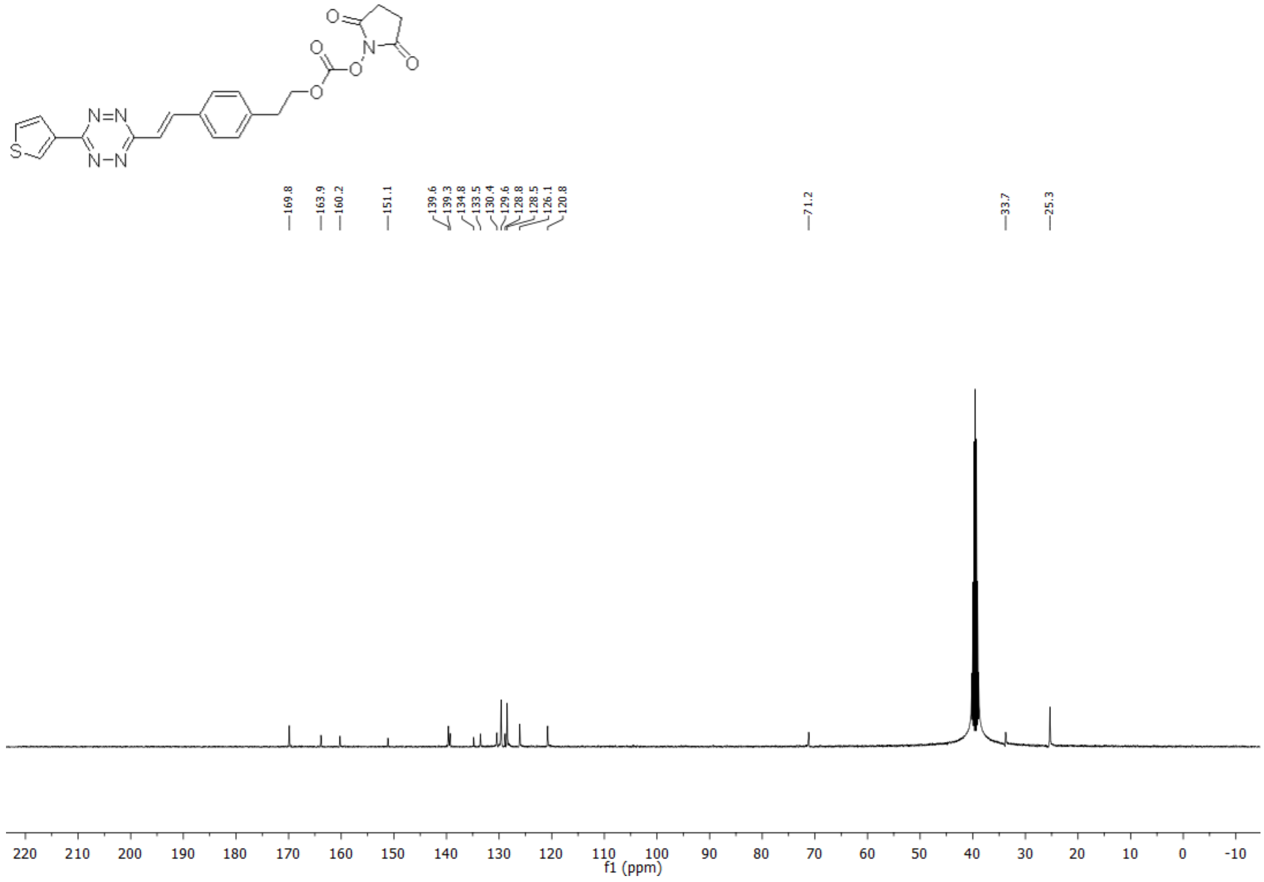
N=N
N–N
S
O
169.8 163.9 160.2 151.1 139.6 139.3 134.8 133.5 130.4 129.6 128.8 128.5 126.1 120.8
71.2
33.7 25.3
f1 (ppm)

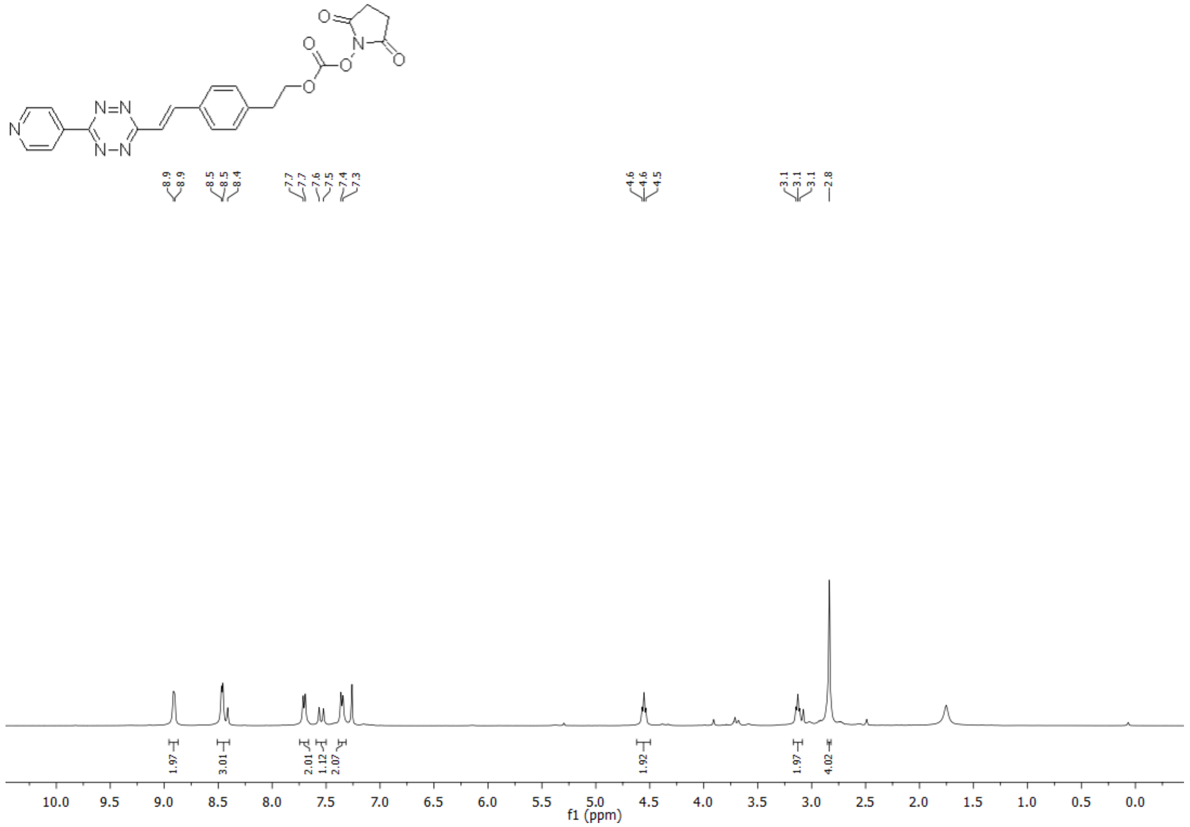
N=N
N–N
N
O
8.9
8.9
8.5
8.5
8.4
7.7
7.7
7.6
7.5
7.4
7.3
4.6
4.6
4.5
3.1
3.1
3.1
2.8
1.97
3.01
2.01
1.12
2.07
1.92
1.97
4.02
10.0 9.5 9.0 8.5 8.0 7.5 7.0 6.5 6.0 5.5 5.0 4.5 4.0 3.5 3.0 2.5 2.0 1.5 1.0 0.5 0.0
f1 (ppm)

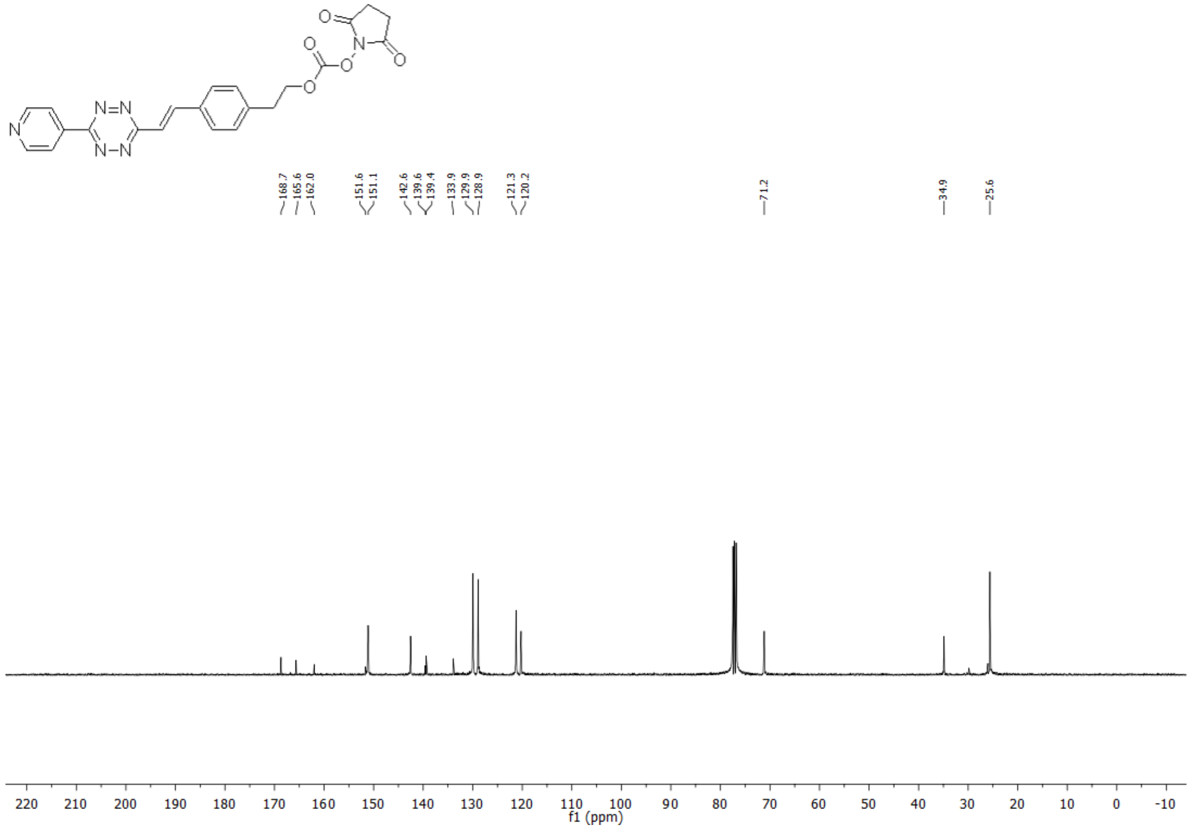
N=N
N–N
N
O
168.7
165.6
162.0
151.6
151.1
142.6
139.6
139.4
133.9
129.9
128.9
121.3
120.2
71.2
34.9
25.6
220 210 200 190 180 170 160 150 140 130 120 110 100 90 80 70 60 50 40 30 20 10 0 -10
f1 (ppm)

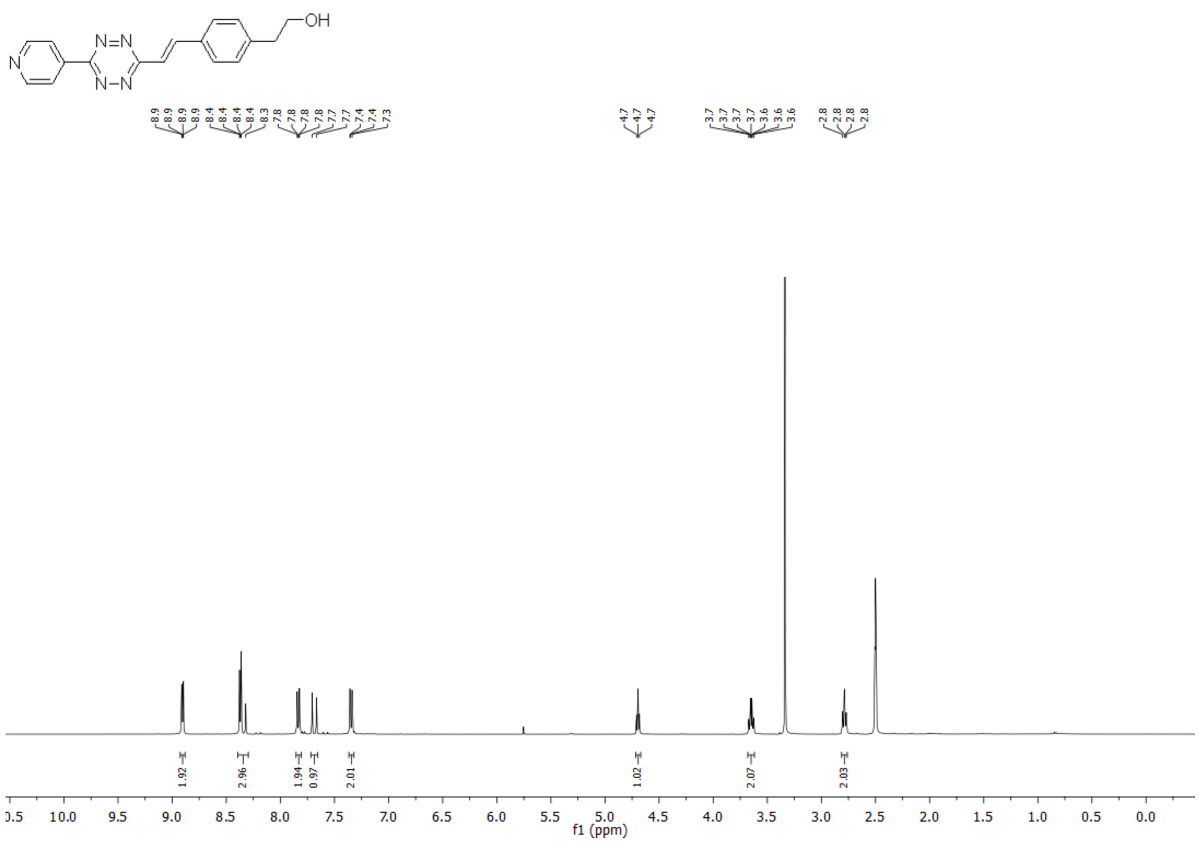
N
N=N
N–N
OH
8.9 8.9 8.9 8.9 8.4 8.4 8.4 8.4 8.3 7.8 7.8 7.8 7.8 7.7 7.7 7.4 7.4 7.3
4.7 4.7 4.7
3.7 3.7 3.7 3.7 3.6 3.6 3.6
2.8 2.8 2.8 2.8
1.92
2.96
1.94
0.97
2.01
1.02
2.07
2.03
f1 (ppm)

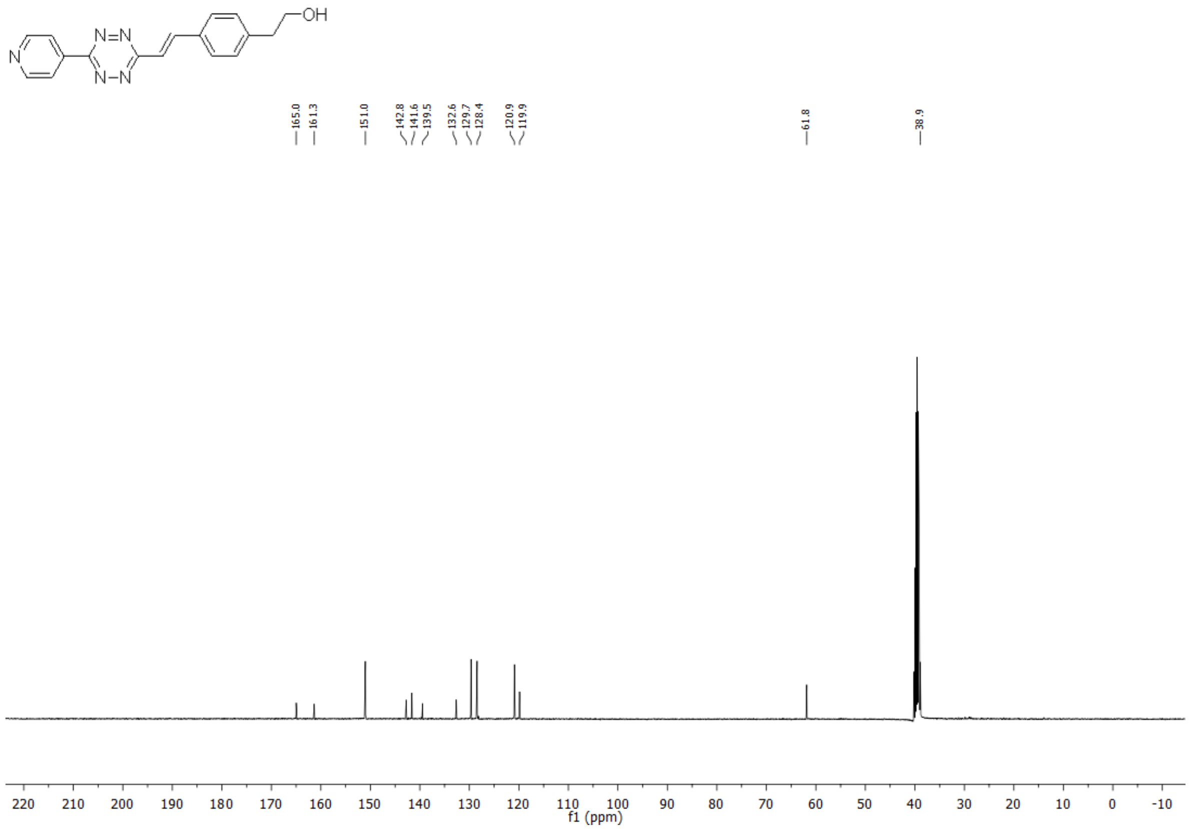
N
N=N
N–N
OH
165.0
161.3
151.0
142.8
141.6
139.5
132.6
129.7
128.4
120.9
119.9
61.8
38.9
f1 (ppm)

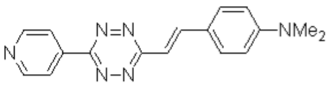
NMe2

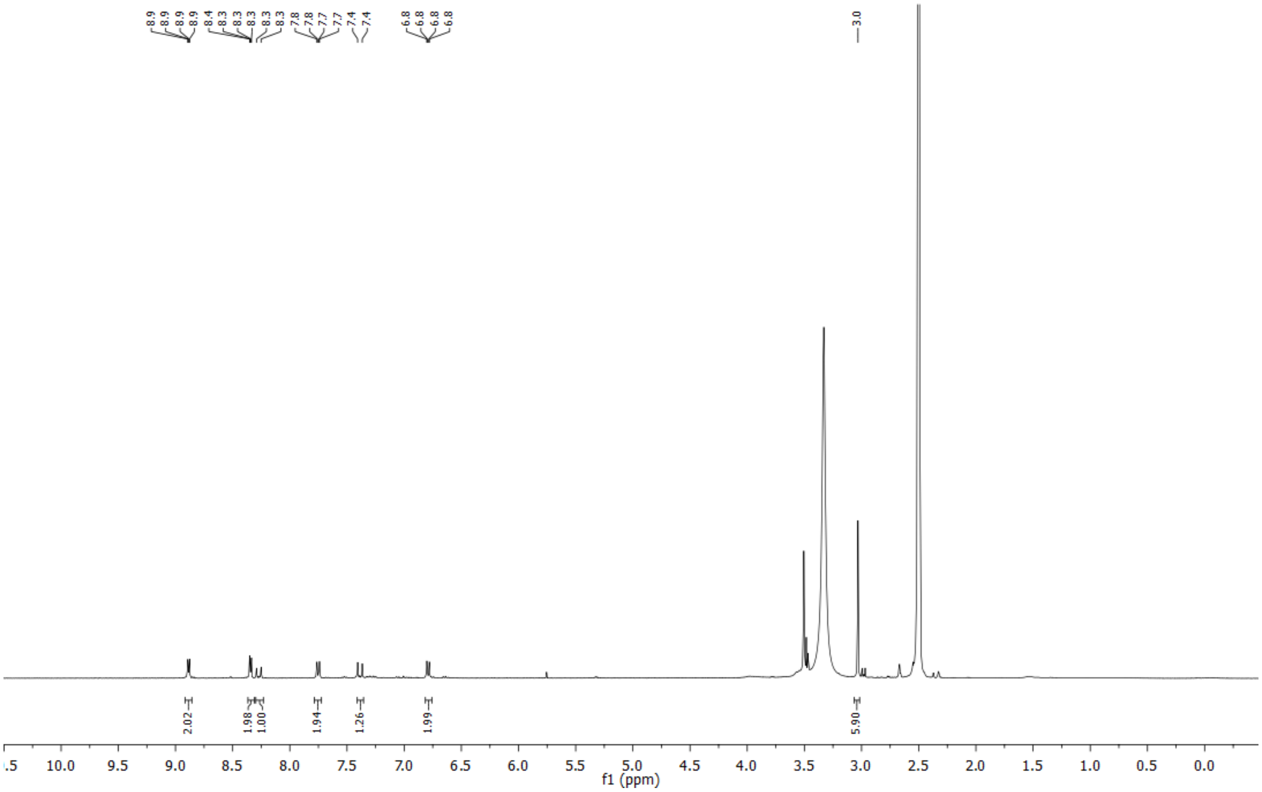
f1 (ppm)

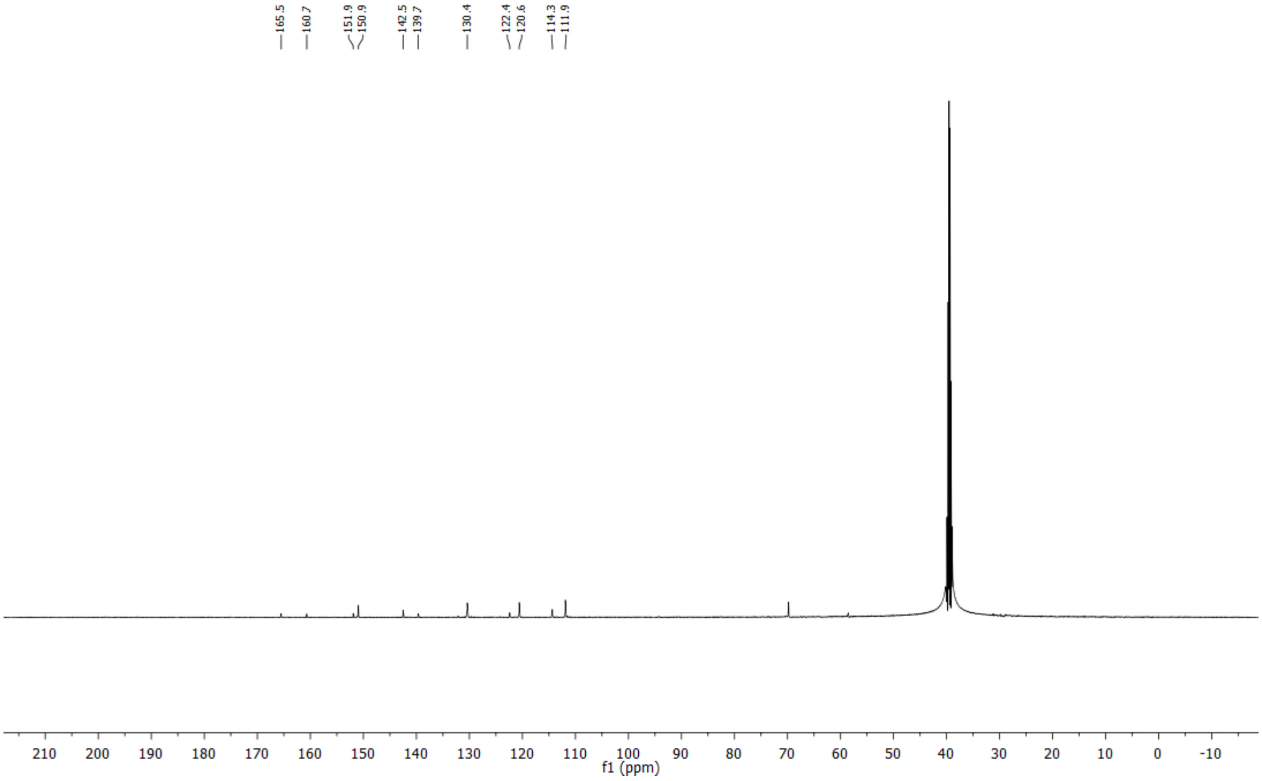
f1 (ppm)

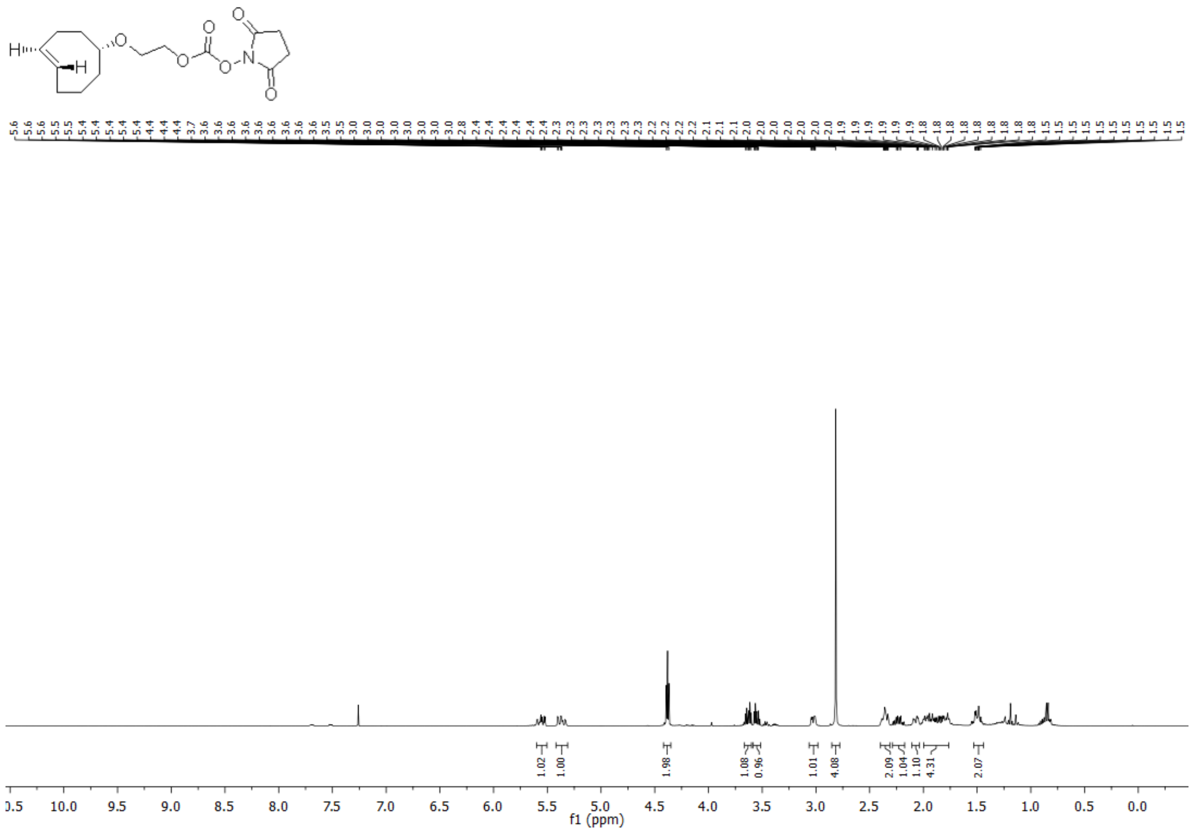

1.02
1.00
1.98
1.08
0.96
1.01
4.08
2.09
1.04
1.10
4.31
2.07
f1 (ppm)

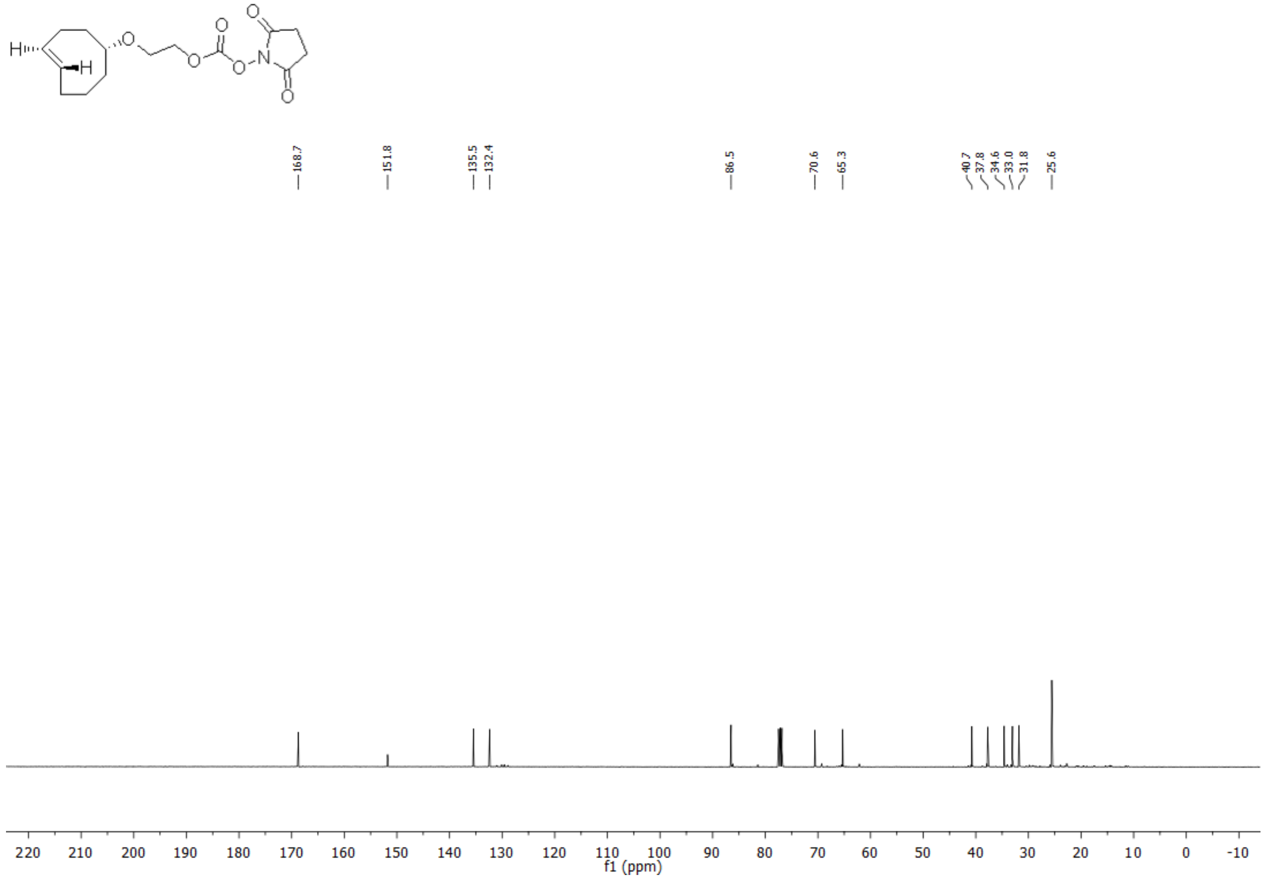

168.7
151.8
135.5
132.4
86.5
70.6
65.3
40.7
37.8
34.6
33.0
31.8
25.6
f1 (ppm)

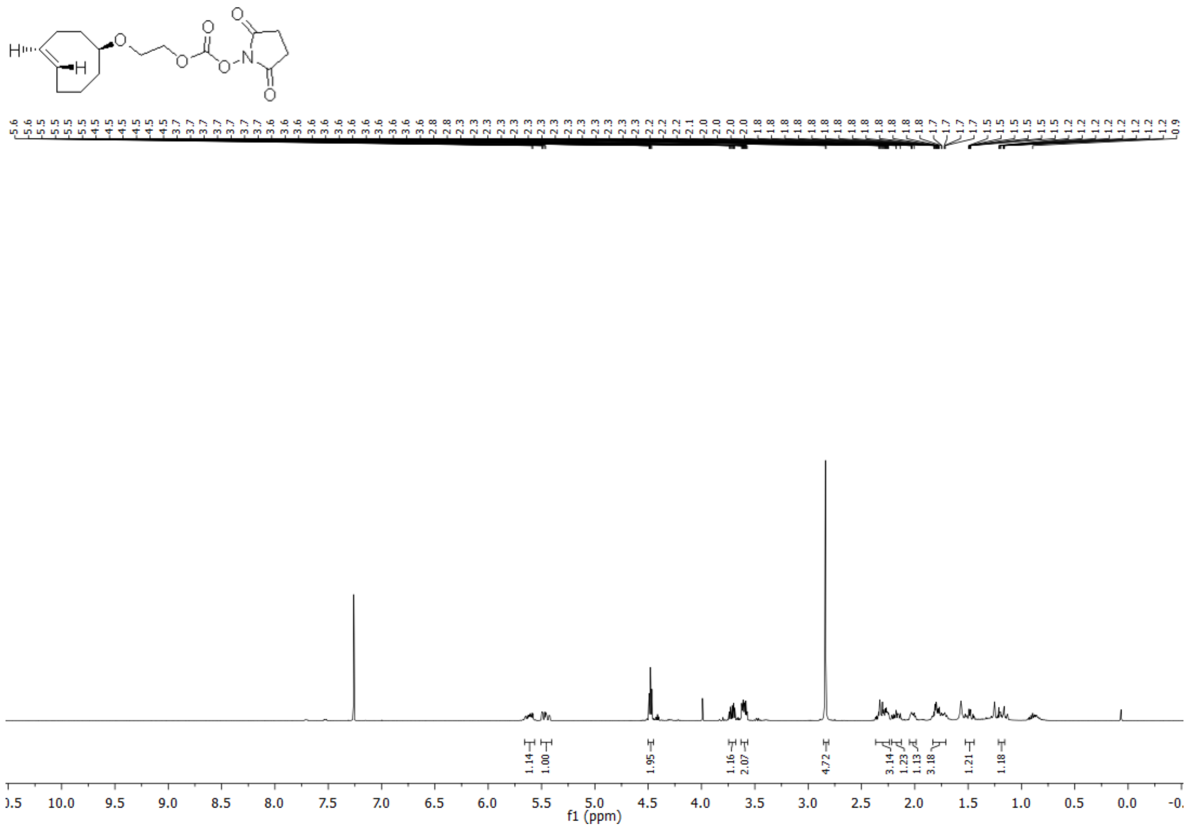
1.14
1.00
1.95
1.16
2.07
4.72
3.14
1.23
1.13
3.18
1.21
1.18
f1 (ppm)

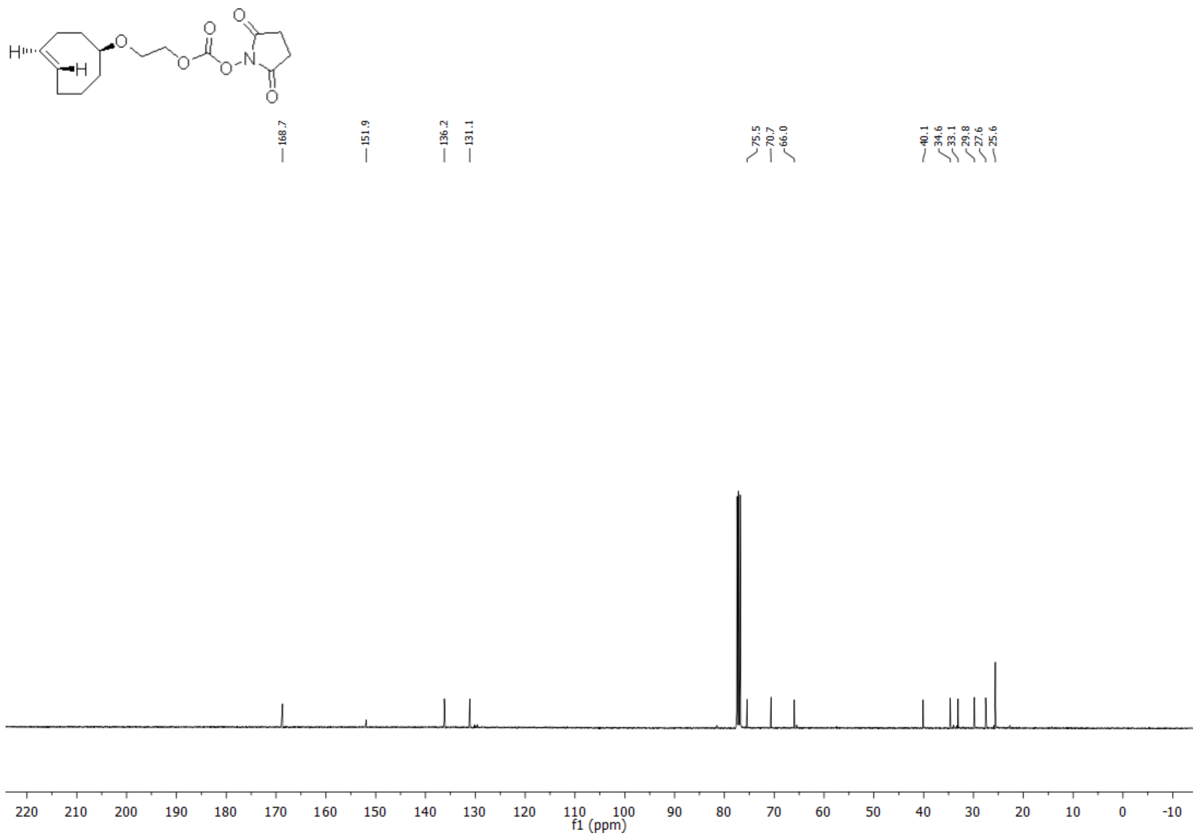
168.7
151.9
136.2
131.1
75.5
70.7
66.0
40.1
34.6
33.1
29.8
27.6
25.6
f1 (ppm)

References

[1] M. Royzen, G. P. Yap, J. M. Fox, *J. Am. Chem. Soc.* **2008**, *130*, 3760-3761.
[2] A. Darko, S. Wallace, O. Dmitrenko, M. M. Machovina, R. A. Mehl, J. W. Chin, J. M. Fox, *Chem. Sci.* **2014**, *5*, 3770-3776.
[3] M. T. Taylor, M. L. Blackman, O. Dmitrenko, J. M. Fox, *J. Am. Chem. Soc.* **2011**, *133*, 9646-9649.
[4] Y. Kurra, K. A. Odoi, Y. J. Lee, Y. Yang, T. Lu, S. E. Wheeler, J. Torres-Kolbus, A. Deiters, W. R. Liu, *Bioconjug. Chem.* **2014**, *25*, 1730-1738.
[5] Z. Li, H. Cai, M. Hassink, M. L. Blackman, R. C. Brown, P. S. Conti, J. M. Fox, *Chem. Commun.* **2010**, *46*, 8043-8045.
[6] H. Wu, J. Yang, J. Seckute, N. K. Devaraj, *Angew. Chem. Int. Ed.* **2014**, *53*, 5915-5919.
[7] J. A. Bull, J. J. Mousseau, A. B. Charette, *Org. Lett.* **2008**, *10*, 5485-5488.
[8] M. Millard, J. D. Gallagher, B. Z. Olenyuk, N. Neamati, *J. Med. Chem.* **2013**, *56*, 9170-9179.
[9] R. Guy, Z. Scott, R. Sloboda, K. Nicolaou, *Chem Biol* **1996**, *3*, 1021-1031.
[10] A. E. Carpenter, T. R. Jones, M. R. Lamprecht, C. Clarke, I. H. Kang, O. Friman, D. A. Guertin, J. H. Chang, R. A. Lindquist, J. Moffat, P. Golland, D. M. Sabatini, *Genome Biol.* **2006**, *7*, R100.
[11] D. F. Eaton, *J. Photochem. Photobiol. B* **1988**, *2*, 523-531.
[12] J. Schindelin, I. Arganda-Carreras, E. Frise, V. Kaynig, M. Longair, T. Pietzsch, S. Preibisch, C. Rueden, S. Saalfeld, B. Schmid, J. Y. Tinevez, D. J. White, V. Hartenstein, K. Eliceiri, P. Tomancak, A. Cardona, *Nat. Methods* **2012**, *9*, 676-682.
[13] a) A. D. Becke, *J. Chem. Phys.* **1993**, *98*, 5648-5652; b) C. T. Lee, W. T. Yang, R. G. Parr, *Phys. Rev. B* **1988**, *37*, 785-789.
[14] a) V. Barone, M. Cossi, *J. Phys. Chem. A* **1998**, *102*, 1995-2001; b) M. Cossi, N. Rega, G. Scalmani, V. Barone, *J. Comput. Chem.* **2003**, *24*, 669-681.
[15] M. J. Frisch, G. W. Trucks, H. B. Schlegel, G. E. Scuseria, M. A. Robb, J. R. Cheeseman, G. Scalmani, V. Barone, B. Mennucci, G. A. Petersson, H. Nakatsuji, X. Caricato, X. Li, H. P. Hratchian, A. F. Izmaylov, J. Bloino, G. Zheng, J. L. Sonnenberg, M. Hada, M. Ehara, K. Toyota, R. Fukuda, J. Hasegawa, M. Ishida, T. Nakajima, Y. Honda, O. Kitao, H. Nakai, T. Vreven, J. Montgomery, J. A., J. E. Peralta, F. Ogliaro, M. Bearpark, J. J. Heyd, E. Brothers, K. N. Kudin, V. N. Staroverov, R. Kobayashi, J. Normand, K. Raghavachari, A. Rendell, J. C. Burant, S. S. Iyengar, J. Tomasi, M. Cossi, N. Rega, J. M. Millam, M. Klene, J. E. Knox, J. B. Cross, V. Bakken, C. Adamo, J. Jaramillo, R. Gomperts, R. E. Stratmann, O. Yazyev, A. J. Austin, R. Cammi, C. Pomelli, J. W. Ochterski, R. L. Martin, K. Morokuma, V. G. Zakrzewski, G. A. Voth, P. Salvador, J. J. Dannenberg, S. Dapprich, A. D. Daniels, O. Farkas, J. B. Foresman, J. V. Ortiz, J. Cioslowski, D. J. Fox, Gaussian, Inc., Wallingford CT, **2009**.
[16] a) C. Peng, P. Y. Ayala, H. B. Schlegel, M. J. Frisch, *J. Comput. Chem.* **1996**, *17*, 49-56; b) C. Peng, H. B. Schlegel, *Israel J. Chem.* **1994**, *33*, 449-454.
